# Metabolomics of mouth-rinsed water for assessing psychophysiological stress in office workers

**DOI:** 10.1038/s41598-026-42241-4

**Published:** 2026-04-06

**Authors:** Yuki Maruyama, Kaoru Yamada, Takuya Inokuchi, Narumi Fujii, Ryosuke Kawamata, Yuko Ichiba, Yasushi Kakizawa, Masahiro Sugimoto, Akiyoshi Hirayama

**Affiliations:** 1 Research and Technology Center, Lion Corporation, 7-2-1 Hirai, Edogawa-Ku, Tokyo 132-0035 Japan; 2 Institute for Advanced Biosciences, Keio University, 246-2 Mizukami, Kakuganji, Tsuruoka, Yamagata 997-0052 Japan; 3 Systems Biology Program, Graduate School of Media and Governance, Keio University, Fujisawa, Kanagawa 252-0882 Japan

**Keywords:** Metabolomics, Occupational stress, Workplace, Biological markers, Saliva, Biochemistry, Biomarkers, Health care, Physiology

## Abstract

Workplace stress impacts productivity and health, necessitating non-invasive, rapid, and objective assessment methods. This study investigates the potential of metabolite profiling of mouth-rinsed water (MW)—an oral biofluid collected in just 10 s—as a screening tool for psychophysiological stress among office workers. Thirty-two participants were classified into high-stress and control groups based on the State-Trait Anxiety Inventory and the Brief Job Stress Questionnaire, and objective physiological measures. MW samples were collected at four time points, including before and after brief mental stress tasks. Biochemical features were profiled using capillary electrophoresis–mass spectrometry and liquid chromatography–mass spectrometry. A total of 559 analytes, including 532 water-soluble metabolites, 25 steroids, and 2 salivary proteins, were measured, of which 127 analytes with acceptable analytical precision were selected for subsequent analyses. Statistically significant differences in numerous metabolites indicated stress-associated alterations in metabolic activity. Notably, a predictive model using the ratio of *N*-acetyl-*β*-alanine to asymmetric dimethylarginine achieved high accuracy, with an area under the receiver operating characteristic curve (AUC) of 0.845 for identifying high stress. The distinct metabolite dynamics under varying stress conditions suggest multiple stress-response mechanisms. These findings underscore MW metabolite profiling as a promising approach for assessing and managing workplace stress.

**Supplementary Information:**

The online version contains supplementary material available at 10.1038/s41598-026-42241-4.

## Introduction

Stress is a major contributor to both mental and physical health issues, including depression and cardiovascular disease, and it significantly reduces workplace productivity, leading to substantial economic losses for individuals and organizations^1^. Consequently, accurate and objective stress assessment is essential. Although various screening methods, such as questionnaires and salivary biomarkers, have been proposed, they have both merits and demerits, and achieving a balance of objectivity, accuracy, and convenience remains a challenge^2,3^.

Salivary biomarkers are commonly studied tools for stress evaluation due to the non-invasive nature of saliva collection and the ability to obtain objective data compared with subjective measures, such as questionnaires^3^. However, a primary logistical challenge of saliva collection is the time required, typically around 5 min, which poses difficulties for simplicity in large-scale studies.

In addition to this practical issue, certain salivary components exhibit intrapersonal variation influenced by factors such as circadian rhythms^4^, posing challenges to both accuracy and simplicity. Furthermore, the complex nature of stress means that single biomarkers, such as cortisol, often fail to capture the full psychophysiological state, highlighting the need for multi-marker approaches. Therefore, an ideal evaluation method needs to be not only faster but also more comprehensive and stable.

Mouth-rinsed water—a sample that can be collected in just 10 s and imposes minimal burden on participants—has been shown to reliably reflect individual variability, comparable to resting and stimulated saliva^5^, and has also been evaluated for metabolites with circadian and sex-related variations^6^. The effectiveness of multi-biomarker strategies has been demonstrated in other fields^7^, suggesting their potential applicability to stress screening. Based on these findings, we hypothesized that metabolite profiling of mouth-rinsed water could accurately identify high-stress conditions in office workers and sought to identify a multi-biomarker set capable of capturing these states.

This study aimed to develop a novel stress assessment method using a multi-biomarker approach based on low molecular-weight metabolite profiling of mouth-rinsed water. To provide a scientific basis for defining the “high-stress state” underlying this approach, this study incorporated not only psychological assessments, which are subject to individual bias, but also objective physiological measurements, including functional near-infrared spectroscopy (fNIRS) and assessment of autonomic nervous system activity. Additionally, established stress markers, such as cortisol, dehydroepiandrosterone (DHEA), dehydroepiandrosterone sulfate (DHEA-S)^8,9^, secretory immunoglobulin A (sIgA), and chromogranin A (CgA)^9^, were quantified. This comprehensive design enhanced the validity of stress marker identification, and the obtained data were integrated to identify biomarker candidates, with known stress markers serving as controls to evaluate their performance.

## Results

### Comparison of profiles and psychophysiological indices between high-stress and control groups

**Fig. 1:**
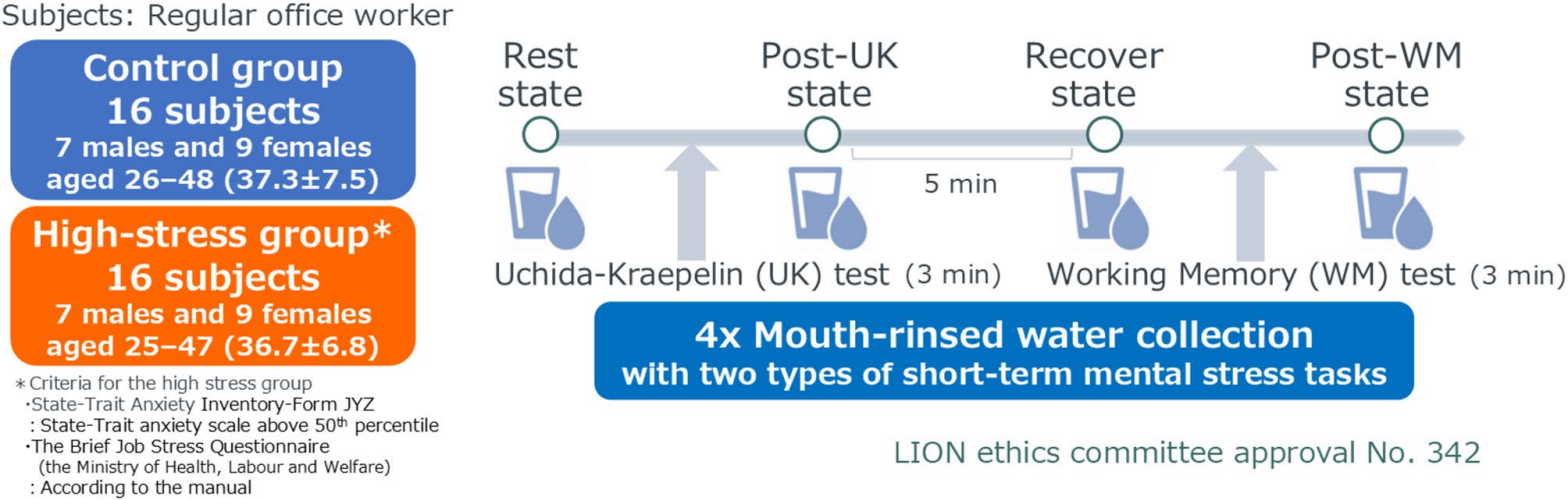
Clinical study design. A total of 32 participants were divided into a control group and a high-stress group, with 16 individuals in each group. Oral rinse samples were collected at four time points: (1) initial resting state (pre-task baseline, Rest), (2) post-Uchida–Kraepelin test (Post-UK), (3) second resting state (Recover), and (4) post-working memory task (Post-WM).

The study design is illustrated in Fig. 1, and Table 1 presents the profiles of the high-stress and control groups. The high-stress group was identified using two psychological measures. First, on the State-Trait Anxiety Inventory (STAI), which assesses both temporary situational anxiety (state) and long-standing anxious personality traits (trait), participants scored at or above the 50th percentile. Notably, all individuals in this group scored above the 70th percentile for state anxiety, indicating significant current distress. Second, all participants also met the criteria for high stress on the Brief Job Stress Questionnaire, a measure of occupational stress. The scores, which are detailed in Table S1, confirmed that all participants met the established national standards for identifying individuals at high risk for stress-related health problems. In contrast, the control group had scores well below these high-stress thresholds (Table 1).

**Table 1:** Group demographic and clinical characteristics.

Characteristics		Control group	High-stress group	*p*-value	*q*-value
Participants, *n* (males, females)		16 (7, 9)	16 (7, 9)	1.000	1.000
Age (years)		37.5 [30.8 – 45.0]	36.0 [31.8 – 41.5]	0.836	0.955
STAI (new)	State Anxiety (percentile) Trait Anxiety (percentile)	1.0 [0.0 – 4.3]	83.0 [81.0 – 89.5]	< 0.001	< 0.001
		2.5 [0.0 – 5.3]	91.0 [76.3 – 98.0]	< 0.001	< 0.001
BJSQ	Total score for area B	35.0 [31.8 – 38.8]	87.5 [77.8 – 98.5]	< 0.001	< 0.001
	Total combined score for areas A and C	49.0 [41.5 – 53.3]	70.5 [66.8 – 81.3]	< 0.001	< 0.001
	Total score for area B based on the raw score conversion table	27.5 [25.8 – 29.0]	10.5 [8.0 – 12.0]	< 0.001	< 0.001
	Total combined score for areas A and C based on the raw score conversion table	47.5 [44.0 – 54.0]	32.5 [27.0 – 34.3]	< 0.001	< 0.001

Data are presented as median [interquartile range (IQR)] or as n (males, females). There were no significant differences between the two groups in age or sex distribution (*q* > 0.05 for both; the Mann–Whitney U test for age, Fisher’s exact test for sex).

STAI, State-Trait Anxiety Inventory; BJSQ, Brief Job Stress Questionnaire.

Next, we compared psychophysiological indices between the high-stress and control groups. Physiological measures included blood pressure, body temperature, autonomic nervous system activity, and cerebral blood flow. Psychological assessment was based on the Profile of Mood States 2nd Edition (POMS 2). Subjective physical condition was evaluated using the Pittsburgh Sleep Quality Index (PSQI). As summarized in Table 2, statistically significant differences were observed in a range of these indices, including mood scores, cerebral blood flow, and autonomic activity, confirming that the two groups were in distinct psychophysiological states. Table 2 summarizes indices with significant differences, while Table S2 provides the complete dataset.

**Table 2:** Comparison of psychophysiological indicators between high-stress and control groups.

Factors	Control group	High-stress group	*p*-value	*q*-value
POMS 2_T	37.5 [35.0 – 39.3]	49.0 [42.0 – 62.0]	< 0.001	0.015
POMS 2_TMD	− 6.0 [-10.5 – -0.8]	18.0 [2.8 – 43.0]	< 0.001	0.015
POMS 2_CB	0.5 [0.0 – 1.0]	2.5 [1.0 – 10.0]	< 0.001	0.024
POMS 2_F	14.5 [11.8 – 15.3]	8.5 [7.8 – 11.0]	0.001	0.024
POMS 2_TA	2.0 [1.0 – 3.3]	6.0 [3.8 – 9.0]	0.001	0.024
POMS 2_VA	12.0 [10.0 – 15.0]	5.5 [4.0 – 9.3]	0.001	0.033
POMS 2_FI	2.5 [1.8 – 3.3]	7.0 [3.8 – 13.0]	0.001	0.033
POMS 2_DD	0.0 [0.0 – 1.0]	3.0 [0.0 – 7.8]	0.002	0.065
POMS 2_AH	1.0 [0.0 – 2.0]	3.5 [1.0 – 7.3]	0.006	0.137
Initial Activation_13CH_UK	0.0202 [0.00334 – 0.0331]	8.02 × 10⁻^4^ [-9.71 × 10⁻^3^ – 4.25 × 10⁻^3^]	0.010	0.207
PSQIG	3.0 [2.0 – 4.0]	5.0 [3.8 – 6.3]	0.014	0.263
Initial Activation_10CH_WM	0.00493 [0.00276 – 0.00655]	− 6.66 × 10⁻^4^ [− 2.50 × 10⁻^3^ – 2.94 × 10⁻^3^]	0.018	0.325
BodyTemp(°C)	36.8 [36.2 – 36.8]	36.3 [36.1 – 36.5]	0.029	0.456
SDNN_Recover	52.5 [41.0 – 63.6]	38.2 [28.0 – 45.6]	0.030	0.456
Gravity_Range2_12CH_UK	129.5 [123.3 – 142.5]	151.0 [133.8 – 161.3]	0.038	0.536

Data are presented as median [interquartile range (IQR)]. Statistical significance was determined using the Mann–Whitney U test.

POMS 2, Profile of Mood States Second Edition; T, Total Score; TMD, Total Mood Disturbance; CB, Confusion-Bewilderment; F, Friendliness; TA, Tension-Anxiety; VA, Vigor-Activity; FI, Fatigue-Inertia; DD, Depression-Dejection; AH, Anger-Hostility; UK, Uchida–Kraepelin test; WM, working memory test; PSQI, Pittsburgh Sleep Quality Index; SDNN, standard deviation of all normal-to-normal intervals.

### Overall analysis of metabolite profiles in mouth-rinsed water from high-stress and control groups

**Fig. 2:**
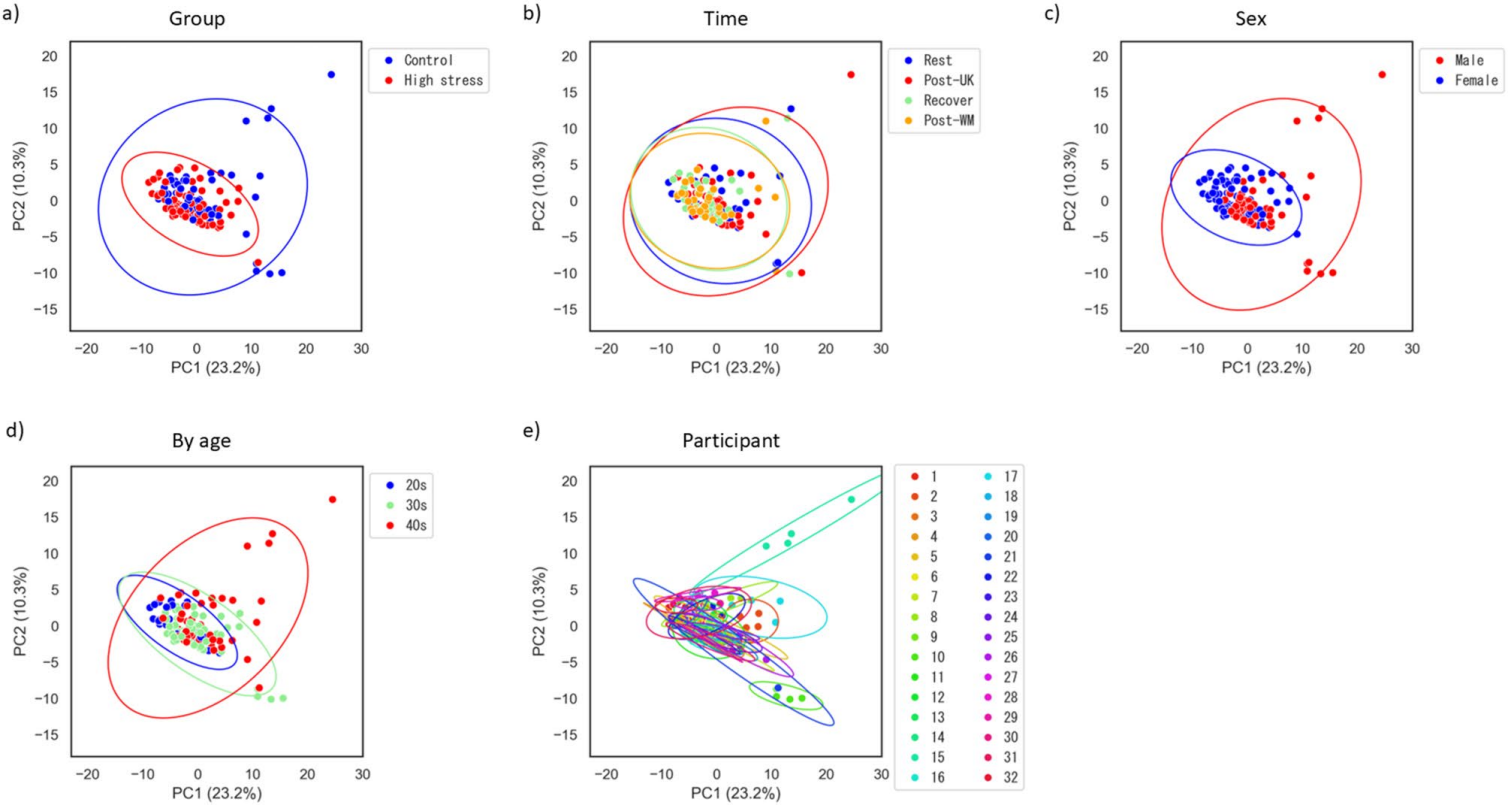
Principal component analysis (PCA) score plot. Each point is an individual sample, colored by (**a**) group, (**b**) time, (**c**) sex, (**d**) age, and (**e**) participant. Ellipses represent the 95% confidence regions for each group. The horizontal and vertical axes represent the first and second principal components (PC1 and PC2), respectively.

To characterize metabolite profiles of the mouth-rinsed water from the high-stress and control groups, principal component analysis and clustering were performed on 128 samples. The analysis included a total of 559 analytes, comprising 532 water-soluble metabolites detected by capillary electrophoresis-mass spectrometry (CE-MS), 25 steroids detected by liquid chromatography-mass spectrometry (LC–MS), and 2 salivary proteins (sIgA and CgA). Additionally, sex and age were evaluated as covariates. Regarding the 532 water-soluble metabolites, applying quality criteria based on CE-MS validation—recovery rate within 100 ± 30% and relative standard deviation (RSD) ≤ 20%—reduced the features to 310. Further filtering of components detected in at least 50% of samples in either group yielded 100 robust metabolites. These were combined with the steroids and salivary proteins, resulting in a total of 127 biochemical features for the final statistical analysis. The measured values of these 127 features were standardized by z-score before the subsequent multivariate analyses. The resulting score plot and heat map with clustering are presented in Fig. 2 and Figure S1, respectively.

Score plots analyzed by group, collection timing, sex, and age showed some data points outside the 95% confidence ellipse. However, when analyzed by individual participants, all data points for each participant fell within the respective 95% confidence ellipses, indicating that these outliers reflect individual variability rather than errors and were therefore retained. Therefore, keeping these data points was considered essential for identifying biomarker candidates that capture group trends and stress responses over and above this individual variability. Although no clear separation was observed between groups or time points, a certain degree of separation by sex and age was observed, suggesting that individual differences are the dominant factor.

### Comparative analysis between the high-stress group and the control group

To identify chronic stress markers unaffected by short-term mental stress, inter-group significance tests were conducted on the 127 selected variables across all 128 samples. The Mann–Whitney U test with Benjamini–Hochberg correction was used for multiple comparisons. The analysis was performed on data from all four collection points—including rest and before and after the two stress tasks—and on median values across these points. The *p*-value indicated the uncorrected significance, while the *q*-value indicated the corrected significance. Although 24 features showed statistical significance by *p*-value at individual time points, none remained significant after correction (*q*-value) (Table S3 and Table S4).

### Longitudinal analysis at the four time points, including short-term mental stress load and recovery process

Samples were collected at four time points: initial rest (Rest), immediately after the Uchida–Kraepelin test (Post-UK), after a 5-min rest following the Uchida–Kraepelin test (Recover), and after the working memory test (Post-WM). Temporal changes in each metabolite were analyzed using the Friedman test. In the combined group (control and high stress), 68 metabolites showed significant changes after multiple comparison correction (*q* < 0.05). When analyzed by group, 15 metabolites in the control group and 43 metabolites in the high-stress group exhibited significant differences (Table S5, Table S6).

The number of metabolites showing significant changes between time points was calculated. Table S7 lists all metabolites showing statistically significant changes (*q* < 0.05, Nemenyi test) between time points. Table S8 highlights a subset of these, focusing on those exhibiting a large magnitude of change (30% or more). Across all participants, 7 metabolites (the highest number) changed between the Post-UK and Recover stages, with 6 decreasing during this interval. In the control group, the most significant change occurred between Rest and Post-UK, involving 3 metabolites—including sIgA—all of which increased. In the high-stress group, 15 metabolites changed significantly between Post-UK and Post-WM, with all decreasing over this period.

### Developing a predictive model for high-stress markers

We attempted to develop a predictive model for identifying high-stress states using 127 metabolite variables from 128 samples. Initially, models combining feature selection methods (forward–backward stepwise selection, L1 regularization, and Boruta) with logistic regression were evaluated. Evaluation using a combination of double cross-validation with Leave-One-Out Cross-Validation (LOOCV) and the bootstrap method revealed that the selected features varied widely across the folds, and no predictive model with good generalization performance could be developed. Subsequently, single-variable threshold models were constructed for each metabolite, with thresholds set by the Youden index and performance assessed via LOOCV and bootstrapping. Although some features achieved an average area under the curve (AUC) above 0.7, their accuracy and F1 score confidence intervals (CIs) were below 0.5, indicating insufficient predictive accuracy for single-variable models (Table 3). Similar evaluations of known salivary stress markers—sIgA, CgA, cortisol, DHEA, and DHEA-S—yielded average AUCs ranging from 0.543 to 0.740. However, except for DHEA at the Post-WM time point, the lower limit of the 95% CI for the AUC was below 0.5 (Table S9). These markers did not show adequate predictive performance in identifying the high-stress group in this study.

**Table 3:** Predictive performance of the top-ranked single-metabolite models.

Time Point	Metabolite	AUC [95% CI]	Accuracy [95% CI]	F1 Score [95% CI]	Association with High Stress
Rest	AICAR	0.688 [0.542 – 0.825]	0.693 [0.531 – 0.829]	0.576 [0.318 – 0.800]	↑
	Ile	0.688 [0.513 – 0.870]	0.667 [0.359 – 0.875]	0.708 [0.353 – 0.900]	↓
	Agmatine	0.680 [0.491 – 0.833]	0.636 [0.375 – 0.829]	0.595 [0.105 – 0.846]	↑
Post-UK	Tetrahydrocortisone	0.747 [0.525 – 0.901]	0.706 [0.359 – 0.875]	0.710 [0.146 – 0.895]	↓
	25 – OHD	0.746 [0.279 – 0.953]	0.705 [0.375 – 0.906]	0.730 [0.448 – 0.914]	↑
	Androstenediol	0.734 [0.543 – 0.889]	0.723 [0.498 – 0.860]	0.702 [0.425 – 0.850]	↓
Recover	Estrone	0.794 [0.590 – 0.933]	0.762 [0.563 – 0.891]	0.757 [0.463 – 0.914]	↓
	Hypoxanthine	0.729 [0.578 – 0.849]	0.683 [0.421 – 0.906]	0.700 [0.444 – 0.916]	↓
	AICAR	0.723 [0.565 – 0.861]	0.694 [0.515 – 0.829]	0.629 [0.259 – 0.811]	↑
Post-WM	*N*^6^*,N*^6^*,N*^6^-trimethyllysine	0.778 [0.638 – 0.921]	0.743 [0.469 – 0.891]	0.702 [0.462 – 0.874]	↓
	Choline	0.741 [0.559 – 0.881]	0.737 [0.515 – 0.875]	0.765 [0.571 – 0.911]	↓
	DHEA	0.740 [0.540 – 0.868]	0.692 [0.469 – 0.844]	0.698 [0.448 – 0.875]	↓
All	Choline	0.691 [0.537 – 0.809]	0.645 [0.457 – 0.766]	0.671 [0.446 – 0.799]	↓
	Ile	0.681 [0.499 – 0.855]	0.615 [0.370 – 0.778]	0.672 [0.378 – 0.834]	↓
	Estrone	0.666 [0.537 – 0.780]	0.607 [0.387 – 0.754]	0.595 [0.380 – 0.756]	↓

The table shows the performance of predictive models constructed using each metabolite as a single feature. The top 3 models at each time point, ranked by the highest area under the receiver operating characteristic curve (AUC), are presented.

Association with High Stress: ↑ indicates that higher values are associated with the high-stress group; ↓ indicates that lower values are associated with the high-stress group.

UK, Uchida-Kraepelin test; WM, working memory test; CI, confidence interval.

25-OHD, 25-hydroxyvitamin D; AICAR, 5-aminoimidazole-4-carboxamide ribonucleotide; DHEA, dehydroepiandrosterone; Ile, isoleucine.

**Fig. 3:**
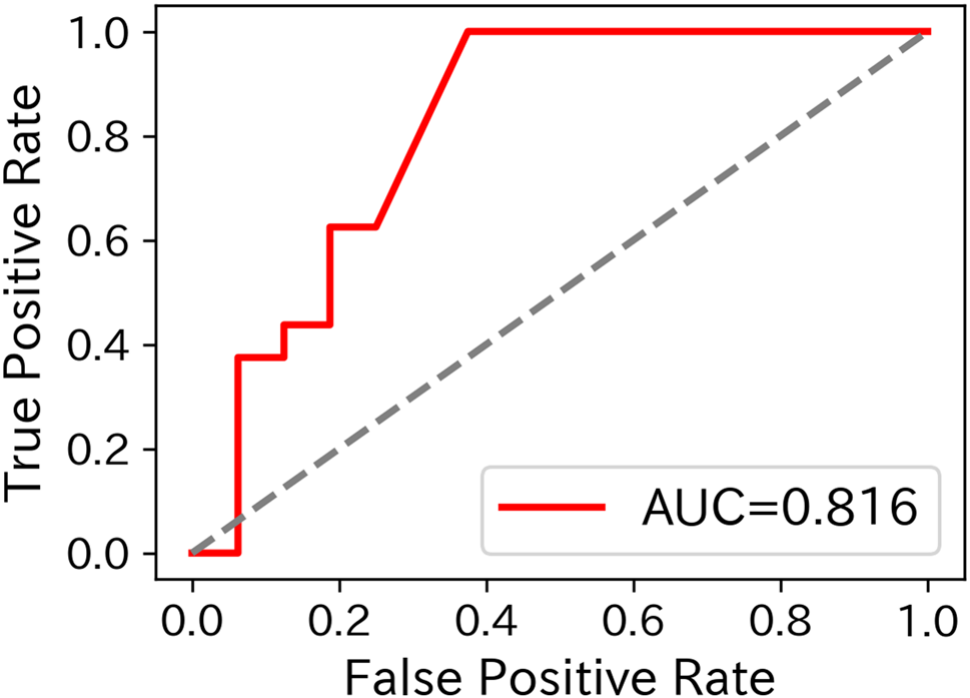
Receiver operating characteristic (ROC) curve for the high-stress prediction model using the *N*-acetyl-*β*-alanine/ADMA ratio, constructed from resting state data. The ROC curve illustrates the relationship between sensitivity (true positive rate) and 1-specificity (false positive rate) across different threshold settings. The x-axis represents the false positive rate, while the y-axis indicates the true positive rate. The area under the curve (AUC) serves as an indicator of predictive accuracy. A higher AUC reflects better model performance. The AUC value (0.816) shown in the figure represents the performance on the entire dataset and is distinct from the cross-validated performance.

Therefore, the predictive model was developed using the ratio of two metabolite variables (A/B) as features, evaluated with LOOCV and bootstrapping. Several metabolite pairs demonstrated strong predictive performance, achieving AUCs above 0.8 and lower 95% confidence limits for accuracy and F1 score exceeding 0.5. However, the optimal metabolite pair and its accuracy varied by collection time, with no pair consistently performing well across all time points. The best-performing model was based on the* N*-acetyl-*β*-alanine/ Asymmetric dimethylarginine (ADMA) ratio from resting samples, achieving an AUC of 0.845 (95% CI: 0.669 – 0.971; *p* = 0.042), an F1 score of 0.833 (95% CI: 0.667 – 0.952), and an accuracy of 0.815 (95% CI: 0.640 – 0.938). The receiver operating characteristic (ROC) curve (AUC = 0.816) for the entire dataset is shown in Fig. 3.

**Fig. 4:**
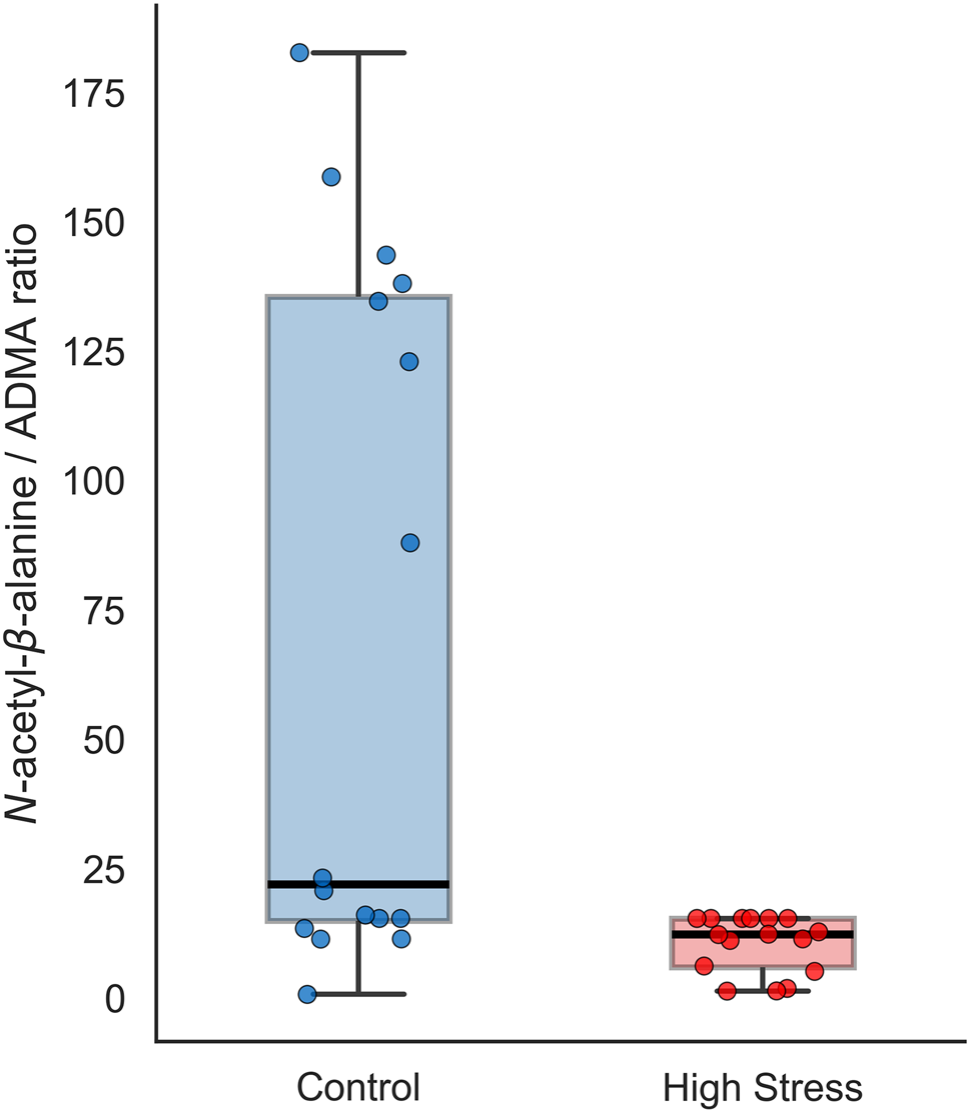
Distribution of the *N*-acetyl-*β*-alanine/ADMA ratio at rest. Box plots show the median (central horizontal line) and interquartile range (box boundaries); whiskers indicate 1.5 × interquartile range (IQR). Each dot represents an individual sample (Control group, n = 16; High-stress group, n = 16). A significant decrease in the ratio was observed in the High-stress group compared to the Control group (*p* < 0.05, Mann–Whitney U test). ADMA, asymmetric dimethylarginine.

When this model was applied to data from other time points, the F1 scores for Post-UK, Recover, and Post-WM were 0.55, 0.68, and 0.69, respectively, and the accuracy was 0.44, 0.59, and 0.66, respectively. Detailed results and model performance across time points are presented in Table 4 (top performers) and Table S10 (full results). Figure 4 illustrates the distribution of the *N*-acetyl-*β*-alanine/ADMA ratio in the pooled cohort (all participants combined). To further assess the potential influence of sex differences on the study results, we conducted both stratified and covariate-adjusted analyses. A sex-stratified analysis demonstrated a consistent decreasing trend of the *N*-acetyl-*β*-alanine/ADMA ratio in the high-stress group for both males and females (Supplementary Figure S2). To evaluate the potential confounding effects of sex and age, logistic regression analyses were performed with sex and age group included as covariates. In this model, neither sex nor age group was a statistically significant covariate (all *p*-values > 0.05; Table S11). The unadjusted odds ratio (OR) for the *N*-acetyl-*β*-alanine/ADMA ratio was 1.84 × 10⁻^3^ (95% CI: 1.56 × 10⁻⁶–2.15). After adjustment for sex and age group, the OR was 1.00 × 10⁻^4^ (95% CI: 1.40 × 10⁻⁸–0.714), retaining the same direction and a comparable magnitude to that observed in the unadjusted model, suggesting that the association was not materially confounded by sex or age group.

**Table 4:** Predictive performance of the metabolite ratio-based model.

Time Point	Ratio	AUC [95% CI]	Accuracy [95% CI]	F1 Score [95% CI]	Association with High Stress
Rest	*N*-acetyl-*β*-alanine/ADMA	0.845 [0.669 – 0.971]	0.815 [0.640 – 0.938]	0.833 [0.667 – 0.952]	↓
	*N*-acetyl-*β*-alanine/7-Methylguanine	0.829 [0.678 – 0.955]	0.777 [0.576 – 0.906]	0.785 [0.558 – 0.917]	↓
	Ile/7-Methylguanine	0.793 [0.648 – 0.936]	0.768 [0.577 – 0.906]	0.770 [0.586 – 0.929]	↓
	Glycerate/6-Phosphogluconate	0.787 [0.607 – 0.907]	0.744 [0.546 – 0.891]	0.749 [0.544 – 0.912]	↓
	Val/ADMA	0.783 [0.628 – 0.946]	0.759 [0.563 – 0.923]	0.743 [0.527 – 0.925]	↓
	7-Methylguanine/Val	0.771 [0.623 – 0.935]	0.741 [0.563 – 0.938]	0.757 [0.519 – 0.951]	↑
	His/Glu	0.760 [0.614 – 0.896]	0.729 [0.531 – 0.875]	0.707 [0.531 – 0.889]	↑
Post-UK	Progesterone/25-OHD	0.835 [0.630 – 0.968]	0.825 [0.625 – 0.954]	0.817 [0.604 – 0.956]	↓
	Thr/25-OHD	0.833 [0.639 – 0.986]	0.801 [0.594 – 0.954]	0.796 [0.586 – 0.959]	↓
	25-OHD/Inosine	0.833 [0.628 – 0.982]	0.778 [0.546 – 0.954]	0.777 [0.533 – 0.960]	↑
	25-OHD/Adenine	0.827 [0.624 – 0.975]	0.781 [0.531 – 0.938]	0.775 [0.522 – 0.946]	↑
	Hypoxanthine/25-OHD	0.827 [0.648 – 0.962]	0.826 [0.688 – 0.969]	0.823 [0.664 – 0.966]	↓
	Gln/25-OHD	0.826 [0.647 – 0.949]	0.776 [0.577 – 0.906]	0.767 [0.505 – 0.917]	↓
	25-OHD/Adenosine	0.815 [0.622 – 0.950]	0.763 [0.531 – 0.906]	0.754 [0.530 – 0.907]	↑
	1,3-Diaminopropane/25-OHD	0.807 [0.646 – 0.958]	0.746 [0.513 – 0.923]	0.757 [0.516 – 0.932]	↓
	25-OHD / *β*-Ala	0.802 [0.616 – 0.963]	0.756 [0.563 – 0.923]	0.759 [0.579 – 0.923]	↑
	11-Deoxycorticosterone/25-OHD	0.801 [0.605 – 0.957]	0.755 [0.546 – 0.891]	0.770 [0.563 – 0.911]	↓
	Tetrahydrocortisol/Homovanillate	0.801 [0.634 – 0.939]	0.738 [0.546 – 0.875]	0.758 [0.584 – 0.898]	↓
	Glu/25-OHD	0.800 [0.631 – 0.935]	0.780 [0.563 – 0.923]	0.769 [0.509 – 0.929]	↓
	Leu/25-OHD	0.794 [0.609 – 0.945]	0.744 [0.515 – 0.891]	0.752 [0.593 – 0.916]	↓
	Tetrahydrocortisol/Lithocholic acid	0.785 [0.651 – 0.940]	0.746 [0.594 – 0.906]	0.754 [0.503 – 0.925]	↓
Recover	Ser/His	0.806 [0.646 – 0.962]	0.801 [0.640 – 0.938]	0.792 [0.613 – 0.949]	↓
	Glu/His	0.805 [0.652 – 0.945]	0.821 [0.640 – 0.938]	0.829 [0.636 – 0.943]	↓
	Tyr/Val	0.794 [0.627 – 0.925]	0.748 [0.531 – 0.906]	0.756 [0.517 – 0.918]	↑
	His/Estrone	0.786 [0.628 – 0.912]	0.756 [0.563 – 0.906]	0.749 [0.542 – 0.921]	↑
	His/*N*-acetylglucosamine 6-phosphate	0.779 [0.625 – 0.920]	0.752 [0.563 – 0.891]	0.723 [0.533 – 0.900]	↑
	Tyr/Estrone	0.777 [0.611 – 0.919]	0.777 [0.594 – 0.923]	0.777 [0.547 – 0.922]	↑
	Tyr/Ser	0.774 [0.603 – 0.907]	0.774 [0.577 – 0.906]	0.747 [0.535 – 0.907]	↑
	Trp/AICAR	0.769 [0.611 – 0.912]	0.750 [0.594 – 0.906]	0.751 [0.540 – 0.916]	↓
	Glycerate/*N*-acetylglucosamine 6-phosphate	0.762 [0.613 – 0.913]	0.792 [0.671 – 0.906]	0.775 [0.618 – 0.918]	↑
Post-WM	4-Methyl-2-oxopentanoate/*N*^6^,*N*^6^,*N*^6^-trimethyllysine	0.867 [0.735 – 0.981]	0.807 [0.656 – 0.938]	0.786 [0.582 – 0.939]	↑
	Ile/*N*-*ε*-acetyllysine	0.849 [0.727 – 0.952]	0.788 [0.577 – 0.938]	0.785 [0.538 – 0.947]	↓
	Val/*N*-*ε*-acetyllysine	0.834 [0.685 – 0.968]	0.821 [0.688 – 0.938]	0.822 [0.677 – 0.956]	↓
	4-Methyl-2-oxopentanoate/Val	0.834 [0.686 – 0.950]	0.764 [0.531 – 0.906]	0.762 [0.508 – 0.932]	↑
	Agmatine/* N*^6^,*N*^6^,*N*^6^-trimethyllysine	0.831 [0.705 – 0.941]	0.751 [0.563 – 0.906]	0.747 [0.539 – 0.909]	↑
	His/* N*^6^,*N*^6^,*N*^6^-trimethyllysine	0.828 [0.700 – 0.946]	0.771 [0.515 – 0.906]	0.758 [0.524 – 0.925]	↑
	Leu/*N-ε*-acetyllysine	0.820 [0.686 – 0.961]	0.815 [0.625 – 0.938]	0.818 [0.583 – 0.951]	↓
	Cortisol/*N*-acetylglucosamine 1-phosphate	0.807 [0.655 – 0.918]	0.767 [0.546 – 0.906]	0.762 [0.516 – 0.909]	↓
	2AB/*N-ε*-acetyllysine	0.805 [0.656 – 0.959]	0.832 [0.688 – 0.938]	0.829 [0.664 – 0.949]	↓
All	4-Methyl-2-oxopentanoate/Choline	0.724 [0.563 – 0.850]	0.684 [0.523 – 0.789]	0.703 [0.501 – 0.821]	↑
	Choline/Azelate	0.653 [0.514 – 0.773]	0.671 [0.550 – 0.789]	0.720 [0.573 – 0.834]	↓

The table shows the top-performing models, which were selected if the lower bound of the 95% confidence interval for their performance metrics exceeded the following thresholds: AUC > 0.60, Accuracy > 0.50, and F1 Score > 0.50 (for Post-WM: AUC > 0.65; for All time points: AUC > 0.50).

Association with High Stress: ↑ indicates that a higher model output is associated with the high-stress group; ↓ indicates that a higher model output is associated with the control group.

UK, Uchida–Kraepelin test; WM, working memory test; AUC, area under the receiver operating characteristic curve; CI, confidence interval.

25-OHD, 25-hydroxyvitamin D; ADMA, asymmetric dimethylarginine; Glu, glutamic acid; His, histidine; Ile, isoleucine; Leu, leucine; Ser, serine; Thr, threonine; Trp, tryptophan; Tyr, tyrosine; Val, valine.

### Biological pathway analysis

**Fig. 5:**
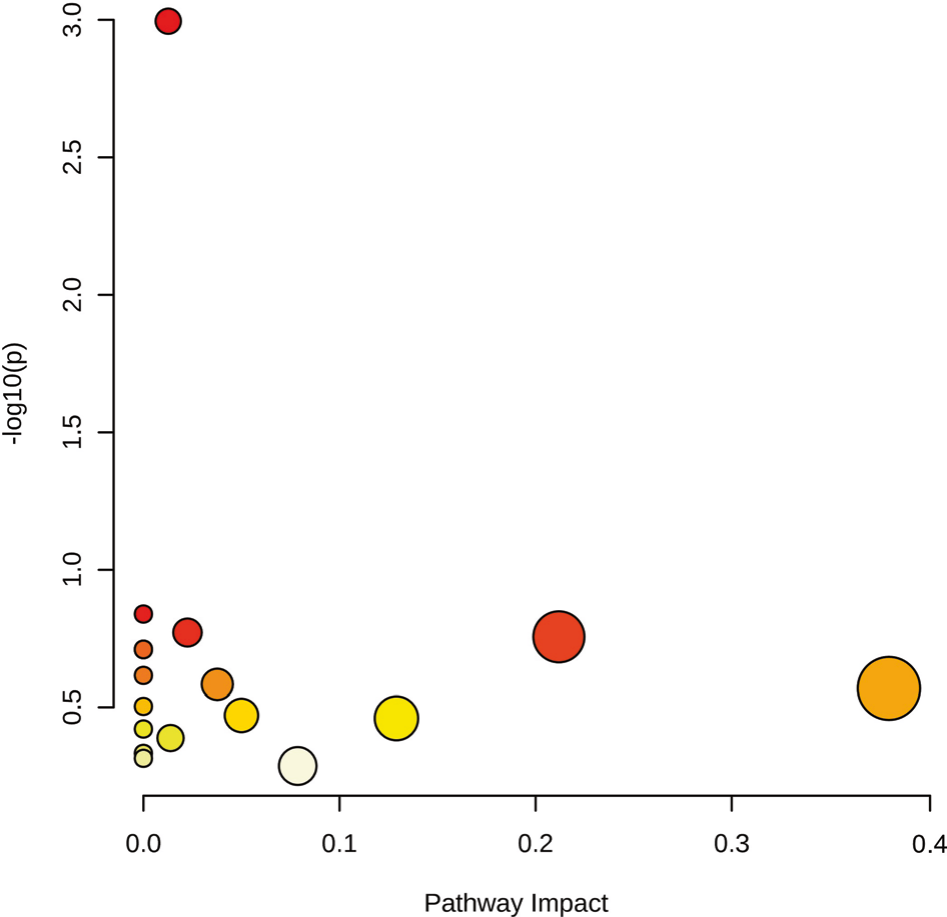
Metabolic pathway analysis based on discriminatory metabolites. The bubble plot displays the results of the pathway analysis conducted using MetaboAnalyst 6.0. The x-axis represents the pathway impact calculated from topology analysis, and the y-axis represents the statistical significance (-log_10_(*p*)). Each node represents a metabolic pathway; the node size corresponds to the pathway impact score, and the color gradient (from yellow to red) indicates increasing statistical significance. The most significantly enriched pathway was “Steroidogenesis” (*p* = 0.001), followed by "Beta-Alanine Metabolism" which showed a high pathway impact (0.38). The pathway data was obtained from the The Small Molecule Pathway Database (SMPDB) database.

To explore the biological implications of the metabolic shifts associated with chronic stress, a pathway analysis was performed using MetaboAnalyst 6.0. The analysis was conducted using the specific set of metabolites that exhibited nominal statistical significance (*p* < 0.05) at any time point during the study. The analysis identified several enriched metabolic pathways (Fig. 5). Notably, " Steroidogenesis" was most significantly enriched (*p* = 0.001), reflecting the endocrine response to stress. Although not reaching statistical significance after multiple testing correction, "Beta-Alanine Metabolism" exhibited a high pathway impact of 0.38; this pathway directly involves *N*-acetyl-*β*-alanine, one of our key marker candidates. Additionally, the "Arginine and proline metabolism" pathway, to which ADMA belongs, was also identified (driven by metabolites such as hydroxyproline). These results suggest that the *N*-acetyl-*β*-alanine/ADMA ratio reflects a composite of alterations in steroidogenesis and amino acid metabolic pathways that are central to the physiological stress response.

## Discussion

In this study, 128 samples of mouth-rinsed water were collected from 32 workers (16 high-stress, 16 control) at four time points. Group classification was based on the Brief Job Stress Questionnaire and the updated STAI, both well-established mental health assessment tools. Individuals scoring high on both were assigned to the high-stress group. Both groups completed the POMS 2, which assesses seven mood states^10^. The total mood disturbance score—calculated from six scales excluding vigor and vitality—and its normalized T score were significantly higher in the high-stress group, indicating more negative mood states (Table 2). Significant differences (q < 0.05) were observed in confusion-bewilderment, friendliness, and tension-anxiety. These findings confirm that classification using the Brief Occupational Stress Inventory and updated STAI accurately reflected differences in stress levels.

Statistically significant differences (*p* < 0.05) were observed between the high-stress and control groups in psychophysiological indices, including cerebral blood flow, PSQI scores, autonomic nervous activity, and body temperature (Table 2). The high-stress group showed a significantly lower initial activation—reflecting the early brain response during cognitive tasks or mental stress—and a delayed gravity range, indicating an imbalance in left–right cerebral blood flow timing during functions. Similar cerebral blood flow patterns, such as reduced initial activation and a delayed center of gravity, have been reported in patients with depression^11^; however, this resemblance should be interpreted cautiously. The PSQI, a self-administered sleep quality questionnaire, uses a cutoff score of 5.5 or higher to indicate sleep disorders^12^. Although the high-stress group’s average PSQI score was 4.88—below this threshold—it was significantly higher than the control group’s average of 3.19. These results indicate reduced sleep quality in the high-stress group, though this study could not clarify the causal relationship between sleep disturbances and high stress, warranting further investigation. Autonomic nervous activity analysis showed that the standard deviation of all normal-to-normal interval—an indicator of heart rate variability and autonomic regulation^13^—was significantly lower in the high-stress group after a 5-min rest (Recover) following the Uchida–Kraepelin test. This lower SDNN value suggests diminished autonomic flexibility and regulatory capacity in response to acute stress in the high-stress group. Regarding body temperature, the median value was significantly lower in the high-stress group (36.3 °C) than in the controls (36.8 °C) (*p* = 0.029). Prior studies have linked chronic stress to hypothermia^14^, suggesting that hypothermia may represent a stress-related phenotype in this group. Overall, these findings support the notion that classification by the Brief Job Stress Questionnaire and the updated STAI accurately reflects differences in stress levels between the high- and low-stress groups.

At the initial resting state (Rest), reflecting baseline physiological conditions before stress tasks, the high-stress group exhibited elevated 5-aminoimidazole-4-carboxamide ribonucleotide (AICAR) levels compared to those in the controls (*p* = 0.049), indicating stress-related activation of the AMPK pathway, as AICAR activates AMPK in cells^15^. Immediately following the Uchida–Kraepelin test (Post-UK), the high-stress group showed lower 25-hydroxyvitamin D (25-OHD) levels (*p* = 0.004) and higher tetrahydrocortisone and 11-deoxycortisol levels (*p* = 0.018) than the controls, suggesting increased synthesis and metabolism of vitamin D and cortisol, both of which are linked to stress responses^16,17^. At recovery, the high-stress group showed lower estrone levels (*p* = 0.006) and higher AICAR levels (*p* = 0.018), indicating suppressed sex hormone metabolism^18^ and activation of the AMPK pathway^15^. Immediately after the working memory test (Post-WM), the levels of *N*^6^, *N*^6^, *N*^6^-trimethyllysine (*p* = 0.009), and choline (*p* = 0.015) were reduced in the high-stress group, potentially reflecting effects on the synthesis of neurotransmitters, such as carnitine and acetylcholine^19,20^. Although these changes suggest dynamic metabolic responses to stress, none reached significance after multiple testing correction (*q* values), necessitating further validation to clarify specific mechanisms. Importantly, these individual metabolic shifts observed at different time points are collectively represented in the pathway analysis results, where steroid hormone and amino acid metabolism pathways emerged as the primary biological clusters. This holistic view reinforces that the discriminatory metabolites are part of a larger systemic response to psychophysiological stress.

No metabolites reached significance after multiple testing correction (*q* value) in two-group comparisons at individual time points; however, analysis of time-course changes across the four collection points identified numerous metabolites with significant differences (*q* < 0.05) (Tables S5, S6, S7, and S8). Among all participants, 68 metabolites showed significant temporal changes (Table S5), demonstrating the broad impact of acute stress on metabolism. The greater number of changing metabolites in the high-stress group than in the control group (43 vs. 15) suggests that chronic stress amplifies acute stress responses. Among these, seven metabolites exhibited significant changes of 30% or more from Post-UK to Recover across all participants, with additional modifications observed at other intervals (Table S8). These findings imply activation of metabolic shifts induced by short-term stress and subsequent homeostatic recovery processes. In the control group, few metabolites showed significant changes between time points overall. The three metabolites with the most frequent changes increased by 30% or more from Rest to Post-UK, including sIgA, a known stress marker. Salivary sIgA levels are known to transiently rise in response to acute stressors, such as tests and exercise^21^, indicating that this reflects a typical stress response to the calculation task. In contrast, the high-stress group exhibited the most significant number of metabolites with changes (15), all of which decreased between Post-UK and Post-WM. Notably, *γ*-aminobutyric acid (GABA), a key inhibitory neurotransmitter involved in regulating excessive neuronal excitation during stress^22^, was among these metabolites. The reduction of GABA in the high-stress group suggests a weakened inhibitory function, potentially amplifying the stress response. Since these changes were absent in the control group, they may reflect metabolic alterations specific to individuals with high stress.

We searched for biomarkers in mouth-rinsed water that could predict high-stress status, which were validated by both subjective assessments and objective psychophysiological measures. Initial attempts at model construction using logistic regression showed low feature selection stability and poor reproducibility in double cross-validation and bootstrapping. Stepwise variable selection revealed that single-variable models performed best, with accuracy declining when multiple features were added. Concluding that linear models were insufficient, we applied nonlinear feature transformations by constructing threshold-based models using ratios of metabolite pairs. This approach identified metabolite pairs with high predictive accuracy (Table 4).

In particular, the* N*-acetyl-*β*-alanine/ADMA ratio at rest demonstrated high predictive accuracy, indicating a metabolic response to chronic stress. ADMA is known to impact cardiovascular health by inhibiting nitric oxide synthase^23^. Furthermore, serum ADMA levels have been reported to be significantly elevated in individuals with schizophrenia, schizoaffective disorder, bipolar disorder, and depression compared to healthy controls^24^. Additionally, levels of *N*-acetyl-*β*-alanine, a blood metabolite linked to major depressive disorder (MDD), have been shown to correlate with MDD risk^25^. In the present study, a lower *N*-acetyl-*β*-alanine/ADMA ratio was associated with higher stress levels. Although the observed trend in ADMA is consistent with previous findings, the decrease in *N*-acetyl-*β*-alanine contrasts with recent reports of elevated levels in patients with MDD. This discrepancy may be explained by the clinical status of the current participants, who did not meet diagnostic criteria for MDD, suggesting a stage-specific metabolic response—namely, an early suppression of the β-alanine pathway under acute stress, followed by a compensatory increase during chronic depressive states.

Because no individual metabolite remained statistically significant after correction for multiple testing, the pathway analysis presented in Fig. 5 should be interpreted as exploratory and hypothesis-generating rather than confirmatory. The purpose of this analysis was not to claim definitive pathway-level dysregulation, but to assess whether metabolites showing nominal significance (p < 0.05) clustered within biologically plausible metabolic pathways related to stress responses. Among the identified pathways, “Steroidogenesis” showed the highest statistical significance (*p* = 0.001), which is consistent with the activation of the hypothalamic–pituitary–adrenal (HPA) axis—a hallmark of the physiological stress response. Notably, while the "Arginine and proline metabolism" pathway was enriched in our analysis, ADMA is a key component of this metabolic system. On the other hand, *N*-acetyl-*β*-alanine is intrinsically linked to *β*-alanine metabolism, which exhibited a high pathway impact (0.38) in our results. The clustering of these markers into stress-relevant metabolic pathways suggests that the *N*-acetyl-*β*-alanine/ADMA ratio does not merely represent isolated changes but reflects a coordinated biological response integrating endocrine signals and amino acid metabolism under chronic stress.

At other collection points, the progesterone/25-OHD ratio at Post-UK and the 4-methyl-2-oxopentanoate/*N*^6^,*N*^6^,*N*^6^-trimethyllysine ratio at Post-WM also showed high AUC values. Progesterone contributes to endocrine function, and 25-OHD regulates immune responses, implying that these ratios may reflect stress-related hormonal balance^26,27^. 4-Methyl-2-oxopentanoate is a leucine metabolic intermediate that produces acetyl-CoA in mitochondria, participating in energy metabolism and regulating autophagy^28^. N6,N6,N6-trimethyllysine is derived from histone lysine methylation and serves as a precursor for carnitine biosynthesis^19,29^. Changes in these metabolites may represent metabolic adaptations to acute stress. At the Recover stage, the serine/histidine ratio exhibited the highest AUC. Serine is crucial for cell cycle regulation and neurotransmitter synthesis^30^, while histidine functions in the stress response as a precursor for histamine^31^, suggesting that this ratio reflects metabolic adjustments during the recovery from acute stress.

The 4-methyl-2-oxopentanoate/choline ratio showed the highest AUC when combining data from all time points. Choline, a precursor of acetylcholine, is vital for nervous system function^20^, while 4-methyl-2-oxopentanoate regulates the mTORC1 pathway and autophagy^28^, potentially influencing stress response and neuronal activity. This ratio may serve as a comprehensive marker reflecting both the time course and cumulative stress load. In summary, single-metabolite models, much like the established single biomarkers (Table S9), lacked sufficient accuracy, likely due to high inter-individual variability. In contrast, models based on two-metabolite ratios successfully captured dynamic stress responses at each collection point, suggesting that a ratiometric approach can normalize for individual differences and reveal more robust stress signatures. Notably, the *N*-acetyl-*β*-alanine/ADMA ratio at rest demonstrated a strong predictive performance with an AUC of 0.845 (95% CI: 0.669 – 0.971, *p* = 0.042). Although an AUC > 0.9 is often considered ideal for standalone clinical diagnosis, an AUC of 0.845 is comparable to or exceeds the performance of established stress biomarkers. In fact, in this study, the single biomarkers such as salivary cortisol and sIgA showed AUCs ranging from 0.54 to 0.63 (Table S9). This relatively low performance is consistent with the known high inter-individual variability and influence of confounding factors associated with these markers^8^. Therefore, our model exhibits sufficient accuracy to serve as a non-invasive screening tool for office workers. However, the findings also suggest that multiple metabolites contribute to stress responses under varying conditions. Considering individual variability in stress reactions, further validation with larger sample sizes is necessary.

Regarding the origin of the detected metabolites, their presence in mouth-rinsed water is likely attributable to transport from the systemic circulation. Small molecular weight metabolites are thought to migrate into the oral cavity primarily via filtration through the gingival crevicular fluid (GCF)^32^ or through passive diffusion and active transport from salivary gland cells. Consequently, the metabolic profiles observed in mouth-rinsed water may reflect systemic physiological states mediated by these transport mechanisms.

A limitation of this study is the small sample size, as the analysis relied on 128 samples from 32 participants, which restricts the generalizability of the findings. Although objective physiological measures were incorporated to support group classification, the definition of high-stress status ultimately relied on validated questionnaire-based assessments, which may limit reproducibility across different populations and study settings. Furthermore, while the *N*-acetyl-*β*-alanine/ADMA ratio showed promise for stress evaluation, its validation is currently limited to the specific, short-term stress tests employed here. A deeper validation is needed, which should include not only different types of stress tests and conditions, but also a direct correlational analysis between the identified biomarkers and the objective physiological indices (e.g., fNIRS, SDNN) to elucidate the underlying mechanisms. Future studies should include larger, more diverse populations to account for individual variability. Additionally, exploring the roles of other physiological markers and metabolites involved in stress responses will be valuable. Addressing these points will be essential for advancing the practical application of stress screening using mouth-rinsed water.

In conclusion, this study identified candidate biomarkers in mouth-rinsed water that differentiated individuals experiencing high stress from those who are healthy. Although further research is needed to clarify the biological significance of these findings, they highlight the potential for a more straightforward and more accurate method to detect high stress in office workers. Future large-scale clinical studies are needed to validate the effectiveness of these biomarkers. This research provides a novel foundation for assessing workplace stress, with potential benefits for productivity and quality of life.

## Methods

### Ethics statement

This study was approved by the ethics committee of LION (Tokyo, Japan, approval No. 342). All participants understood the purpose of the study and provided written informed consent. All experiments were conducted in accordance with the approved guidelines and the principles outlined in the Declaration of Helsinki.

### Participant recruitment

From approximately 40,000 participants, 456 individuals were selected based on the inclusion and exclusion criteria detailed below. The inclusion criteria were as follows: (a) college graduate or higher, (b) regular office worker, (c) full-time day shift worker, and (d) no current or past physical illness under treatment. The exclusion criteria were as follows: (a) diagnosed mental disorders, (b) intellectual disabilities, (c) exclusive telecommuting workers, (d) denture users (including implants), (e) smokers, (f) antibiotic use within the past month, (g) ongoing orthodontic treatment, (h) diagnosed severe cardiovascular, liver, renal, digestive, respiratory, endocrine, or metabolic diseases, or alcoholism, (i) continuous medication use (excluding OTC topical medications), (j) severe periodontal disease, (k) untreated dental caries, (l) tooth extraction within the past week, (m) stomatitis with bleeding, (n) pregnancy, intention to become pregnant by study day, or ongoing breastfeeding, and (o) individuals judged unsuitable by the principal investigator (e.g., those who had difficulty adhering to the study protocol, such as maintaining the required schedule or condition). No participants were excluded from the analysis.

For the second screening, 500 selected participants completed the new version of the STAI (40 items)^33^ and the Brief Job Stress Questionnaire (57 items)^34^. Sixteen individuals meeting the high-stress criteria on both assessments were assigned to the high-stress group, while 16 individuals who did not meet the high-stress criteria on either assessment were assigned to the control group. High stress was defined as scoring at or above the 50th percentile on both the state and trait anxiety scales of the STAI^33^ and meeting the high-stress criteria of the Brief Job Stress Questionnaire^34^, either by the total score method (B total ≥ 77 or A + C total ≥ 76 and B total ≥ 63) or the raw score conversion method (B total ≤ 12, or A + C total ≤ 26 and B total ≤ 17). To prevent sex bias, each group was evenly divided between men and women, with ages balanced across the 20 s to 40 s range.

### Sample collection and psychophysiological examination

Before sampling, the participants were instructed to brush their teeth at least 1 h after eating or drinking and were not allowed to eat or drink for at least 1 h before sample collection. Participants first wore a wearable heart rate sensor (Union Tool Co., Ltd., Tokyo, Japan; myBeat WHS-1) to begin heart rate monitoring. They then completed a physical condition questionnaire, including the PSQI-J^12,35,36^ and the abbreviated POMS 2 psychological test^37^, followed by a 20-min rest before the first mouth-rinsed water sample collection. Subsequently, blood pressure was measured using an upper arm monitor (Omron Corp., Kyoto, Japan, HEM-7130-HP; medical device certification: 225AABZX00105000), and axillary temperature was recorded using a hospital-grade thermometer (Terumo, Tokyo, Japan, C207S; 30 s). A sensor band for fNIRS (Spectratech Corp., Yokohama, Kanagawa, Japan, Spectratech OEG-16ME; medical device certification: 227AFBZX00011000) was then applied to measure cerebral blood volume. For concentration stress loading, participants completed a 3-min Uchida–Kraepelin test (modified version, test paper by Japan Mental Technology Research Institute Co., Ltd., Tokyo, Japan), after which mouth-rinsed water was collected immediately.

After a 5-min rest, mouth-rinsed water was collected again. Participants then completed a 3-min working memory test (Spectratech, Yokohama, Kanagawa, Japan) as a memory stress task, followed by immediate collection of mouth-rinsed water. All samples were collected in 25 mL centrifuge tubes, mixed, and 200 μL aliquots were transferred into two 1.5 mL microtubes for Enzyme-Linked Immunosorbent Assay (ELISA) analysis. The remaining sample was moved to 5 mL microtubes for water-soluble metabolite and steroid analysis. All samples were immediately stored at –80 °C. Thereafter, samples in the 5 mL tubes were thawed, centrifuged again, aliquoted into 200 μL portions, and promptly stored at –80 °C.

### Metabolome analysis

Metabolome analysis was conducted using the method previously described in our study^6^, utilizing the same equipment, reagents, and conditions. Briefly, samples were thawed, centrifuged at 13,000 × *g* for 5 min at 4 °C, and filtered through a 5 kDa cutoff filter to remove large molecules. The filtrate was concentrated and dried using a centrifugal concentrator (CentriVap; Labconco, MO, USA) at 40 °C, followed by the addition of 60 μL of Milli-Q water containing the internal standards. Analysis was performed using capillary electrophoresis–time-of-flight mass spectrometry using Agilent Technologies systems^38–42^.

To ensure reliable quantification, the CE-MS method was validated, focusing on recovery rate and relative standard deviation (RSD) criteria. Only metabolites that met the requirements of 100 ± 30% recovery rate and within 20% RSD were included in the analysis. CE-MS analysis was performed in both cation and anion modes using dedicated systems. For each mode, measurements were executed in two separate batches. To monitor analytical stability, pooled quality control (QC) samples were prepared by mixing aliquots from 15 samples randomly selected from the total 128 samples. These QC samples were analyzed approximately every 15 samples throughout the analytical sequence. Samples were analyzed in a randomized order. Analytical consistency was verified by monitoring internal standards. For the CE-MS analysis, the median relative standard deviation (RSD) of the peak areas for the 100 metabolites included in the statistical analysis was 12.0%, demonstrating high analytical reproducibility. Data processing and absolute quantification were performed using MasterHands software^39,43^. Metabolites were identified by matching *m/z* values and migration times with those of authentic standard compounds. The identification confidence was considered ‘Level 1’ according to the Metabolomics Standards Initiative (MSI) guidelines^44^. Results are presented in Table S12.

### Steroid analysis

Following the previously reported method^45^, quantitative analysis was performed using LC–MS with stable isotope-labeled analogs as internal standards. When isotope-labeled analogs were not available, structurally similar stable isotope-labeled compounds were used as surrogate internal standards. The dispensed sample was concentrated and dried at 25 °C using centrifugation. Then, 30 μL of 0.1% formic acid water, 330 μL of 0.1% formic acid in methanol (MeOH), 10 μL of an internal standard mix (stable isotope-labeled analytes), and 30 μL of MeOH were added, mixed thoroughly, and centrifuged (15,300 × *g*, 10 min, 4 °C). The supernatant was transferred to a 1.5 mL Eppendorf tube. After adding 1 mL of 0.1% formic acid water and mixing, the solution was loaded onto a solid-phase extraction 96-well plate (Strata-X, 10 mg, 2 mL; Phenomenex) preconditioned with 1 mL of 0.1% formic acid/MeOH and 1 mL of 0.1% formic acid water. The plate was washed sequentially with 1 mL of 0.1% formic acid in water and 1 mL of 0.1% formic acid in 15% ethanol. Elution was performed with 200 μL of MeOH followed by 200 μL of isopropanol, and the eluate was dried using a nitrogen spray concentrator (SPE Dry 96, Biotage, Uppsala, Sweden). The residue was reconstituted in 40 μL of MeOH and mixed thoroughly for analysis.

The LC–MS system consisted of a Nexera LC system and a SHIMADZU LCMS8060 (Shimadzu Corporation, Kyoto, Japan). The analytical column was a Kinetex Biphenyl (2.6 μm, 2.1 mm × 100 mm, 00D-4622-AN; Phenomenex, Torrance, CA, USA) maintained at 30 °C. The mobile phase employed a gradient at a flow rate of 0.4 mL/min, with solution A as Milli-Q water containing 0.15 mM NH4F and solution B as MeOH containing 0.15 mM NH4F. The sample injection volume was 10 μL. The gradient program began at 50% mobile phase B, increasing linearly to 60% at 3 min, 90% at 8 min, and 95% at 10 min, then returning to 50% at 12.1 min, and held at 50% until 17 min to re-equilibrate to the starting conditions. Mass spectrometry was performed using ESI ionization in positive/negative switching mode (switching time: 5 ms) and multiple reaction monitoring (MRM) mode with 41 transitions (31 positive and 10 negative). Channel switching time was 1 ms, and MRM dwell times ranged from 31 to 199 ms per transition. The temperatures of the interface, heat block, and desolvation tube were set to 400, 500, and 150 °C, respectively. The flow rates for the heating, drying, and nebulizer gases were 10, 10, and 3 L/min, respectively. Raw data were processed using LabSolutions (ver. 5.2.2, Shimadzu Corporation, Kyoto, Japan). Identification was conducted by comparing retention times and *m/z* values with those of authentic standard compounds. The identification confidence was considered ‘Level 1’ according to MSI guidelines^44^. Concentrations were determined using peak area ratios of the analytes to surrogate internal standards with known added amounts, which co-elute with the analytes and correct for analytical variability, including differences in ionization efficiency and matrix effects. Measurements were executed in two separate batches in a randomized order. Within each batch, quality control was ensured by monitoring the peak intensities of stable isotope-labeled internal standards added to all samples. Samples exhibiting poor peak quality or significant deviations in internal standard response, indicative of technical errors or severe matrix interference, were re-analyzed to ensure data reliability.

### ELISA

The dispensed mouth-rinsed water samples were brought to room temperature (20–30 °C) and stirred. CgA was measured using a CgA ELISA kit (product number: YK070, Yanaihara Laboratory, Lot No. 2200824), and sIgA was measured using a sIgA ELISA kit (product number: YK280, Yanaihara Laboratory, Shizuoka, Japan, Lot No. 2200715), following the manufacturer’s instructions. Measurements were performed using a SpectraMax M5 microplate spectrophotometer (Molecular Devices, San Jose, CA, USA), and data were analyzed using SoftMax Pro version 5.0 (Molecular Devices, San Jose, CA, USA). For CgA, protein correction was conducted using a protein assay stain (BioRad, Lot No. 210006958) and human serum albumin (Sigma, Lot No. SLBM7779V) according to the kit’s manual.

### Data analysis

The variables analyzed included water-soluble metabolites, steroids, sIgA, and CgA. Sex, age were included as covariates. Water-soluble metabolites were selected based on analytical method validation by CE-MS, with recovery rates within 100 ± 30%, RSD within 20%, and a detection rate of at least 50% in either group. Consequently, 100 water-soluble metabolites met these criteria. These were combined with 25 steroids quantified by LC–MS and 2 salivary proteins (sIgA and CgA), resulting in a total of 127 biochemical features included in the statistical analysis. When metabolite concentrations were below the detection limit, a value equal to one-fifth of the minimum quantifiable value was substituted.

Principal component analysis (PCA) was performed using the scikit-learn library in Python to evaluate the natural grouping of samples. Prior to PCA, data were normalized using Z-score transformation. Hierarchical clustering analysis was also conducted using Euclidean distance and Ward’s method to visualize metabolic profiles.

For statistical comparisons, the significance level for biomarker testing was set at 5% (two-sided). Differences between groups were analyzed using the Mann–Whitney U test. Comparisons across multiple time points were made using the Friedman test, followed by the Nemenyi test for post-hoc analysis. Multiple testing correction was performed using the Benjamini–Hochberg method to obtain q-values.

To develop predictive models, logistic regression was employed. Feature selection was performed using forward–backward stepwise selection, L1 regularization, and the Boruta algorithm. For the threshold-based models, optimal cut-off values were determined using the Youden index. To evaluate the potential influence of sex on the identified stress markers, we confirmed the consistency of the results through a sex-stratified analysis. Furthermore, a logistic regression model adjusted for sex and age was employed to assess the robustness of the marker candidates.

Biological pathway analysis was also conducted using MetaboAnalyst 6.0^46,47^ with the list of metabolites showing significant differences (*p* < 0.05) to investigate the physiological relevance of the identified markers. The Small Molecule Pathway Database (SMPDB) library for Homo sapiens was utilized, and pathway impact was calculated using relative-betweenness centrality.

For the evaluation of predictive models, the statistical significance of the AUC was evaluated using DeLong’s method to estimate the variance of the AUC, followed by a one-sided Z-test under the null hypothesis that AUC = 0.5 and the alternative hypothesis that AUC > 0.5. Accuracy was defined as the proportion of correctly classified participants ((True Positives + True Negatives) / Total Participants). Accuracy was used as one of the evaluation metrics because the dataset had a balanced class distribution (16 participants per group). Accuracy and F1 score are reported as mean values across 100 bootstrap iterations, each involving LOOCV. The 95% CIs were estimated using the percentile method based on 100 bootstrap iterations. All statistical analyses were performed in Python 3.11.8^48^ using numpy 1.24.3^49^, pandas 2.1.4^50^, statsmodels 0.14.0^51^, and SciPy 1.7.3^52^.

## Supplementary Information

Supplementary Information.

## Data Availability

The datasets generated during and/or analyzed during the current study are available from the corresponding author on reasonable request.
