# Supplementary material for: Metabolomics of mouth-rinsed water for assessing psychophysiological stress in office workers
Source: Sci Rep. 2026 Apr 6;16:11735. doi: 10.1038/s41598-026-42241-4 (PMC13061908; doi:10.1038/s41598-026-42241-4)
Supplement: Supplementary file 1 — Supplementary Information. [file 41598_2026_42241_MOESM1_ESM.pdf]

Metabolomics of mouth-rinsed water for assessing psychophysiological stress in office workers

Yuki Maruyama[1, 2, 3*], Kaoru Yamada[1], Takuya Inokuchi[1], Narumi Fujii[1], Ryosuke Kawamata[1], Yuko Ichiba[1], Yasushi Kakizawa[1], Masahiro Sugimoto[2, 3], and Akiyoshi Hirayama[2, 3]

[1]Research and Technology Center, Lion Corporation. 7-2-1 Hirai, Edogawa-ku, Tokyo 132-0035, Japan

[2]Institute for Advanced Biosciences, Keio University, 246-2 Mizukami, Kakuganji, Tsuruoka, Yamagata 997-0052, Japan

[3]Systems Biology Program, Graduate School of Media and Governance, Keio University, Fujisawa, Kanagawa 252-0882, Japan

*Corresponding author:

Yuki Maruyama

Lion Corporation, 7-2-1 Hirai, Edogawa-ku, Tokyo, 132-0035, Japan

Email: yuki-u@lion.co.jp

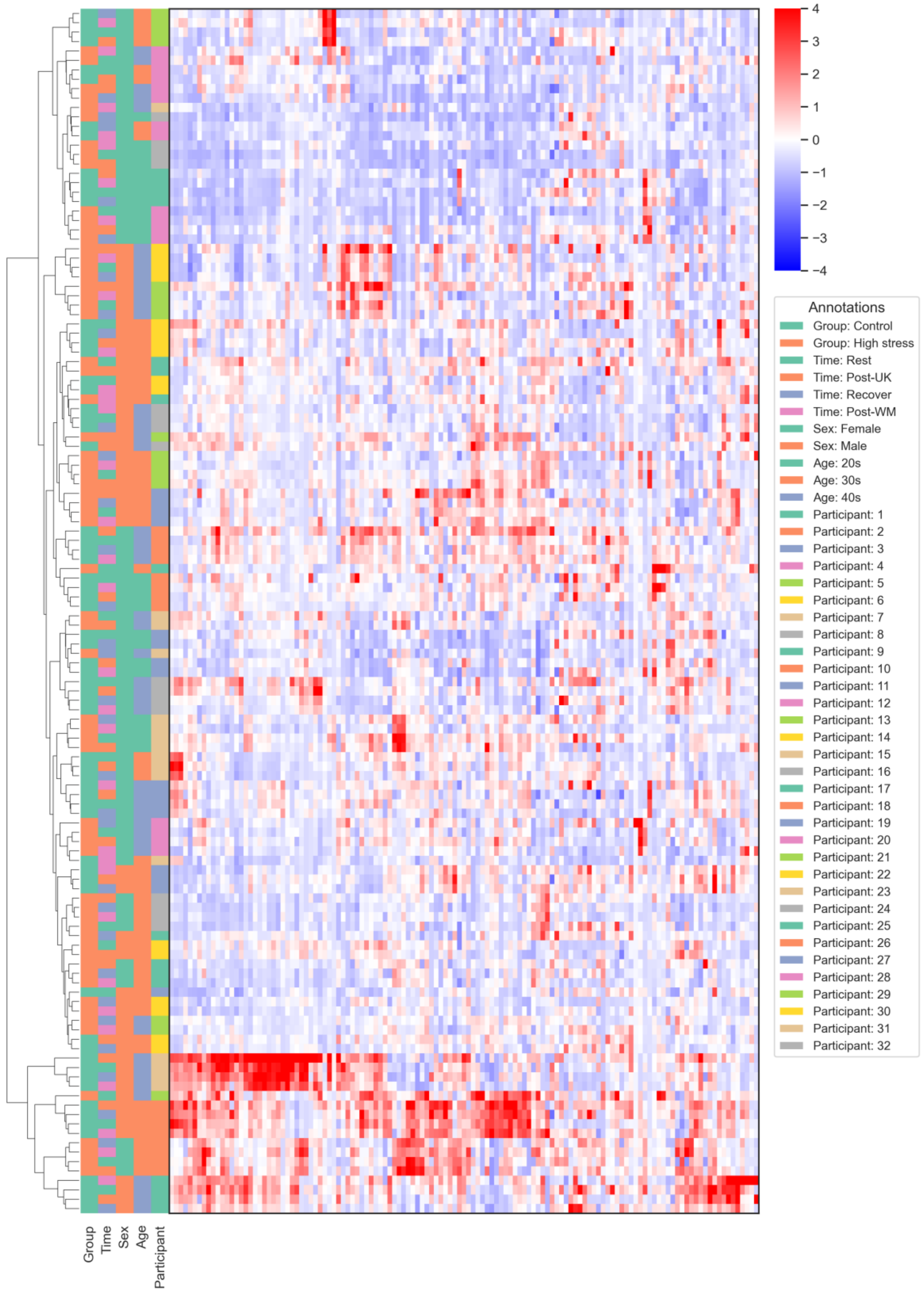

Figure S1. Hierarchical clustering heat map of mouth-rinsed water metabolites (Euclidean distance, Ward's method). Absolute metabolite concentrations were converted to z-scores for each metabolite and were colored based on the blue-red color scheme. "Group" indicates a control group (red) or a high-stress group (light blue). "Time" indicates the time of sample collection: initial resting state (red), post-UK (green), second resting state (light blue), and post-WM (purple). "Sex" indicates female (red) or male (light blue). "Age" indicates 20s (red), 30s (green), and 40s (blue). "Participant" indicates each of the 32 participants with a different color.

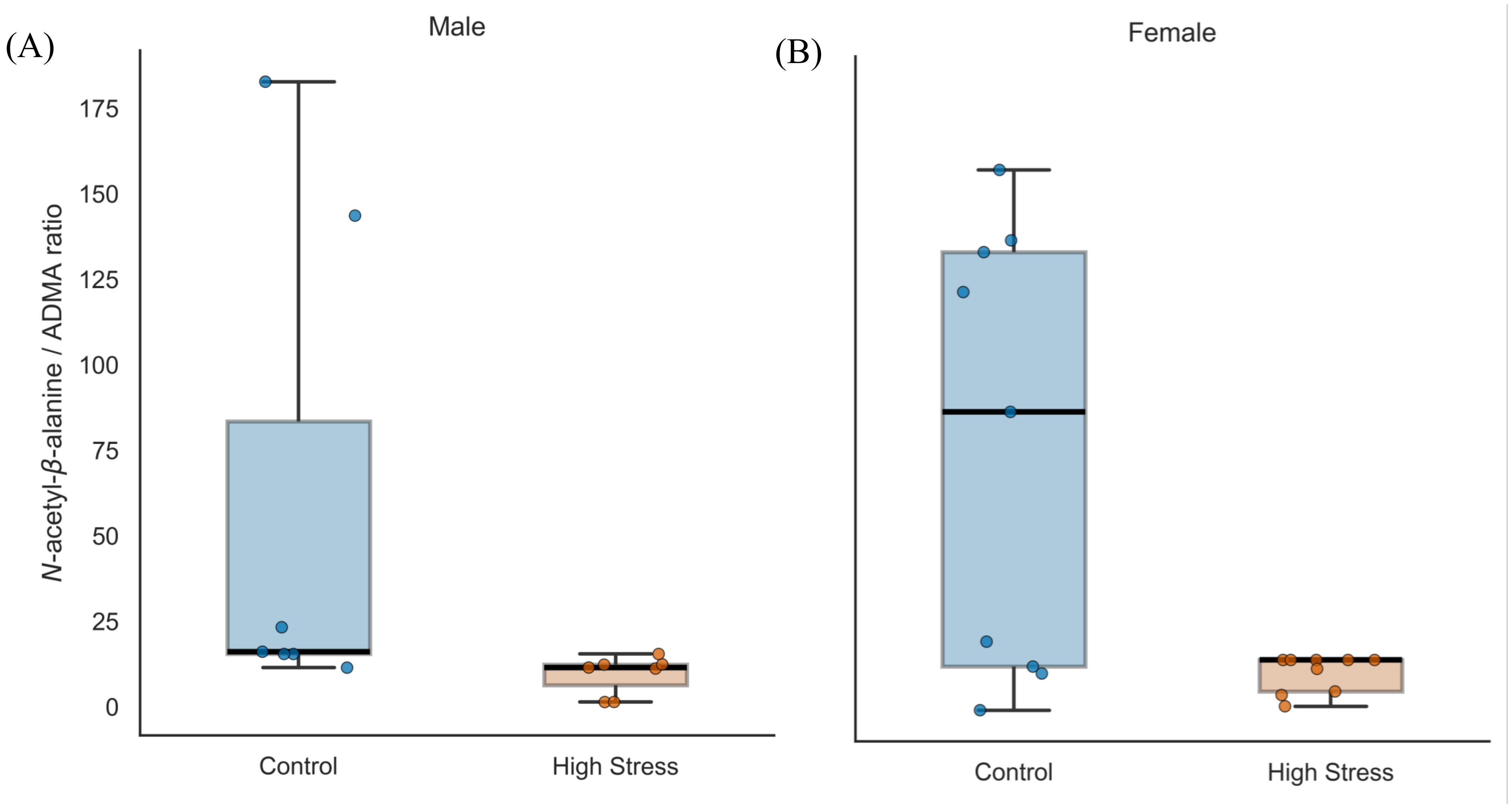

Figure S2. Gender-stratified analysis of the *N*-acetyl-*β*-alanine/ADMA ratio. Box plots show the median and interquartile range; whiskers indicate 1.5 × IQR. Each dot represents an individual sample. Data are shown separately for (A) male participants (Control, n = 7; High Stress, n = 7) and (B) female participants (Control, n = 9; High Stress, n = 9). Similar decreasing trends in the High-stress group were observed in both genders. ADMA, asymmetric dimethylarginine.

Table S1. Detailed demographic and clinical characteristics of study participants

Participant ID	Group	Gender	Age	STAI(State-Trait Anxiety Inventory)		BJSQ(Brief Job Stress Questionnaire)			
				State Anxiety (PA-P)	Trait Anxiety (PA-P)	Total Score for Domain B (Stress Response)	Total Score for Domains A+C (Stressor & Support)	Standardized Score for Domain B (Stress Response)	Standardized Score for Domains A+C (Stressor & Support)
1	Control	Female	27	7	5	41	49	25	45
3	Control	Female	48	1	5	38	53	26	44
6	Control	Male	34	0	0	30	42	29	55
8	Control	Female	42	1	0	34	42	27	56
10	Control	Male	30	13	24	35	49	26	51
11	Control	Male	31	5	5	32	40	29	54
13	Control	Female	39	1	2	35	44	28	48
14	Control	Male	36	4	1	31	38	30	54
15	Control	Male	45	0	1	37	56	25	47
17	Control	Male	45	0	0	30	33	29	60
18	Control	Female	26	1	6	53	54	21	42
20	Control	Female	34	0	0	31	39	29	52
23	Control	Female	39	4	0	33	50	28	46
24	Control	Male	46	0	3	45	54	22	44
26	Control	Female	46	2	7	38	53	28	41
27	Control	Female	28	7	7	41	55	26	42
2	High-stress	Female	33	81	98	94	72	8	32
4	High-stress	Female	25	81	95	90	82	9	27
5	High-stress	Male	44	83	95	104	85	7	21
7	High-stress	Female	29	81	87	77	58	12	39
9	High-stress	Female	34	98	99	101	91	6	21
12	High-stress	Female	47	83	71	98	68	8	35
16	High-stress	Female	31	98	100	100	69	9	34
19	High-stress	Male	39	87	87	87	56	11	41
21	High-stress	Male	47	87	81	88	61	11	37
22	High-stress	Male	37	76	68	78	67	12	34
25	High-stress	Male	35	97	97	85	79	8	27
28	High-stress	Female	41	81	99	75	70	11	33
29	High-stress	Male	32	76	63	72	81	14	27
30	High-stress	Male	43	87	78	85	66	12	34
31	High-stress	Female	40	98	98	102	95	10	19
32	High-stress	Female	28	83	51	77	71	13	31

Table S2. Complete comparison of psychophysiological indicators between high-stress and control groups

	Factor	Control Median	Control Q1	Control Q3	High-stress Median	High-stress Q1	High-stress Q3	*p* -value	*q* -value
1	POMS2_T	37.5	35	39.25	49	42	62	< 0.001	0.015
2	POMS2_TMD	-6	-10.5	-0.75	18	2.75	43	< 0.001	0.015
3	POMS2_CB	0.5	0	1	2.5	1	10	< 0.001	0.024
4	POMS2_F	14.5	11.75	15.25	8.5	7.75	11	0.001	0.024
5	POMS2_TA	2	1	3.25	6	3.75	9	0.001	0.024
6	POMS2_VA	12	10	15	5.5	4	9.25	0.001	0.033
7	POMS2_FI	2.5	1.75	3.25	7	3.75	13	0.001	0.033
8	POMS2_DD	0	0	1	3	0	7.75	0.002	0.065
9	POMS2_AH	1	0	2	3.5	1	7.25	0.006	0.137
10	Initial Activation_13CH_UK	0.0202	0.00334	0.03305	0.000802	-0.0097125	0.00425	0.010	0.207
11	PSQIG	3	2	4	5	3.75	6.25	0.014	0.263
12	Initial Activation_10CH_WM	0.004925	0.0027575	0.00655	-0.000666	-0.0025025	0.002945	0.018	0.325
13	BodyTemp(°C)	36.75	36.175	36.825	36.25	36.1	36.5	0.029	0.456
14	SDNN_Recover	52.5	41.025	63.575	38.15	27.95	45.6	0.030	0.456
15	Gravity_Range2_12CH_UK	129.5	123.25	142.5	151	133.75	161.25	0.038	0.536
16	Initial Activation_11CH_UK	0.0139	0.0057175	0.029125	0.0006735	-0.0065075	0.012325	0.057	0.705
17	Initial Activation_05CH_UK	0.01855	0.00405	0.0297	0.005415	-0.00606	0.015675	0.059	0.705
18	Initial Activation_08CH_UK	0.01505	0.000825	0.0233	0.0011	-0.0057825	0.007145	0.062	0.705
19	Initial Activation_AVERAGE_UK	0.0171	0.0024525	0.019775	0.003145	-0.0024625	0.0084975	0.068	0.705
20	Initial Activation_12CH_UK	0.01445	0.0026125	0.021725	-0.00244	-0.004935	0.00372	0.068	0.705
21	Gravity_Range2_08CH_WM	150	140.25	155.25	134	124.75	149.5	0.070	0.705
22	Initial Activation_14CH_UK	0.01235	0.00645	0.0251	0.0051	-0.0035525	0.010055	0.101	0.900
23	Gravity_Range1_13CH_UK	116.5	110.5	125	128	120.25	147.5	0.105	0.900
24	Gravity_Range1_10CH_WM	131	120.5	143	119.5	112	131	0.113	0.900
25	Initial Activation_14CH_WM	0.005475	-0.0006965	0.0085	-0.000842	-0.002755	0.00499	0.118	0.900
26	Initial Activation_10CH_UK	0.00319	-0.0041025	0.021125	-0.003465	-0.007885	0.00171025	0.118	0.900
27	Gravity_Range2_03CH_UK	133	118.75	148.5	145	134.25	170.25	0.127	0.900
28	Initial Activation_13CH_WM	0.00394	-0.00187	0.010725	0.0002315	-0.0053325	0.002485	0.127	0.900
29	Initial Activation_01CH_WM	0.006895	0.0027475	0.00941	0.002205	0.0005969	0.0066825	0.127	0.900
30	Integral_Range1_14CH_UK	41.25	28.925	56.025	25.25	13.74	41.6	0.137	0.900
31	LF/HF_Post-WM	1.195	0.924	1.7375	1.645	1.18225	3.0525	0.137	0.900
32	HF_Power_Post-UK	480	223	776.25	280.5	155.75	397	0.137	0.900
33	Gravity_Range1_02CH_UK	112.5	101.7	125.25	124	116.5	128.5	0.141	0.903
34	Initial Activation_15CH_UK	0.01046	0.00044975	0.02065	0.001415	-0.0058675	0.0111475	0.147	0.911
35	Gravity_Range2_14CH_UK	128.5	122.5	147.5	140	137.5	155.25	0.169	0.954
36	Gravity_Range1_01CH_UK	119.5	109	127.75	123	119.25	132.5	0.169	0.954
37	Integral_Range2_14CH_UK	48.8	37.975	67.15	31.1	15.39	50.55	0.169	0.954
38	LF_Power_Recover	538.5	352.25	1050	327	208.5	755	0.175	0.954
39	Initial Activation_07CH_UK	0.01665	-0.0039325	0.023975	-0.0010365	-0.008095	0.00652	0.181	0.954
40	HF_Power_Recover	424.5	140	812.5	179.5	142.25	332.5	0.181	0.954
41	Integral_Range2_04CH_UK	59.3	28.1	84.9	40.55	12.2175	63.475	0.194	0.974
42	Gravity_Range2_15CH_UK	133	123	145.5	141	134.25	158.5	0.200	0.974
43	HeartRate_Recover	44.6	38.875	50.9	49.6	43	53.425	0.221	0.974
44	Integral_Range2_13CH_UK	51.05	29.55	75.175	33.65	7.7875	54.5	0.221	0.974
45	Initial Activation_AVERAGE_WM	0.00244	0.0014025	0.0058375	0.000879	-0.0007785	0.0048775	0.221	0.974
46	Gravity_Range1_14CH_UK	118	111	135	128	123.75	141.5	0.227	0.974
47	Initial Activation_15CH_WM	0.0016	-0.0075775	0.00389	0.001735	-0.00106475	0.00838	0.235	0.974
48	LF_Power_Rest	627	308.75	1210	395.5	255.25	606.25	0.235	0.974
49	Gravity_Range2_AVERAGE_UK	126	119.5	139	132.5	127.25	143.75	0.242	0.974
50	Gravity_Range2_10CH_WM	144	133.75	154.25	133	121.75	148.5	0.242	0.974
51	SBP(mmHg)	118.5	100.75	125	107.5	99.5	115	0.250	0.974
52	Initial Activation_02CH_WM	0.007465	0.0018225	0.0108575	0.002735	0.0006995	0.00736	0.250	0.974
53	HF_Power_Rest	384	220.5	672.25	205.5	127.75	366.5	0.266	0.974
54	Gravity_Range2_05CH_WM	137.5	132	147.75	130.5	126.75	141.25	0.274	0.974
55	Gravity_Range1_15CH_UK	119.5	107.75	131.5	125	118	133	0.282	0.974
56	Integral_Range1_05CH_UK	36.2	13.1	78.85	23.15	2.24325	41.225	0.283	0.974
57	HeartRate_Post-WM	42.05	37.9	51.175	48.15	42.225	51.45	0.283	0.974
58	Initial Activation_02CH_UK	0.01595	0.0018385	0.026675	0.007665	0.0009325	0.01305	0.283	0.974
59	Integral_Range2_05CH_UK	43	13.725	93.325	28.15	4.06	48.6	0.283	0.974
60	Initial Activation_03CH_WM	0.00323	-3.47E-05	0.0093125	0.0009785	-0.0012425	0.0042075	0.283	0.974
61	Gravity_Range1_AVERAGE_UK	116	109.75	121	121.5	112.75	128.25	0.291	0.974
62	Gravity_Range1_08CH_WM	130	122.25	136.75	121.5	115	133	0.291	0.974
63	Integral_Range1_04CH_UK	48.05	24.1875	71.15	33.25	9.0325	54.825	0.300	0.974
64	Gravity_Range2_11CH_WM	141	133.75	148	137.5	126.75	141.25	0.308	0.974
65	Meals/day	3	3	3	3	3	3	0.309	0.974
66	HeartRate_Rest	47.9	44.05	51.35	50.3	44.575	52.35	0.318	0.974
67	Gravity_Range1_10CH_UK	121.5	115	136.25	132	120.5	139.5	0.327	0.974
68	Gravity_Range2_16CH_WM	137.5	130.75	143.5	131.5	125.75	145.25	0.336	0.974
69	LF/HF_Rest	2.66	1.5825	3.6575	2.37	1.4225	2.7625	0.336	0.974
70	HF_Power_Post-WM	345.5	98.625	695.5	183.5	135.75	342.25	0.336	0.974
71	LF_Power_Post-UK	629.5	190.5	1152.5	410.5	189.25	533.75	0.337	0.974
72	Integral_Range1_01CH_UK	30.6	22.8	42.25	42.15	28.775	56.9	0.337	0.974
73	Pulse(bpm)	72.5	66.75	81	67.5	64.25	74.25	0.345	0.974
74	Gravity_Range1_16CH_WM	121.5	115.75	128.25	119	111.5	127.25	0.346	0.974
75	Integral_Range2_03CH_UK	47.2	35.95	74.125	42.55	11.825	59.9	0.346	0.974

76	Initial Activation_09CH_UK	0.002595	-0.0016275	0.016675	0.0001235	-0.0056875	0.0084675	0.356	0.975
77	Integral_Range1_13CH_UK	38.45	16.2425	63.15	25.95	5.315	44.675	0.356	0.975
78	Initial Activation_06CH_UK	0.01076	0.000575	0.028525	0.007925	-0.00492	0.0198	0.376	0.987
79	Initial Activation_06CH_WM	0.003715	0.00030225	0.007755	0.001355	-0.001845	0.0061125	0.376	0.987
80	Integral_Range1_03CH_UK	40.65	27.9	62.6	38.3	6.4815	52.15	0.376	0.987
81	Gravity_Range2_11CH_UK	127.5	117	144	140	120.75	148.25	0.386	0.987
82	Gravity_Range1_07CH_UK	116.5	106.25	121	119	109	137.5	0.396	0.987
83	SDNN_Post-UK	51	34.825	69.975	43.5	35.05	57.375	0.429	0.987
84	Gravity_Range2_06CH_WM	130	124.75	133.25	131.5	127.75	144.25	0.439	0.987
85	Gravity_Range1_08CH_UK	120.5	108.75	125.25	123	113	135	0.440	0.987
86	SDNN_Rest	47	36.95	56.875	38.7	34.5	51.05	0.440	0.987
87	Integral_Range2_09CH_UK	43.45	11.125	65.05	47.3	27.05	81.5	0.440	0.987
88	Initial Activation_04CH_UK	0.006585	5.00E-06	0.027625	0.004275	-0.004895	0.02075	0.440	0.987
89	Integral_Range2_01CH_UK	38.25	26.8	52.075	45.9	32.275	64.2	0.440	0.987
90	Integral_Range2_04CH_WM	34.1	25.7	63.225	59.85	39.25	69.775	0.462	0.987
91	Gravity_Range1_06CH_WM	118	113.75	124.25	120.5	115	127.25	0.473	0.987
92	Gravity_Range1_11CH_UK	114.5	111.75	132	125	112.25	133	0.473	0.987
93	Gravity_Range2_09CH_WM	133.5	129.5	140	138.5	123.5	152.25	0.474	0.987
94	Integral_Range1_15CH_WM	32.65	26.625	55.925	44.1	32.825	54.85	0.474	0.987
95	Gravity_Range1_02CH_WM	125	115.5	130	123	112.75	125.5	0.485	0.987
96	Integral_Range1_09CH_UK	36.15	10.575	55.425	39.05	18.575	70.325	0.486	0.987
97	Integral_Range1_04CH_WM	31.4	22.15	55.05	47.85	31.7	60	0.486	0.987
98	Integral_Range2_15CH_WM	42.25	32.3	69.75	55.2	40.225	68.05	0.497	0.987
99	Gravity_Range1_05CH_WM	123.5	117.75	129.75	119.5	113.75	129.5	0.509	0.987
100	Gravity_Range2_03CH_WM	134	121.75	139.5	140	129.25	143.5	0.509	0.987
101	Integral_Range2_15CH_UK	62.5	29.95	83.675	46.6	21.425	88.75	0.509	0.987
102	Gravity_Range2_AVERAGE_WM	138.5	129.75	143	136	128.25	140.25	0.521	0.987
103	UK_Errors	0.5	0	2	1	0	2	0.525	0.987
104	Integral_Range1_14CH_WM	37.55	27.05	55.8	36.9	21.725	50.125	0.534	0.987
105	Integral_Range1_15CH_UK	47.25	26.075	70.675	36.55	14.725	69.925	0.534	0.987
106	Gravity_Range1_16CH_UK	113	100.625	126	117.5	111.25	127	0.546	0.987
107	Initial Activation_01CH_UK	0.00536	-0.0042475	0.022	0.00366	-0.0033375	0.00707	0.546	0.987
108	Gravity_Range1_14CH_WM	123.5	117.75	128.5	122.5	119	133.5	0.558	0.987
109	Gravity_Range2_08CH_UK	131	122.75	142	134	117.55	149.75	0.559	0.987
110	LF/HF_Post-UK	1.62	0.99225	2.2525	1.725	1.3575	2.5725	0.559	0.987
111	Integral_Range1_06CH_WM	30.2	20.425	47.225	39.55	25.35	45	0.559	0.987
112	Integral_Range2_14CH_WM	44.8	33.6	68.875	44.85	24.975	61.45	0.559	0.987
113	Initial Activation_03CH_UK	0.003255	-0.0007753	0.01765	0.0006935	-0.0025675	0.0125	0.559	0.987
114	Initial Activation_16CH_UK	0.005175	-0.0005733	0.016875	0.002675	-0.00050475	0.0099725	0.559	0.987
115	Initial Activation_12CH_WM	0.003595	0.00128193	0.0064	0.0007485	-0.004445	0.0116	0.559	0.987
116	Gravity_Range1_04CH_UK	113	102.75	126.75	118	108.5	129.5	0.571	0.987
117	UK_Workload	138.5	120.25	159	152.5	115.75	184.25	0.584	0.987
118	Initial Activation_16CH_WM	0.000716	-0.0035575	0.0030725	0.001267	-0.0019825	0.005075	0.585	0.987
119	Initial Activation_05CH_WM	0.00206	0.0015425	0.0042075	0.003035	0.00105275	0.0064575	0.585	0.987
120	Integral_Range2_AVERAGE_UK	43.95	25.8	66.45	40.4	28.5	52	0.585	0.987
121	Gravity_Range2_02CH_UK	128.5	120	143.5	136	124.75	139.5	0.597	0.987
122	Gravity_Range2_07CH_WM	135.5	128.5	153	136.5	125.75	148.25	0.598	0.987
123	DBP(mmHg)	78.5	72.75	87	76.5	71.75	83.5	0.611	0.987
124	BodySurfaceTemp_Rest	30.1	28.55	31.025	29.5	28.4	30.75	0.611	0.987
125	Gravity_Range2_13CH_UK	136.5	123.75	157.75	141.5	130	163	0.624	0.987
126	Integral_Range1_16CH_WM	42.45	32.225	55.15	53.5	29.475	63.65	0.624	0.987
127	Gravity_Range1_15CH_WM	126.5	118.75	131	124	118.25	131	0.637	0.987
128	Integral_Range2_11CH_UK	39.8	21.7375	58.325	32.1	21.2	49.025	0.638	0.987
129	HeartRate_Post-UK	48.55	41.325	51.125	49.05	43.925	51.7	0.651	0.987
130	Gravity_Range2_01CH_UK	138	130.5	146	140	132.25	149	0.664	0.987
131	Gravity_Range1_07CH_WM	122.5	118	135.25	122	117.25	129.75	0.664	0.987
132	Gravity_Range2_06CH_UK	125	118.25	133.5	130.5	118.5	140.75	0.664	0.987
133	Integral_Range1_10CH_WM	31.25	21.5	41.7	27.4	20.05	34.85	0.665	0.987
134	Integral_Range2_10CH_WM	37.55	24.1	51.65	34.25	25.025	41.85	0.665	0.987
135	Integral_Range2_06CH_WM	34.65	23.85	59.925	47.5	29.85	56.05	0.665	0.987
136	Gravity_Range1_AVERAGE_WM	123.5	118.25	129.5	122	116	125.25	0.678	0.987
137	Integral_Range2_01CH_WM	51.4	37.275	79.95	49.35	39.675	67.8	0.678	0.987
138	Integral_Range1_08CH_UK	17.5	13.375	43.65	22.65	4.3625	40.775	0.678	0.987
139	Integral_Range2_AVERAGE_WM	35.7	28.9	59.9	48.75	32.475	57.1	0.692	0.987
140	Integral_Range2_16CH_WM	51.35	39.5	67.475	62.1	36.875	77.025	0.692	0.987
141	Initial Activation_11CH_WM	0.002448	-0.001885	0.010475	0.002475	-0.00121825	0.0062675	0.692	0.987
142	BodySurfaceTemp_Post-UK	30.4	29.575	31.5	30.05	29.425	31.2	0.720	0.987
143	Integral_Range1_AVERAGE_UK	35.6	18.275	56.25	32.25	25.225	42.275	0.720	0.987
144	Integral_Range2_10CH_UK	43.35	19.575	68.5	40.55	13	58.675	0.720	0.987
145	Initial Activation_09CH_WM	0.001805	-0.0009468	0.0085	0.002237	-0.00060225	0.008925	0.720	0.987
146	Integral_Range1_13CH_WM	31.4	16.25	52.15	36.1	24.4	46.625	0.720	0.987
147	Integral_Range1_AVERAGE_WM	30	25.075	50.3	39.6	27.45	47.45	0.720	0.987
148	Integral_Range1_01CH_WM	42.35	31.65	65.85	40.9	34.625	53.425	0.720	0.987
149	Integral_Range1_10CH_UK	33.2	16.3	55.825	33.5	13.2725	49.325	0.734	0.987
150	Initial Activation_08CH_WM	0.0028	-0.003445	0.0055375	0.0005805	-0.001395	0.0045875	0.749	0.987
151	Integral_Range1_11CH_UK	32.25	19	49.525	28.35	18.975	43.4	0.763	0.987
152	Gravity_Range1_12CH_UK	122.5	111.5	129.75	123	111.75	141	0.763	0.987

153	Gravity_Range2_01CH_WM	135.5	129.75	141.25	137.5	126.5	144	0.777	0.987
154	Gravity_Range2_09CH_UK	130.5	124	144.25	138	117.5	149.5	0.777	0.987
155	Gravity_Range2_10CH_UK	138	127.75	158.25	136	127	153.25	0.777	0.987
156	Integral_Range1_07CH_UK	28.95	18.4	58.95	33.95	6.805	62.525	0.777	0.987
157	Integral_Range2_08CH_UK	24.7	15.0025	54.375	27.8	4.64	51.725	0.777	0.987
158	Gravity_Range1_11CH_WM	124.5	113	134	122	116.5	127.25	0.792	0.987
159	Gravity_Range2_07CH_UK	125	113.75	135	128.5	112.75	138.25	0.792	0.987
160	Integral_Range1_07CH_WM	29.05	12.325	45.425	28.35	15.225	48.375	0.792	0.987
161	Integral_Range2_06CH_UK	45.25	23.6	69.95	36.15	15.825	66.075	0.792	0.987
162	Integral_Range2_11CH_WM	35.95	24.025	57	36.05	22.425	52.45	0.806	0.987
163	Integral_Range2_13CH_WM	36.85	19.525	60.175	43.65	31.275	53.35	0.806	0.987
164	Integral_Range1_12CH_UK	34.25	13.025	60.3	31.85	12.4925	54.525	0.806	0.987
165	Integral_Range2_07CH_UK	34.25	20.3	71.075	40.6	10.685	72.7	0.806	0.987
166	LF_Power_Post-WM	325	174.75	887.75	287.5	228.5	491	0.806	0.987
167	Gravity_Range2_15CH_WM	142	137	145.25	142.5	131.75	146	0.821	0.987
168	Gravity_Range2_12CH_WM	138.5	132.25	144.5	135.5	131.25	147	0.821	0.987
169	Gravity_Range2_04CH_WM	130.5	122.75	134.75	129	124	139.5	0.821	0.987
170	Integral_Range1_11CH_WM	32.05	19.875	47.775	29.3	18.725	42.225	0.821	0.987
171	Gravity_Range1_09CH_WM	123	119	130	124	115.75	139.25	0.836	0.987
172	Gravity_Range1_01CH_WM	119	113.5	129	122	115.5	126	0.836	0.987
173	Gravity_Range2_16CH_UK	132	111.25	142.25	129.5	122.75	141.5	0.836	0.987
174	BodySurfaceTemp_Recover	30.55	29.575	31.5	30.2	29.55	31.25	0.836	0.987
175	LF/HF_Recover	2.68	1.1475	3.81	1.76	1.32	3.1525	0.836	0.987
176	Initial Activation_04CH_WM	0.004455	0.002545	0.009845	0.004465	0.00137	0.009785	0.836	0.987
177	Integral_Range2_07CH_WM	37.2	14.175	51.925	34.4	19.3	59.375	0.836	0.987
178	Integral_Range2_12CH_UK	42.1	16.525	71.65	42.55	16.45	65.975	0.836	0.987
179	BodySurfaceTemp_Post-WM	30.8	29.75	31.675	30.55	29.6	31.35	0.850	0.987
180	Integral_Range2_08CH_WM	31.05	18.65	56.375	29.3	21.075	44.1	0.851	0.987
181	Gravity_Range1_05CH_UK	112.5	104.75	121	113.5	98.35	133.25	0.865	0.987
182	Integral_Range2_05CH_WM	37.1	21.6	57.65	42.35	32.575	48.125	0.865	0.987
183	Integral_Range1_06CH_UK	37.25	17.05	58.075	34.1	12.35	54.225	0.865	0.987
184	Initial Activation_07CH_WM	0.00222	0.000882	0.003105	0.00216	0.0002515	0.0068575	0.865	0.987
185	Integral_Range1_05CH_WM	30.6	18.775	49.575	35.3	27.425	39.7	0.865	0.987
186	Gravity_Range1_09CH_UK	117	110.75	138.75	125	108	137.25	0.880	0.993
187	Integral_Range1_12CH_WM	25.45	16.75	50.25	32.2	13.9325	38.325	0.880	0.993
188	Gravity_Range1_03CH_WM	121	111.75	131.5	124	118.25	127.25	0.895	0.994
189	Integral_Range1_02CH_UK	28.4	16.5	63.9	37.8	18.75	52.7	0.895	0.994
190	Integral_Range2_12CH_WM	30.35	21.4	59.35	40.9	16.51	47.025	0.895	0.994
191	Gravity_Range2_13CH_WM	137	128.75	147	139.5	125.5	142.5	0.910	0.999
192	Gravity_Range2_04CH_UK	125.5	114.75	140	126.5	110.5	139.75	0.910	0.999
193	Integral_Range2_02CH_WM	38.25	29.625	69.45	43.8	35.9	56.125	0.925	0.999
194	Gravity_Range2_02CH_WM	138	132.25	140.25	136	123.5	142.5	0.940	0.999
195	Integral_Range2_03CH_WM	43.65	28.15	80.575	54	29.725	73.775	0.940	0.999
196	Gravity_Range1_13CH_WM	125.5	116.25	129.75	120	117.5	132.5	0.955	0.999
197	Integral_Range1_08CH_WM	24.9	14.8	48.3	24.85	18.175	37.95	0.955	0.999
198	Integral_Range1_02CH_WM	32.7	25.375	58.35	35.85	28.325	47.85	0.955	0.999
199	Integral_Range1_16CH_UK	31.45	15.725	53.4	29.9	10.2575	62.725	0.955	0.999
200	Gravity_Range2_14CH_WM	137	133.75	142.25	135.5	133	144.5	0.970	0.999
201	Gravity_Range1_03CH_UK	118.5	109.75	132	122	107.25	131	0.970	0.999
202	Integral_Range1_03CH_WM	36.8	23.025	69.925	43.5	23.875	61.625	0.970	0.999
203	Integral_Range2_09CH_WM	27.75	14.675	45.275	31.8	8.4125	53.575	0.970	0.999
204	Gravity_Range2_05CH_UK	124.5	117.75	138.25	128	117	141.5	0.985	0.999
205	Gravity_Range1_06CH_UK	115.5	108.5	129.75	120.5	109	132.5	0.985	0.999
206	SDNN_Post-WM	47.3	32.65	71.25	49.2	38.575	60.65	0.985	0.999
207	Integral_Range1_09CH_WM	22.75	11.475	38.95	26.3	6.5075	43.75	0.985	0.999
208	Integral_Range2_16CH_UK	38.95	18.025	65.15	40.65	11.9925	72.9	0.985	0.999
209	Gravity_Range1_12CH_WM	124.5	118.5	131	121	116.5	137.25	1.000	1.000
210	Gravity_Range1_04CH_WM	117.5	112.75	124	120	110	123	1.000	1.000
211	Integral_Range2_02CH_UK	37.95	17.8	75.625	41	23.775	58.375	1.000	1.000

Table S3. Metabolites with nominally significant differences between groups ($p < 0.05$)

Time point	Metabolite	Control group	High-stress group	p-value	q-value
Rest	AICAR	0.0528 [0.0528 – 0.106]	0.182 [0.0528 – 0.457]	0.049	1.000
Post-UK	25-OHD	6.34×10^{-5} [5.05×10^{-5} – 1.18×10^{-4}]	1.82×10^{-4} [1.13×10^{-4} – 2.64×10^{-4}]	0.004	0.563
	Tetrahydrocortisone	0.00187 [0.000317 – 0.00209]	3.10×10^{-4} [1.81×10^{-4} – 1.23×10^{-3}]	0.018	0.649
	11-Deoxycortisol	1.90×10^{-5} [7.93×10^{-6} – 3.25×10^{-5}]	6.63×10^{-6} [5.63×10^{-6} – 1.35×10^{-5}]	0.018	0.649
	Androstenediol	7.62×10^{-4} [4.07×10^{-4} – 8.81×10^{-4}]	2.91×10^{-4} [2.48×10^{-4} – 6.01×10^{-4}]	0.020	0.649
	Cholesterol	0.0398 [0.00755 – 0.0466]	0.00650 [0.00377 – 0.0242]	0.029	0.732
	Corticosterone	5.13×10^{-5} [4.58×10^{-5} – 6.54×10^{-5}]	3.65×10^{-5} [1.72×10^{-5} – 5.36×10^{-5}]	0.044	0.777
Recover	Estrone	3.76×10^{-6} [2.72×10^{-6} – 4.50×10^{-6}]	2.03×10^{-6} [1.55×10^{-6} – 2.50×10^{-6}]	0.006	0.713
	AICAR	0.00632 [0.00632 – 0.0126]	0.321 [0.00632 – 0.487]	0.018	0.713
	Hypoxanthine	1.92 [1.54 – 2.43]	1.31 [0.907 – 1.86]	0.027	0.713
	Hydroxyproline	0.195 [0.161 – 0.257]	0.143 [0.124 – 0.186]	0.032	0.713
	N-acetyl-*β*-alanine	0.144 [0.0224 – 0.180]	0.0224 [0.0224 – 0.137]	0.033	0.713
	2AB	0.296 [0.195 – 0.351]	0.189 [0.139 – 0.282]	0.044	0.713
	Adenine	0.269 [0.195 – 0.297]	0.188 [0.138 – 0.227]	0.046	0.713
Post-WM	N^6,N^6,N^6-trimethyllysine	0.0301 [0.0251 – 0.0394]	0.0181 [0.00189 – 0.0312]	0.009	0.513
	Choline	3.34 [2.35 – 3.91]	2.10 [1.67 – 2.63]	0.015	0.513
	Cystine	0.0484 [0.0259 – 0.0805]	0.00648 [0.00648 – 0.0355]	0.019	0.513
	DHEA	1.46×10^{-4} [8.55×10^{-5} – 2.87×10^{-4}]	7.71×10^{-5} [4.32×10^{-5} – 1.33×10^{-4}]	0.020	0.513
	Val	2.10 [1.63 – 3.94]	1.68 [1.06 – 1.90]	0.024	0.513
	Ile	0.554 [0.425 – 1.20]	0.430 [0.238 – 0.518]	0.030	0.513
	Glucosamine	0.0479 [0.0246 – 0.101]	0.00616 [0.00616 – 0.0428]	0.032	0.513
	Cysteine-glutathione disulfide	0.0658 [0.00586 – 0.107]	0.00586 [0.00586 – 0.0149]	0.032	0.513
	β- Ala	0.426 [0.279 – 0.697]	0.265 [0.239 – 0.430]	0.048	0.603
	Adenine	0.266 [0.200 – 0.312]	0.185 [0.157 – 0.236]	0.048	0.603

Data are presented as median [interquartile range (IQR)]. The p-values were determined by the Mann-Whitney U test. The q-values were calculated using the Benjamini-Hochberg procedure to control the false discovery rate.

UK, Uchida-Kraepelin test; WM, working memory test.

25-OHD, 25-hydroxyvitamin D; 2AB, 2-aminobutanoic acid; AICAR, 5-aminoimidazole-4-carboxamide ribonucleotide; DHEA, dehydroepiandrosterone; Ile, isoleucine; Val, valine.

Table S4. Full results of group comparisons for all metabolites by time point

[Time point: Rest]

Metabolite	Control Median	Control Q1	Control Q3	High-stress Median	High-stress Q1	High-stress Q3	*p* -value	*q* -value
1 AICAR	0.0528	0.0528	0.1056	0.1819	0.0528	0.4565	0.049	1.000
2 Ile	0.9135	0.37975	1.51	0.5005	0.278	0.668	0.059	1.000
3 *N* -acetyl-*β* -alanine	0.1985	0.16625	0.27325	0.0256	0.0256	0.23575	0.075	1.000
4 Glycerate	1.05	0.83625	1.18	0.658	0.44075	1.0975	0.113	1.000
5 Cysteine-glutathione disulfide	0.081	0.00624	0.12625	0.00624	0.00624	0.07365	0.115	1.000
6 Inosine	0.288	0.120815	0.38975	0.03678	0.01226	0.29925	0.117	1.000
7 Adenosine	0.06705	0.055425	0.080775	0.05415	0.0439	0.076425	0.118	1.000
8 Hydroxyproline	0.229	0.18325	0.2825	0.1835	0.1555	0.214	0.127	1.000
9 Agmatine	0.0357	0.027075	0.045875	0.043	0.034525	0.062825	0.141	1.000
10 *β* -Ala	0.403	0.3305	0.71775	0.3325	0.238	0.59775	0.158	1.000
11 Choline	3.335	2.83	4.175	2.985	2.0425	3.375	0.158	1.000
12 *N* ,*N* -dimethylglycine	0.1235	0.097175	0.16475	0.0996	0.081675	0.123	0.174	1.000
13 Val	2.68	1.29	4.8925	1.89	1.31	2.7775	0.175	1.000
14 Leu	2.07	1.245	4.5925	1.585	1.0325	2.335	0.207	1.000
15 Progesterone	0.0001225	7.16E-05	0.0001605	0.000167	9.74E-05	0.00018125	0.221	1.000
16 7-Methylguanine	0.02675	0.0132	0.0339	0.034	0.022875	0.041525	0.227	1.000
17 *N* -acetylglucosamine 6-phosphate	0.0924	0.01552	0.13125	0.01552	0.01552	0.099	0.235	1.000
18 Estriol	3.58E-05	1.81E-05	7.47E-05	2.27E-05	1.20E-05	2.86E-05	0.235	1.000
19 Glycolate	3.05	2.6075	3.39	3.535	2.4475	3.9825	0.266	1.000
20 Hypoxanthine	2.51	1.425	3.06	1.66	1.01275	2.6125	0.266	1.000
21 Methionine sulfoxide	0.0377	0.006	0.087625	0.006	0.006	0.070525	0.270	1.000
22 Glutathione(ox)	0.06135	0.0305	0.118	0.03905	0.00576	0.084425	0.271	1.000
23 Creatinine	1.155	0.95	1.4075	0.9725	0.81025	1.5875	0.283	1.000
24 4-Methyl-2-oxopentanoate	0.337	0.2335	0.48425	0.418	0.237	0.5125	0.291	1.000
25 25-OHD	0.0001035	5.56E-05	0.00031775	0.0001535	9.89E-05	0.00028575	0.300	1.000
26 DHEA-S	0.00432	0.0036925	0.006765	0.00372	0.0026975	0.0050075	0.300	1.000
27 Guanosine	0.1625	0.098925	0.24775	0.1029	0.060975	0.20175	0.300	1.000
28 Anserine	0.02445	0.00944	0.073425	0.00236	0.00236	0.06945	0.301	1.000
29 N^6,N^6,N^6-trimethyllysine	0.02855	0.02145	0.036225	0.0197	0.00324	0.0338	0.301	1.000
30 ADMA	0.00242	0.00242	0.0182	0.0159	0.00242	0.02155	0.310	1.000
31 Lithocholic acid	0.000127	0.0001095	0.00015	0.000111	9.97E-05	0.0001385	0.317	1.000
32 PEP	0.2805	0.2155	0.3855	0.241	0.16275	0.3735	0.327	1.000
33 Ser	3.135	2.4175	4.4875	2.995	2.0575	3.695	0.346	1.000
34 2AB	0.281	0.1975	0.45225	0.225	0.14175	0.32575	0.356	1.000
35 Asp	3.01	2.17	3.6825	2.39	2.1425	3.06	0.366	1.000
36 His	2.38	1.9325	3.075	2.905	2.105	3.1575	0.386	1.000
37 Gln	4.5	3.415	6.8225	3.965	2.465	6.5475	0.386	1.000
38 Glu	9.14	6.92	12.225	7.395	6.7425	10.5	0.407	1.000
39 11-Deoxycortisol	1.44E-05	7.59E-06	2.43E-05	1.03E-05	5.33E-06	2.28E-05	0.418	1.000
40 Proline betaine	0.02215	0.0136	0.05445	0.016	0.0034	0.03585	0.427	1.000
41 Homovanillate	0.5595	0.34775	0.86475	0.635	0.54525	0.85125	0.428	1.000
42 GABA	0.4135	0.26875	0.427	0.3535	0.22275	0.4145	0.428	1.000
43 Trp	0.188	0.1115	0.334	0.166	0.13375	0.20925	0.429	1.000
44 Pregnenolone	0.0002555	0.000167	0.000329	0.0002335	0.000115	0.000349	0.440	1.000
45 Citrulline	1.83	0.941	2.1825	1.44	1.24125	1.7425	0.440	1.000
46 Thr	1.09	0.86575	1.885	1.12	0.71075	1.5025	0.451	1.000
47 *N* -*ε* -acetyllysine	0.0416	0.0066	0.058175	0.0513	0.03905	0.060775	0.460	1.000
48 3-Methylhistidine	0.06265	0.0461	0.123	0.05835	0.03855	0.07865	0.462	1.000
49 2-Hydroxy-4-methylpentanoate	0.2325	0.132	0.307	0.243	0.16425	0.44175	0.473	1.000
50 Carnitine	0.4785	0.37775	0.6895	0.4235	0.3315	0.5265	0.486	1.000
51 Tyr	3.765	2.43	6.32	4.45	3.1025	6.99	0.486	1.000
52 Estrone	2.96E-06	2.19E-06	4.66E-06	2.30E-06	1.71E-06	4.17E-06	0.486	1.000
53 Betaine	0.8295	0.47125	0.99725	0.835	0.74275	1.0675	0.509	1.000
54 Threonate	0.4465	0.0552	0.59375	0.1656	0.0552	0.5695	0.516	1.000
55 Aldosterone	1.62E-05	1.17E-05	4.31E-05	1.43E-05	8.64E-06	2.19E-05	0.522	1.000
56 3-Phenyllactate	0.2885	0.13425	0.38275	0.2385	0.18975	0.3495	0.534	1.000
57 4-Androstene-3,17-dione	4.45E-05	3.13E-05	5.86E-05	4.70E-05	2.85E-05	8.75E-05	0.534	1.000
58 Adenine	0.198	0.14675	0.29725	0.203	0.117725	0.24575	0.534	1.000
59 6-Phosphogluconate	0.131	0.05456	0.1555	0.1335	0.112	0.16625	0.546	1.000
60 Asn	0.127	0.00672	0.228	0.182	0.00672	0.4085	0.548	1.000
61 *N* -acetylputrescine	0.3165	0.253	0.58625	0.4775	0.30825	0.6615	0.559	1.000
62 Creatine	4.5	3.7125	6.885	4.08	3.29	5.7975	0.559	1.000
63 Cystine	0.02016	0.00672	0.0555	0.00672	0.00672	0.0496	0.575	1.000
64 Carnosine	0.0242	0.01288	0.03445	0.01765	0.00322	0.0382	0.579	1.000
65 Sarcosine	1.52	1.245	2.405	1.76	1.195	1.9725	0.585	1.000
66 Cholesterol	0.0153	0.003215	0.04745	0.009695	0.0033025	0.0341	0.585	1.000
67 Cortisol	0.00145	0.00083075	0.002185	0.00165	0.0010125	0.002315	0.598	1.000
68 SAM+	0.0208	0.00328	0.028075	0.0184	0.00328	0.026475	0.603	1.000
69 Gly-Leu	0.06625	0.040025	0.08745	0.0468	0.037375	0.0826	0.611	1.000
70 2-Hydroxypentanoate	0.4915	0.2885	0.613	0.505	0.36775	0.736	0.611	1.000
71 Isopropanolamine	0.1135	0.074825	0.1505	0.106	0.0843	0.205	0.624	1.000

72	Thymine	2.265	1.885	3.0575	2.32	1.37475	3.83	0.637	1.000
73	Cytidine	0.111	0.0288	0.13025	0.1045	0.048525	0.15625	0.649	1.000
74	4-Hydroxymandelate	0.8265	0.614	1.4325	1.0875	0.47255	1.9575	0.650	1.000
75	1,3-Diaminopropane	0.109	0.085625	0.246	0.1095	0.042375	0.2415	0.664	1.000
76	5-Oxoproline	1.87	1.5925	2.3875	1.99	1.28	2.4375	0.665	1.000
77	sIgA(μg/mL)	41	36.1	50.625	43.75	36.625	51.975	0.665	1.000
78	DHEA	9.59E-05	4.58E-05	0.000221	0.0001365	9.48E-05	0.00020575	0.665	1.000
79	Indole-3-acetate	0.856	0.661	1.05	0.928	0.0844	1.345	0.677	1.000
80	o-Acetylcarnitine	0.3575	0.2305	0.5235	0.413	0.38625	0.6195	0.678	1.000
81	DHT	8.75E-05	5.95E-05	0.00015725	0.00010125	3.82E-05	0.0001925	0.692	1.000
82	17-OHP	7.43E-05	4.65E-05	9.02E-05	9.20E-05	3.11E-05	0.00011675	0.692	1.000
83	Cortisone	0.005705	0.0041975	0.0081325	0.00625	0.00475	0.00883	0.692	1.000
84	γ-Butyrobetaine	0.4515	0.341	0.67	0.4335	0.231	0.72925	0.692	1.000
85	18-OH-Corticosterone	0.000136	8.68E-05	0.000476	0.000275	9.14E-05	0.00063475	0.692	1.000
86	Lys	3.505	2.2	6.56	3.98	3.005	6.7675	0.692	1.000
87	N^1,N^{12}-Diacetylspermine	0.01865	0.013105	0.0256	0.01735	0.00175	0.027525	0.704	1.000
88	Gly-Gly	0.0762	0.02624	0.123	0.0821	0.056725	0.11575	0.748	1.000
89	Corticosterone	5.64E-05	3.47E-05	8.19E-05	4.81E-05	2.74E-05	7.82E-05	0.749	1.000
90	Glycerophosphate	4.24	3.75	5.325	4.865	3.8925	5.855	0.749	1.000
91	3-Aminoisobutyrate	0.252	0.16025	0.3525	0.26	0.14275	0.3545	0.749	1.000
92	11-Deoxycorticosterone	0.00687	0.0049625	0.0084925	0.00653	0.005505	0.0071425	0.763	1.000
93	Trimethylamine N-oxide	0.0286	0.01176	0.13525	0.0365	0.024285	0.08225	0.776	1.000
94	Ala-Ala	0.10165	0.055095	0.15875	0.116	0.05112	0.143	0.776	1.000
95	Androstenediol	0.0005425	0.0002715	0.00080125	0.0005095	0.00029175	0.000823	0.777	1.000
96	CgA/protein(pmol/mg)	24.15	5.36	53.8	8.67	6.415	29.825	0.777	1.000
97	Ornithine	2.4	1.18	3.5675	2.685	1.5375	3.74	0.777	1.000
98	Glucosamine	0.06235	0.0064	0.13375	0.0532	0.0064	0.084275	0.818	1.000
99	3-(4-Hydroxyphenyl)propionate	1.68	1.08	2.2325	1.94	0.58025	2.435	0.821	1.000
100	1-Methyladenosine	0.0165	0.00204	0.028725	0.016	0.00966	0.022925	0.834	1.000
101	Hypotaurine	0.3065	0.187	0.41975	0.2895	0.1376	0.41425	0.835	1.000
102	Met	0.118	0.04745	0.2145	0.1345	0.0308	0.2595	0.849	1.000
103	Tetrahydrocortisone	0.000794	0.000207	0.001805	0.0005745	0.00020125	0.001785	0.851	1.000
104	N-acetylornithine	0.00322	0.00322	0.0374	0.00966	0.00322	0.03935	0.854	1.000
105	Nicotinate	0.251	0.17225	0.30625	0.217	0.025	0.4135	0.864	1.000
106	N^1,N^8-Diacetylspermidine	0.0184	0.012875	0.0235	0.0157	0.008625	0.0278	0.880	1.000
107	Histamine	0.0427	0.029625	0.1365	0.044	0.026325	0.12925	0.880	1.000
108	Tetrahydrocortisol	0.0026	0.00110725	0.00854	0.00346	0.00112	0.007715	0.895	1.000
109	N-acetylaspartate	0.3955	0.18775	0.64975	0.315	0.21125	0.7195	0.895	1.000
110	17-OH-Pregnenolone	0.0003445	0.00011525	0.00046875	0.0002795	0.0001037	0.00055875	0.895	1.000
111	N-acetylglutamate	0.06005	0.0372	0.084475	0.06625	0.0093	0.084625	0.924	1.000
112	Galacturonate 1-phosphate	0.08175	0.009	0.13025	0.07155	0.049275	0.1365	0.924	1.000
113	Biotin	0.0314	0.0314	0.233	0.0314	0.0314	0.222	0.934	1.000
114	Azelate	0.1325	0.11225	0.14625	0.124	0.102225	0.15425	0.940	1.000
115	Homoserine	0.07385	0.0095	0.09125	0.07025	0.0095	0.088825	0.954	1.000
116	Urocanate	0.2975	0.22975	0.4305	0.3025	0.20125	0.42075	0.955	1.000
117	α-Aminoadipate	0.4555	0.3425	0.57325	0.5035	0.325	0.60325	0.955	1.000
118	Glu-Glu	0.04375	0.0054	0.06425	0.04585	0.0216	0.055475	0.969	1.000
119	CMP	0.2395	0.1795	0.36175	0.28	0.14925	0.45075	0.985	1.000
120	Spermidine	0.257	0.18225	0.35175	0.2495	0.17875	0.39625	0.985	1.000
121	Arg	4.62	2.7025	6.0975	4.85	3.8975	5.4425	0.985	1.000
122	Pipecolate	0.06775	0.055775	0.1255	0.07725	0.05875	0.1135	0.985	1.000
123	Phe	2.98	2.1075	4.4425	2.755	2.2575	4.75	1.000	1.000
124	N-acetylglucosamine 1-phosphate	0.1035	0.05664	0.14625	0.1215	0.01416	0.15725	1.000	1.000
125	Testosterone	4.52E-05	2.09E-05	6.48E-05	5.57E-05	8.06E-06	0.00011375	1.000	1.000
126	Cytosine	0.0533	0.01656	0.070725	0.04815	0.035725	0.06295	1.000	1.000
127	17β-Estradiol	7.93E-06	4.36E-06	1.23E-05	7.90E-06	5.44E-06	1.02E-05	1.000	1.000

[Time point: Post-UK]

	Metabolite	Control Median	Control Q1	Control Q3	High-stress Median	High-stress Q1	High-stress Q3	p-value	q-value
1	25-OHD	6.34E-05	5.05E-05	0.0001175	0.000182	0.00011325	0.0002635	0.004	0.563
2	Tetrahydrocortisone	0.001865	0.00031725	0.0020925	0.00031	0.00018075	0.0012325	0.018	0.649
3	11-Deoxycortisol	1.90E-05	7.93E-06	3.25E-05	6.63E-06	5.63E-06	1.35E-05	0.018	0.649
4	Androstenediol	0.000762	0.00040675	0.0008805	0.000291	0.0002475	0.00060125	0.020	0.649
5	Cholesterol	0.03975	0.007545	0.046625	0.006495	0.00377	0.0242	0.029	0.732
6	Corticosterone	5.13E-05	4.58E-05	6.54E-05	3.65E-05	1.72E-05	5.36E-05	0.044	0.777
7	Tetrahydrocortisol	0.004815	0.0035375	0.00854	0.003725	0.00137575	0.00492	0.052	0.777
8	18-OH-Corticosterone	0.0003745	0.000154	0.00051825	0.0001565	5.81E-05	0.0003365	0.052	0.777
9	Choline	3.41	3.01	4.1575	2.915	2.2025	3.4075	0.057	0.777
10	Adenine	0.265	0.21525	0.3405	0.1915	0.157	0.30025	0.062	0.777
11	Nicotinate	0.2815	0.14615	0.3675	0.1205	0.0146	0.25125	0.068	0.777
12	17β-Estradiol	8.82E-06	6.12E-06	1.20E-05	4.96E-06	4.38E-06	7.78E-06	0.073	0.777
13	Estriol	4.23E-05	2.33E-05	6.91E-05	1.48E-05	7.62E-06	6.79E-05	0.080	0.778
14	11-Deoxycorticosterone	0.007545	0.00674	0.0082475	0.00661	0.0055375	0.007835	0.105	0.953
15	Ile	0.8665	0.36375	1.5175	0.439	0.348	0.77875	0.122	0.969

16	Adenosine	0.07925	0.06315	0.102325	0.059	0.05025	0.08515	0.132	0.969
17	DHEA-S	0.00516	0.00427	0.0074425	0.00352	0.0023675	0.0056225	0.147	0.969
18	Aldosterone	2.75E-05	1.48E-05	4.52E-05	1.72E-05	1.20E-05	2.97E-05	0.147	0.969
19	Estrone	2.94E-06	2.05E-06	5.14E-06	2.11E-06	1.25E-06	3.11E-06	0.158	0.969
20	DHEA	0.0001775	0.00013075	0.0002635	0.0001185	2.91E-05	0.00019	0.169	0.969
21	Met	0.191	0.1315	0.33925	0.154	0.062975	0.22575	0.180	0.969
22	Progesterone	0.000176	0.00013	0.0001875	0.0001146	3.13E-05	0.00018725	0.181	0.969
23	Ala-Ala	0.0967	0.01158	0.115	0.1495	0.06735	0.17575	0.189	0.969
24	Val	2.695	1.6325	4.6325	1.96	1.3625	2.83	0.200	0.969
25	Hydroxyproline	0.2545	0.18075	0.3315	0.209	0.17775	0.24725	0.221	0.969
26	Agmatine	0.03585	0.034125	0.049525	0.05105	0.035825	0.064125	0.227	0.969
27	Hypoxanthine	2.405	1.47	3.1475	1.7	1.1425	2.49	0.235	0.969
28	Testosterone	5.10E-05	1.80E-05	0.000215	3.34E-05	1.03E-05	6.13E-05	0.250	0.969
29	Gln	4.955	3.4775	6.5475	3.79	3.54	5.23	0.250	0.969
30	Creatine	5.015	4.19	6.7775	4.515	3.7975	5.4075	0.266	0.969
31	Leu	2.505	1.2425	4.4825	1.54	1.35	2.6175	0.291	0.969
32	2AB	0.358	0.2075	0.428	0.272	0.19925	0.36575	0.291	0.969
33	Homovanillate	0.5055	0.29305	0.96525	0.7635	0.6595	0.8905	0.298	0.969
34	β-Ala	0.48	0.395	0.759	0.3925	0.33775	0.66925	0.300	0.969
35	Inosine	0.329	0.2805	0.42025	0.2795	0.19225	0.42375	0.309	0.969
36	o-Acetylcarnitine	0.4115	0.28425	0.56725	0.483	0.348	0.652	0.309	0.969
37	Thr	1.22	0.86175	1.6525	1.0185	0.88125	1.2725	0.327	0.969
38	Cortisone	0.004205	0.0032925	0.0065125	0.00522	0.0037975	0.007455	0.336	0.969
39	N-acetylputrescine	0.402	0.35	0.57225	0.553	0.38975	0.66975	0.336	0.969
40	Ser	2.725	2.21	4.57	2.815	1.93	3.355	0.337	0.969
41	His	2.31	1.73	3.03	2.73	2.3175	3.4175	0.346	0.969
42	Asn	0.162	0.0089	0.24675	0.161	0.076925	0.334	0.353	0.969
43	3-Methylhistidine	0.06265	0.054275	0.124	0.0582	0.045375	0.092225	0.356	0.969
44	Glu-Glu	0.0452	0.02072	0.062725	0.02419	0.00518	0.05945	0.363	0.969
45	Glucosamine	0.07045	0.042225	0.12825	0.0571	0.00484	0.12	0.373	0.969
46	Glu	8.575	6.5125	10.8	8.015	6.0675	8.9975	0.376	0.969
47	Creatinine	1.215	0.9115	1.6225	0.9145	0.84525	1.4575	0.386	0.969
48	N^6,N^6,N^6-trimethyllysine	0.03415	0.022625	0.041425	0.0276	0.022875	0.03615	0.396	0.969
49	GABA	0.479	0.39075	0.555	0.455	0.3335	0.499	0.396	0.969
50	N-acetylglutamate	0.02902	0.00864	0.0718	0.0637	0.00864	0.082	0.402	0.969
51	Asp	2.955	2.1325	3.825	2.455	2.2075	3.04	0.407	0.969
52	Pregnenolone	0.000307	0.00020975	0.00036125	0.000227	0.0001465	0.00036575	0.407	0.969
53	Threonate	0.4655	0.357	1.02	0.465	0.0658	0.68925	0.415	0.969
54	17-OHP	6.08E-05	2.72E-05	9.14E-05	4.19E-05	2.31E-05	7.59E-05	0.429	0.969
55	AICAR	0.353	0.01474	0.40625	0.11285	0.01474	0.40875	0.439	0.969
56	Tyr	3.715	2.92	5.56	4.055	3.2925	6.325	0.440	0.969
57	1,3-Diaminopropane	0.129	0.08955	0.22725	0.1039	0.07955	0.19475	0.451	0.969
58	4-Androstene-3,17-dione	5.99E-05	3.45E-05	6.91E-05	6.75E-05	3.48E-05	7.86E-05	0.462	0.969
59	CMP	0.2665	0.1651	0.34375	0.1785	0.08297	0.34825	0.472	0.969
60	Glycerate	0.75	0.62825	0.84025	0.8165	0.662	1.055	0.474	0.969
61	Cytosine	0.06405	0.04435	0.0909	0.05645	0.040825	0.070425	0.486	0.969
62	DHT	9.79E-05	7.02E-05	0.000139	7.86E-05	4.69E-05	0.0001595	0.486	0.969
63	Carnitine	0.4515	0.4115	0.6235	0.4485	0.35125	0.54025	0.497	0.969
64	Galacturonate 1-phosphate	0.0893	0.01048	0.1205	0.105	0.04192	0.1235	0.501	0.969
65	Trp	0.204	0.1585	0.24875	0.171	0.13575	0.2435	0.509	0.969
66	Lys	3.22	2.0475	3.8875	3.49	2.8275	5.27	0.510	0.969
67	N-acetylglucosamine 6-phosphate	0.01174	0.01174	0.11225	0.01174	0.01174	0.080725	0.511	0.969
68	Azelate	0.131	0.107	0.16075	0.142	0.11575	0.168	0.534	0.972
69	N-ε-acetyllysine	0.05395	0.042875	0.081625	0.0637	0.046925	0.086725	0.534	0.972
70	N-acetyl-β-alanine	0.1855	0.0254	0.23575	0.163	0.0254	0.2	0.545	0.972
71	Ornithine	1.825	1.1275	3.905	2.055	1.7125	3.825	0.559	0.972
72	Hypotaurine	0.3415	0.25525	0.5285	0.342	0.19675	0.437	0.571	0.972
73	6-Phosphogluconate	0.102	0.01274	0.134	0.1065	0.06505	0.1765	0.607	0.972
74	N,N-Dimethylglycine	0.1375	0.10195	0.157	0.112	0.09455	0.14025	0.611	0.972
75	Cytidine	0.1185	0.00998	0.167	0.113	0.0873	0.18325	0.649	0.972
76	Pipecolate	0.0912	0.072	0.20125	0.09795	0.064725	0.12625	0.665	0.972
77	Carnosine	0.0228	0.0021	0.032775	0.02315	0.0084	0.0462	0.688	0.972
78	sIgA(μg/mL)	54.95	43.7	76.025	64.35	51.6	73.1	0.706	0.972
79	Glycolate	2.905	2.585	4.215	3.065	2.5575	3.515	0.720	0.972
80	Glycerophosphate	5.11	4.58	6.685	5.185	4.285	6.25	0.720	0.972
81	Isopropanolamine	0.1815	0.1365	0.214	0.1665	0.0936	0.24025	0.734	0.972
82	Sarcosine	1.935	1.44	3	2.12	1.6375	2.485	0.734	0.972
83	N-acetylglucosamine 1-phosphate	0.04911	0.01252	0.13525	0.07495	0.01252	0.14125	0.735	0.972
84	7-Methylguanine	0.03445	0.02176	0.043625	0.0363	0.00544	0.045575	0.746	0.972
85	1-Methyladenosine	0.01795	0.00832	0.02575	0.01845	0.01252	0.025925	0.747	0.972
86	N^1,N^8-Diacetylspermidine	0.0214	0.0100995	0.02615	0.018	0.0113475	0.0239	0.747	0.972
87	Gly-Gly	0.0805	0.053375	0.13975	0.106	0.059875	0.14725	0.749	0.972
88	Guanosine	0.1595	0.111	0.203	0.138	0.09845	0.21025	0.749	0.972
89	17-OH-Pregnenolone	0.000228	0.00017475	0.000543	0.000334	0.00021275	0.00046175	0.749	0.972

90 4-Hydroxymandelate	0.86	0.1444	1.4575	0.999	0.1444	1.7925	0.759	0.972
91 4-Methyl-2-oxopentanoate	0.2595	0.20625	0.37975	0.2575	0.226	0.3835	0.763	0.972
92 Spermidine	0.2775	0.149	0.422	0.308	0.19375	0.41075	0.763	0.972
93 Cystine	0.03775	0.00436	0.103	0.0458	0.00436	0.069425	0.772	0.972
94 N^1,N^{12}-Diacetylspermine	0.02125	0.015375	0.0267	0.022	0.016075	0.034675	0.777	0.972
95 Lithocholic acid	0.000132	0.000107	0.000146	0.000124	0.0001075	0.00014525	0.777	0.972
96 3-Phenyllactate	0.348	0.1915	0.3665	0.322	0.195	0.3715	0.777	0.972
97 5-Oxoproline	1.755	1.53	2.495	2.07	1.535	2.3575	0.777	0.972
98 Arg	4.185	2.71	7.1125	4.725	3.9525	5.635	0.777	0.972
99 Cysteine-glutathione disulfide	0.00616	0.00616	0.105175	0.00616	0.00616	0.058375	0.778	0.972
100 ADMA	0.01575	0.0017	0.025375	0.0094	0.0017	0.022925	0.783	0.972
101 Glutathione(ox)	0.04975	0.00358	0.11625	0.03765	0.025425	0.0512	0.791	0.972
102 Proline betaine	0.0239	0.011705	0.0713	0.02525	0.01165	0.0426	0.820	0.972
103 CgA/protein（pmol/mg）	15.35	5.1575	29.6	11.8	9.23	30.425	0.821	0.972
104 Histamine	0.04005	0.032875	0.178	0.07065	0.033125	0.187	0.821	0.972
105 *N*-acetylaspartate	0.4535	0.22475	0.749	0.3885	0.24525	0.9335	0.836	0.972
106 Thymine	2.715	2.1875	3.635	2.675	1.785	3.24	0.836	0.972
107 2-Hydroxy-4-methylpentanoate	0.1655	0.0202	0.2935	0.209	0.0202	0.26375	0.848	0.972
108 3-(4-Hydroxyphenyl)propionate	1.79	1.1425	2.6875	1.74	0.84175	3.21	0.851	0.972
109 Methionine sulfoxide	0.02355	0.0074	0.071375	0.0402	0.0074	0.07185	0.858	0.972
110 Homoserine	0.07045	0.03096	0.0921	0.06605	0.00774	0.10375	0.864	0.972
111 *α*-Aminoadipate	0.4825	0.391	0.63475	0.5315	0.38725	0.64625	0.865	0.972
112 Biotin	0.0278	0.0278	0.246	0.0278	0.0278	0.2245	0.865	0.972
113 Anserine	0.03545	0.01011	0.061175	0.02405	0.018675	0.07815	0.880	0.972
114 Gly-Leu	0.04705	0.04085	0.08575	0.05435	0.0315	0.0817	0.880	0.972
115 Citrulline	1.225	0.80275	2.175	1.325	0.94225	1.725	0.880	0.972
116 Trimethylamine *N*-oxide	0.03255	0.00358	0.1285	0.03545	0.01432	0.05755	0.894	0.972
117 Cortisol	0.00135	0.00075725	0.00189	0.001055	0.0007865	0.0018975	0.895	0.972
118 SAM+	0.0182	0.003	0.03475	0.01825	0.003	0.027625	0.923	0.985
119 2-Hydroxypentanoate	0.325	0.28675	0.42225	0.3385	0.24875	0.48325	0.925	0.985
120 *γ*-Butyrobetaine	0.5655	0.442	0.668	0.5785	0.40525	0.675	0.955	0.985
121 Betaine	0.9665	0.61675	1.2175	0.835	0.71	1.17	0.955	0.985
122 Urocanate	0.3215	0.221	0.38075	0.3005	0.19075	0.4625	0.955	0.985
123 Phe	3.265	2.395	5.0775	3.595	2.2725	4.16	0.955	0.985
124 *N*-acetylornithine	0.02595	0.00468	0.03825	0.01404	0.00468	0.05235	0.984	0.985
125 Indole-3-acetate	0.907	0.578	1.16	0.868	0.6645	1.1425	0.985	0.985
126 PEP	0.2435	0.13	0.31025	0.231	0.172	0.30775	0.985	0.985
127 3-Aminoisobutyrate	0.27	0.20025	0.371	0.2455	0.2225	0.42925	0.985	0.985

[Time point: Recover]

Metabolite	Control Median	Control Q1	Control Q3	High-stress Median	High-stress Q1	High-stress Q3	*p*-value	*q*-value
1 Estrone	3.76E-06	2.72E-06	4.50E-06	2.03E-06	1.55E-06	2.50E-06	0.006	0.713
2 AICAR	0.00632	0.00632	0.01264	0.3205	0.00632	0.4865	0.018	0.713
3 Hypoxanthine	1.915	1.54	2.425	1.31	0.90675	1.855	0.027	0.713
4 Hydroxyproline	0.1945	0.16125	0.25675	0.1425	0.124	0.18575	0.032	0.713
5 *N*-acetyl-*β*-alanine	0.144	0.0224	0.17975	0.0224	0.0224	0.1365	0.033	0.713
6 2AB	0.2955	0.19475	0.3505	0.1885	0.13925	0.2815	0.044	0.713
7 Adenine	0.269	0.195	0.29675	0.188	0.138	0.227	0.046	0.713
8 Choline	3.125	2.5125	3.52	2.31	2.1175	2.8425	0.052	0.713
9 DHEA-S	0.004535	0.0035075	0.00603	0.00254	0.002015	0.00426	0.057	0.713
10 Ile	0.674	0.39275	1.035	0.3825	0.3145	0.569	0.068	0.713
11 *N*-acetylglucosamine 6-phosphate	0.03966	0.01162	0.1205	0.01162	0.01162	0.02324	0.079	0.713
12 Gln	4.165	3.5025	5.465	3.285	2.89	3.9275	0.080	0.713
13 SAM+	0.0216	0.010455	0.027475	0.01415	0.00252	0.02055	0.080	0.713
14 Cysteine-glutathione disulfide	0.0941	0.00868	0.126	0.00868	0.00868	0.0544	0.084	0.713
15 *β*-Ala	0.4045	0.3045	0.692	0.2695	0.21775	0.4935	0.086	0.713
16 Val	2.105	1.675	4.04	1.54	1.2075	2.2325	0.090	0.713
17 Inosine	0.2795	0.246	0.43275	0.2325	0.1168	0.30525	0.097	0.722
18 Adenosine	0.0649	0.0459	0.08305	0.0464	0.039225	0.071425	0.118	0.810
19 Asn	0.03478	0.00546	0.17825	0.1995	0.054275	0.2315	0.121	0.810
20 Thr	1.14	0.84025	1.44	0.924	0.757	1.04	0.142	0.834
21 PEP	0.1965	0.1525	0.27475	0.174	0.021	0.2	0.145	0.834
22 Glucosamine	0.02437	0.00654	0.0883	0.00654	0.00654	0.01308	0.147	0.834
23 Carnitine	0.42	0.33125	0.571	0.347	0.29775	0.45275	0.152	0.834
24 *N*,*N*-Dimethylglycine	0.123	0.0886	0.1665	0.09175	0.067575	0.11575	0.158	0.834
25 Sarcosine	1.495	1.2475	2.1925	1.415	0.95225	1.59	0.169	0.858
26 Guanosine	0.1435	0.10925	0.1885	0.111	0.07555	0.15125	0.181	0.883
27 GABA	0.358	0.279	0.433	0.301	0.241	0.36125	0.200	0.887
28 *N*-acetylornithine	0.00414	0.00414	0.033575	0.00414	0.00414	0.00828	0.205	0.887
29 Lithocholic acid	0.000124	0.000105025	0.0001465	0.000108	9.78E-05	0.00012525	0.206	0.887
30 Creatinine	1.095	0.79325	1.2875	0.749	0.6855	1.25	0.214	0.887
31 Glycerate	0.8335	0.62675	1.105	0.9515	0.87675	1.205	0.221	0.887
32 Leu	1.805	1.17725	2.97	1.565	1.0625	1.93	0.235	0.887
33 Creatine	4.425	3.6075	5.2275	3.705	3.245	4.22	0.235	0.887
34 1-Methyladenosine	0.0169	0.012365	0.019725	0.0121	0.001912	0.017925	0.237	0.887

35	4-Methyl-2-oxopentanoate	0.27	0.22	0.3535	0.284	0.2595	0.45675	0.258	0.888
36	His	1.76	1.455	2.5225	2.315	2.025	2.6225	0.258	0.888
37	Lys	2.3	1.89	3.1825	3.605	2.3175	4.6325	0.266	0.888
38	Isopropanolamine	0.135	0.10575	0.162	0.0964	0.068525	0.1705	0.266	0.888
39	Met	0.128	0.08975	0.29325	0.106	0.00706	0.173	0.273	0.888
40	3-Methylhistidine	0.05165	0.041325	0.084775	0.0441	0.036575	0.07005	0.283	0.898
41	Glu	6.76	5.6075	8.61	5.845	4.91	7.375	0.291	0.901
42	Ser	2.545	2.1425	3.8225	2.525	1.8275	2.96	0.318	0.901
43	Tyr	3.11	2.35	3.7	3.625	2.505	5.4575	0.337	0.901
44	Cystine	0.03425	0.0063	0.063175	0.0063	0.0063	0.046975	0.337	0.901
45	o-Acetylcarnitine	0.339	0.297	0.493	0.3985	0.349	0.56225	0.346	0.901
46	1,3-Diaminopropane	0.1235	0.0895	0.222	0.10005	0.077225	0.145	0.356	0.901
47	N^1,N^8-Diacetylspermidine	0.01555	0.010735	0.019625	0.0131	0.000932	0.0183	0.363	0.901
48	7-Methylguanine	0.0261	0.00944	0.03365	0.02285	0.00236	0.028075	0.389	0.901
49	11-Deoxycorticosterone	0.006435	0.0052375	0.007595	0.00734	0.005275	0.00781	0.396	0.901
50	Testosterone	2.87E-05	2.02E-05	8.03E-05	5.53E-05	3.13E-05	0.000101875	0.396	0.901
51	CgA/protein(pmol/mg)	14.55	8.2075	37.625	20.65	10.34	49.675	0.396	0.901
52	Hypotaurine	0.283	0.25575	0.372	0.258	0.1556	0.3525	0.405	0.901
53	5-Oxoproline	1.55	1.37	2.0825	1.445	1.2325	1.8875	0.407	0.901
54	Biotin	0.169	0.0292	0.2085	0.0292	0.0292	0.256	0.409	0.901
55	N^6,N^6,N^6-trimethyllysine	0.02365	0.001286	0.03415	0.01775	0.005144	0.026125	0.413	0.901
56	γ-Butyrobetaine	0.5015	0.34175	0.64325	0.42	0.189	0.613	0.418	0.901
57	Gly-Leu	0.0593	0.03235	0.073975	0.0379	0.025175	0.071125	0.429	0.901
58	6-Phosphogluconate	0.07665	0.0128	0.1335	0.1305	0.0512	0.15	0.430	0.901
59	3-Phenyllactate	0.302	0.159125	0.352	0.252	0.159	0.29725	0.439	0.901
60	*N*-acetylputrescine	0.3225	0.27	0.37225	0.368	0.31425	0.51025	0.440	0.901
61	Progesterone	0.0001845	9.93E-05	0.00020925	0.0001125	8.15E-05	0.000196	0.440	0.901
62	DHEA	6.88E-05	5.16E-05	0.00014025	0.00011175	6.07E-05	0.00023675	0.440	0.901
63	Ornithine	1.555	0.96125	2.595	1.905	1.465	3.135	0.451	0.903
64	Agmatine	0.0348	0.023725	0.040125	0.03985	0.02066	0.05225	0.460	0.903
65	Aldosterone	3.04E-05	8.84E-06	3.87E-05	1.90E-05	1.29E-05	3.43E-05	0.462	0.903
66	18-OH-Corticosterone	0.0002695	5.44E-05	0.0003735	0.000331	0.00011925	0.0005705	0.486	0.933
67	Trp	0.152	0.132	0.21025	0.159	0.115825	0.1945	0.509	0.933
68	Asp	2.455	2.1425	3.5325	2.45	1.9125	3.115	0.510	0.933
69	*N*-acetylglutamate	0.0485	0.00914	0.067725	0.00914	0.00914	0.067225	0.512	0.933
70	Glutathione(ox)	0.0554	0.01935	0.097575	0.04335	0.027175	0.06215	0.520	0.933
71	Methionine sulfoxide	0.02462	0.00804	0.069925	0.00804	0.00804	0.060825	0.526	0.933
72	DHT	6.79E-05	5.73E-05	0.0001295	9.30E-05	6.23E-05	0.00018975	0.559	0.933
73	Pipecolate	0.0773	0.0552	0.17125	0.0748	0.054625	0.094275	0.572	0.933
74	Tetrahydrocortisol	0.00397	0.0014625	0.0075975	0.003325	0.001695	0.006025	0.572	0.933
75	Gly-Gly	0.077	0.0425	0.10725	0.07805	0.00796	0.098425	0.583	0.933
76	4-Hydroxymandelate	0.602	0.433	0.7835	0.7035	0.0586	1.745	0.583	0.933
77	Spermidine	0.245	0.152	0.3365	0.243	0.194	0.37475	0.585	0.933
78	*N*-acetylaspartate	0.3505	0.196	0.53	0.3085	0.255	0.82375	0.585	0.933
79	Glu-Glu	0.00302	0.00302	0.041875	0.00302	0.00302	0.023325	0.603	0.933
80	Proline betaine	0.0215	0.00256	0.082625	0.02255	0.00256	0.033775	0.605	0.933
81	Citrulline	1.19	0.75075	1.8175	1.17	0.92725	1.51	0.611	0.933
82	Homoserine	0.05875	0.00352	0.06545	0.0502	0.036425	0.07865	0.621	0.933
83	N^1,N^{12}-Diacetylspermine	0.0144	0.001808	0.02005	0.01745	0.001808	0.02255	0.646	0.933
84	3-(4-Hydroxyphenyl)propionate	1.55	1.02175	2.1475	1.775	0.70975	2.44	0.664	0.933
85	Pregnenolone	0.0001875	0.00014	0.00038475	0.00021	0.00016725	0.0003515	0.665	0.933
86	Azelate	0.1205	0.107	0.143	0.128	0.100975	0.1675	0.665	0.933
87	Arg	3.735	2.1575	6.0875	4.41	3.04	6.01	0.665	0.933
88	Tetrahydrocortisone	0.0004825	0.00018475	0.0023025	0.0010595	0.00022725	0.0020475	0.665	0.933
89	Cortisone	0.00446	0.0029825	0.006365	0.004395	0.0024	0.00687	0.665	0.933
90	17-OH-Pregnenolone	0.00034	0.00013625	0.0004545	0.0003595	0.00019825	0.00055325	0.665	0.933
91	*N*-acetylglucosamine 1-phosphate	0.0821	0.01152	0.129	0.09585	0.01152	0.14775	0.674	0.933
92	sIgA(μg/mL)	43.5	39.225	63.275	55.6	39.075	65.5	0.692	0.933
93	3-Aminoisobutyrate	0.2335	0.1395	0.3215	0.1985	0.128	0.28625	0.692	0.933
94	Urocanate	0.2535	0.2045	0.3865	0.2775	0.1375	0.39275	0.692	0.933
95	Galacturonate 1-phosphate	0.00952	0.00952	0.082325	0.03701	0.00952	0.096625	0.698	0.933
96	Glycolate	2.915	2.235	3.445	2.75	2.4075	3.8875	0.720	0.941
97	Indole-3-acetate	0.798	0.60675	1.115	0.7735	0.0574	1.12425	0.747	0.941
98	Cytosine	0.04955	0.02256	0.06445	0.05145	0.044025	0.057475	0.748	0.941
99	Corticosterone	4.05E-05	2.94E-05	6.42E-05	4.19E-05	2.70E-05	4.89E-05	0.749	0.941
100	2-Hydroxypentanoate	0.3825	0.2825	0.505	0.4025	0.245	0.79025	0.749	0.941
101	25-OHD	0.000116	8.86E-05	0.000177	9.82E-05	7.09E-05	0.00022575	0.749	0.941
102	Homovanillate	0.5345	0.31125	0.7445	0.6085	0.28875	0.674	0.762	0.949
103	*N*-ε-acetyllysine	0.0421	0.037	0.059625	0.04405	0.0066	0.05855	0.776	0.954
104	Nicotinate	0.245	0.2115	0.28575	0.217	0.023	0.38575	0.790	0.954
105	Androstenediol	0.0004935	0.0002955	0.000876	0.000473	0.00033425	0.00065275	0.792	0.954
106	Anserine	0.01845	0.00232	0.056375	0.00696	0.00232	0.06515	0.796	0.954
107	Cortisol	0.001165	0.00078425	0.0022325	0.001185	0.00066325	0.0023575	0.836	0.992
108	17-OHP	5.77E-05	3.35E-05	7.75E-05	4.23E-05	2.98E-05	9.34E-05	0.865	1.000

109	4-Androstene-3,17-dione	4.44E-05	2.91E-05	6.73E-05	4.89E-05	2.63E-05	8.93E-05	0.865	1.000
110	Trimethylamine *N*-oxide	0.0393	0.006696	0.1285	0.0407	0.0212	0.055275	0.895	1.000
111	Thymine	2.255	1.8225	2.9625	2.515	1.8675	2.9225	0.895	1.000
112	Betaine	0.827	0.67475	1.2825	0.778	0.6665	1.08675	0.895	1.000
113	Phe	2.455	2.0275	3.35	2.435	1.9725	3.31	0.895	1.000
114	CMP	0.1985	0.15175	0.25825	0.1805	0.14775	0.289	0.910	1.000
115	Cytidine	0.09965	0.027	0.1395	0.10445	0.05125	0.14875	0.910	1.000
116	ADMA	0.004368	0.001456	0.017825	0.001456	0.001456	0.0199	0.919	1.000
117	11-Deoxycortisol	1.02E-05	6.39E-06	2.65E-05	1.27E-05	5.06E-06	2.02E-05	0.925	1.000
118	Carnosine	0.01755	0.00197	0.0322	0.01585	0.00197	0.0283	0.938	1.000
119	Glycerophosphate	4.185	3.4625	5.6675	4.435	3.8125	5.7875	0.955	1.000
120	α-Aminoadipate	0.3495	0.30725	0.45125	0.386	0.275	0.42325	0.970	1.000
121	Cholesterol	0.02795	0.0024075	0.037575	0.02305	0.0038	0.041575	0.985	1.000
122	17β-Estradiol	7.01E-06	5.62E-06	1.06E-05	7.01E-06	5.27E-06	8.38E-06	0.985	1.000
123	Histamine	0.04385	0.03365	0.13575	0.04325	0.0314	0.12525	0.985	1.000
124	Estriol	5.10E-05	1.17E-05	0.00010225	4.87E-05	9.94E-06	0.000116025	0.985	1.000
125	2-Hydroxy-4-methylpentanoate	0.276	0.18025	0.35425	0.252	0.1565	0.405	1.000	1.000
126	Threonate	0.3295	0.0506	0.5295	0.35	0.0506	0.50725	1.000	1.000
127	Ala-Ala	0.0946	0.01324	0.126	0.09135	0.01324	0.138	1.000	1.000

[Time point: Post-WM]

	Metabolite	Control Median	Control Q1	Control Q3	High-stress Median	High-stress Q1	High-stress Q3	*p*-value	*q*-value
1	N^6,N^6,N^6-trimethyllysine	0.0301	0.025075	0.039425	0.01805	0.00189	0.031175	0.009	0.513
2	Choline	3.34	2.3525	3.9075	2.1	1.675	2.625	0.015	0.513
3	Cystine	0.04835	0.02592	0.080475	0.00648	0.00648	0.0355	0.019	0.513
4	DHEA	0.0001465	8.55E-05	0.000287	7.71E-05	4.32E-05	0.00013325	0.020	0.513
5	Val	2.095	1.6325	3.9375	1.675	1.0625	1.9025	0.024	0.513
6	Ile	0.5535	0.42475	1.1975	0.4295	0.23775	0.518	0.030	0.513
7	Glucosamine	0.0479	0.02464	0.10145	0.00616	0.00616	0.042825	0.032	0.513
8	Cysteine-glutathione disulfide	0.06585	0.00586	0.10725	0.00586	0.00586	0.014945	0.032	0.513
9	β-Ala	0.4255	0.279	0.697	0.2645	0.23925	0.42975	0.048	0.603
10	Adenine	0.2655	0.19975	0.31225	0.1845	0.15725	0.23625	0.048	0.603
11	2AB	0.2725	0.22625	0.403	0.1825	0.14625	0.244	0.052	0.603
12	*N*-acetyl-β-alanine	0.1695	0.0254	0.24	0.0762	0.0254	0.15725	0.073	0.606
13	Glu-Glu	0.018	0.006	0.04795	0.006	0.006	0.006	0.079	0.606
14	Estrone	3.16E-06	2.13E-06	4.81E-06	1.87E-06	1.29E-06	3.79E-06	0.080	0.606
15	GABA	0.393	0.27375	0.45125	0.29	0.2555	0.3525	0.101	0.606
16	Adenosine	0.0721	0.056825	0.086175	0.0455	0.038475	0.0744	0.101	0.606
17	Thr	1.125	0.93975	1.5575	0.8715	0.76325	1.11	0.105	0.606
18	Leu	1.695	1.17	3.365	1.54	0.8105	1.7275	0.109	0.606
19	Creatine	4.445	3.515	5.465	3.39	2.9125	4.585	0.109	0.606
20	DHEA-S	0.00422	0.0028575	0.0065725	0.00279	0.00175	0.0045325	0.109	0.606
21	Glu	6.57	5.775	10.525	5.39	4.7875	7.0925	0.118	0.606
22	Isopropanolamine	0.1605	0.1085	0.19375	0.1075	0.083975	0.16	0.118	0.606
23	*N*-acetylglucosamine 1-phosphate	0.05985	0.01182	0.1405	0.135	0.08245	0.14625	0.118	0.606
24	Hypoxanthine	1.895	1.4325	2.5125	1.35	0.8985	2.0475	0.122	0.606
25	SAM+	0.01995	0.0096	0.02465	0.0024	0.0024	0.020325	0.123	0.606
26	Met	0.154	0.077775	0.243	0.0824	0.00592	0.171	0.130	0.606
27	*N*-acetylornithine	0.0267	0.00368	0.0453	0.00368	0.00368	0.025775	0.136	0.606
28	Hydroxyproline	0.1965	0.145	0.28375	0.165	0.1255	0.185	0.136	0.606
29	Gln	4.365	2.8225	6.8	2.9	2.5775	4.2275	0.147	0.606
30	Asp	2.75	2.01	4.23	2.18	1.69	3.3825	0.147	0.606
31	Ser	2.8	1.88	3.84	2.16	1.685	2.8275	0.157	0.606
32	Guanosine	0.1445	0.101925	0.19	0.1235	0.08255	0.16425	0.157	0.606
33	γ-Butyrobetaine	0.563	0.365	0.75675	0.45	0.21875	0.61475	0.158	0.606
34	Sarcosine	1.66	1.29	2.225	1.5	1.02975	1.625	0.169	0.631
35	Gly-Gly	0.0868	0.0547	0.115	0.0644	0.00352	0.10525	0.186	0.657
36	1-Methyladenosine	0.0181	0.0106025	0.02125	0.01295	0.010425	0.016725	0.186	0.657
37	*N*-ε-acetyllysine	0.03865	0.0071	0.055875	0.048	0.04115	0.0616	0.210	0.683
38	Creatinine	1.075	0.7435	1.275	0.7755	0.6315	1.1675	0.214	0.683
39	Phe	2.905	2.2075	3.525	2.37	2.07	2.7175	0.214	0.683
40	Cortisol	0.00124	0.00069	0.0020925	0.000658	0.00047525	0.001645	0.221	0.683
41	Carnitine	0.3835	0.33675	0.622	0.361	0.27275	0.445	0.221	0.683
42	Nicotinate	0.2575	0.20275	0.3585	0.1925	0.0236	0.439	0.265	0.768
43	N^1,N^8-Diacetylspermidine	0.019	0.0102125	0.02655	0.0165	0.0076425	0.0201	0.265	0.768
44	1,3-Diaminopropane	0.1555	0.091425	0.20075	0.1055	0.082025	0.13475	0.266	0.768
45	Glutathione(ox)	0.04495	0.02415	0.0867	0.0293	0.00426	0.051	0.277	0.777
46	17beta-Estradiol	7.54E-06	5.07E-06	9.00E-06	6.20E-06	4.59E-06	8.05E-06	0.283	0.777
47	Gly-Leu	0.07055	0.029325	0.08955	0.0407	0.026125	0.076825	0.300	0.777
48	17-OH-Pregnenolone	0.000281	0.00019825	0.000611	0.0002675	0.00014075	0.0003945	0.300	0.777
49	11-Deoxycorticosterone	0.00678	0.0056225	0.00845	0.00651	0.0053175	0.0070475	0.318	0.777
50	Pipecolate	0.0868	0.05485	0.17175	0.0662	0.056075	0.09355	0.327	0.777
51	Hypotaurine	0.2675	0.115855	0.394	0.231	0.01642	0.313	0.331	0.777
52	Agmatine	0.0311	0.027225	0.04385	0.03785	0.034675	0.04695	0.336	0.777

53	Citrulline	1.225	0.781	2.7275	0.95	0.78525	1.3525	0.336	0.777
54	3-Methylhistidine	0.055	0.047225	0.106975	0.0512	0.03785	0.0751	0.337	0.777
55	11-Deoxycortisol	1.33E-05	5.74E-06	2.39E-05	8.38E-06	4.12E-06	1.48E-05	0.337	0.777
56	Glycerophosphate	4.48	3.5725	6.24	3.815	3.04	4.97	0.346	0.783
57	Trp	0.1765	0.1265	0.2115	0.1705	0.1025	0.19125	0.376	0.783
58	3-Phenyllactate	0.2975	0.1955	0.3815	0.278	0.20025	0.30525	0.376	0.783
59	*N*-acetylaspartate	0.521	0.247	0.678	0.269	0.2005	0.556	0.376	0.783
60	Tetrahydrocortisol	0.00319	0.001695	0.00504	0.002285	0.001075	0.0049575	0.376	0.783
61	PEP	0.189	0.09505	0.218	0.1365	0.1265	0.18825	0.383	0.783
62	Galacturonate 1-phosphate	0.03612	0.01204	0.1035	0.01204	0.01204	0.0898	0.385	0.783
63	Tetrahydrocortisone	0.0003015	0.0001605	0.002155	0.0002365	0.0001385	0.00129	0.396	0.783
64	Asn	0.01584	0.00528	0.17775	0.08565	0.00528	0.2195	0.402	0.783
65	7-Methylguanine	0.0275	0.016725	0.04065	0.0238	0.0036	0.030525	0.402	0.783
66	Cortisone	0.00448	0.0033825	0.008415	0.0037	0.00291	0.0052375	0.407	0.783
67	*N*,*N*-dimethylglycine	0.1165	0.0766	0.15225	0.1035	0.084225	0.11875	0.417	0.787
68	Betaine	0.9575	0.6585	1.22	0.833	0.538	1.045	0.429	0.787
69	Anserine	0.029	0.004224	0.0496	0.0138	0.001056	0.046425	0.430	0.787
70	4-Methyl-2-oxopentanoate	0.289	0.17625	0.336	0.298	0.22525	0.426	0.440	0.787
71	18-OH-Corticosterone	0.0001415	7.06E-05	0.000519	8.42E-05	6.65E-05	0.00018875	0.440	0.787
72	Proline betaine	0.0246	0.0024	0.072125	0.01365	0.0024	0.036225	0.447	0.788
73	Cholesterol	0.005765	0.004215	0.026275	0.004555	0.0025225	0.028175	0.462	0.804
74	Ala-Ala	0.1064	0.067	0.149	0.106	0.03424	0.12925	0.483	0.811
75	4-Hydroxymandelate	1.065	0.74675	1.4425	0.8185	0.37525	1.4175	0.485	0.811
76	2-Hydroxypentanoate	0.332	0.286	0.55	0.2875	0.2205	0.732	0.486	0.811
77	Aldosterone	1.97E-05	7.17E-06	4.42E-05	1.46E-05	9.93E-06	2.25E-05	0.510	0.840
78	Methionine sulfoxide	0.00818	0.00818	0.064675	0.00818	0.00818	0.053625	0.516	0.840
79	3-Aminoisobutyrate	0.2415	0.1555	0.303	0.176	0.1445	0.29125	0.534	0.859
80	AICAR	0.0602	0.0602	0.38025	0.0602	0.0602	0.322	0.548	0.870
81	Cytosine	0.05365	0.042925	0.078425	0.0501	0.044325	0.061225	0.559	0.876
82	o-Acetylcarnitine	0.363	0.27075	0.5005	0.3195	0.249	0.43	0.585	0.906
83	Inosine	0.2745	0.20925	0.40325	0.266	0.1585	0.3065	0.611	0.924
84	N^1,N^{12}-Diacetylspermine	0.0209	0.01465	0.026525	0.0192	0.010137	0.02485	0.623	0.924
85	5-Oxoproline	1.925	1.3375	2.2625	1.535	1.35	1.715	0.624	0.924
86	Estriol	2.91E-05	1.24E-05	5.74E-05	1.88E-05	1.33E-05	3.51E-05	0.637	0.924
87	4-Androstene-3,17-dione	6.05E-05	2.19E-05	8.24E-05	7.09E-05	2.43E-05	8.64E-05	0.638	0.924
88	Threonate	0.466	0.286	0.64475	0.4545	0.056	0.53125	0.662	0.924
89	Indole-3-acetate	0.7195	0.5805	1.065	0.7265	0.1072	1.6225	0.663	0.924
90	Androstenediol	0.0003805	0.00028375	0.00072575	0.000381	0.000271	0.00055875	0.665	0.924
91	His	1.605	1.485	2.4875	2.14	1.475	2.5325	0.678	0.924
92	Urocanate	0.326	0.18525	0.45175	0.247	0.14075	0.43975	0.692	0.924
93	DHT	9.38E-05	5.84E-05	0.00013075	7.38E-05	5.31E-05	0.0001415	0.692	0.924
94	6-Phosphogluconate	0.03984	0.01328	0.1415	0.0724	0.01328	0.132	0.705	0.924
95	Progesterone	0.000124	7.43E-05	0.000154	0.0001035	7.15E-05	0.00015375	0.720	0.924
96	Arg	3.44	2.5425	6.3125	3.675	2.5525	5.43	0.720	0.924
97	Lys	2.675	1.67	4.52	3.355	2.655	3.8325	0.720	0.924
98	Testosterone	3.59E-05	1.21E-05	7.67E-05	3.16E-05	8.29E-06	6.84E-05	0.720	0.924
99	17-OHP	4.94E-05	2.72E-05	8.14E-05	4.23E-05	2.86E-05	6.38E-05	0.720	0.924
100	*N*-acetylglutamate	0.05775	0.00794	0.079475	0.0506	0.03176	0.06925	0.745	0.941
101	CgA/protein(pmol/mg)	16.7	11.85	63.65	25	12.55	54.975	0.749	0.941
102	Pregnenolone	0.00021	0.00015425	0.00028175	0.0002175	0.000164	0.000281	0.763	0.949
103	Tyr	3.405	2.5275	4.325	3.83	3.265	4.1025	0.777	0.949
104	Spermidine	0.2465	0.176	0.36925	0.2785	0.21425	0.34075	0.777	0.949
105	CMP	0.1845	0.09245	0.27525	0.159	0.0872	0.2405	0.820	0.965
106	Lithocholic acid	0.000111	9.90E-05	0.00012975	0.000114	0.000102	0.0001495	0.821	0.965
107	Carnosine	0.0112	0.003	0.033025	0.01635	0.003	0.031475	0.829	0.965
108	Cytidine	0.1045	0.07405	0.1925	0.10585	0.067275	0.14875	0.836	0.965
109	3-(4-Hydroxyphenyl)propionate	1.995	1.14	2.8575	1.6	0.7115	3.1025	0.836	0.965
110	Corticosterone	4.13E-05	3.16E-05	4.73E-05	4.20E-05	3.46E-05	4.72E-05	0.836	0.965
111	ADMA	0.00966	0.001778	0.016575	0.005694	0.001778	0.023975	0.859	0.973
112	Azelate	0.118	0.10475	0.15925	0.121	0.107	0.14975	0.865	0.973
113	α-Aminoadipate	0.365	0.314	0.4725	0.363	0.24525	0.4635	0.865	0.973
114	Trimethylamine N-oxide	0.02985	0.015775	0.0934	0.0333	0.01945	0.05385	0.880	0.980
115	Histamine	0.0363	0.032575	0.13875	0.04255	0.028775	0.1021	0.895	0.988
116	sIgA(μg/mL)	48.45	38.925	66.025	51.25	40.75	58.9	0.910	0.995
117	Biotin	0.151	0.0234	0.18525	0.0702	0.0234	0.2185	0.921	0.995
118	*N*-acetylputrescine	0.329	0.2645	0.48525	0.3635	0.245	0.4985	0.925	0.995
119	Glycolate	2.74	2.285	3.5125	2.645	2.24	3.7275	0.940	0.995
120	2-Hydroxy-4-methylpentanoate	0.272	0.14725	0.3425	0.281	0.17625	0.346	0.940	0.995
121	*N*-acetylglucosamine 6-phosphate	0.03312	0.01104	0.11	0.04952	0.01104	0.115	0.952	0.999
122	Ornithine	1.715	1.11	2.8975	1.91	1.2675	2.29	0.970	1.000
123	Homovanillate	0.6365	0.44975	0.8725	0.6645	0.6115	0.786	1.000	1.000
124	25-OHD	0.0001465	0.000105075	0.0002365	0.0001595	8.20E-05	0.0002455	1.000	1.000
125	Glycerate	0.795	0.62925	0.96425	0.775	0.72	1.03	1.000	1.000
126	Homoserine	0.04975	0.00848	0.075925	0.04845	0.00848	0.06025	1.000	1.000
127	Thymine	2.03	1.57	3.28	2.15	1.69	2.385	1.000	1.000

Table S5. Metabolites with significant temporal changes (Friedman test)

Group	Number of metabolites	Metabolites
Control group	15	PEP, Creatinine, α-Aminoadipate, 3-Aminoisobutyrate, sIgA, Glu, Sarcosine, Isopropanolamine, *N*-Acetylputrescine, His, Anserine, GABA, Hypoxanthine, Pipecolate, 3-Methylhistidine
High-stress group	43	3-Aminoisobutyrate, Sarcosine, Creatinine, β-Alanine, Hydroxyproline, α-Aminoadipate, *N*-acetylputrescine, GABA, His, 2AB, Gly-Gly, Phe, Glu-Glu, Agmatine, CgA/protein, Anserine, Isopropanolamine, Carnitine, Galacturonate 1-phosphate, Guanosine, Cortisone, Hypoxanthine, Glu, Val, PEP, Glucosamine, 2-Hydroxypentanoate, 3-Methylhistidine, Pipecolate, Choline, Homovanillate, Tyr, 5-Oxoproline, Cytidine, N^6,N^6,N^6-trimethyllysine, Citrulline, Creatine, Ile, *N*-acetylornithine, Histamine, 7-Methylguanine, sIgA, *N*-acetylglutamate
All participants	68	Creatinine, 3-Aminoisobutyrate, Sarcosine, α-Aminoadipate, *N*-acetylputrescine, His, PEP, GABA, Glu, β-Alanine, Hydroxyproline, Isopropanolamine, Anserine, sIgA, Hypoxanthine, Phe, Val, 2AB, 5-Oxoproline, Agmatine, Pipecolate, CgA/protein, 3-Methylhistidine, Creatine, Citrulline, Glu-Glu, Ile, DHEA-S, Ornithine, Lys, Tyr, Leu, Adenosine, Homovanillate, Cytidine, Gly-Gly, Choline, N^1,N^{12}-diacetylspermine, Threonate, Glucosamine, Glycerophosphate, CMP, 3-(4-Hydroxyphenyl)propionate, 3-Phenyllactate, Guanosine, 4-Hydroxymandelate, Carnitine, Galacturonate 1-phosphate, 6-Phosphogluconate, 2-Hydroxypentanoate, Histamine, N^6,N^6,N^6-trimethyllysine, Trp, Cortisone, Gly-Leu, *N*-acetyl-β-alanine, Carnosine, *N*-ε-acetyllysine, *N*-acetylglutamate, Homoserine, Cystine, Thymine, Asn, Ala-Ala, γ-Butyrobetaine, Inosine, 2-Hydroxy-4-methylpentanoate, 7-Methylguanine

Metabolites listed showed significant temporal variation across the four time points, as determined by the Friedman test with a false discovery rate correction ($q < 0.05$).

2AB, 2-aminobutanoic acid; Asn, asparagine; CgA, chromogranin A; DHEA-S, dehydroepiandrosterone sulfate; GABA, γ-aminobutanoic acid; Glu, glutamic acid; His, histidine; Ile, isoleucine; Leu, leucine; Lys, lysine; PEP, phosphoenolpyruvate; Phe, phenylalanine; sIgA, secretory immunoglobulin A; Trp, tryptophan; Tyr, tyrosine; Val, valine.

TableS6. Full results of the longitudinal analysis by group (Friedman test)

[Group: Control group]

	Metabolite	Rest Median	Rest Q1	Rest Q3	Post-UK Median	Post-UK Q1	Post-UK Q3	Recover Median	Recover Q1	Recover Q3	Post-WM Median	Post-WM Q1	Post-WM Q3	*p* -value	*q* -value
1	PEP	0.2805	0.2155	0.3855	0.2435	0.13	0.31025	0.1965	0.1525	0.27475	0.189	0.09341	0.218	< 0.001	0.003
2	Creatinine	1.155	0.95	1.4075	1.215	0.9115	1.6225	1.095	0.79325	1.2875	1.075	0.7435	1.275	< 0.001	0.003
3	α-Aminoadipate	0.4555	0.3425	0.57325	0.4825	0.391	0.63475	0.3495	0.30725	0.45125	0.365	0.314	0.4725	< 0.001	0.013
4	3-Aminoisobutyrate	0.252	0.16025	0.3525	0.27	0.20025	0.371	0.2335	0.1395	0.3215	0.2415	0.1555	0.303	< 0.001	0.013
5	sIgA(μg/mL)	41	36.1	50.625	54.95	43.7	76.025	43.5	39.225	63.275	48.45	38.925	66.025	0.001	0.013
6	Glu	9.14	6.92	12.225	8.575	6.5125	10.8	6.76	5.6075	8.61	6.57	5.775	10.525	0.001	0.013
7	Sarcosine	1.52	1.245	2.405	1.935	1.44	3	1.495	1.2475	2.1925	1.66	1.29	2.225	0.001	0.013
8	Isopropanolamine	0.1135	0.074825	0.1505	0.1815	0.1365	0.214	0.135	0.10575	0.162	0.1605	0.1085	0.19375	0.001	0.014
9	*N*-acetylputrescine	0.3165	0.253	0.58625	0.402	0.35	0.57225	0.3225	0.27	0.37225	0.329	0.2645	0.48525	0.001	0.015
10	His	2.38	1.9325	3.075	2.31	1.73	3.03	1.76	1.455	2.5225	1.605	1.485	2.4875	0.001	0.016
11	Anserine	0.02445	0.009114	0.073425	0.03545	0.01011	0.061175	0.01845	0.001056	0.056375	0.029	0.004224	0.0496	0.002	0.018
12	GABA	0.4135	0.26875	0.427	0.479	0.39075	0.555	0.358	0.279	0.433	0.393	0.27375	0.45125	0.002	0.020
13	Hypoxanthine	2.51	1.425	3.06	2.405	1.47	3.1475	1.915	1.54	2.425	1.895	1.4325	2.5125	0.003	0.030
14	Pipecolate	0.06775	0.055775	0.1255	0.0912	0.072	0.20125	0.0773	0.0552	0.17125	0.0868	0.05485	0.17175	0.004	0.033
15	3-Methylhistidine	0.06265	0.0461	0.123	0.06265	0.054275	0.124	0.05165	0.041325	0.084775	0.055	0.047225	0.106975	0.005	0.044
16	CgA/protein(pmol/mg)	24.15	5.36	53.8	15.35	5.1575	29.6	14.55	8.2075	37.625	16.7	11.85	63.65	0.007	0.053
17	5-Oxoproline	1.87	1.5925	2.3875	1.755	1.53	2.495	1.55	1.37	2.0825	1.925	1.3375	2.2625	0.008	0.055
18	3-(4-Hydroxyphenyl)propionate	1.68	1.08	2.2325	1.79	1.1425	2.6875	1.55	1.02175	2.1475	1.995	1.14	2.8575	0.008	0.055
19	Lys	3.505	2.2	6.56	3.22	2.0475	3.8875	2.3	1.89	3.1825	2.675	1.67	4.52	0.008	0.055
20	Adenosine	0.06705	0.055425	0.080775	0.07925	0.06315	0.102325	0.0649	0.0459	0.08305	0.0721	0.056825	0.086175	0.009	0.060
21	CMP	0.2395	0.1795	0.36175	0.2665	0.1651	0.34375	0.1985	0.15175	0.25825	0.1845	0.09114	0.27525	0.012	0.065
22	Hydroxyproline	0.229	0.18325	0.2825	0.2545	0.18075	0.3315	0.1945	0.16125	0.25675	0.1965	0.145	0.28375	0.012	0.065
23	Citrulline	1.83	0.941	2.1825	1.225	0.80275	2.175	1.19	0.75075	1.8175	1.225	0.781	2.7275	0.013	0.065
24	Val	2.68	1.29	4.8925	2.695	1.6325	4.6325	2.105	1.675	4.04	2.095	1.6325	3.9375	0.013	0.065
25	DHEA-S	0.00432	0.0036925	0.006765	0.00516	0.00427	0.0074425	0.004535	0.0035075	0.00603	0.00422	0.0028575	0.0065725	0.013	0.065
26	Asn	0.127	0.00528	0.228	0.162	0.00528	0.24675	0.03469	0.00528	0.17825	0.01584	0.00528	0.17775	0.014	0.067
27	Glycerophosphate	4.24	3.75	5.325	5.11	4.58	6.685	4.185	3.4625	5.6675	4.48	3.5725	6.24	0.014	0.067
28	β-Ala	0.403	0.3305	0.71775	0.48	0.395	0.759	0.4045	0.3045	0.692	0.4255	0.279	0.697	0.018	0.077
29	AICAR	0.00632	0.00632	0.07074	0.353	0.00632	0.40625	0.00632	0.00632	0.01264	0.00632	0.00632	0.38025	0.018	0.077
30	Creatine	4.5	3.7125	6.885	5.015	4.19	6.7775	4.425	3.6075	5.2275	4.445	3.515	5.465	0.018	0.077
31	Ornithine	2.4	1.18	3.5675	1.825	1.1275	3.905	1.555	0.96125	2.595	1.715	1.11	2.8975	0.023	0.094
32	N^1,N^{12}-Diacetylspermine	0.01865	0.013105	0.0256	0.02125	0.015375	0.0267	0.0144	0.00175	0.02005	0.0209	0.01465	0.026525	0.024	0.095
33	Corticosterone	5.64E-05	3.47E-05	8.19E-05	5.13E-05	4.58E-05	6.54E-05	4.05E-05	2.94E-05	6.42E-05	4.13E-05	3.16E-05	4.73E-05	0.026	0.102
34	3-Phenyllactate	0.2885	0.13425	0.38275	0.348	0.1915	0.3665	0.302	0.159125	0.352	0.2975	0.1955	0.3815	0.028	0.103
35	Leu	2.07	1.245	4.5925	2.505	1.2425	4.4825	1.805	1.17725	2.97	1.695	1.17	3.365	0.030	0.110
36	4-Hydroxymandelate	0.8265	0.614	1.4325	0.86	0.035	1.4575	0.602	0.433	0.7835	1.065	0.74675	1.4425	0.031	0.111
37	Trp	0.188	0.1115	0.334	0.204	0.1585	0.24875	0.152	0.132	0.21025	0.1765	0.1265	0.2115	0.033	0.112
38	Ile	0.9135	0.37975	1.51	0.8665	0.36375	1.5175	0.674	0.39275	1.035	0.5535	0.42475	1.1975	0.034	0.112
39	Progesterone	0.0001225	7.16E-05	0.0001605	0.000176	0.00013	0.0001875	0.0001845	9.93E-05	0.00020925	0.000124	7.43E-05	0.000154	0.035	0.113
40	Agmatine	0.0357	0.027075	0.045875	0.03585	0.034125	0.049525	0.0348	0.023725	0.040125	0.0311	0.027225	0.04385	0.037	0.118
41	Phe	2.98	2.1075	4.4425	3.265	2.395	5.0775	2.455	2.0275	3.35	2.905	2.2075	3.525	0.041	0.128
42	Met	0.118	0.047005	0.2145	0.191	0.1315	0.33925	0.128	0.08975	0.29325	0.154	0.077775	0.243	0.051	0.154
43	Cytidine	0.111	0.02758	0.13025	0.1185	0.00232	0.167	0.09965	0.027	0.1395	0.1045	0.07405	0.1925	0.060	0.178
44	Glutathione(ox)	0.06135	0.0305	0.118	0.04975	0.00358	0.11625	0.0554	0.01935	0.097575	0.04495	0.02415	0.0867	0.064	0.182
45	2-Hydroxy-4-methylpentanoate	0.2325	0.132	0.307	0.1655	0.01304	0.2935	0.276	0.18025	0.35425	0.272	0.14725	0.3425	0.065	0.182
46	Cytosine	0.0533	0.01656	0.070725	0.06405	0.04435	0.0909	0.04955	0.022185	0.06445	0.05365	0.042925	0.078425	0.071	0.196
47	Inosine	0.288	0.120815	0.38975	0.329	0.2805	0.42025	0.2795	0.246	0.43275	0.2745	0.20925	0.40325	0.074	0.200
48	Threonate	0.4465	0.0506	0.59375	0.4655	0.357	1.02	0.3295	0.0506	0.5295	0.466	0.286	0.64475	0.075	0.200
49	25-OHD	0.0001035	5.56E-05	0.00031775	6.34E-05	5.05E-05	0.0001175	0.000116	8.86E-05	0.000177	0.0001465	0.000105075	0.0002365	0.089	0.228
50	Glu-Glu	0.04375	0.00302	0.06425	0.0452	0.02018	0.062725	0.00302	0.00302	0.041875	0.01651	0.00302	0.04795	0.094	0.228
51	γ-Butyrobetaine	0.4515	0.341	0.67	0.5655	0.442	0.668	0.5015	0.34175	0.64325	0.563	0.365	0.75675	0.095	0.228
52	Homovanillate	0.5595	0.34775	0.86475	0.5055	0.29025	0.96525	0.5345	0.31125	0.7445	0.6365	0.44975	0.8725	0.095	0.228
53	2AB	0.281	0.1975	0.45225	0.358	0.2075	0.428	0.2955	0.19475	0.3505	0.2725	0.22625	0.403	0.095	0.228
54	6-Phosphogluconate	0.131	0.054335	0.1555	0.102	0.01274	0.134	0.07665	0.01274	0.1335	0.03957	0.01274	0.1415	0.097	0.228
55	Cystine	0.01898	0.00436	0.0555	0.03775	0.00436	0.103	0.03425	0.00436	0.063175	0.04835	0.02539	0.080475	0.106	0.245
56	DHEA	9.59E-05	4.58E-05	0.000221	0.0001775	0.00013075	0.0002635	6.88E-05	5.16E-05	0.00014025	0.0001465	8.55E-05	0.000287	0.112	0.253
57	Carnosine	0.0242	0.0125675	0.03445	0.0228	0.00197	0.032775	0.01755	0.00197	0.0322	0.010685	0.00197	0.033025	0.123	0.270
58	Tyr	3.765	2.43	6.32	3.715	2.92	5.56	3.11	2.35	3.7	3.405	2.5275	4.325	0.123	0.270
59	Tetrahydrocortisol	0.0026	0.00110725	0.00854	0.004815	0.0035375	0.00854	0.00397	0.0014625	0.0075975	0.00319	0.001695	0.00504	0.127	0.270
60	Histamine	0.0427	0.029625	0.1365	0.04005	0.032875	0.178	0.04385	0.03365	0.13575	0.0363	0.032575	0.13875	0.128	0.270
61	Asp	3.01	2.17	3.6825	2.955	2.1325	3.825	2.455	2.1425	3.5325	2.75	2.01	4.23	0.140	0.287
62	Choline	3.335	2.83	4.175	3.41	3.01	4.1575	3.125	2.5125	3.52	3.34	2.3525	3.9075	0.140	0.287
63	Glycerate	1.05	0.83625	1.18	0.75	0.62825	0.84025	0.8335	0.62675	1.105	0.795	0.62925	0.96425	0.149	0.301
64	*N*-ε-acetyllysine	0.0416	0.0066	0.058175	0.05395	0.042875	0.081625	0.0421	0.037	0.059625	0.03865	0.0066	0.055875	0.156	0.309
65	Spermidine	0.257	0.18225	0.35175	0.2775	0.149	0.422	0.245	0.152	0.3365	0.2465	0.176	0.36925	0.183	0.358
66	Homoserine	0.07385	0.00352	0.09125	0.07045	0.029905	0.0921	0.05875	0.00352	0.06545	0.04975	0.00352	0.075925	0.197	0.379
67	*N*-acetylaspartate	0.3955	0.18775	0.64975	0.4535	0.22475	0.749	0.3505	0.196	0.53	0.521	0.247	0.678	0.209	0.396
68	Glucosamine	0.06235	0.00484	0.13375	0.07045	0.042225	0.12825	0.02352	0.00484	0.0883	0.0479	0.02431	0.10145	0.217	0.403
69	Ser	3.135	2.4175	4.4875	2.725	2.21	4.57	2.545	2.1425	3.8225	2.8	1.88	3.84	0.219	0.403
70	Arg	4.62	2.7025	6.0975	4.185	2.71	7.1125	3.735	2.1575	6.0875	3.44	2.5425	6.3125	0.226	0.410
71	*N*-acetyl-β-alanine	0.1985	0.16625	0.27325	0.1855	0.0224	0.23575	0.144	0.0224	0.17975	0.1695	0.0224	0.24	0.233	0.412
72	Gly-Gly	0.0762	0.02548	0.123	0.0805	0.053375	0.13975	0.077	0.0425	0.10725	0.0868	0.0547	0.115	0.237	0.412
73	Ala-Ala	0.10165	0.05404	0.15875	0.0967	0.00856	0.115	0.0946	0.00856	0.126	0.1064	0.067	0.149	0.240	0.412
74	Cortisone	0.005705	0.0041975	0.0081325	0.004205	0.0032925	0.0065125	0.00446	0.0029825	0.006365	0.00448	0.0033825	0.008415	0.240	0.412
75	Gly-Leu	0.06625	0.040025	0.08745	0.04705	0.04085	0.08575	0.0593	0.03235	0.073975	0.07055	0.029325	0.08955	0.248	0.416
76	Hypotaurine	0.3065	0.187	0.41975	0.3415	0.25525	0.5285	0.283	0.25575	0.372	0.2675	0.115855	0.394	0.249	0.416
77	Thymine	2.265	1.885	3.0575	2.715	2.1875	3.635	2.255	1.8225	2.9625	2.03	1.57	3.28	0.264	0.430
78	Betaine	0.8295	0.47125	0.99725	0.9665	0.61675	1.2175	0.827	0.67475	1.2825	0.9575	0.6585	1.22	0.264	0.430
79	ADMA	0.001456	0.001456	0.0182	0.01575	0.001456	0.025375	0.004368	0.001456	0.017825	0.00966	0.001456	0.016575	0.267	0.430
80	11-Deoxycorticosterone	0.00687	0.0049625	0.0084925	0.007545	0.00674	0.0082475	0.006435	0.0052375	0.007595	0.00678	0.0056225	0.00845	0.276	0.433
81	Thr	1.09	0.86575	1.885	1.22	0.86175	1.6525	1.14	0.84025	1.44	1.125	0.93975	1.5575	0.276	0.433
82	N^6,N^6,N^6-trimethyllysine	0.02855	0.02145	0.036225	0.03415	0.022625	0.041425	0.02365	0.001286	0.03415	0.0301	0.025075	0.039425	0.285	0.441
83	Indole-3-acetate	0.856	0.661	1.05	0.907	0.578	1.16	0.798	0.60675	1.115	0.7195	0.5805	1.065	0.293	0.449
84	17-OHP	7.43E-05	4.65E-05	9.02E-05	6.08E-05	2.72E-05	9.14E-05	5.77E-05	3.35E-05	7.75E-05	4.94E-05	2.72E-05	8.14E-05	0.299	0.451
85	*N*,*N*-dimethylglycine	0.1235	0.097175	0.16475	0.1375	0.10195	0.157	0.123	0.0886	0.1665	0.1165	0.0766	0.15225	0.302	0.451
86	Nicotinate	0.251	0.17225	0.30625	0.2815	0.14615	0.3675	0.245	0.2115	0.28575	0.2575	0.20275	0.3585	0.336	0.491
87	*N*-acetylglucosamine 6-phosphate	0.0924	0.01104	0.13125	0.01104	0.01104	0.11225	0.03937	0.01104	0.1205	0.03312	0.01104	0.11	0.336	0.491
88	Gln	4.5	3.415	6.8225	4.955	3.4775	6.5475	3.5025	5.465		4.365	2.8225	6.8	0.347	0.501
89	Cholesterol	0.0153	0.003215	0.04745	0.03975	0.007545	0.046625	0.02795	0.0024075	0.037575	0.005765	0.004215	0.026275	0.358	0.511
90	Testosterone	4.52E-05	2.09E-05	6.48E-05	5.10E-05	1.80E-05	0.000215	2.87E-05	2.02E-05	8.03E-05	3.59E-05	1.21E-05	7.67E-05	0.363	0.513
91	18-OH-Corticosterone	0.000136	8.68E-05	0.000476	0.0003745	0.000154	0.00051825	0.0002695	5.44E-05	0.0003735	0.0001415	7.06E-05	0.000519	0.369	0.515
92	1,3-Diaminopropane	0.109	0.085625	0.246	0.129	0.08955	0.22725	0.1235	0.0895	0.222	0.1555	0.091425	0.20075	0.386	0.532
93	7-Methylguanine	0.02675	0.012965	0.0339	0.03445	0.02099	0.043625	0.0261	0.00944	0.03365	0.0275	0.016415	0.04065	0.413	0.555
94	Androstenediol	0.0005425	0.0002715	0.00080125	0.000762	0.00040675	0.0008805	0.0004935	0.0002955	0.000876	0.0003805	0.00028375	0.00072575	0.415	0.555
95	Aldosterone	1.62E-05	1.17E-05	4.31E-05	2.75E-05	1.48E-05	4.52E-05	3.04E-05	8.84E-06	3.87E-05	1.97E-05	7.17E-06	4.42E-05	0.415	0.555
96	Guanosine	0.1625	0.098925	0.24775	0.1595	0.111	0.203	0.1435	0.10925	0.1885	0.1445	0.101925	0.19	0.453	0.599
97	Adenine	0.198	0.14675	0.29725	0.265	0.21525	0.3405	0.269	0.195	0.29675	0.2655	0.19975	0.31225	0.460	0.602
98	*N*-acetylornithine	0.00322	0.00322	0.0374	0.02595	0.00322	0.03825	0.00322	0.00322	0.033575	0.0267	0.00322	0.0453	0.470	0.608
99	Azelate	0.1325	0.11225	0.14625	0.131	0.107	0.16075	0.1205	0.107	0.143	0.118	0.10475	0.15925	0.474	0.608
100	*N*-acetylglutamate	0.06005	0.03686	0.084475	0.02867	0.00794	0.0718	0.0485	0.00794	0.067725	0.05775	0.00794	0.079475	0.482	0.613
101	Glycolate	3.05	2.6075	3.39	2.905	2.585	4.215	2.915	2.235	3.445	2.74	2.285	3.5125	0.508	0.634
102	4-Methyl-2-oxopentanoate	0.337	0.2335	0.48425	0.2595	0.20625	0.37975	0.27	0.22	0.3535	0.289	0.17625	0.336	0.510	0.634
103	Galacturonate 1-phosphate	0.08175	0.009	0.13025	0.0893	0.009	0.1205	0.009	0.009	0.082325	0.0346	0.009	0.1035	0.525	0.647
104	Pregnenolone	0.0002555	0.000167	0.000329	0.000307	0.00020975	0.00036125	0.0001875	0.00014	0.00038475	0.00021	0.00015425	0.00028175	0.537	0.656
105	Cysteine-glutathione disulfide	0.081	0.00586	0.12625	0.00586	0.00586	0.105175	0.0941	0.00586	0.126	0.06585	0.00586	0.10725	0.546	0.660
106	17β-Estradiol	7.93E-06	4.36E-06	1.23E-05	8.82E-06	6.12E-06	1.20E-05	7.01E-06	5.62E-06	1.06E-05	7.54E-06	5.07E-06	9.00E-06	0.552	0.661
107	o-Acetylcarnitine	0.3575	0.2305	0.5235	0.4115	0.28425	0.56725	0.339	0.297	0.493	0.363	0.27075	0.5005	0.561	0.666
108	Tetrahydrocortisone	0.000794	0.000207	0.001805	0.001865	0.00031725	0.0020925	0.0004825	0.00018475	0.0023025	0.0003015	0.0001605	0.002155	0.567	0.667

109	Trimethylamine *N*-oxide	0.0286	0.0114435	0.13525	0.03255	0.001674	0.1285	0.0393	0.006696	0.1285	0.02985	0.015775	0.0934	0.577	0.672
110	2-Hydroxypentanoate	0.4915	0.2885	0.613	0.325	0.28675	0.42225	0.3825	0.2825	0.505	0.332	0.286	0.55	0.599	0.691
111	Carnitine	0.4785	0.37775	0.6895	0.4515	0.4115	0.6235	0.42	0.33125	0.571	0.3835	0.33675	0.622	0.631	0.722
112	DHT	8.75E-05	5.95E-05	0.00015725	9.79E-05	7.02E-05	0.000139	6.79E-05	5.73E-05	0.0001295	9.38E-05	5.84E-05	0.00013075	0.648	0.735
113	Estriol	3.58E-05	1.81E-05	7.47E-05	4.23E-05	2.33E-05	6.91E-05	5.10E-05	1.17E-05	0.00010225	2.91E-05	1.24E-05	5.74E-05	0.665	0.747
114	Methionine sulfoxide	0.0377	0.006	0.087625	0.02285	0.006	0.071375	0.0236	0.006	0.069925	0.006	0.006	0.064675	0.674	0.751
115	11-Deoxycortisol	1.44E-05	7.59E-06	2.43E-05	1.90E-05	7.93E-06	3.25E-05	1.02E-05	6.39E-06	2.65E-05	1.33E-05	5.74E-06	2.39E-05	0.700	0.766
116	4-Androstene-3,17-dione	4.45E-05	3.13E-05	5.86E-05	5.99E-05	3.45E-05	6.91E-05	4.44E-05	2.91E-05	6.73E-05	6.05E-05	2.19E-05	8.24E-05	0.700	0.766
117	Cortisol	0.00145	0.00083075	0.002185	0.00135	0.00075725	0.00189	0.001165	0.00078425	0.0022325	0.00124	0.00069	0.0020925	0.717	0.766
118	Lithocholic acid	0.000127	0.0001095	0.00015	0.000132	0.000107	0.000146	0.000124	0.000105025	0.0001465	0.000111	9.90E-05	0.00012975	0.717	0.766
119	Biotin	0.0234	0.0234	0.233	0.0234	0.0234	0.246	0.169	0.0234	0.2085	0.151	0.0234	0.18525	0.724	0.766
120	SAM+	0.0208	0.0024	0.028075	0.0182	0.0024	0.03475	0.0216	0.010425	0.027475	0.01995	0.0096	0.02465	0.724	0.766
121	Urocanate	0.2975	0.22975	0.4305	0.3215	0.221	0.38075	0.2535	0.2045	0.3865	0.326	0.18525	0.45175	0.745	0.782
122	N^1,N^8-diacetylspermidine	0.0184	0.012875	0.0235	0.0214	0.009983	0.02615	0.01555	0.010735	0.019625	0.019	0.0102125	0.02655	0.798	0.831
123	1-Methyladenosine	0.0165	0.001642	0.028725	0.01795	0.0082105	0.02575	0.0169	0.012365	0.019725	0.0181	0.0106025	0.02125	0.809	0.836
124	17-OH-Pregnenolone	0.0003445	0.00011525	0.00046875	0.000228	0.00017475	0.000543	0.00034	0.00013625	0.0004545	0.000281	0.00019825	0.000611	0.843	0.864
125	*N*-acetylglucosamine 1-phosphate	0.1035	0.05598	0.14625	0.04861	0.01152	0.13525	0.0821	0.01152	0.129	0.05985	0.01152	0.1405	0.855	0.868
126	Estrone	2.96E-06	2.19E-06	4.66E-06	2.94E-06	2.05E-06	5.14E-06	3.76E-06	2.72E-06	4.50E-06	3.16E-06	2.13E-06	4.81E-06	0.879	0.886
127	Proline betaine	0.02215	0.01328	0.05445	0.0239	0.011705	0.0713	0.0215	0.00212	0.082625	0.0246	0.00212	0.072125	0.996	0.996

[Group: High-stress group]

	Metabolite	Rest Median	Rest Q1	Rest Q3	Post-UK Median	Post-UK Q1	Post-UK Q3	Recover Median	Recover Q1	Recover Q3	Post-WM Median	Post-WM Q1	Post-WM Q3	*p*-value	*q*-value
1	3-Aminoisobutyrate	0.26	0.14275	0.3545	0.2455	0.2225	0.42925	0.1985	0.128	0.28625	0.176	0.1445	0.29125	< 0.001	0.001
2	Sarcosine	1.76	1.195	1.9725	2.12	1.6375	2.485	1.415	0.95225	1.59	1.5	1.02975	1.625	< 0.001	0.001
3	Creatinine	0.9725	0.81025	1.5875	0.9145	0.84525	1.4575	0.749	0.6855	1.25	0.7755	0.6315	1.1675	< 0.001	0.001
4	β-Ala	0.3325	0.238	0.59775	0.3925	0.33775	0.66925	0.2695	0.21775	0.4935	0.2645	0.23925	0.42975	< 0.001	0.001
5	Hydroxyproline	0.1835	0.1555	0.214	0.209	0.17775	0.24725	0.1425	0.124	0.18575	0.165	0.1255	0.185	< 0.001	0.002
6	α-Aminoadipate	0.5035	0.325	0.60325	0.5315	0.38725	0.64625	0.386	0.275	0.42325	0.363	0.24525	0.4635	< 0.001	0.002
7	*N*-acetylputrescine	0.4775	0.30825	0.6615	0.553	0.38975	0.66975	0.368	0.31425	0.51025	0.3635	0.245	0.4985	< 0.001	0.002
8	GABA	0.3535	0.22275	0.4145	0.455	0.3335	0.499	0.301	0.241	0.36125	0.29	0.2555	0.3525	< 0.001	0.002
9	His	2.905	2.105	3.1575	2.73	2.3175	3.4175	2.315	2.025	2.6225	2.14	1.475	2.5325	< 0.001	0.003
10	2AB	0.225	0.14175	0.32575	0.272	0.19925	0.36575	0.1885	0.13925	0.2815	0.1825	0.14625	0.244	< 0.001	0.003
11	Gly-Gly	0.0821	0.056725	0.11575	0.106	0.059875	0.14725	0.07805	0.00352	0.098425	0.0644	0.00352	0.10525	< 0.001	0.005
12	Phe	2.755	2.2575	4.75	3.595	2.2725	4.16	2.435	1.9725	3.31	2.37	2.07	2.7175	0.001	0.006
13	Glu-Glu	0.04585	0.021005	0.055475	0.02311	0.00302	0.05945	0.00302	0.00302	0.023325	0.00302	0.00302	0.00302	0.001	0.008
14	Agmatine	0.043	0.034525	0.062825	0.05105	0.035825	0.064125	0.03985	0.02011	0.05225	0.03785	0.034675	0.04695	0.001	0.010
15	CgA/protein(pmol/mg)	8.67	6.415	29.825	11.8	9.23	30.425	20.65	10.34	49.675	25	12.55	54.975	0.002	0.014
16	Anserine	0.001056	0.001056	0.06945	0.02405	0.018675	0.07815	0.006328	0.001056	0.06515	0.0138	0.001056	0.046425	0.002	0.014
17	Isopropanolamine	0.106	0.0843	0.205	0.1665	0.0936	0.24025	0.0964	0.068525	0.1705	0.1075	0.083975	0.16	0.002	0.014
18	Carnitine	0.4235	0.3315	0.5265	0.4485	0.35125	0.54025	0.347	0.29775	0.45275	0.361	0.27275	0.445	0.002	0.014
19	Galacturonate 1-phosphate	0.07155	0.049275	0.1365	0.105	0.04155	0.1235	0.03675	0.009	0.096625	0.009	0.009	0.0898	0.003	0.014
20	Guanosine	0.1029	0.060975	0.20175	0.138	0.09845	0.21025	0.111	0.07555	0.15125	0.1235	0.08255	0.16425	0.003	0.014
21	Cortisone	0.00625	0.00475	0.00883	0.00522	0.0037975	0.007455	0.004395	0.0024	0.00687	0.0037	0.00291	0.0052375	0.003	0.014
22	Hypoxanthine	1.66	1.01275	2.6125	1.7	1.1425	2.49	1.31	0.90675	1.855	1.35	0.8985	2.0475	0.003	0.014
23	Glu	7.395	6.7425	10.5	8.015	6.0675	8.9975	5.845	4.91	7.375	5.39	4.7875	7.0925	0.003	0.014
24	Val	1.89	1.31	2.7775	1.96	1.3625	2.83	1.54	1.2075	2.2325	1.675	1.0625	1.9025	0.003	0.014
25	PEP	0.241	0.16275	0.3735	0.231	0.172	0.30775	0.174	0.01664	0.2	0.1365	0.1265	0.18825	0.003	0.014
26	Glucosamine	0.0532	0.00484	0.084275	0.0571	0.00484	0.12	0.00484	0.00484	0.011805	0.00484	0.00484	0.042825	0.003	0.014
27	2-Hydroxypentanoate	0.505	0.36775	0.736	0.3385	0.24875	0.48325	0.4025	0.245	0.79025	0.2875	0.2205	0.732	0.003	0.016
28	3-Methylhistidine	0.05835	0.03855	0.07865	0.0582	0.045375	0.092225	0.0441	0.036575	0.07005	0.0512	0.03785	0.0751	0.005	0.023
29	Pipecolate	0.07725	0.05875	0.1135	0.09795	0.064725	0.12625	0.0748	0.054625	0.094275	0.0662	0.056075	0.09355	0.005	0.023
30	Choline	2.985	2.0425	3.375	2.915	2.2025	3.4075	2.31	2.1175	2.8425	2.1	1.675	2.625	0.005	0.023
31	Homovanillate	0.635	0.54525	0.85125	0.7635	0.6595	0.8905	0.6085	0.28875	0.674	0.6645	0.6115	0.786	0.006	0.023
32	Tyr	4.45	3.1025	6.99	4.055	3.2925	6.325	3.625	2.505	5.4575	3.83	3.265	4.1025	0.006	0.024
33	5-Oxoproline	1.99	1.28	2.4375	2.07	1.535	2.3575	1.445	1.2325	1.8875	1.535	1.35	1.715	0.009	0.035
34	Cytidine	0.1045	0.048525	0.15625	0.113	0.0873	0.18325	0.10445	0.05125	0.14875	0.10585	0.067275	0.14875	0.011	0.040
35	N^6,N^6,N^6-trimethyllysine	0.0197	0.001286	0.0338	0.0276	0.022875	0.03615	0.01775	0.005144	0.026125	0.01805	0.001286	0.031175	0.011	0.040
36	Citrulline	1.44	1.24125	1.7425	1.325	0.94225	1.725	1.17	0.92725	1.51	0.95	0.78525	1.3525	0.012	0.041
37	Creatine	4.08	3.29	5.7975	4.515	3.7975	5.4075	3.705	3.245	4.22	3.39	2.9125	4.585	0.012	0.041
38	Ile	0.5005	0.278	0.668	0.439	0.348	0.77875	0.3825	0.3145	0.569	0.4295	0.23775	0.518	0.012	0.041
39	*N*-acetylornithine	0.00966	0.00322	0.03935	0.01331	0.00322	0.05235	0.00322	0.00322	0.00759	0.00322	0.00322	0.025775	0.012	0.041
40	Histamine	0.044	0.026325	0.12925	0.07065	0.033125	0.187	0.04325	0.0314	0.12525	0.04255	0.028775	0.1021	0.013	0.042
41	7-Methylguanine	0.034	0.022875	0.041525	0.0363	0.00236	0.045575	0.02285	0.00236	0.028075	0.0238	0.00236	0.030525	0.014	0.042
42	sIgA(μg/mL)	43.75	36.625	51.975	64.35	51.6	73.1	55.6	39.075	65.5	51.25	40.75	58.9	0.015	0.046
43	*N*-acetylglutamate	0.06625	0.00794	0.084625	0.0637	0.00794	0.082	0.00794	0.00794	0.067225	0.0506	0.03176	0.06925	0.017	0.049
44	Gly-Leu	0.0468	0.037375	0.0826	0.05435	0.0315	0.0817	0.0379	0.025175	0.071125	0.0407	0.026125	0.076825	0.023	0.068
45	*N*-acetyl-β-alanine	0.0224	0.0224	0.23575	0.163	0.0224	0.2	0.0224	0.0224	0.1365	0.0747	0.0224	0.15725	0.026	0.074
46	DHEA-S	0.00372	0.0026975	0.0050075	0.00352	0.0023675	0.0056225	0.00254	0.002015	0.00426	0.00279	0.00175	0.0045325	0.030	0.084
47	3-(4-Hydroxyphenyl)propionate	1.94	0.58025	2.435	1.74	0.84175	3.21	1.775	0.70975	2.44	1.6	0.7115	3.1025	0.031	0.085
48	Ornithine	2.685	1.5375	3.74	2.055	1.7125	3.825	1.905	1.465	3.135	1.91	1.2675	2.29	0.036	0.095
49	Threonate	0.1633	0.0506	0.5695	0.465	0.0506	0.68925	0.35	0.0506	0.50725	0.4545	0.0506	0.53125	0.041	0.107
50	Glycerate	0.658	0.44075	1.0975	0.8165	0.662	1.055	0.9515	0.87675	1.205	0.775	0.72	1.03	0.043	0.108
51	Proline betaine	0.01536	0.00212	0.03585	0.02525	0.01165	0.0426	0.02255	0.00212	0.033775	0.01365	0.00212	0.036225	0.046	0.114
52	Leu	1.585	1.0325	2.335	1.54	1.35	2.6175	1.565	1.0625	1.93	1.54	0.8105	1.7275	0.054	0.131
53	Adenosine	0.05415	0.0439	0.076425	0.059	0.05025	0.08515	0.0464	0.039225	0.071425	0.0455	0.038475	0.0744	0.060	0.143
54	N^1,N^{12}-Diacetylspermine	0.01735	0.00175	0.027525	0.022	0.016075	0.034675	0.01745	0.00175	0.02255	0.0192	0.0101125	0.02485	0.069	0.163
55	o-Acetylcarnitine	0.413	0.38625	0.6195	0.483	0.348	0.652	0.3985	0.349	0.56225	0.3195	0.249	0.43	0.073	0.168
56	1-Methyladenosine	0.016	0.0095605	0.022925	0.01845	0.0124105	0.025925	0.0121	0.001642	0.017925	0.01295	0.010425	0.016725	0.077	0.175
57	Thymine	2.32	1.37475	3.83	2.675	1.785	3.24	2.515	1.8675	2.9225	2.15	1.69	2.385	0.080	0.177
58	Ala-Ala	0.116	0.050065	0.143	0.1495	0.06735	0.17575	0.09135	0.00856	0.138	0.106	0.03424	0.12925	0.081	0.177
59	6-Phosphogluconate	0.1335	0.112	0.16625	0.1065	0.06505	0.1765	0.1305	0.051185	0.15	0.0724	0.01274	0.132	0.086	0.186
60	3-Phenyllactate	0.2385	0.18975	0.3495	0.322	0.195	0.3715	0.252	0.159	0.29725	0.278	0.20025	0.30525	0.092	0.194
61	*N*,*N*-dimethylglycine	0.0996	0.081675	0.123	0.112	0.09455	0.14025	0.09175	0.067575	0.11575	0.1035	0.084225	0.11875	0.094	0.194
62	Lys	3.98	3.005	6.7675	3.49	2.8275	5.27	3.605	2.3175	4.6325	3.355	2.655	3.8325	0.095	0.194
63	4-Hydroxymandelate	1.0875	0.46625	1.9575	0.999	0.035	1.7925	0.7035	0.035	1.745	0.8185	0.37525	1.4175	0.101	0.203
64	*N*-ε-acetyllysine	0.0513	0.03905	0.060775	0.0637	0.046925	0.086725	0.04405	0.0066	0.05855	0.048	0.04115	0.0616	0.106	0.207
65	Cystine	0.00436	0.00436	0.0496	0.0458	0.00436	0.069425	0.00436	0.00436	0.046975	0.00436	0.00436	0.0355	0.107	0.207
66	N^1,N^8-diacetylspermidine	0.0157	0.008333	0.0278	0.018	0.0113475	0.0239	0.0131	0.000932	0.0183	0.0165	0.007493	0.0201	0.107	0.207
67	Homoserine	0.07025	0.00352	0.088825	0.06605	0.00352	0.10375	0.0502	0.036425	0.07865	0.04845	0.00352	0.06025	0.113	0.213
68	Indole-3-acetate	0.928	0.0574	1.345	0.868	0.6645	1.1425	0.7735	0.0574	1.12425	0.7265	0.0574	1.6225	0.114	0.213
69	17-OHP	9.20E-05	3.11E-05	0.00011675	4.19E-05	2.31E-05	7.59E-05	4.23E-05	2.98E-05	9.34E-05	4.23E-05	2.86E-05	6.38E-05	0.118	0.218
70	Cortisol	0.00165	0.0010125	0.002315	0.001055	0.0007865	0.0018975	0.001185	0.00066325	0.0023575	0.000658	0.00047525	0.001645	0.131	0.238
71	CMP	0.28	0.14925	0.45075	0.1785	0.08289	0.34825	0.1805	0.14775	0.289	0.159	0.08589	0.2405	0.140	0.250
72	*N*-acetylglucosamine 6-phosphate	0.01104	0.01104	0.099	0.01104	0.01104	0.080725	0.01104	0.01104	0.022805	0.04952	0.01104	0.115	0.143	0.250
73	Azelate	0.124	0.102225	0.15425	0.142	0.11575	0.168	0.128	0.100975	0.1675	0.121	0.107	0.14975	0.144	0.250
74	γ-Butyrobetaine	0.4335	0.231	0.72925	0.5785	0.40525	0.675	0.42	0.189	0.613	0.45	0.21875	0.61475	0.149	0.257
75	Carnosine	0.01765	0.00197	0.0382	0.02315	0.0083675	0.0462	0.01585	0.00197	0.0283	0.01635	0.00197	0.031475	0.153	0.259
76	Ser	2.995	2.0575	3.695	2.815	1.93	3.355	2.525	1.8275	2.96	2.16	1.685	2.8275	0.159	0.266
77	*N*-acetylaspartate	0.315	0.21125	0.7195	0.3885	0.24525	0.9335	0.3085	0.255	0.82375	0.269	0.2005	0.556	0.170	0.280
78	Nicotinate	0.217	0.0146	0.4135	0.1205	0.0146	0.25125	0.217	0.0146	0.38575	0.1925	0.0146	0.439	0.176	0.287
79	Trp	0.166	0.13375	0.20925	0.171	0.13575	0.2435	0.159	0.115825	0.1945	0.1705	0.1025	0.19125	0.186	0.296
80	SAM+	0.0184	0.0024	0.026475	0.01825	0.0024	0.027625	0.01415	0.0024	0.02055	0.0024	0.0024	0.020325	0.187	0.296
81	Asp	2.39	2.1425	3.06	2.455	2.2075	3.04	2.45	1.9125	3.115	2.18	1.69	3.3825	0.206	0.319
82	Glycerophosphate	4.865	3.8925	5.855	5.185	4.285	6.25	4.435	3.8125	5.7875	3.815	3.04	4.97	0.206	0.319
83	AICAR	0.15866	0.00632	0.4565	0.11285	0.00632	0.40875	0.3205	0.00632	0.4865	0.00632	0.00632	0.322	0.213	0.326
84	Methionine sulfoxide	0.006	0.006	0.070525	0.0402	0.006	0.07185	0.006	0.006	0.060825	0.006	0.006	0.053625	0.222	0.336
85	Gln	3.965	2.465	6.5475	3.79	3.54	5.23	3.285	2.89	3.9275	2.9	2.5775	4.2275	0.226	0.338
86	Urocanate	0.3025	0.20125	0.42075	0.3005	0.19075	0.4625	0.2775	0.1375	0.39275	0.247	0.14075	0.43975	0.248	0.367
87	Thr	1.12	0.71075	1.5025	1.0185	0.88125	1.2725	0.924	0.757	1.04	0.8715	0.76325	1.11	0.264	0.379
88	Lithocholic acid	0.000111	9.97E-05	0.0001385	0.000124	0.0001075	0.00014525	0.000108	9.78E-05	0.00012525	0.000114	0.000102	0.0001495	0.264	0.379
89	Hypotaurine	0.2895	0.133855	0.41425	0.342	0.19675	0.437	0.258	0.150355	0.3525	0.231	0.01642	0.313	0.266	0.379
90	Inosine	0.03678	0.01226	0.29925	0.2795	0.19225	0.42375	0.2325	0.112565	0.30525	0.266	0.1585	0.3065	0.269	0.379

91	Estrone	2.30E-06	1.71E-06	4.17E-06	2.11E-06	1.25E-06	3.11E-06	2.03E-06	1.55E-06	2.50E-06	1.87E-06	1.29E-06	3.79E-06	0.272	0.380
92	Adenine	0.203	0.117725	0.24575	0.1915	0.157	0.30025	0.188	0.138	0.227	0.1845	0.15725	0.23625	0.298	0.412
93	25-OHD	0.0001535	9.89E-05	0.00028575	0.000182	0.00011325	0.0002635	9.82E-05	7.09E-05	0.00022575	0.0001595	8.20E-05	0.0002455	0.308	0.421
94	Testosterone	5.57E-05	8.06E-06	0.00011375	3.34E-05	1.03E-05	6.13E-05	5.53E-05	3.13E-05	0.000101875	3.16E-05	8.29E-06	6.84E-05	0.318	0.429
95	Betaine	0.835	0.74275	1.0675	0.835	0.71	1.17	0.778	0.6665	1.08675	0.833	0.538	1.045	0.337	0.451
96	17-OH-Pregnenolone	0.0002795	0.0001037	0.00055875	0.000334	0.00021275	0.00046175	0.0003595	0.00019825	0.00055325	0.0002675	0.00014075	0.0003945	0.358	0.461
97	Estriol	2.27E-05	1.20E-05	2.86E-05	1.48E-05	7.62E-06	6.79E-05	4.87E-05	9.94E-06	0.000116025	1.88E-05	1.33E-05	3.51E-05	0.358	0.461
98	Androstenediol	0.0005095	0.00029175	0.000823	0.000291	0.0002475	0.00060125	0.000473	0.00033425	0.00065275	0.000381	0.000271	0.00055875	0.358	0.461
99	Asn	0.182	0.00528	0.4085	0.161	0.076925	0.334	0.1995	0.054275	0.2315	0.08565	0.00528	0.2195	0.360	0.461
100	2-Hydroxy-4-methylpentanoate	0.243	0.16425	0.44175	0.209	0.01304	0.26375	0.252	0.1565	0.405	0.281	0.17625	0.346	0.398	0.505
101	Met	0.1345	0.030355	0.2595	0.154	0.062975	0.22575	0.106	0.00592	0.173	0.0824	0.00592	0.171	0.412	0.515
102	4-Methyl-2-oxopentanoate	0.418	0.237	0.5125	0.2575	0.226	0.3835	0.284	0.2595	0.45675	0.298	0.22525	0.426	0.415	0.515
103	Glutathione(ox)	0.03905	0.00358	0.084425	0.03765	0.025425	0.0512	0.04335	0.027175	0.06215	0.0293	0.00358	0.051	0.417	0.515
104	Aldosterone	1.43E-05	8.64E-06	2.19E-05	1.72E-05	1.20E-05	2.97E-05	1.90E-05	1.29E-05	3.43E-05	1.46E-05	9.93E-06	2.25E-05	0.428	0.522
105	Pregnenolone	0.0002335	0.000115	0.000349	0.000227	0.0001465	0.00036575	0.00021	0.00016725	0.0003515	0.0002175	0.000164	0.000281	0.447	0.541
106	Progesterone	0.000167	9.74E-05	0.00018125	0.0001146	3.13E-05	0.00018725	0.0001125	8.15E-05	0.000196	0.0001035	7.15E-05	0.00015375	0.480	0.571
107	*N*-acetylglucosamine 1-phosphate	0.1215	0.01152	0.15725	0.07495	0.01152	0.14125	0.09585	0.01152	0.14775	0.135	0.08245	0.14625	0.481	0.571
108	Arg	4.85	3.8975	5.4425	4.725	3.9525	5.635	4.41	3.04	6.01	3.675	2.5525	5.43	0.508	0.597
109	ADMA	0.0159	0.001456	0.02155	0.0094	0.001456	0.022925	0.001456	0.001456	0.0199	0.005533	0.001456	0.023975	0.516	0.602
110	Trimethylamine *N*-oxide	0.0365	0.0239685	0.08225	0.03545	0.0138435	0.05755	0.0407	0.0212	0.055275	0.0333	0.01945	0.05385	0.528	0.607
111	Cytosine	0.04815	0.035725	0.06295	0.05645	0.040825	0.070425	0.05145	0.044025	0.057475	0.0501	0.044325	0.061225	0.530	0.607
112	18-OH-Corticosterone	0.000275	9.14E-05	0.00063475	0.0001565	5.81E-05	0.0003365	0.000331	0.00011925	0.0005705	8.42E-05	6.65E-05	0.00018875	0.552	0.626
113	Spermidine	0.2495	0.17875	0.39625	0.308	0.19375	0.41075	0.243	0.194	0.37475	0.2785	0.21425	0.34075	0.567	0.638
114	Glycolate	3.535	2.4475	3.9825	3.065	2.5575	3.515	2.75	2.4075	3.8875	2.645	2.24	3.7275	0.600	0.669
115	Tetrahydrocortisone	0.0005745	0.00020125	0.001785	0.00031	0.00018075	0.0012325	0.0010595	0.00022725	0.0020475	0.0002365	0.0001385	0.00129	0.631	0.697
116	Corticosterone	4.81E-05	2.74E-05	7.82E-05	3.65E-05	1.72E-05	5.36E-05	4.19E-05	2.70E-05	4.89E-05	4.20E-05	3.46E-05	4.72E-05	0.648	0.710
117	11-Deoxycorticosterone	0.00653	0.005505	0.0071425	0.00661	0.0055375	0.007835	0.00734	0.005275	0.00781	0.00651	0.0053175	0.0070475	0.682	0.741
118	17*β*-Estradiol	7.90E-06	5.44E-06	1.02E-05	4.96E-06	4.38E-06	7.78E-06	7.01E-06	5.27E-06	8.38E-06	6.20E-06	4.59E-06	8.05E-06	0.700	0.753
119	11-Deoxycortisol	1.03E-05	5.33E-06	2.28E-05	6.63E-06	5.63E-06	1.35E-05	1.27E-05	5.06E-06	2.02E-05	8.38E-06	4.12E-06	1.48E-05	0.771	0.816
120	DHEA	0.0001365	9.48E-05	0.00020575	0.0001185	2.91E-05	0.00019	0.00011175	6.07E-05	0.00023675	7.71E-05	4.32E-05	0.00013325	0.771	0.816
121	Cysteine-glutathione disulfide	0.00586	0.00586	0.07365	0.00586	0.00586	0.058375	0.00586	0.00586	0.0544	0.00586	0.00586	0.014945	0.804	0.834
122	4-Androstene-3,17-dione	4.70E-05	2.85E-05	8.75E-05	6.75E-05	3.48E-05	7.86E-05	4.89E-05	2.63E-05	8.93E-05	7.09E-05	2.43E-05	8.64E-05	0.807	0.834
123	Biotin	0.0234	0.0234	0.222	0.0234	0.0234	0.2245	0.0234	0.0234	0.256	0.0702	0.0234	0.2185	0.807	0.834
124	Cholesterol	0.009695	0.0033025	0.0341	0.006495	0.00377	0.0242	0.02305	0.0038	0.041575	0.004555	0.0025225	0.028175	0.843	0.864
125	DHT	0.00010125	3.82E-05	0.0001925	7.86E-05	4.69E-05	0.0001595	9.30E-05	6.23E-05	0.00018975	7.38E-05	5.31E-05	0.0001415	0.995	0.998
126	Tetrahydrocortisol	0.00346	0.00112	0.007715	0.003725	0.00137575	0.00492	0.003325	0.001695	0.006025	0.002285	0.001075	0.0049575	0.995	0.998
127	1,3-Diaminopropane	0.1095	0.042375	0.2415	0.1039	0.07955	0.19475	0.10005	0.077225	0.145	0.1055	0.082025	0.13475	0.998	0.998

[Group: All participants]

	Metabolite	Rest Median	Rest Q1	Rest Q3	Post-UK Median	Post-UK Q1	Post-UK Q3	Recover Median	Recover Q1	Recover Q3	Post-WM Median	Post-WM Q1	Post-WM Q3	*p*-value	*q*-value
1	Creatinine	1.025	0.84025	1.4675	1.16	0.85375	1.6225	0.9315	0.7	1.2725	0.9575	0.6475	1.2275	< 0.001	< 0.001
2	3-Aminoisobutyrate	0.258	0.14275	0.354	0.262	0.2145	0.42125	0.2075	0.13275	0.29725	0.2365	0.14575	0.303	< 0.001	< 0.001
3	Sarcosine	1.645	1.2275	2.0725	2.09	1.52	2.74	1.445	1.0625	1.785	1.525	1.2575	1.8975	< 0.001	< 0.001
4	*α*-Aminoadipate	0.477	0.325	0.60325	0.497	0.38725	0.64625	0.3615	0.27575	0.43075	0.365	0.29625	0.4635	< 0.001	< 0.001
5	*N*-acetylputrescine	0.3765	0.2555	0.6615	0.4515	0.35475	0.65475	0.3385	0.29025	0.46675	0.3565	0.2535	0.4985	< 0.001	< 0.001
6	His	2.505	2.045	3.1575	2.395	2.0475	3.2475	2.12	1.49	2.6225	1.825	1.485	2.5325	< 0.001	< 0.001
7	PEP	0.259	0.179	0.3795	0.234	0.1555	0.30775	0.1795	0.12675	0.2385	0.157	0.11825	0.21175	< 0.001	< 0.001
8	GABA	0.371	0.2345	0.427	0.4625	0.355	0.52375	0.3285	0.2585	0.38825	0.3235	0.26175	0.4125	< 0.001	< 0.001
9	Glu	8.15	6.92	11.1	8.225	6.3975	9.7175	6.48	4.9175	7.5825	6.18	4.95	8.27	< 0.001	< 0.001
10	*β*-Ala	0.3815	0.274	0.6115	0.464	0.34125	0.6795	0.37	0.237	0.5175	0.329	0.25575	0.465	< 0.001	< 0.001
11	Hydroxyproline	0.1945	0.15775	0.25	0.2165	0.1785	0.27525	0.1735	0.1305	0.19975	0.1765	0.1295	0.21725	< 0.001	< 0.001
12	Isopropanolamine	0.109	0.075225	0.1635	0.1815	0.11725	0.21475	0.117	0.0916	0.162	0.1235	0.09675	0.19075	< 0.001	< 0.001
13	Anserine	0.02285	0.001056	0.071025	0.02575	0.016675	0.068525	0.0129	0.001056	0.06515	0.0216	0.001056	0.0496	< 0.001	< 0.001
14	sIgA(μg/mL)	43.45	36.175	50.925	60.5	46.4	74.975	46.25	39.15	65.15	49.8	40.375	62.4	< 0.001	< 0.001
15	Hypoxanthine	1.98	1.2275	2.9125	2.215	1.15	2.5425	1.615	1.0045	2.0725	1.535	0.93025	2.24	< 0.001	< 0.001
16	Phe	2.88	2.1675	4.75	3.305	2.2725	4.6725	2.455	1.99	3.35	2.51	2.1075	3.13	< 0.001	< 0.001
17	Val	2.015	1.29	3.64	2.185	1.5725	3.5625	1.845	1.385	2.3925	1.875	1.2425	2.2625	< 0.001	< 0.001
18	2AB	0.2495	0.17775	0.403	0.281	0.2045	0.40625	0.2255	0.17025	0.343	0.2365	0.15675	0.283	< 0.001	< 0.001
19	5-Oxoproline	1.97	1.525	2.395	1.91	1.53	2.395	1.5	1.2975	2.0425	1.56	1.35	2.2125	< 0.001	< 0.001
20	Agmatine	0.03995	0.029175	0.05315	0.0424	0.034125	0.054425	0.03645	0.023725	0.042275	0.03595	0.028325	0.04695	< 0.001	< 0.001
21	Pipecolate	0.0725	0.055975	0.115	0.09795	0.067125	0.16675	0.07605	0.054625	0.13375	0.0715	0.05495	0.11825	< 0.001	< 0.001
22	CgA/protein(pmol/mg)	11.85	5.905	49.35	13.65	7.4	30.425	15.55	8.75	46.4	17.75	12.025	54.975	< 0.001	0.001
23	3-Methylhistidine	0.0622	0.04005	0.093425	0.0608	0.048975	0.10775	0.04885	0.038975	0.08235	0.05155	0.041625	0.084225	< 0.001	0.001
24	Creatine	4.28	3.405	6.2425	4.735	4.11	5.8375	3.925	3.2625	4.8775	3.905	2.9825	5.1175	< 0.001	0.001
25	Citrulline	1.625	0.97175	1.9025	1.305	0.8135	1.9175	1.17	0.76525	1.545	1.125	0.781	1.775	< 0.001	0.001
26	Glu-Glu	0.04585	0.00302	0.061725	0.041	0.00302	0.0606	0.00302	0.00302	0.041875	0.00302	0.00302	0.042225	< 0.001	0.001
27	Ile	0.6125	0.287	1.1725	0.552	0.35025	1.015	0.4615	0.3145	0.72375	0.4725	0.322	0.6695	< 0.001	0.001
28	DHEA-S	0.004025	0.0031275	0.0064375	0.004495	0.0029075	0.0066725	0.003725	0.0022325	0.00553	0.00324	0.001935	0.0062175	< 0.001	0.001
29	Ornithine	2.48	1.18	3.74	1.825	1.2125	3.905	1.77	1.0185	2.885	1.82	1.14	2.5225	< 0.001	0.002
30	Lys	3.625	2.2175	6.6975	3.265	2.1425	4.9725	2.835	1.89	4.48	3.035	1.7025	4.1425	0.001	0.002
31	Tyr	4.095	2.905	6.4375	3.715	3.225	6.1525	3.33	2.385	5.37	3.71	2.53	4.115	0.001	0.002
32	Leu	1.585	1.16	3.19	1.785	1.2825	3.1225	1.685	1.0625	2.2125	1.62	0.87975	1.845	0.001	0.003
33	Adenosine	0.05785	0.046875	0.080775	0.072	0.054475	0.0922	0.0559	0.0423	0.07645	0.06395	0.0418	0.078675	0.001	0.004
34	Homovanillate	0.6025	0.4315	0.85125	0.7415	0.38825	0.89825	0.608	0.31125	0.69175	0.66	0.508	0.815	0.001	0.004
35	Cytidine	0.106	0.04095	0.14525	0.114	0.038005	0.17675	0.09965	0.034225	0.14625	0.10535	0.067275	0.16	0.001	0.004
36	Gly-Gly	0.0821	0.0426	0.123	0.0982	0.056175	0.14725	0.077	0.03995	0.10225	0.0773	0.0242	0.10675	0.001	0.004
37	Choline	3.19	2.2025	4.1025	3.135	2.2875	3.81	2.785	2.1875	3.2225	2.595	1.86	3.345	0.001	0.005
38	N^1,N^{12}-Diacetylspermine	0.01815	0.007	0.02625	0.0218	0.016075	0.02795	0.0148	0.00175	0.021475	0.01955	0.01255	0.02555	0.002	0.006
39	Threonate	0.328	0.0506	0.59125	0.4655	0.332	0.75525	0.3385	0.0506	0.52825	0.4545	0.22265	0.61125	0.002	0.007
40	Glucosamine	0.05395	0.00484	0.088775	0.05795	0.0287	0.12	0.00484	0.00484	0.057975	0.036	0.00484	0.062375	0.002	0.007
41	Glycerophosphate	4.735	3.7725	5.5725	5.13	4.285	6.3925	4.24	3.62	5.7875	4.18	3.1675	5.22	0.002	0.007
42	CMP	0.244	0.14925	0.44725	0.2135	0.09985	0.34375	0.19	0.151	0.2715	0.159	0.08589	0.27525	0.002	0.007
43	3-(4-Hydroxyphenyl)propionate	1.82	0.96325	2.435	1.74	1.06125	2.865	1.6	0.92025	2.4325	1.85	0.986	2.9125	0.002	0.007
44	3-Phenyllactate	0.259	0.13425	0.356	0.325	0.1915	0.3695	0.254	0.159	0.32375	0.28	0.1955	0.33925	0.003	0.007
45	Guanosine	0.137	0.0739	0.239	0.156	0.10775	0.21025	0.1265	0.08445	0.1745	0.13	0.091575	0.175	0.003	0.008
46	4-Hydroxymandelate	0.8925	0.59375	1.6525	0.907	0.035	1.6675	0.6315	0.3575	1.3075	0.874	0.62975	1.4425	0.004	0.010
47	Carnitine	0.4485	0.36125	0.635	0.4515	0.36725	0.56675	0.4085	0.311	0.53675	0.3675	0.296	0.53725	0.005	0.013
48	Galacturonate 1-phosphate	0.0738	0.009	0.1365	0.1008	0.009	0.12125	0.009	0.009	0.090575	0.009	0.009	0.1005	0.005	0.014
49	6-Phosphogluconate	0.131	0.099125	0.1555	0.102	0.01274	0.164	0.0917	0.01274	0.15	0.06885	0.01274	0.13725	0.006	0.015
50	2-Hydroxypentanoate	0.505	0.29525	0.70675	0.332	0.2685	0.48325	0.3935	0.26675	0.76225	0.3195	0.2515	0.62025	0.006	0.015
51	Histamine	0.04345	0.027775	0.12925	0.0404	0.032875	0.187	0.04325	0.03365	0.13575	0.0374	0.032075	0.11575	0.006	0.015
52	N^6,N^6,N^6-trimethyllysine	0.0271	0.001286	0.035075	0.03175	0.022625	0.0396	0.01875	0.001286	0.03255	0.027	0.01725	0.03335	0.006	0.015
53	Trp	0.1845	0.1165	0.28275	0.1905	0.14425	0.2435	0.159	0.12575	0.206	0.1735	0.11075	0.19275	0.007	0.016
54	Cortisone	0.00577	0.0042325	0.0087275	0.004675	0.0032925	0.006865	0.004395	0.0029025	0.00687	0.00402	0.00302	0.00655	0.007	0.017
55	Gly-Leu	0.06005	0.037375	0.086575	0.0482	0.035675	0.0817	0.04905	0.026675	0.073975	0.0488	0.02635	0.084375	0.007	0.017
56	*N*-acetyl-*β*-alanine	0.1825	0.0224	0.24475	0.172	0.0224	0.21025	0.0672	0.0224	0.16825	0.139	0.0224	0.20375	0.008	0.018
57	Carnosine	0.0231	0.00197	0.036025	0.0228	0.00197	0.0386	0.01685	0.00197	0.029875	0.01635	0.00197	0.033025	0.012	0.027
58	*N*-*ε*-acetyllysine	0.04875	0.033225	0.0587	0.0582	0.04395	0.08275	0.0437	0.0351	0.059525	0.0455	0.028275	0.0576	0.014	0.031
59	*N*-acetylglutamate	0.061	0.00794	0.084625	0.05495	0.00794	0.079375	0.04645	0.00794	0.067725	0.05165	0.00794	0.0758	0.015	0.031
60	Homoserine	0.0714	0.00352	0.09125	0.06905	0.00352	0.096825	0.05575	0.01408	0.069525	0.04845	0.00352	0.073525	0.015	0.031
61	Cystine	0.00436	0.00436	0.053125	0.03965	0.00436	0.085575	0.00436	0.00436	0.054475	0.03475	0.00436	0.059775	0.019	0.039
62	Thymine	2.265	1.66	3.7225	2.715	1.785	3.54	2.445	1.8525	2.96	2.11	1.57	2.5325	0.019	0.039
63	Asn	0.149	0.00528	0.277	0.162	0.04615	0.302	0.125	0.00528	0.21725	0.0647	0.00528	0.2195	0.020	0.040
64	Ala-Ala	0.1105	0.050065	0.1555	0.1055	0.00856	0.17	0.09135	0.00856	0.135	0.106	0.0542	0.134	0.022	0.043
65	*γ*-Butyrobetaine	0.445	0.30475	0.68325	0.5705	0.43	0.675	0.471	0.308	0.63775	0.4985	0.32	0.7435	0.023	0.044
66	Inosine	0.2085	0.01226	0.371	0.3055	0.25525	0.42025	0.265	0.189	0.358	0.2745	0.16975	0.3575	0.024	0.046
67	2-Hydroxy-4-methylpentanoate	0.2375	0.15275	0.3875	0.183	0.01304	0.27425	0.264	0.1565	0.397	0.281	0.16525	0.346	0.025	0.047
68	7-Methylguanine	0.02895	0.020325	0.0388	0.0358	0.00236	0.044725	0.0246	0.00236	0.029025	0.0258	0.00236	0.034725	0.026	0.048
69	Asp	2.73	2.1425	3.2925	2.515	2.14	3.22	2.455	1.9775	3.1525	2.51	1.82	3.635	0.029	0.053
70	*N*,*N*-Dimethylglycine	0.1105	0.08665	0.14775	0.119	0.09775	0.147	0.1025	0.078	0.14275	0.1055	0.076875	0.1455	0.029	0.053
71	Ser	3.035	2.155	4.0525	2.8	2.1075	3.9175	2.545	1.9025	3.22	2.475	1.81	3.585	0.031	0.056
72	17-OHP	7.79E-05	4.32E-05	0.00011025	5.43E-05	2.31E-05	7.91E-05	4.81E-05	3.21E-05	8.20E-05	4.63E-05	2.80E-05	7.60E-05	0.033	0.058

73	Cytosine	0.04905	0.0232	0.068525	0.06155	0.0409	0.079375	0.0498	0.03395	0.05965	0.0517	0.0439	0.0679	0.039	0.068
74	o-Acetylcarnitine	0.413	0.264	0.55525	0.4645	0.30275	0.612	0.3685	0.301	0.56225	0.326	0.252	0.4855	0.042	0.071
75	Glutathione(ox)	0.05545	0.022495	0.0949	0.03935	0.022025	0.095875	0.05155	0.019525	0.0873	0.0308	0.00358	0.0693	0.061	0.104
76	Met	0.118	0.030355	0.24375	0.191	0.099375	0.24825	0.114	0.0754	0.2055	0.131	0.0401	0.1835	0.063	0.105
77	Gln	4.01	2.705	6.665	3.895	3.54	5.7925	3.575	3.015	4.955	3.87	2.6225	4.8	0.067	0.110
78	Thr	1.09	0.75325	1.57	1.09	0.8745	1.4725	0.9885	0.785	1.265	0.989	0.78725	1.205	0.070	0.112
79	Indole-3-acetate	0.856	0.48725	1.215	0.9045	0.616	1.16	0.7735	0.44075	1.115	0.7265	0.55475	1.15	0.070	0.112
80	*N*-acetylglucosamine 6-phosphate	0.04432	0.01104	0.12025	0.01104	0.01104	0.10475	0.01104	0.01104	0.095875	0.03312	0.01104	0.11225	0.070	0.112
81	Corticosterone	5.19E-05	3.35E-05	8.19E-05	4.85E-05	2.94E-05	6.40E-05	4.19E-05	2.74E-05	5.37E-05	4.20E-05	3.16E-05	4.73E-05	0.079	0.124
82	Spermidine	0.257	0.17875	0.37725	0.2985	0.16375	0.41075	0.243	0.16275	0.371	0.2675	0.20075	0.3465	0.104	0.160
83	Progesterone	0.000148	7.17E-05	0.000172	0.000168	6.31E-05	0.0001875	0.0001405	8.77E-05	0.0001975	0.000113	7.15E-05	0.00015375	0.105	0.160
84	1-Methyladenosine	0.016	0.001642	0.02345	0.0182	0.0082105	0.025925	0.01495	0.001642	0.01875	0.01525	0.010425	0.0188	0.106	0.161
85	Hypotaurine	0.2995	0.169	0.4195	0.342	0.1985	0.46025	0.2735	0.21525	0.35825	0.2495	0.01642	0.35825	0.118	0.176
86	Arg	4.65	3.0825	5.7925	4.665	3.15	6.3475	4.205	2.6125	6.01	3.5	2.5425	6.2625	0.125	0.185
87	N^1,N^8-diacetylspermidine	0.0169	0.01195	0.0249	0.01995	0.0113475	0.024875	0.0151	0.0070975	0.0193	0.0167	0.0092975	0.022325	0.146	0.213
88	SAM+	0.0191	0.0024	0.026925	0.0182	0.0024	0.0332	0.01815	0.0024	0.023025	0.0171	0.0024	0.021725	0.164	0.237
89	Aldosterone	1.44E-05	1.05E-05	2.66E-05	2.34E-05	1.26E-05	3.64E-05	2.16E-05	8.84E-06	3.87E-05	1.58E-05	8.74E-06	3.25E-05	0.178	0.254
90	Adenine	0.203	0.14075	0.2675	0.246	0.168	0.3125	0.2125	0.1495	0.27525	0.2175	0.164	0.29175	0.181	0.255
91	Urocanate	0.2975	0.20875	0.4305	0.3005	0.21325	0.3995	0.2645	0.14775	0.39025	0.283	0.144	0.44575	0.204	0.285
92	Cortisol	0.0015	0.00087	0.0022975	0.00126	0.00076775	0.00189	0.001185	0.000707	0.00233	0.0009825	0.00056125	0.0020275	0.216	0.298
93	Methionine sulfoxide	0.006	0.006	0.0801	0.03835	0.006	0.071375	0.006	0.006	0.066	0.006	0.006	0.061775	0.223	0.305
94	Proline betaine	0.02215	0.00212	0.037275	0.0239	0.01165	0.04535	0.02255	0.00212	0.04175	0.0217	0.00212	0.050425	0.246	0.333
95	*N*-acetylornithine	0.00322	0.00322	0.03815	0.02415	0.00322	0.044425	0.00322	0.00322	0.028975	0.00322	0.00322	0.033025	0.253	0.338
96	Trimethylamine *N*-oxide	0.03335	0.0114435	0.088075	0.0342	0.001674	0.0685	0.03935	0.0168	0.0676	0.0314	0.015775	0.06225	0.274	0.362
97	Testosterone	4.92E-05	9.51E-06	0.00010525	4.35E-05	1.08E-05	8.70E-05	4.98E-05	2.44E-05	8.28E-05	3.59E-05	9.07E-06	7.34E-05	0.303	0.397
98	Nicotinate	0.2305	0.0146	0.36725	0.2195	0.0146	0.29675	0.235	0.0899	0.33225	0.2305	0.14975	0.37575	0.324	0.420
99	18-OH-Corticosterone	0.000183	8.68E-05	0.00053025	0.0002245	0.000103025	0.000465	0.000302	0.0001017	0.00052075	0.00011	6.65E-05	0.00034125	0.337	0.433
100	4-Methyl-2-oxopentanoate	0.37	0.237	0.49025	0.2595	0.21675	0.3835	0.275	0.228	0.428	0.298	0.21	0.3785	0.395	0.501
101	ADMA	0.013	0.001456	0.019925	0.0111	0.001456	0.024925	0.001456	0.001456	0.0199	0.009465	0.001456	0.019225	0.401	0.504
102	Cysteine-glutathione disulfide	0.0603	0.00586	0.11375	0.00586	0.00586	0.071925	0.0456	0.00586	0.101	0.00586	0.00586	0.084775	0.405	0.504
103	AICAR	0.00632	0.00632	0.34025	0.3035	0.00632	0.40625	0.00632	0.00632	0.37675	0.00632	0.00632	0.334	0.418	0.512
104	DHEA	0.0001275	4.95E-05	0.0002125	0.0001715	8.10E-05	0.00022425	7.70E-05	5.29E-05	0.0002035	0.00010485	6.15E-05	0.0001735	0.421	0.512
105	Azelate	0.1315	0.10825	0.153	0.1345	0.10775	0.16075	0.1255	0.1035	0.15375	0.12	0.10475	0.15375	0.423	0.512
106	Lithocholic acid	0.0001175	0.000106	0.00014975	0.0001255	0.0001075	0.00014525	0.0001095	9.89E-05	0.00013875	0.000114	9.98E-05	0.0001385	0.434	0.520
107	*N*-acetylaspartate	0.354	0.20375	0.658	0.409	0.2365	0.76475	0.316	0.22975	0.60375	0.363	0.2005	0.6365	0.442	0.525
108	Glycerate	0.93	0.5895	1.18	0.788	0.65625	0.94575	0.9355	0.765	1.1775	0.781	0.65075	1.015	0.457	0.537
109	11-Deoxycorticosterone	0.00653	0.0050925	0.007905	0.007295	0.006205	0.007935	0.006895	0.0052375	0.0077375	0.006685	0.005405	0.0077225	0.463	0.540
110	17-OH-Pregnenolone	0.0003285	0.00010475	0.00050575	0.000278	0.00017475	0.00048575	0.0003595	0.000152	0.000523	0.000269	0.0001635	0.000484	0.501	0.573
111	25-OHD	0.000113	7.92E-05	0.00031275	0.0001135	6.21E-05	0.00020225	0.000104	7.60E-05	0.0001995	0.0001535	9.27E-05	0.00024025	0.501	0.573
112	Tetrahydrocortisol	0.0027	0.00112	0.00788	0.00441	0.0020175	0.006065	0.003795	0.00153	0.006615	0.00251	0.00122	0.005025	0.515	0.584
113	Pregnenolone	0.000238	0.0001615	0.0003425	0.000255	0.0001695	0.00036125	0.000206	0.000146	0.0003785	0.000215	0.00015425	0.000281	0.534	0.600
114	Betaine	0.835	0.678	1.0125	0.9045	0.638	1.2175	0.825	0.6665	1.2825	0.892	0.622	1.1375	0.560	0.623
115	*N*-acetylglucosamine 1-phosphate	0.108	0.01152	0.15725	0.07415	0.01152	0.1375	0.0868	0.01152	0.14625	0.1128	0.01152	0.1435	0.595	0.657
116	Cholesterol	0.01215	0.00325	0.039225	0.0169	0.0045925	0.040525	0.0253	0.0034075	0.0393	0.005545	0.00267	0.028175	0.607	0.664
117	1,3-Diaminopropane	0.1095	0.058525	0.24325	0.116	0.084225	0.20525	0.1075	0.078475	0.187	0.109	0.083225	0.18	0.624	0.678
118	Estriol	2.64E-05	1.29E-05	6.39E-05	2.84E-05	1.37E-05	6.79E-05	4.87E-05	1.07E-05	0.00010225	2.33E-05	1.29E-05	5.08E-05	0.708	0.763
119	Tetrahydrocortisone	0.0006015	0.00020125	0.001785	0.000856	0.0002545	0.0019	0.000673	0.0002055	0.002255	0.000264	0.00014175	0.0019825	0.726	0.775
120	Androstenediol	0.0005425	0.000287	0.000823	0.0004825	0.00028425	0.00081375	0.0004935	0.00032075	0.0008325	0.000381	0.0002785	0.0006305	0.780	0.826
121	4-Androstene-3,17-dione	4.57E-05	2.97E-05	7.51E-05	6.30E-05	3.45E-05	7.41E-05	4.49E-05	2.70E-05	7.86E-05	6.62E-05	2.34E-05	8.56E-05	0.789	0.828
122	Glycolate	3.085	2.495	3.665	2.98	2.585	3.8225	2.75	2.2575	3.5725	2.715	2.285	3.68	0.822	0.856
123	Estrone	2.66E-06	1.85E-06	4.39E-06	2.55E-06	1.42E-06	4.56E-06	2.55E-06	1.76E-06	4.03E-06	2.58E-06	1.42E-06	4.26E-06	0.852	0.880
124	DHT	9.24E-05	5.16E-05	0.0001775	8.50E-05	5.89E-05	0.00015025	8.17E-05	5.73E-05	0.000162	7.97E-05	5.42E-05	0.0001365	0.905	0.925
125	Biotin	0.0234	0.0234	0.2245	0.0234	0.0234	0.246	0.0847	0.0234	0.233	0.134	0.0234	0.2145	0.911	0.925
126	11-Deoxycortisol	1.22E-05	6.63E-06	2.43E-05	8.24E-06	6.46E-06	2.67E-05	1.15E-05	5.53E-06	2.43E-05	8.55E-06	5.07E-06	1.96E-05	0.938	0.945
127	17β-Estradiol	7.90E-06	4.87E-06	1.19E-05	6.64E-06	4.58E-06	1.14E-05	7.01E-06	5.46E-06	9.23E-06	7.14E-06	4.59E-06	8.34E-06	0.953	0.953

Table S7. Metabolites with significant time-dependent changes (Friedman test followed by post hoc Nemenyi test, $q < 0.05$)

Time point pair	Direction of change	Control group	High-stress group	All participants
Rest → Post-UK	up	sIgA(μg/mL) (1.34), Pipecolate (1.35), Isopropanolamine (1.60), GABA (1.16)		sIgA(μg/mL) (1.39), GABA (1.25), Pipecolate (1.35), Isopropanolamine (1.67)
	down			
Post-UK → Recover	up			2-Hydroxy-4-methylpentanoate (1.44)
	down	Isopropanolamine (0.74), GABA (0.75), 3-Aminoisobutyrate (0.86), *N*-acetylputrescine (0.80), α-Aminoadipate (0.72), Sarcosine (0.77), Creatinine (0.90), 3-Methylhistidine (0.82), Glu (0.79), His (0.76), Anserine (0.52)	GABA (0.66), Sarcosine (0.67), Cytidine (0.92), His (0.85), Creatinine (0.82), Hydroxyproline (0.68), beta-Ala (0.69), 3-Aminoisobutyrate (0.81), Gly-Gly (0.74), Agmatine (0.78), α-Aminoadipate (0.73), 2AB (0.69), Glu (0.73), Val (0.79), *N*-acetylputrescine (0.67), Phe (0.68), Carnitine (0.77), 3-Methylhistidine (0.76), Hypoxanthine (0.77), 5-Oxoproline (0.70), Guanosine (0.80), Isopropanolamine (0.58), Homovanillate (0.80)	sIgA(μg/mL) (0.76), GABA (0.71), Pipecolate (0.78), Isopropanolamine (0.64), 3-Aminoisobutyrate (0.79), *N*-acetylputrescine (0.75), Sarcosine (0.69), β-Ala (0.80), Threonate (0.73), Cytidine (0.87), Glycerophosphate (0.83), Hydroxyproline (0.80), N^6,N^6,N^6-trimethyllysine (0.59), α-Aminoadipate (0.73), Adenosine (0.78), Creatinine (0.80), 3-(4-Hydroxyphenyl)propionate (0.92), Guanosine (0.81), Creatine (0.83), 2AB (0.80), N^1,N^{12}-diacetylspermine (0.68), Anserine (0.50), Phe (0.74), 3-Phenyllactate (0.78), Ile (0.84), Agmatine (0.86), Gly-Gly (0.78), 3-Methylhistidine (0.80), DHEA-S (0.83), Ala-Ala (0.87), Homovanillate (0.82), 5-Oxoproline (0.79), Glu (0.79), His (0.89), Lys (0.87), Val (0.84), Hypoxanthine (0.73), Tyr (0.90), Leu (0.94), Carnitine (0.90), Trp (0.83), Glucosamine (0.08)
Recover → Post-WM	up			
	down			
Rest → Post-WM	up			CgA/protein(pmol/mg) (1.50)
	down	PEP (0.67)		Creatinine (0.93), Citrulline (0.69), PEP (0.61), Glu (0.76), His (0.73)
Rest → Recover	up			
	down			Sarcosine (0.88), α-Aminoadipate (0.76), Citrulline (0.72), Ornithine (0.71), 5-Oxoproline (0.76), PEP (0.69), Glu (0.80), His (0.85), Lys (0.78), Hypoxanthine (0.82), 4-Hydroxymandelate (0.71)
Post-UK → Post-WM	up			CgA/protein(pmol/mg) (1.30)
	down	3-Aminoisobutyrate (0.89), *N*-acetylputrescine (0.82), α-Aminoadipate (0.76), Creatinine (0.88), Hypoxanthine (0.79)	GABA (0.64), Sarcosine (0.71), Pipecolate (0.68), Choline (0.72), His (0.78), Creatinine (0.85), Hydroxyproline (0.79), PEP (0.59), β-Ala (0.67), 3-Aminoisobutyrate (0.72), Gly-Gly (0.61), Agmatine (0.74), α-Aminoadipate (0.68), 2AB (0.67), Glu (0.67), Tyr (0.94), Val (0.85), Galacturonate 1-phosphate (0.09), *N*-acetylputrescine (0.66), Phe (0.66), Creatine (0.75), 3-Methylhistidine (0.88), Ile (0.98), N^6,N^6,N^6-Trimethyllysine (0.65), Guanosine (0.89), Anserine (0.57), Isopropanolamine (0.65), Histamine (0.60)	sIgA(μg/mL) (0.82), GABA (0.70), Pipecolate (0.73), Isopropanolamine (0.68), 3-Aminoisobutyrate (0.90), *N*-Acetylputrescine (0.79), Sarcosine (0.73), β-Ala (0.71), Histamine (0.93), Glycerophosphate (0.81), Inosine (0.90), Hydroxyproline (0.82), N^6,N^6,N^6-trimethyllysine (0.85), α-Aminoadipate (0.73), Adenosine (0.89), Creatinine (0.83), Guanosine (0.83), Creatine (0.82), 2AB (0.84), Anserine (0.84), Phe (0.76), Ile (0.86), Agmatine (0.85), Gly-Gly (0.79), DHEA-S (0.72), PEP (0.67), Glu (0.75), His (0.76), Val (0.86), Choline (0.83), Hypoxanthine (0.69), Tyr (1.00), Leu (0.91), Thymine (0.78)

The table lists all metabolites that showed a statistically significant difference between specific time points (post-hoc Nemenyi test, $q < 0.05$).
Fold-change values, shown in parentheses, represent the ratio of metabolite levels between the two indicated time points.
"Up" and "down" indicate an increase or decrease, respectively, in metabolite levels at the second time point compared to the first.

Table S8. Metabolites with significant time-dependent changes and fold changes <0.70 or >1.30 (Friedman test with post hoc Nemenyi test, $q < 0.05$)

**Time point comparison**	**Direction of change**	**Control group**	**High-stress group**	**All participants**
Rest → Post-UK	up	sIgA(μg/mL) (1.34), Pipecolate (1.35), Isopropanolamine (1.60)		sIgA(μg/mL) (1.39), Pipecolate (1.35), Isopropanolamine (1.67)
	down			
Post-UK → Recover	up			2-Hydroxy-4-methylpentanoate (1.44)
	down	Anserine (0.52)	GABA (0.66), Sarcosine (0.67), Hydroxyproline (0.68), β-Ala (0.69), 2AB (0.69), N-Acetylputrescine (0.67), Phe (0.68), 5-Oxoproline (0.70), Isopropanolamine (0.58)	Isopropanolamine (0.64), Sarcosine (0.69), N^6,N^6,N^6-trimethyllysine (0.59), N^1,N^{12}-diacetylspermine (0.68), Anserine (0.50), Glucosamine (0.08)
Recover → Post-WM	up			
	down			
Rest → Post-WM	up			CgA/protein(pmol/mg) (1.50)
	down	PEP (0.67)		Citrulline (0.69), PEP (0.61)
Rest → Recover	up			
	down			PEP (0.69)
Post-UK → Post-WM	up			CgA/protein(pmol/mg) (1.30)
	down		GABA (0.64), Pipecolate (0.68), PEP (0.59), β-Ala (0.67), Gly-Gly (0.61), α-Aminoadipate (0.68), 2AB (0.67), Glu (0.67), Galacturonate 1-phosphate (0.09), N-acetylputrescine (0.66), Phe (0.66), N^6,N^6,N^6-trimethyllysine (0.65), Anserine (0.57), Isopropanolamine (0.65), Histamine (0.60)	GABA (0.70), Isopropanolamine (0.68), PEP (0.67), Hypoxanthine (0.69)

Significant time-dependent changes were first identified using the Friedman test. The table lists only those metabolites where the post-hoc Nemenyi test showed a q-value < 0.05 and the fold change was ≤ 0.70 or ≥ 1.30.

[illegible]

Table S9. Predictive performance of known stress markers

Time Point	Metabolite	AUC [95% CI]	Accuracy [95% CI]	F1 Score [95% CI]	Association with High Stress
Rest	sIgA	0.570 [0.415 – 0.746]	0.528 [0.234 – 0.766]	0.488 [0.00 – 0.785]	↑
	CgA	0.584 [0.392 – 0.775]	0.600 [0.296 – 0.813]	0.637 [0.363 – 0.821]	↓
	Cortisol	0.580 [0.444 – 0.721]	0.576 [0.234 – 0.750]	0.572 [0.00 – 0.811]	↑
	DHEA	0.518 [0.327 – 0.785]	0.571 [0.155 – 0.781]	0.626 [0.174 – 0.831]	↓
	DHEA-S	0.626 [0.437 – 0.780]	0.621 [0.234 – 0.781]	0.587 [0.0633 – 0.825]	↓
Post-UK	sIgA	0.545 [0.353 – 0.748]	0.552 [0.219 – 0.798]	0.624 [0.252 – 0.841]	↓
	CgA	0.595 [0.456 – 0.738]	0.626 [0.342 – 0.798]	0.653 [0.342 – 0.848]	↓
	Cortisol	0.543 [0.405 – 0.704]	0.581 [0.234 – 0.798]	0.450 [0.00 – 0.806]	↑
	DHEA	0.567 [0.312 – 0.819]	0.550 [0.202 – 0.813]	0.426 [0.00 – 0.804]	↑
	DHEA-S	0.652 [0.483 – 0.815]	0.650 [0.313 – 0.860]	0.607 [0.00 – 0.858]	↓
Recover	sIgA	0.552 [0.341 – 0.748]	0.568 [0.248 – 0.781]	0.579 [0.0452 – 0.800]	↓
	CgA	0.587 [0.388 – 0.797]	0.561 [0.296 – 0.781]	0.585 [0.189 – 0.831]	↓
	Cortisol	0.575 [0.472 – 0.726]	0.534 [0.188 – 0.766]	0.503 [0.00 – 0.785]	↑
	DHEA	0.605 [0.433 – 0.774]	0.597 [0.265 – 0.766]	0.571 [0.167 – 0.789]	↑
	DHEA-S	0.693 [0.363 – 0.889]	0.691 [0.452 – 0.875]	0.658 [0.0864 – 0.880]	↓
Post-WM	sIgA	0.559 [0.404 – 0.767]	0.558 [0.281 – 0.813]	0.605 [0.167 – 0.838]	↓
	CgA	0.553 [0.352 – 0.751]	0.550 [0.140 – 0.766]	0.604 [0.143 – 0.805]	↓
	Cortisol	0.625 [0.456 – 0.819]	0.623 [0.296 – 0.844]	0.542 [0.00 – 0.838]	↓
	DHEA	0.740 [0.540 – 0.868]	0.692 [0.469 – 0.844]	0.698 [0.448 – 0.875]	↓
	DHEA-S	0.668 [0.469 – 0.820]	0.618 [0.313 – 0.813]	0.557 [0.00 – 0.835]	↓
All	sIgA	0.552 [0.405 – 0.713]	0.469 [0.218 – 0.704]	0.527 [0.120 – 0.767]	↓
	CgA	0.578 [0.434 – 0.715]	0.459 [0.144 – 0.645]	0.503 [0.142 – 0.727]	↓
	Cortisol	0.552 [0.467 – 0.675]	0.450 [0.191 – 0.672]	0.326 [0.00 – 0.656]	↑
	DHEA	0.556 [0.410 – 0.673]	0.491 [0.281 – 0.664]	0.525 [0.198 – 0.765]	↓
	DHEA-S	0.652 [0.435 – 0.812]	0.551 [0.269 – 0.758]	0.499 [0.00 – 0.774]	↓

The predictive performance of established stress markers was evaluated using a single-variable threshold model, with the classification threshold determined by the Youden index.

Association with High Stress: ↑ indicates that higher values are associated with the high-stress group; ↓ indicates that lower values are associated with the high-stress group.

UK, Uchida-Kraepelin test; WM, working memory test; AUC, area under the receiver operating characteristic curve; CI, confidence interval.

CgA, chromogranin A; DHEA, dehydroepiandrosterone; DHEA-S, dehydroepiandrosterone sulfate; sIgA, secretory immunoglobulin A.

Table S10. Full performance results for metabolite ratio models

	Time Point	Ratio	AUC_mean	AUC_ci_lo	AUC_ci_up	ACC_mean	ACC_ci_lo	ACC_ci_up	F1_mean	F1_ci_lo	F1_ci_up	Recall_mean	Recall_ci_lo	Recall_ci_up	Precision_mean	Precision_ci_lo	Precision_ci_up	Association with High Stress
1	Rest	*N*-acetyl-*β*-alanine / ADMA	0.845	0.669	0.971	0.815	0.640	0.938	0.833	0.667	0.952	0.921	0.733	1.000	0.766	0.588	0.937	↓
2	Rest	*N*-acetyl-*β*-alanine / 7-Methylguanine	0.829	0.678	0.955	0.777	0.576	0.906	0.785	0.558	0.917	0.820	0.588	1.000	0.767	0.513	0.943	↓
3	Rest	Ile / 7-Methylguanine	0.793	0.648	0.936	0.768	0.577	0.906	0.770	0.586	0.929	0.789	0.601	1.000	0.764	0.544	0.943	↓
4	Rest	Glycerate / 6-Phosphogluconate	0.787	0.607	0.907	0.744	0.546	0.891	0.749	0.544	0.912	0.780	0.418	1.000	0.747	0.511	0.970	↓
5	Rest	Val / ADMA	0.783	0.628	0.946	0.759	0.563	0.923	0.743	0.527	0.925	0.703	0.460	0.941	0.804	0.539	1.000	↓
6	Rest	7-Methylguanine / Val	0.771	0.623	0.935	0.741	0.563	0.938	0.757	0.519	0.951	0.828	0.469	1.000	0.710	0.458	0.936	↑
7	Rest	His / Glu	0.760	0.614	0.896	0.729	0.531	0.875	0.707	0.531	0.889	0.659	0.447	0.946	0.796	0.525	1.000	↑
1	Post-UK	Progesterone / 25-OHD	0.835	0.630	0.968	0.825	0.625	0.954	0.817	0.604	0.956	0.782	0.594	0.977	0.866	0.627	1.000	↓
2	Post-UK	Thr / 25-OHD	0.833	0.639	0.986	0.801	0.594	0.954	0.796	0.586	0.959	0.798	0.529	1.000	0.804	0.575	1.000	↓
3	Post-UK	25-OHD / Inosine	0.833	0.628	0.982	0.778	0.546	0.954	0.777	0.533	0.960	0.779	0.500	1.000	0.787	0.533	1.000	↑
4	Post-UK	25-OHD / Adenine	0.827	0.624	0.975	0.781	0.531	0.938	0.775	0.522	0.946	0.760	0.474	1.000	0.805	0.510	1.000	↑
5	Post-UK	Hypoxanthine / 25-OHD	0.827	0.648	0.962	0.826	0.688	0.969	0.823	0.664	0.966	0.812	0.646	0.977	0.843	0.615	1.000	↓
6	Post-UK	Gln / 25-OHD	0.826	0.647	0.949	0.776	0.577	0.906	0.767	0.505	0.917	0.736	0.483	0.929	0.816	0.483	1.000	↓
7	Post-UK	25-OHD / Adenosine	0.815	0.622	0.950	0.763	0.531	0.906	0.754	0.530	0.907	0.733	0.488	0.946	0.791	0.500	1.000	↑
8	Post-UK	1,3-Diaminopropane / 25-OHD	0.807	0.646	0.958	0.746	0.513	0.923	0.757	0.516	0.932	0.808	0.471	1.000	0.738	0.486	1.000	↓
9	Post-UK	25-OHD / beta-Ala	0.802	0.616	0.963	0.756	0.563	0.923	0.759	0.579	0.923	0.773	0.524	0.946	0.760	0.534	0.947	↑
10	Post-UK	11-Deoxycorticosterone / 25-OHD	0.801	0.605	0.957	0.755	0.546	0.891	0.770	0.563	0.911	0.821	0.559	1.000	0.738	0.500	0.966	↓
11	Post-UK	Tetrahydrocortisol / Homovanillate	0.801	0.634	0.939	0.738	0.546	0.875	0.758	0.584	0.898	0.827	0.589	1.000	0.714	0.500	0.889	↓
12	Post-UK	Glu / 25-OHD	0.800	0.631	0.935	0.780	0.563	0.923	0.769	0.509	0.929	0.743	0.451	0.944	0.814	0.563	1.000	↓
13	Post-UK	Leu / 25-OHD	0.794	0.609	0.945	0.744	0.515	0.891	0.752	0.593	0.916	0.778	0.514	1.000	0.743	0.499	1.000	↓
14	Post-UK	Tetrahydrocortisol / Lithocholic acid	0.785	0.651	0.940	0.746	0.594	0.906	0.754	0.503	0.925	0.788	0.534	1.000	0.740	0.500	0.976	↓
1	Recover	Ser / His	0.806	0.646	0.962	0.801	0.640	0.938	0.792	0.613	0.949	0.766	0.586	0.949	0.831	0.594	1.000	↓
2	Recover	Glu / His	0.805	0.652	0.945	0.821	0.640	0.938	0.829	0.636	0.943	0.884	0.588	1.000	0.792	0.605	1.000	↓
3	Recover	Tyr / Val	0.794	0.627	0.925	0.748	0.531	0.906	0.756	0.517	0.918	0.801	0.471	1.000	0.738	0.485	1.000	↑
4	Recover	His / Estrone	0.786	0.628	0.912	0.756	0.563	0.906	0.749	0.542	0.921	0.732	0.485	0.943	0.782	0.531	1.000	↑
5	Recover	His / *N*-acetylglucosamine 6-phosphate	0.779	0.625	0.920	0.752	0.563	0.891	0.723	0.533	0.900	0.652	0.411	0.909	0.836	0.516	1.000	↑
6	Recover	Tyr / Estrone	0.777	0.611	0.919	0.777	0.594	0.923	0.777	0.547	0.922	0.797	0.460	1.000	0.775	0.600	0.946	↑
7	Recover	Tyr / Ser	0.774	0.603	0.907	0.774	0.577	0.906	0.747	0.535	0.907	0.669	0.439	0.917	0.861	0.667	1.000	↑
8	Recover	Trp / AICAR	0.769	0.611	0.912	0.750	0.594	0.906	0.751	0.540	0.916	0.761	0.500	1.000	0.756	0.556	0.943	↓
9	Recover	Glycerate / *N*-acetylglucosamine 6-phosphate	0.762	0.613	0.913	0.792	0.671	0.906	0.775	0.618	0.918	0.725	0.542	0.907	0.845	0.639	1.000	↑
1	Post-WM	4-Methyl-2-oxopentanoate / N^6,N^6,N^6-trimethyllysine	0.867	0.735	0.981	0.807	0.656	0.938	0.786	0.582	0.939	0.719	0.485	0.918	0.883	0.623	1.000	↑
2	Post-WM	Ile / *N*-*ε*-acetyllysine	0.849	0.727	0.952	0.788	0.577	0.938	0.785	0.538	0.947	0.791	0.529	1.000	0.791	0.518	0.949	↓
3	Post-WM	Val / *N*-*ε*-acetyllysine	0.834	0.685	0.968	0.821	0.688	0.938	0.822	0.677	0.956	0.836	0.656	1.000	0.817	0.613	1.000	↓
4	Post-WM	4-Methyl-2-oxopentanoate / Val	0.834	0.686	0.950	0.764	0.531	0.906	0.762	0.508	0.932	0.776	0.440	1.000	0.775	0.485	1.000	↑
5	Post-WM	Agmatine / N^6,N^6,N^6-trimethyllysine	0.831	0.705	0.941	0.751	0.563	0.906	0.747	0.539	0.909	0.744	0.491	1.000	0.774	0.513	1.000	↑
6	Post-WM	His / N^6,N^6,N^6-trimethyllysine	0.828	0.700	0.946	0.771	0.515	0.906	0.758	0.524	0.925	0.736	0.462	1.000	0.805	0.536	1.000	↑
7	Post-WM	Leu / *N*-*ε*-acetyllysine	0.820	0.686	0.961	0.815	0.625	0.938	0.818	0.583	0.951	0.853	0.583	1.000	0.796	0.577	0.976	↓
8	Post-WM	Cortisol / *N*-acetylglucosamine 1-phosphate	0.807	0.655	0.918	0.767	0.546	0.906	0.762	0.516	0.909	0.757	0.486	1.000	0.782	0.504	1.000	↓
9	Post-WM	2AB / *N*-*ε*-acetyllysine	0.805	0.656	0.959	0.832	0.688	0.938	0.829	0.664	0.949	0.820	0.656	0.976	0.844	0.613	1.000	↓
1	All	4-Methyl-2-oxopentanoate / Choline	0.724	0.563	0.850	0.684	0.523	0.789	0.703	0.501	0.821	0.752	0.548	0.919	0.667	0.432	0.827	↑
2	All	Choline / Azelate	0.653	0.514	0.773	0.671	0.550	0.789	0.720	0.573	0.834	0.843	0.693	0.931	0.633	0.454	0.788	↓

The performance of predictive models constructed using the ratio of two metabolites as a single feature is shown. The classification threshold was determined by the Youden index.

Models were considered top performers if the lower bound of the 95% confidence interval for their performance metrics exceeded the following thresholds: AUC > 0.60, Accuracy > 0.50, and F1 Score > 0.50 (for Post-WM: AUC > 0.65; for All time points: AUC > 0.50).

Association with High Stress: ↑ indicates that higher values are associated with the high-stress group; ↓ indicates that lower values are associated with the high-stress group.

UK, Uchida–Kraepelin test; WM, working memory test; AUC, area under the receiver operating characteristic curve; CI, confidence interval.

25-OHD, 25-hydroxyvitamin D; ADMA, asymmetric dimethylarginine; Gln, glutamine; Glu, glutamic acid; His, histidine; Ile, isoleucine; Leu, leucine; Ser, serine; Thr, threonine; Trp, tryptophan; Tyr, tyrosine; Val, valine.

Table S11. Assessment of confounding factors on the predictive model

Generalized Linear Model Regression Results			
**Dep. Variable:**	GROUP	**No. Observations:**	32
**Model:**	GLM	**Df Residuals:**	27
**Model Family:**	Binomial	**Df Model:**	4
**Link Function:**	Logit	**Scale:**	1
**Method:**	IRLS	**Log-Likelihood:**	-12.419
**Date:**	Tue, 17 Jun 2025	**Deviance:**	24.838
**Time:**	14:44:11	**Pearson chi2:**	27.8
**No. Iterations:**	8	**Pseudo R-squ. (CS):**	0.4567
**Covariance Type:**	nonrobust		

	**coef**	**std err**	**z**	**P>|z|**	**[0.025**	**0.975]**
**Intercept**	-5.554	3.034	-1.831	0.067	-11.5	0.392
**AGEID_30s[T.True]**	0.9182	1.517	0.605	0.545	-2.055	3.892
**AGEID_40s[T.True]**	2.6029	1.715	1.517	0.129	-0.759	5.965
**Q('*N*-Acetyl-beta-alanine/ADMA')**	-9.2101	4.527	-2.034	0.042	-18.084	-0.337
**SEX**	0.7297	1.188	0.614	0.539	-1.599	3.059

A binomial GLM with a logit link function was used to model group classification (0 = control group, 1 = high-stress group) based on sex, age category, and *N*-Acetyl-beta-alanine/ADMA. Sex was coded as 0 = male and 1 = female. Age category was encoded using dummy variables: AGEID_30s and AGEID_40s, where individuals with AGEID_30s = 0 and AGEID_40s = 0 were considered to be in their 20s.

The model formula was: GROUP ~ SEX + AGEID_30s + AGEID_40s + (*N*-Acetyl-beta-alanine/ADMA)

Estimation was conducted using IRLS in the statsmodels Python package (v0.14.0).

ADMA, asymmetric dimethylarginine

Table S12. Concentrations of metabolites in mouth-rinsed water for each participant (μM)

Participant ID	1	2	3	4	5	6	7	8	9	10	11	12	13	14	15	16	17	18	19	20	21	22	23	24	25	26	27	28	29	30	31	32
GROUP	Control	High-stress	Control	High-stress	High-stress	Control	High-stress	Control	High-stress	Control	Control	High-stress	Control	Control	Control	High-stress	Control	Control	High-stress	Control	High-stress	High-stress	Control	Control	High-stress	Control	Control	High-stress	High-stress	High-stress	High-stress	High-stress
GENDER	Female	Female	Female	Female	Male	Male	Female	Female	Female	Male	Male	Female	Female	Male	Male	Female	Male	Female	Male	Female	Male	Male	Female	Male	Male	Female	Female	Female	Male	Male	Female	Female
AGE	27	33	48	25	44	34	29	42	34	30	31	47	39	36	45	31	45	26	39	34	47	37	39	46	35	46	28	41	32	43	40	28
sIgA(μg/mL)_Rest	35.8	94.6	37	34.7	66.6	47.7	42.6	25.2	36.8	116	48.9	41.1	36.2	43.2	55.3	36.1	56.5	37.5	89.4	49.9	43.8	57	52.8	38.8	50.3	19.6	31.7	29.1	48.3	43.7	46.6	22.5
sIgA(μg/mL)_Post-UK	58.9	71.4	41	67.2	104	51	52.2	36.1	69.9	219	114	78.2	70.2	82.4	113	66.6	49.1	47	130	68.4	58.9	43.4	73.9	44.6	80.2	28.4	37.5	30	53.4	62.1	49.8	36.7
sIgA(μg/mL)_Recover	43.5	64	43	64	70	53.6	40.1	35	77.5	208	85	63.5	61.5	68.6	97.4	76	45.3	34.4	89.2	43	47.2	36	43.5	36.3	50.6	19.7	40.2	25	60.6	43.3	35.4	27
sIgA(μg/mL)_Post-WM	42.2	77.7	31.5	43.5	48.2	54.1	28.2	28	55.9	136	121	54.3	61.8	76.5	71.5	80.6	64.2	33.6	73.3	40.7	41.2	39.4	51.4	41	58.3	28.2	45.5	38.4	59.5	58.7	29	46.6
CgA/protein(pmol/mg)_Rest	143	71.2	3.47	102	5.27	1.59	5.12	5.99	7.87	67.7	49.1	123	30.9	24.7	50.1	12.2	164	23.6	4.56	11.5	7.27	6.76	1.78	6.27	9.47	2.02	64.9	5.65	6.67	19.8	29.6	30.5
CgA/protein(pmol/mg)_Post-UK	131	89.7	5.79	70.8	4.3	5.33	10.3	4.37	9.62	32.3	36.7	127	15.8	14.9	28.7	6.92	22.7	22.2	7.56	12.4	15.8	8.06	4.17	4.47	11	4.64	112	31.1	11.9	11.7	30.2	22.3
CgA/protein(pmol/mg)_Recover	180	99.3	34.2	143	25.8	4.33	8.96	8.51	8.46	29.8	48.6	79	15.6	11.7	13.5	8.83	47.9	24	4.44	8.35	15.1	10.8	1.89	5.38	15.5	7.78	87.4	45.9	13.8	29.5	61	36.4
CgA/protein(pmol/mg)_Post-WM	204	95.9	11.1	173	9.55	9	31.1	12.1	14.5	54.4	99.6	55.8	17.9	16.4	50.8	9.23	91.4	17	5.03	13.2	17.6	11.8	2	6.32	12.8	12.6	172	54.7	22.6	28.3	59.7	27.4
Tetrahydrocortisol_Rest	0.00806	0.00989	0.00241	0.000173	0.00256	0.000722	0.00782	0.00998	0.00473	0.00312	0.00119	0.0105	0.0107	0.000377	0.000602	0.00431	0.0129	0.0117	0.00245	0.00204	0.00768	0.00114	0.00279	0.000859	0.00945	0.00191	0.00422	0.000268	0.00106	0.00614	0.00261	0.000108
Tetrahydrocortisol_Post-UK	0.00374	0.00527	0.00263	0.00601	0.00469	0.00623	0.000266	0.00476	0.00378	0.0124	0.00712	0.00753	0.0113	0.00146	0.0119	0.00489	0.00436	0.0132	0.00367	0.00103	0.000583	0.00164	0.00487	0.00762	0.00501	0.00293	0.00416	0.000568	0.00208	0.00183	0.00446	0.000114
Tetrahydrocortisol_Recover	0.00257	0.00718	0.000236	0.00182	0.00254	0.0016	0.000807	0.0218	0.00607	0.00951	0.00696	0.0103	0.00105	0.00422	0.000927	0.00207	0.00608	0.0033	0.0125	0.0104	0.00387	0.000595	0.0065	0.000807	0.00601	0.00973	0.00372	0.00132	0.000173	0.00516	0.00278	0.0057
Tetrahydrocortisol_Post-WM	0.0026	0.0051	0.00929	0.00242	0.00491	0.0033	0.000421	0.00427	0.000727	0.005	0.00209	0.001	0.00165	0.000943	0.00116	0.00225	0.00308	0.00171	0.0032	0.00625	0.00124	0.00662	0.00475	0.00516	0.000797	0.000868	0.0122	0.00198	0.0011	0.00232	0.00965	0.00744
Cortisol_Rest	0.00144	0.00225	0.000713	0.0015	0.00461	0.00482	0.00286	0.00201	0.00147	0.00146	0.00238	0.0015	0.000704	0.00087	0.000644	0.0018	0.00436	0.00212	0.00106	0.000665	0.00245	0.000408	0.000958	0.00289	0.00357	0.00125	0.0016	0.00226	0.000791	0.00087	0.000531	0.00227
Cortisol_Post-UK	0.00158	0.00273	0.000533	0.000797	0.00565	0.00176	0.00468	0.000632	0.000755	0.00363	0.00264	0.00127	0.000772	0.00187	0.000835	0.00134	0.00195	0.00215	0.00115	0.000625	0.00501	0.000361	0.00125	0.00138	0.00162	0.00132	0.000713	0.000496	0.00096	0.000646	0.000847	0.0009
Cortisol_Recover	0.000914	0.00339	0.000294	0.00068	0.00334	0.00281	0.00199	0.00131	0.00108	0.00446	0.00216	0.00374	0.000347	0.0016	0.00039	0.000613	0.00245	0.000807	0.00148	0.000935	0.00123	0.000212	0.00136	0.00275	0.00229	0.00102	0.000716	0.000368	0.00256	0.00114	0.0004	0.00109
Cortisol_Post-WM	0.000447	0.00247	0.00125	0.000407	0.00149	0.00123	0.000895	0.000693	0.000619	0.00171	0.00354	0.00456	0.000303	0.00368	0.000479	0.000647	0.00926	0.00121	0.00107	0.00197	0.00211	0.000414	0.00237	0.000681	0.00448	0.000761	0.002	0.000422	0.000584	0.000407	0.000493	0.000669
18-OH-Corticosterone_Rest	0.000287	0.00045	7.97E-05	1.53E-05	0.000911	3.33E-05	0.000646	0.00102	0.000359	8.92E-05	0.000466	0.000217	0.000116	3.51E-05	0.00014	0.00109	0.00227	0.000603	0.000149	0.000132	0.000631	5.05E-05	0.000123	2.43E-05	0.00135	0.000348	0.000506	2.63E-05	0.000105	0.000333	0.000138	1.70E-05
18-OH-Corticosterone_Post-UK	0.000573	0.000501	8.97E-05	0.000318	0.000935	0.0005	2.78E-05	7.70E-05	0.000142	0.000681	0.000637	0.000159	0.000234	0.000489	0.000162	0.000457	0.000348	0.000589	0.000215	0.00013	3.56E-05	6.00E-05	0.000401	0.000408	0.000154	0.000286	0.000107	5.22E-05	9.11E-05	0.000161	0.000392	8.72E-06
18-OH-Corticosterone_Recover	0.000343	0.00106	2.27E-05	0.000147	0.000619	3.65E-05	0.000499	0.000365	0.000283	0.00104	0.000399	0.0011	5.03E-05	0.000506	5.58E-05	0.000117	0.00163	0.000129	0.000572	0.000321	0.000206	1.63E-05	0.000356	2.20E-05	0.00057	0.000218	0.000134	5.41E-05	9.76E-06	0.000565	0.00012	0.000379
18-OH-Corticosterone_Post-WM	7.74E-05	0.000487	0.000477	7.08E-05	0.000296	0.000151	8.71E-05	8.66E-05	5.65E-05	0.000604	0.00111	3.54E-05	5.02E-05	2.05E-05	3.51E-05	8.12E-05	0.000109	0.000491	0.000184	0.000603	0.000701	0.000167	1.37E-05	0.000132	2.20E-05	0.000183	0.000795	6.43E-05	7.01E-05	6.73E-05	0.000203	0.000111
Tetrahydrocortisone_Rest	0.0017	0.00212	0.000421	4.48E-05	0.00216	0.000123	0.000725	0.00427	0.00118	0.000249	0.00212	0.000327	0.00153	5.47E-05	0.00111	0.00489	0.00241	0.00164	0.000354	0.000108	0.00183	0.000137	0.000235	7.81E-05	0.00177	0.00256	0.000478	0.000142	0.000221	0.00117	0.000424	1.98E-05
Tetrahydrocortisone_Post-UK	0.00216	0.00298	0.000329	0.00145	0.00116	0.00239	3.00E-05	0.000282	0.000323	0.00206	0.00308	0.000202	0.00132	0.00156	0.00219	0.00178	0.000262	0.00207	0.000552	0.000172	7.78E-05	0.000117	0.0019	0.00183	0.000347	0.0019	0.000118	0.000232	0.000297	0.000264	0.00166	4.76E-06
Tetrahydrocortisone_Recover	0.00288	0.00198	7.97E-05	0.00168	0.000633	0.000148	0.00119	0.000487	0.0013	0.00687	0.00274	0.000929	0.000197	0.000478	0.000229	0.000207	0.00227	0.000297	0.00225	0.000713	0.000541	4.86E-05	0.0024	7.15E-05	0.00247	0.00166	0.000111	0.000234	6.33E-05	0.00342	0.000201	0.0035
Tetrahydrocortisone_Post-WM	0.000213	0.00205	0.00783	0.000192	0.000116	0.000324	8.37E-05	0.000269	0.00025	0.00244	0.00567	3.52E-05	0.000171	4.42E-05	0.000129	0.000103	4.69E-05	0.000702	0.000504	0.00246	0.00189	0.00214	2.36E-05	0.000279	0.000147	0.00196	0.00206	0.000259	0.000223	0.000146	0.00212	0.00109
Cortisone_Rest	0.00571	0.00415	0.00401	0.00749	0.0107	0.0197	0.00724	0.00564	0.00664	0.00871	0.00583	0.0113	0.000985	0.00794	0.00233	0.00518	0.0106	0.00793	0.00568	0.00426	0.00898	0.00193	0.00455	0.0145	0.00878	0.00298	0.0057	0.0175	0.00495	0.00415	0.00286	0.00586
Cortisone_Post-UK	0.00464	0.00407	0.00312	0.00292	0.0104	0.00471	0.0136	0.00335	0.00576	0.0115	0.00716	0.00855	0.00145	0.00438	0.00224	0.00432	0.00679	0.00812	0.00709	0.00381	0.023	0.00145	0.00384	0.00642	0.00674	0.00403	0.00298	0.00298	0.00475	0.00492	0.00237	0.00552
Cortisone_Recover	0.003	0.00418	0.00246	0.00203	0.00768	0.0231	0.00461	0.00481	0.00577	0.0124	0.00606	0.0105	0.00121	0.0057	0.00282	0.00297	0.00728	0.00488	0.00681	0.00411	0.00705	0.00101	0.0039	0.0126	0.00506	0.00293	0.00332	0.00249	0.0107	0.00402	0.00213	0.00173
Cortisone_Post-WM	0.00321	0.00387	0.0035	0.00285	0.00484	0.0053	0.00349	0.00364	0.00469	0.00826	0.00975	0.0226	0.00119	0.0186	0.00261	0.00353	0.0275	0.00529	0.00643	0.00426	0.00691	0.00166	0.00888	0.0047	0.0192	0.00281	0.00344	0.00305	0.00417	0.00293	0.00119	0.00203
17-OH-Pregnenolone_Rest	0.00057	0.000235	0.00026	0.000166	0.000851	7.13E-06	0.000358	0.000905	8.14E-05	0.000462	0.000102	0.000324	0.000379	0.000119	0.000333	0.000971	0.000624	0.000243	0.000485	0.000356	0.000105	0.000139	7.91E-05	0.000104	0.000567	0.000489	0.000444	4.47E-05	0.000556	0.000715	9.98E-05	4.52E-06
17-OH-Pregnenolone_Post-UK	0.00117	0.000265	0.000187	0.000231	0.000871	0.000297	5.68E-06	0.000126	0.000158	0.000148	0.000178	0.000417	0.0006	0.000197	0.000259	0.000619	0.0009	0.000291	0.000327	0.000165	7.13E-05	0.000341	0.000524	0.00069	0.00024	0.000186	0.000138	0.000473	0.000353	0.00153	0.000458	2.18E-05
17-OH-Pregnenolone_Recover	0.000689	0.000367	7.96E-05	0.000212	0.000873	2.82E-05	0.000401	0.000411	0.000541	0.000174	0.000131	0.000216	0.000427	0.000444	0.000486	0.000157	0.00204	0.000134	9.99E-05	0.000702	0.000517	9.24E-05	0.0003	0.000137	0.00059	0.000215	0.00038	0.000352	3.15E-05	0.00103	0.000329	0.00162
17-OH-Pregnenolone_Post-WM	0.000377	0.000144	0.00156	0.000392	0.000271	0.000172	0.000131	0.000125	4.14E-05	0.000749	0.000218	0.000166	0.000231	0.000202	0.000776	0.000112	0.000267	0.000187	0.000291	0.000565	0.00087	0.000608	4.97E-06	0.000457	1.17E-05	0.000295	0.000931	0.000156	0.000289	0.000264	0.000681	0.000402
11-Deoxycortisol_Rest	1.10E-05	2.13E-05	6.83E-06	1.23E-06	0.000166	5.41E-06	1.41E-05	2.71E-05	2.73E-05	1.33E-05	1.78E-05	1.01E-05	1.54E-05	4.18E-06	2.34E-05	2.88E-05	3.27E-05	2.25E-05	6.02E-06	8.54E-06	3.16E-05	3.25E-06	4.68E-06	7.84E-06	2.08E-05	5.23E-05	3.46E-05	3.22E-06	7.74E-06	1.04E-05	7.33E-06	1.91E-06
11-Deoxycortisol_Post-UK	2.18E-05	3.78E-05	7.79E-06	1.32E-05	0.000155	3.93E-05	9.00E-07	6.83E-06	6.30E-06	5.30E-05	2.65E-05	7.66E-06	1.67E-05	4.73E-05	3.21E-05	2.72E-05	5.25E-06	2.12E-05	7.01E-06	7.98E-06	6.05E-06	5.47E-06	8.50E-06	3.38E-05	4.79E-06	1.09E-05	7.45E-06	6.51E-06	6.75E-06	5.68E-06	1.42E-05	1.13E-06
11-Deoxycortisol_Recover	2.88E-05	3.95E-05	2.44E-06	2.18E-05	0.000119	5.68E-06	5.07E-06	2.57E-05	1.23E-05	5.76E-05	9.82E-06	4.44E-05	4.95E-06	3.46E-05	8.82E-06	5.02E-06	4.39E-05	3.68E-06	1.31E-05	1.06E-05	1.42E-05	3.94E-06	1.43E-05	6.63E-06	1.97E-05	2.38E-05	8.47E-06	3.96E-06	1.69E-06	1.52E-05	7.08E-06	1.00E-05
11-Deoxycortisol_Post-WM	4.87E-06	3.64E-05	4.51E-05	4.19E-06	4.06E-05	1.08E-05	3.49E-06	5.82E-06	5.14E-06	4.76E-05	2.33E-05	3.92E-06	1.31E-06	6.44E-06	1.44E-05	8.00E-06	1.23E-05	5.49E-06	1.41E-05	1.83E-05	2.93E-05	8.25E-06	1.55E-06	1.42E-05	2.64E-06	2.55E-05	2.95E-05	8.56E-06	8.54E-06	2.99E-06	1.70E-05	8.51E-06
Corticosterone_Rest	6.01E-05	5.13E-05	3.51E-05	3.89E-05	0.000148	1.35E-05	5.25E-05	9.50E-05	0.000116	7.76E-05	5.51E-05	4.21E-05	3.35E-05	2.61E-05	5.77E-05	6.56E-05	0.000201	9.06E-05	2.72E-05	4.74E-05	0.000126	2.71E-05	3.34E-05	4.13E-05	0.000126	0.0001	7.90E-05	2.75E-05	6.52E-05	4.48E-05	2.08E-05	1.49E-05
Corticosterone_Post-UK	6.09E-05	4.83E-05	1.82E-05	5.02E-05	0.000118	5.06E-05	9.87E-06	6.53E-05	2.23E-05	5.90E-05	4.77E-05	3.58E-05	5.09E-05	7.85E-05	8.45E-05	6.39E-05	4.87E-05	5.16E-05	3.72E-05	3.99E-05	1.73E-05	3.71E-05	1.88E-05	0.000109	6.43E-05	6.55E-05	3.69E-05	1.68E-05	6.99E-05	1.66E-05	3.18E-05	1.07E-05
Corticosterone_Recover	4.52E-05	3.89E-05	1.44E-05	4.45E-05	7.62E-05	1.76E-05	4.74E-05	8.28E-05	3.95E-05	6.64E-05	3.70E-05	4.96E-05	3.56E-05	7.90E-05	4.41E-05	2.49E-05	6.37E-05	3.57E-05	4.34E-05	5.50E-05	2.75E-05	2.25E-05	3.02E-05	2.72E-05	6.21E-05	6.56E-05	2.22E-05	4.03E-05	7.11E-06	4.86E-05	2.54E-05	5.32E-05
Corticosterone_Post-WM	2.70E-05	4.65E-05	4.25E-05	4.20E-05	0.000111	2.89E-05	2.55E-05	4.81E-05	1.76E-05	5.46E-05	4.27E-05	2.23E-05	2.40E-05	4.36E-05	3.65E-05	3.68E-05	4.01E-05	7.09E-05	4.59E-05	5.09E-05	4.98E-05	4.57E-05	1.17E-05	3.70E-05	3.80E-05	4.70E-05	3.25E-05	0.000379	3.91E-05	4.91E-05	4.19E-05	2.79E-05
DHEA_Rest	0.000177	0.000156	7.61E-06	7.00E-05	0.000268	4.00E-05	0.000211	5.01E-05	0.000266	9.55E-05	0.000476	0.000141	0.00012	4.77E-05	9.33E-05	0.000103	0.000572	0.000233	0.000126	3.11E-05	0.000335	2.74E-05	9.62E-05	1.07E-05	0.000204	0.00176	0.000217	1.46E-05	0.000129	0.000132	0.000176	5.04E-06
DHEA_Post-UK	0.00017	0.00018	2.39E-05	0.000217	0.000362	0.000577	2.96E-05	0.000179	0.000181	0.000199	0.000115	0.000104	0.000213	0.000807	0.000173	0.000142	3.84E-05	0.000176	0.000315	0.000246	1.98E-05	8.51E-05	0.000136	6.89E-05	0.00186	0.000396	0.000316	2.77E-05	2.68E-05	0.000133	8.64E-05	5.19E-06
DHEA_Recover	0.000122	0.000199	5.17E-05	0.000268	0.000443	1.72E-05	0.000229	6.07E-05	6.31E-05	0.000195	7.15E-05	0.00026	0.000469	0.000217	4.18E-05	0.000127	8.25E-05	6.34E-05	0.000446	8.97E-05	6.53E-05	3.93E-05	5.14E-05	3.13E-05	0.000162	0.000552	6.61E-05	3.88E-05	3.16E-05	9.65E-05	5.33E-05	6.56E-05
DHEA_Post-WM	0.000311	0.000256	7.47E-05	8.89E-05	6.56E-06	0.000129	9.87E-05	0.000119	3.91E-05	0.000164	0.000279	3.35E-05	0.000206	8.91E-05	4.56E-05	6.38E-05	0.000111	0.00096	0.000154	0.000708	8.86E-05	5.44E-05	7.98E-06	6.68E-05	1.75E-05	0.00043	0.000202	0.00013	4.45E-05	0.000156	6.56E-05	0.000143
Androstenediol_Rest	0.000525	0.00085	0.000287	0.000172	0.00116	0.000169	0.000624	0.000677	0.00443	0.000328	0.000584	0.000292	0.000747	0.000189	0.00101	0.000562	0.000964	0.00056	0.000364	0.000225	0.0048	0.000457	0.000287	0.000176	0.000814	0.00114	0.00103	0.00013	0.000291	0.000699	0.000348	0.000148
Androstenediol_Post-UK	0.000872	0.00083	0.000295	0.000749	0.00127	0.000691	0.000162	0.000388	0.000358	0.00104	0.000735	0.000288	0.000906	0.000801	0.000825	0.000552	0.000655	0.00105	0.00026	0.000257	0.000168	0.000307	0.000789	0.000248	0.00021	0.00103	0.000413	0.000289	0.000293	0.000273	0.00081	0.000141
Androstenediol_Recover	0.000869	0.00054	0.000192	0.000924	0.00103	9.04E-05	0.000633	0.000963	0.000406	0.000825	0.000426	0.000655	0.00024	0.00103	0.000486	0.000338	0.000855	0.000314	0.000278	0.000501	0.000306	0.000323	0.000897	0.0002	0.00057	0.000941	0.000333	0.000402	0.000168	0.000823	0.000361	0.000652
Androstenediol_Post-WM	0.000335	0.000621	0.00159	0.000462	0.00037	0.000285	0.000343	0.000426	0.000373	0.000623	0.000836	0.000231	0.000305	0.00013	0.00028	0.000262	0.00023	0.00316	0.000422	0.000689	0.000871	0.000706	0.000191	0.000329	0.000111	0.00102	0.000542	0.000389	0.000274	0.000242	0.000538	0.000653
Testosterone_Rest	4.61E-05	4.61E-05	9.30E-06	1.90E-06	0.000234	3.33E-05	5.22E-05	5.33E-05	8.09E-06	5.91E-05	0.000142	8.00E-06	4.42E-05	2.21E-05	0.000216	0.000104	8.20E-05	3.60E-05	6.24E-05	4.25E-06	0.000137	5.91E-05	9.58E-06	1.75E-05	0.000236	5.24E-05	0.000218	2.31E-06	0.000109	0.000128	8.08E-06	1.61E-06
Testosterone_Post-UK	7.67E-05	4.18E-05	1.04E-05	2.49E-05	0.000262	0.000277	1.77E-06	7.25E-06	6.87E-06	0.000218	0.000202	1.09E-05	4.52E-05	0.000218	0.000223	5.81E-05	2.05E-05	3.02E-05	7.20E-05	5.13E-06	1.67E-05	5.80E-05	2.19E-05	0.000214	7.09E-05	5.67E-05	8.71E-06	8.63E-06	0.000118	4.97E-05	1.96E-05	1.90E-06
Testosterone_Recover	8.02E-05	8.95E-05	4.03E-06	2.90E-05	0.000212	2.50E-05	5.92E-05	2.82E-05	5.13E-05	0.000242	0.00018	4.82E-05	7.12E-06	0.000218	8.05E-05	1.03E-05	5.98E-05	8.61E-06	0.000139	5.34E-05	5.95E-05	4.07E-05	2.58E-05	2.25E-05	0.000248	2.91E-05	1.34E-05	9.29E-06	3.20E-05	0.000151	5.05E-06	7.96E-05
Testosterone_Post-WM	9.29E-06	4.01E-05	7.08E-05	8.40E-06	6.68E-05	0.00012	7.94E-06	1.31E-05	5.85E-06	0.000167	0.000243	1.86E-06	6.51E-06	1.73E-05	8.44E-05	1.23E-05	4.29E-06	2.58E-05	7.32E-05	3.56E-05	0.000199	0.000176	2.92E-06	7.41E-05	1.74E-05	3.61E-05	5.49E-05	7.40E-06	0.00011	4.21E-05	5.19E-05	2.30E-05
17-OHP_Rest	3.10E-05	2.80E-05	2.36E-05	5.78E-06	9.70E-05	0.000209	0.000104	4.99E-05	0.00021	0.000114	7.58E-05	1.09E-05	4.03E-05	9.79E-05	5.36E-05	0.000218	8.00E-05	0.000189	0.000111	7.27E-05	0.000322	8.69E-05	4.41E-05	4.73E-05	0.000134	8.76E-05	8.76E-05	7.15E-05	0.00011	3.21E-05	5.10E-05	1.78E-05
17-OHP_Post-UK	0.000167	8.17E-05	1.54E-05	4.16E-05	7.82E-05	9.51E-05	4.22E-05	6.05E-05	9.20E-06	0.000132	1.13E-05	2.98E-05	1.95E-05	6.10E-05	0.00011	7.51E-05	9.01E-05	7.08E-05	4.51E-05	6.10E-05	6.30E-05	2.27E-05	2.98E-05	5.70E-05	0.000139	5.15E-05	1.39E-05	2.83E-05	9.42E-05	2.32E-05	1.99E-05	1.36E-05
17-OHP_Recover	3.37E-05	0.000115	1.49E-05	3.00E-05	7.92E-05	2.77E-05	4.19E-05	4.23E-05	1.25E-05	9.94E-05	6.34E-05	0.000132	3.28E-05	8.76E-05	7.66E-05	4.32E-05	5.29E-05	0.00015	2.33E-05	7.59E-05	2.92E-05	3.53E-05	6.20E-05	5.33E-05	0.000106	8.01E-05	1.09E-05	2.05E-05	9.71E-05	9.22E-05	4.26E-05	3.77E-05
17-OHP_Post-WM	3.91E-05	0.000115	1.96E-05	1.31E-05	2.27E-05	7.90E-05	1.37E-05	9.85E-05	4.66E-05	5.12E-05	9.17E-05	4.59E-05	5.61E-06	6.30E-05	6.33E-05	6.00E-05	2.97E-05	8.88E-05	3.54E-05	8.85E-05	0.000106	3.33E-05	1.48E-05	1.59E-05	9.83E-05	4.57E-05	4.77E-05	7.50E-05	5.93E-05	1.57E-05	3.87E-05	3.05E-05
DHT_Rest	5.94E-05	3.65E-05	5.11E-05	0.000173	0.000449	0.000113	5.14E-05	0.000157	0.000708	7.64E-05	0.00021	9.05E-05	5.50E-05	0.000158	5.95E-05	3.60E-05	0.000227	7.42E-05	5.17E-05	4.63E-05	0.000191	2.80E-05	9.43E-05	8.07E-05	0.000212	0.00023	0.000113	0.000127	3.50E-05	0.000112	3.87E-05	0.000197
DHT_Post-UK	0.000157	9.28E-05	9.58E-05	0.000302	0.000242	6.66E-05	0.000194	4.18E-05	4.58E-05	1.29E-05	0.000117	6.88E-05	8.27E-05	0.000344	0.000365	7.44E-05	0.000125	8.65E-05	4.72E-05	7.14E-05	1.96E-05	8.35E-05	4.22E-05	0.000224	0.000366	0.0001	0.000133	3.67E-05	3.62E-05	8.28E-05	6.28E-05	0.000148
DHT_Recover	6.89E-05	6.52E-05	6.08E-05	9.97E-05	5.35E-05	4.27E-05	0.000256	6.68E-05	7.36E-05	5.33E-05	4.29E-05	7.38E-05	5.86E-05	0.000113	4.41E-05	1.97E-05	0.000119	6.67E-05	0.000231	0.000165	8.95E-05	0.000176	0.000315	0.000161	9.64E-05	0.000166	9.56E-05	3.33E-05	0.00014	0.000244	2.06E-05	0.000247
DHT_Post-WM	1.03E-05	7.66E-05	0.0002	8.71E-05	5.13E-05	3.09E-05	7.50E-05	5.94E-05	4.55E-05	0.000136	7.92E-05	0.000138	5.52E-05	0.000105	0.000119	0.000152	0.00221	8.26E-05	6.80E-05	0.000115	0.000221	0.000199	0.000129	8.01E-05	5.37E-05	0.000145	4.23E-05	5.44E-05	4.26E-05	4.52E-05	0.000292	7.26E-05
Pregnenolone_Rest	0.000558	0.000568	0.000275	9.61E-05	0.000109	0.000164	0.000385	0.000197	0.000406	0.000168	0.000359	0.00021	0.000319	0.000264	0.000154	0.000193	0.000136	0.000882	0.00032	0.000117	0.000403	0.000117	0.000238	0.000247	0.000238	0.000285	0.000665	0.000233	7.54E-05	0.000337	0.000234	0.000106
Pregnenolone_Post-UK	0.000218	0.00035	0.000137	0.000322	9.29E-05	0.000242	7.31E-05	0.000321	0.000413	0.000422	0.000371	0.000162	0.000299	0.000246	0.00018	0.000421	7.74E-05	0.000449	0.000465	0.000358	0.00015	0.000117	0.000399	0.000335	0.000238	0.000315	0.000185	0.000136	0.000264	0.000172	0.000449	0.000216
Pregnenolone_Recover	0.000164	0.000136	0.000104	0.000202	0.000162	0.000148	0.000202	0.000771	0.000553	0.00014	0.00048	0.00014	0.000128	0.000665	5.81E-05	0.000278	0.000265	0.00014	0.000647	0.000414	0.000389	0.000218	0.000375	0.000165	0.000339	0.000366	0.00021	0.000169	0.000273	0.000191	0.000506	0.000126
Pregnenolone_Post-WM	0.000919	0.000507	0.000199	0.000212	0.000243	0.000205	0.000137	0.000243	0.000215	0.000332	0.000137	8.58E-05	0.000175	0.00016	5.54E-05	0.00022	1.90E-05	0.000649	0.000245	0.000232	0.000297	0.000284	0.000117	0.000453	0.000206	0.000265	0.000215	0.000173	0.000122	0.000128	0.00028	0.00075
4-Androstene-3,17-dione_Rest	6.19E-05	2.63E-05	6.55E-05	1.89E-05	0.000104	4.53E-05	2.58E-05	4.36E-05	0.000164	7.29E-05	3.18E-05	9.72E-05	1.28E-05	4.14E-05	4.06E-05	4.01E-05	2.72E-05	9.18E-05	8.71E-05	5.53E-05	8.18E-05	4.80E-05	5.75E-05	2.44E-05	4.60E-05	4.99E-05	2.99E-05	2.92E-05	8.44E-05	3.78E-05	8.85E-05	1.53E-05
4-Androstene-3,17-dione_Post-UK	1.61E-05	5.46E-05	6.86E-05	2.85E-05	0.000105	7.07E-05	1.97E-05	9.90E-05	9.66E-05	5.47E-05	3.75E-05	6.94E-05	8.67E-06	3.45E-05	6.06E-05	6.20E-05	6.50E-05	8.66E-05	6.89E-05	5.92E-05	3.69E-05	6.61E-05	2.31E-05	3.45E-05	7.36E-05	6.39E-05	0.000136	8.74E-05	9.00E-05	7.57E-05	2.85E-05	1.91E-05
4-Androstene-3,17-dione_Recover	2.69E-05	2.40E-05	4.18E-05	5.98E-05	9.36E-05	3.93E-05	2.07E-05	4.70E-05	2.43E-05	6.43E-05	5.04E-05	3.52E-05	6.11E-05	2.99E-05	9.38E-05	8.86E-05	3.75E-05	8.54E-05	6.54E-05	2.03E-05	0.00011	5.49E-05	2.32E-05	2.61E-05	4.29E-05	7.63E-05	0.000147	9.13E-05	3.18E-05	2.34E-05	0.000116	2.70E-05
4-Androstene-3,17-dione_Post-WM	6.57E-05	6.49E-05	1.72E-05	8.35E-05	0.000101	0.000122	7.05E-05	0.000105	7.12E-05	3.45E-05	2.18E-05	2.45E-05	5.52E-05	2.20E-05	8.63E-05	0.000103	2.13E-05	8.11E-05	0.000118	3.36E-05	4.74E-05	1.75E-05	1.99E-05	6.67E-05	2.11E-05	6.84E-05	9.21E-05	8.54E-05	8.93E-05	7.66E-05	1.25E-05	2.38E-05
11-Deoxycorticosterone_Rest	0.00429	0.00715	0.0077	0.0038	0.00891	0.00361	0.0113	0.0102	0.00659	0.00526	0.00785	0.0067	0.00807	0.00392	0.00616	0.00978	0.0113	0.00515	0.00712	0.00631	0.00647	0.0057	0.00743	0.0044	0.00714	0.00976	0.00988	0.00492	0.00473	0.0059	0.00633	0.00417
11-Deoxycorticosterone_Post-UK	0.0103	0.00657	0.00391	0.00753	0.00902	0.0072	0.00389	0.00662	0.00788	0.00878	0.00789	0.00592	0.00683	0.00807	0.0075	0.00601	0.00986	0.0077	0.00782	0.00759	0.00362	0.00464	0.00657	0.00678	0.00831	0.00895	0.00627	0.00739	0.00665	0.00566	0.00814	0.00517
11-Deoxycorticosterone_Recover	0.00699	0.0105	0.00379	0.00808	0.00753	0.0043	0.00531	0.00779	0.00502	0.00875	0.00615	0.00883	0.00563	0.00988	0.00526	0.00772	0.00931	0.00517	0.00489	0.00557	0.00713	0.00517	0.00753	0.00503	0.0076	0.0068	0.00672	0.00715	0.00438	0.0102	0.00655	0.00769
11-Deoxycorticosterone_Post-WM	0.00819	0.00825	0.0104	0.0055	0.00699	0.00661	0.00638	0.00576	0.00664	0.00768	0.00785	0.00371	0.00521	0.00438	0.00497	0.00722	0.00673	0.0063	0.00745	0.0132	0.0101	0.00697	0.00426	0.00683	0.00442	0.00923	0.0109	0.00486	0.00547	0.00478	0.00584	0.00676
25-OHD_Rest	3.44E-05	0.000105	0.000363	0.000318	0.000143	0.000478	5.54E-05	0.000109	0.000556	0.000141	5.65E-05	0.000256	3.44E-05	0.000102	5.28E-05	6.54E-05	3.80E-05	0.000105	8.38E-05	0.000311	0.000172	6.42E-05	0.000338	0.000604	0.000104	9.86E-05	9.23E-05	0.000478	0.000275	0.000117	0.00047	0.000164
25-OHD_Post-UK	4.52E-05	4.59E-05	0.000185	6.96E-05	0.000158	6.29E-05	0.000564	0.000201	0.000197	8.15E-05	5.48E-05	0.000117	4.02E-05	5.22E-05	3.15E-05	9.51E-05	0.00108	0.00011	0.00039	6.39E-05	0.000167	0.000242	5.99E-05	7.45E-05	0.000343	4.52E-05	0.00014	0.000102	0.000206	0.000237	0.00013	0.000328
25-OHD_Recover	0.000132	4.09E-05	0.000328	7.87E-05	0.000101	0.000169	4.56E-05	3.57E-05	7.18E-05	5.07E-05	9.60E-05	6.81E-05	0.000201	9.23E-05	0.000114	0.000336	7.73E-05	0.000217	5.26E-05	0.000126	0.000163	0.000199	9.44E-05	0.000662	8.95E-05	0.000118	7.23E-05	0.000306	0.000985	0.000107	0.000533	9.54E-05
25-OHD_Post-WM	0.000404	8.19E-05	9.63E-05	0.00017	0.00037	0.000214	9.88E-05	0.00023	0.000195	0.000158	4.85E-05	0.000759	0.000118	0.000506	0.000108	0.000122	0.00152	0.000207	0.000277	6.88E-05	4.26E-05	8.20E-05	0.000256	0.000135	0.0007	0.000121	5.46E-05	0.000235	0.000149	0.000216	7.07E-05	7.73E-05
Progesterone_Rest	9.01E-05	0.000169	0.000162	3.57E-05	0.000235	4.02E-05	4.61E-05	0.000167	0.000235	0.000176	2.96E-05	0.000138	0.000159	7.67E-05	0.000101	0.000172	0.00016	0.000144	0.000135	0.000152	0.0002	0.000175	0.000239	4.21E-05	0.000111	5.64E-05	9.71E-05	5.67E-05	0.000172	0.000165	0.000227	2.80E-05
Progesterone_Post-UK	0.000259	0.000144	0.000123	1.27E-05	0.000206	0.000165	4.65E-05	0.000307	0.000134	0.000177	0.000184	1.39E-05	0.000175	6.86E-05	0.000131	3.04E-05	2.71E-05	0.000186	9.52E-05	0.000197	3.16E-05	0.000181	0.000185	0.000127	4.63E-05	0.000171	0.000192	0.000243	0.000181	0.000279	0.000262	2.61E-05
Progesterone_Recover	0.000246	5.86E-05	0.0001	4.87E-05	0.00012	4.52E-05	8.36E-05	0.000192	0.000106	0.000261	0.000179	0.000285	0.000197	9.71E-05	0.000191	7.53E-05	0.00019	0.000261	8.91E-05	1.93E-05	9.00E-05	0.000119	0.000167	3.72E-05	0.000134	0.000147	0.000347	0.000199	5.01E-05	0.000257	0.000322	0.000195
Progesterone_Post-WM	4.44E-05	9.38E-05	0.000265	0.000124	7.34E-05	0.000145	0.000175	0.000322	0.0001	0.000147	0.000175	4.61E-05	7.75E-05	2.79E-05	8.01E-05	6.57E-05	6.45E-05	0.000119	9.95E-05	0.000129	6.02E-05	0.000174	4.41E-05	0.000107	4.34E-05	0.000129	0.000203	0.000107	0.000138	0.000174	0.000168	0.000149
Cholesterol_Rest	0.0488	0.0133	0.00303	0.00171	0.0383	0.00272	0.0466	0.0838	0.0203	0.00984	0.0196	0.00322	0.047	0.00567	0.011	0.0327	0.0272	0.025	0.00609	0.00326	0.0145	0.00339	0.00308	0.00273	0.042	0.0646	0.0629	0.00335	0.00312	0.0466	0.00321	0.00333
Cholesterol_Post-UK	0.0655	0.0377	0.00218	0.0448	0.0393	0.0402	0.00253	0.0076	0.00379	0.0393	0.0223	0.00618	0.0502	0.0429	0.0376	0.0228	0.00738	0.0458	0.0055	0.00278	0.00328	0.0074	0.0415	0.0491	0.00371	0.0841	0.00168	0.00157	0.00681	0.0115	0.0284	0.00486
Cholesterol_Recover	0.0336	0.0519	0.00241	0.0174	0.0202	0.00229	0.05	0.0312	0.0457	0.0602	0.0247	0.0692	0.00484	0.0629	0.0023	0.00169	0.0371	0.00403	0.0328	0.039	0.00382	0.00193	0.0529	0.0024	0.0353	0.0352	0.00186	0.00122	0.00464	0.0259	0.00374	0.0402
Cholesterol_Post-WM	0.0048	0.0383	0.025	0.0029	0.00136	0.00222	0.00274	0.0048	0.00376	0.0315	0.0204	0.0035	0.00495	0.00579	0.00231	0.00155	0.00574	0.0301	0.00789	0.0412	0.0278	0.0293	0.00211	0.00246	0.00616	0.0713	0.019	0.00141	0.00535	0.00187	0.034	0.0488
Estriol_Rest	7.42E-05	2.03E-05	2.36E-05	1.62E-05	4.92E-05	3.05E-05	0.000126	7.63E-05	2.51E-05	9.06E-06	0.000127	7.29E-06	3.80E-05	1.99E-05	0.00012	3.39E-05	0.000135	6.13E-05	9.05E-06	3.35E-05	8.82E-06	2.61E-05	4.27E-06	6.82E-06	0.000282	7.15E-05	1.27E-05	1.30E-05	7.99E-06	1.41E-05	2.68E-05	2.66E-05
Estriol_Post-UK	9.05E-05	6.58E-05	1.18E-05	7.40E-05	0.000109	0.000102	7.63E-06	2.04E-05	7.59E-06	6.19E-05	5.86E-05	5.89E-06	3.98E-05	4.97E-05	3.44E-05	8.97E-05	9.81E-06	3.24E-05	3.24E-06	2.43E-05	1.26E-05	1.76E-05	4.47E-05	1.62E-05	1.51E-05	0.000166	9.17E-05	1.40E-05	2.24E-05	1.45E-05	0.000144	3.60E-06

Estriol_Recover	0.000163	5.89E-05	1.00E-05	0.00195	0.000225	9.60E-06	0.000244	0.000109	4.62E-05	6.84E-05	3.97E-05	0.00804	3.81E-05	6.15E-06	1.70E-05	5.23E-06	0.000679	1.22E-05	7.97E-05	0.0001	5.91E-06	5.45E-06	6.61E-05	3.93E-06	4.96E-05	0.000199	6.23E-05	1.52E-05	7.04E-06	4.78E-05	1.09E-05	5.19E-05
Estriol_Post-WM	3.07E-05	0.000297	0.000108	0.000246	2.15E-05	1.26E-05	1.34E-05	9.33E-05	1.06E-05	5.07E-05	4.86E-05	2.64E-05	1.18E-05	1.02E-05	1.91E-05	1.30E-05	1.04E-05	2.75E-05	1.06E-05	3.54E-05	7.34E-05	5.11E-05	1.91E-05	5.98E-06	9.54E-06	7.76E-05	0.000385	1.44E-05	1.48E-05	1.60E-05	2.50E-05	2.97E-05
DHEA-S_Rest	0.00399	0.00472	0.00646	0.00188	0.00285	0.0244	0.00572	0.00768	0.00224	0.012	0.00389	0.000606	0.00465	0.00643	0.00313	0.00334	0.0143	0.00629	0.00794	0.00107	0.00477	0.00463	0.00184	0.00361	0.0127	0.00373	0.00372	0.00406	0.0121	0.00218	0.00338	0.00312
DHEA-S_Post-UK	0.00443	0.00535	0.00424	0.00237	0.00302	0.0153	0.005	0.00766	0.00181	0.0139	0.00549	0.000877	0.00519	0.00516	0.00428	0.00376	0.0148	0.00737	0.00837	0.00114	0.00644	0.00328	0.00188	0.00456	0.00786	0.00516	0.00291	0.00229	0.0143	0.00236	0.0029	0.00376
DHEA-S_Recover	0.00357	0.00418	0.00448	0.00204	0.00278	0.0185	0.0045	0.00708	0.00194	0.0116	0.00455	0.000832	0.00465	0.00452	0.00332	0.00224	0.011	0.00568	0.0102	0.00118	0.00355	0.00353	0.00157	0.00548	0.00639	0.00388	0.00291	0.00158	0.0149	0.00221	0.00191	0.0023
DHEA-S_Post-WM	0.00289	0.00544	0.00408	0.00194	0.00154	0.0122	0.00264	0.00654	0.00167	0.00988	0.00436	0.000746	0.00541	0.00667	0.003	0.00302	0.0142	0.00611	0.0108	0.00107	0.00423	0.00364	0.00192	0.00276	0.008	0.00346	0.00269	0.0019	0.00932	0.00172	0.00176	0.00294
17β-Estradiol_Rest	1.75E-05	6.20E-06	3.59E-06	7.97E-06	7.83E-06	1.94E-06	5.00E-06	1.17E-05	0.000108	3.93E-06	1.35E-05	6.85E-07	8.55E-06	4.48E-06	1.23E-05	1.10E-05	1.23E-05	4.01E-06	3.23E-06	8.18E-06	4.68E-05	9.38E-06	6.38E-06	5.35E-06	1.85E-05	7.67E-06	1.52E-05	8.84E-07	9.99E-06	6.46E-06	8.27E-06	5.59E-06
17β-Estradiol_Post-UK	6.42E-06	1.32E-05	8.85E-07	1.38E-05	1.18E-05	1.65E-05	4.61E-06	8.14E-06	5.41E-06	1.46E-05	3.17E-06	2.63E-06	7.68E-06	4.59E-06	1.13E-05	4.91E-06	5.21E-06	9.09E-06	6.47E-06	8.55E-06	4.54E-06	4.22E-06	1.16E-05	1.64E-05	3.01E-06	1.31E-05	1.08E-05	6.80E-06	5.01E-06	4.44E-06	1.07E-05	3.25E-06
17β-Estradiol_Recover	1.88E-05	1.06E-05	8.81E-06	2.04E-05	7.46E-06	6.71E-06	8.54E-06	8.26E-06	3.94E-06	1.05E-05	3.05E-06	7.91E-06	5.69E-06	1.52E-05	5.42E-06	3.06E-06	1.27E-05	2.08E-06	7.05E-06	5.76E-06	4.65E-06	5.47E-06	7.30E-06	1.82E-06	6.96E-06	1.10E-05	5.96E-06	6.47E-06	8.33E-06	1.95E-05	4.60E-06	5.80E-06
17β-Estradiol_Post-WM	6.52E-06	8.05E-06	1.56E-05	3.50E-06	2.25E-06	5.30E-06	5.68E-06	3.64E-06	5.57E-06	7.46E-06	4.36E-06	8.70E-06	2.67E-06	8.24E-06	7.62E-06	4.50E-06	3.36E-06	4.31E-05	8.05E-06	7.43E-06	5.10E-06	3.10E-06	8.63E-06	7.93E-06	6.71E-06	1.18E-05	1.01E-05	8.66E-06	6.85E-06	4.62E-06	7.42E-06	8.99E-06
Aldosterone_Rest	1.89E-05	1.96E-05	1.09E-05	3.59E-06	1.43E-05	1.20E-05	0.000116	7.57E-05	1.42E-05	9.12E-07	5.41E-05	1.45E-05	1.57E-05	3.68E-06	1.23E-05	2.55E-05	8.20E-05	0.000114	1.20E-05	9.19E-06	1.34E-05	5.32E-06	1.66E-05	1.23E-05	2.07E-05	2.21E-05	3.94E-05	6.10E-06	8.78E-06	3.80E-05	3.00E-05	8.20E-06
Aldosterone_Post-UK	2.52E-05	5.91E-05	1.10E-05	3.12E-05	2.19E-05	1.58E-05	5.04E-05	3.37E-05	1.37E-05	5.39E-05	4.83E-05	8.50E-06	2.48E-05	2.57E-05	8.60E-06	2.92E-05	4.41E-05	0.00012	1.29E-05	9.46E-06	1.83E-05	9.13E-06	3.58E-05	5.86E-05	1.80E-05	1.16E-05	2.93E-05	7.42E-06	1.64E-05	1.54E-05	3.81E-05	7.19E-06
Aldosterone_Recover	3.29E-05	2.06E-05	7.07E-06	2.24E-05	1.73E-05	8.54E-06	6.21E-05	3.56E-05	2.83E-05	2.08E-05	5.66E-05	4.74E-05	8.93E-06	3.94E-05	2.59E-06	5.87E-06	0.000111	4.51E-05	4.04E-05	3.85E-05	1.69E-05	2.55E-06	3.05E-05	8.57E-06	3.23E-05	3.03E-05	1.92E-05	4.61E-06	3.54E-06	4.96E-05	1.53E-05	1.63E-05
Aldosterone_Post-WM	9.11E-06	3.15E-05	1.84E-05	1.01E-05	3.42E-06	5.74E-06	2.24E-05	2.10E-05	1.32E-05	7.85E-05	4.25E-05	1.97E-05	2.74E-06	1.65E-06	7.64E-06	1.53E-05	0.000103	6.65E-05	3.10E-05	3.80E-05	3.75E-05	2.28E-05	9.56E-06	5.20E-06	1.39E-05	4.91E-05	3.53E-05	9.41E-06	3.40E-06	6.59E-06	1.12E-05	1.63E-05
Estrone_Rest	5.04E-06	2.08E-06	2.28E-06	1.42E-06	4.12E-06	2.85E-06	2.93E-06	8.02E-06	1.45E-05	3.23E-06	1.58E-06	1.77E-06	2.15E-06	7.72E-07	3.32E-06	4.32E-06	4.82E-06	8.96E-06	1.88E-06	2.20E-06	3.25E-06	2.14E-06	4.60E-06	1.18E-06	2.46E-06	3.07E-06	2.32E-06	5.09E-06	1.01E-06	1.52E-06	5.86E-06	1.00E-06
Estrone_Post-UK	6.62E-06	5.10E-06	2.02E-06	1.78E-06	1.33E-06	2.88E-06	2.62E-06	4.65E-06	2.14E-06	3.49E-06	2.06E-06	1.45E-06	2.97E-06	2.48E-06	3.35E-06	7.12E-06	1.15E-06	1.10E-05	8.53E-07	8.58E-07	2.10E-06	2.64E-06	2.92E-06	6.37E-07	6.47E-07	6.75E-06	7.81E-06	4.53E-06	2.11E-06	1.00E-06	6.66E-06	5.74E-07
Estrone_Recover	7.15E-06	2.40E-06	6.75E-07	1.74E-06	2.49E-06	3.08E-06	2.55E-06	3.56E-06	2.02E-06	3.47E-06	3.97E-06	1.22E-06	1.77E-06	2.32E-06	2.55E-06	8.67E-07	4.45E-06	4.63E-06	2.04E-06	2.78E-06	3.87E-06	5.06E-07	4.20E-06	3.96E-06	2.44E-06	6.98E-06	8.07E-06	4.82E-06	1.68E-06	1.30E-06	4.30E-06	1.63E-06
Estrone_Post-WM	4.69E-06	2.93E-06	1.27E-06	1.27E-06	1.40E-06	3.38E-06	2.28E-06	5.18E-06	1.74E-06	4.07E-06	1.11E-06	1.29E-06	1.42E-06	2.93E-06	2.16E-06	3.98E-06	2.70E-06	1.67E-05	7.10E-07	2.46E-06	3.72E-06	1.70E-06	4.25E-06	2.04E-06	1.99E-06	5.87E-06	1.24E-05	4.98E-06	4.27E-06	1.05E-06	5.08E-06	1.12E-06
Lithocholic acid_Rest	8.39E-05	0.000153	0.000133	0.000134	0.000152	0.000119	0.000173	0.000121	0.000102	0.000108	0.000149	0.000114	0.000144	0.000147	0.000114	9.21E-05	0.000153	0.00011	9.29E-05	8.81E-05	0.000121	8.88E-05	0.000106	0.000153	0.000116	0.000157	0.000197	0.000108	0.000106	0.000106	9.12E-05	0.000157
Lithocholic acid_Post-UK	0.000124	0.000123	6.63E-05	0.000158	0.000173	0.00013	0.000108	0.0001	0.000126	0.000159	0.000143	0.000103	0.000134	0.000142	0.000143	0.000115	0.000104	0.000155	0.000143	0.000108	0.000143	6.77E-05	0.000125	0.000163	0.000113	0.00018	8.82E-05	0.000106	9.40E-05	0.000125	0.000152	0.000166
Lithocholic acid_Recover	0.000107	8.34E-05	0.000122	0.000133	0.000147	9.83E-05	9.36E-05	0.000183	9.84E-05	0.000141	0.000126	8.93E-05	9.21E-05	0.000197	9.91E-05	0.00011	0.000176	8.74E-05	9.92E-05	0.000109	0.000109	0.000107	0.000138	0.000146	0.000126	0.000148	0.000107	0.000125	0.000184	0.000116	0.000102	9.61E-05
Lithocholic acid_Post-WM	0.000104	0.000151	9.93E-05	9.53E-05	0.000102	8.34E-05	0.000105	0.000118	0.000118	0.000164	9.59E-05	0.000152	8.28E-05	0.000153	0.0001	0.00012	0.000135	0.000126	0.000108	0.000128	0.000151	0.000119	0.000124	0.000104	0.000149	0.000166	9.82E-05	0.000102	8.61E-05	8.89E-05	0.00011	0.000158
Urea_Rest	195	395	266	269	326	256	483	175	265	289	207	1120	247	376	1810	262	2290	432	534	571	434	393	647	782	238	229	538	470	417	860	668	150
Pyrazole_Rest	0	0	0	0	0	0	0	0	0	0	0	0	0	0	0	0	0	0	0	0	0	0	0	0	0	0	0	0	0	0	0	0
3-Aminopropionitrile_Rest	0	0	0	0	0	0	0	0	0	0	0	0	0	0	0	0	0	0	0	0	0	0	0	0	0	0	0	0	0	0	0	0
Methylguanidine_Rest	0	0	0	0	0	0	0	0	0	0	0	0	0	0	0	0	0	0	0	0	0	0	0	0	0	0	0	0	0	0	0	0
Isobutylamine_Rest	0	0	0	0	0	0	0	0	0	0	0	0	0	0	0	0	0	0	0	0	0	0	0	0	0	0	0	0	0	0	0	0
1,3-Diaminopropane_Rest	0.139	0	0.0897	0	0	0.379	0.137	0.0949	0.11	0.387	0.133	0.075	0.0734	0.112	0	0.12	0.0538	0.244	0.262	0.1	0.723	0.109	0.106	0.281	0.629	0.252	0	0.243	0.241	0.0601	0.0461	0.0312
Gly_Rest	5.5	11.6	22.6	6.48	18.8	16.8	19.9	12.3	17.3	53.4	4.34	8.38	8.12	10.5	60.9	11.1	14.2	10.9	7.56	11.6	30	38.8	27.1	13.6	15.2	45.1	7.61	9.09	9.72	54.4	9.65	4.22
Trimethylamine *N*-oxide_Rest	0	0.0672	0.215	0.0314	0	0	0.0324	0.038	0.0878	0.0228	0.128	0.0324	0.021	0.0308	0.157	0	1.43	0	0.713	0.0264	0.0889	0.0408	0.044	0	0	0.246	0.0147	0.175	0.0387	0.0343	0.0804	0
Isopropanolamine_Rest	0	1.03	0.27	0.0596	0.105	0.148	0.348	0.18	0.106	0.556	0.0784	0.235	0.158	0.0878	0.0861	0.0338	0.115	0.115	0.116	0.0641	0.195	0.148	0.138	0.112	0.0657	0.0542	0	0.0905	0.106	0.104	0.355	0.0263
Hydroxyurea_Rest	0	0	0	0	0	0	0	0	0	0	0	0	0	0	0	0	0	0	0	0	0	0	0	0	0	0	0	0	0	0	0	0
Trimethylsulfonium_Rest	0	0	0	0	0	0	0	0	0	0	0	0	0	0	0	0	0	0	0	0	0	0	0	0	0	0	0	0	0	0	0	0
Cysteamine_Rest	0	0	0	0	0	0	0	0	0	0	0	0	0	0	0	0	0	0	0	0	0	0	0	0	0	0	0	0	0	0	0	0
Isoamylamine_Rest	0	0	0	0	0	0	0	0	0	0	0	0	0	0	0	0	0	0	0	0	0	0	0	0	0	0	0	0	0	0	0	0
Putrescine(1,4-Butanediamine)_Rest	7.06	23.4	20.8	2.82	8.2	17.3	21.1	11.4	13	45.6	2.16	11	4.71	6.45	9.12	12.5	1.97	7.57	8.01	4.7	25.1	18.8	13.6	6.95	9.06	13.8	5.38	2.05	12.7	19.3	11.2	1.31
β-Ala_Rest	0.225	0.609	0.331	0.208	0.498	0.327	0.873	0.411	0.22	0.801	0.443	0.239	0.777	0.346	1.09	0.235	1.09	0.436	0.594	0.377	0.313	0.386	0.254	0.395	0.619	0.329	0.698	0.352	0.665	0.256	0.28	0.199
Ala_Rest	3.23	6.85	4.3	4.75	5.66	9.38	7.1	9.73	6.68	12.1	3.59	5.38	4.5	6.62	16.5	4.01	8.06	9.03	5.29	5.85	9.3	10.3	3.88	7.73	9.51	7.76	8.19	6.23	7.23	4.65	8.5	2.36
Sarcosine_Rest	0.596	2.31	3.47	1.27	1.38	1.44	2.38	1.46	1.12	4.37	2.02	1.76	1.14	1.2	1.53	1.76	4.06	1.25	0.899	1.51	1.98	3.34	2.23	1.23	1.9	1.76	2.93	0.881	1.97	1.22	1.79	0.908
3-Aminopropane-1,2-diol_Rest	0	0.0438	0.0256	0	0	0	0	0.0284	0	0	0	0	0.0154	0	0	0	0	0	0	0	0	0	0	0	0	0	0.472	0	0	0.0182	0.0254	0
Aniline_Rest	0	0	0	0	0	0	0	0	0	0	0	0	0	0	0	0	0	0	0	0	0	0	0	0	0	0	0	0	0	0	0	0
4-Hydroxymethylimidazole_Rest	0	0	0	0	0	0	0	0	0	0	0	0	0	0	0	0	0	0	0	0	0	0	0	0	0	0	0	0	0	0	0	0
1-Methyl-2-pyrrolidinone_Rest	0	0	0	0	0	0	0	0	0	0	0	0	0	0	0	0	0	0	0	0	0	0	0	0	0	0	0	0	0	0	0	0
Cyclohexylamine_Rest	0	0	0	0	0	0	0	0	0	0	0	0	0	0	0	0	0	0	0	0	0	0	0	0	0	0	0	0	0	0	0	0
1-Aminocyclopropane-1-carboxylate_Rest	0	0	0	0	0	0	0	0	0	0	0	0	0	0	0	0	0	0	0	0	0	0	0	0	0	0	0	0	0	0	0	0
Betaine aldehyde_Rest	0	0	0	0	0	0	0	0	0	0	0	0	0	0	0	0	0	0	0	0	0	0	0	0	0	0	0	0	0	0	0	0
Spermine_Rest	0	0.211	0.097	0	0.186	0.0537	0.0592	0.0898	0.16	0.0515	0	0.103	0.0716	0.0334	0.179	0	0.0444	0.0403	0.0368	0.0179	0.0396	0.109	0.0394	0.0754	0	0.0181	0.0557	0.08	0.0373	0.0703	0.165	0.0214
Hexylamine_Rest	0	0	0	0	0	0	0	0	0	0	0	0	0	0	0	0	0	0	0	0	0	0	0	0	0	0	0	0	0	0	0	0
Cadaverine_Rest	0.601	0.802	14.4	0.307	4.41	6.6	4.27	6.33	3.04	8.16	1.36	1.58	1.36	1.24	0.696	4.38	1.85	1.38	4.61	0.657	2.72	3	0.783	2.76	3.34	3.08	0.959	0.658	5.34	4	1.5	0.904
GABA_Rest	0.169	0.35	0.427	0.202	0.235	0.407	0.357	0.543	0.228	0.553	0.28	0.259	1.31	0.16	0.233	0.431	0.421	0.42	0.207	0.342	0.409	0.558	0.354	0.427	0.4	0.427	0.235	0.146	0.385	0.829	0.608	0.151
3-Aminoisobutyrate_Rest	0.0762	0.657	0.126	0.103	0.255	0.349	0.259	0.363	0.139	0.168	0.39	0.343	0.288	0.137	0.33	0.261	0.247	0.172	0.365	0.178	0.0951	0.276	0.432	0.257	0.504	0.41	0.134	0.351	0.24	0.144	0.586	0.0819
2AB_Rest	0.0965	0.539	0.367	0.108	0.135	0.646	0.394	0.456	0.216	0.846	0.101	0.303	0.196	0.2	0.492	0.209	0.451	0.221	0.174	0.179	0.442	0.301	0.292	0.274	0.234	0.198	0.288	0.144	0.43	0.125	0.265	0.0914
N-Methylalanine_Rest	0	0	0	0	0	0	0	0	0	0	0	0	0	0	0	0	0	0	0	0	0	0	0	0	0	0	0	0	0	0	0	0
N,N-Dimethylglycine_Rest	0.205	0.26	0.0972	0	0.107	0.224	0.153	0	0.0865	0.173	0.116	0.0867	0.146	0.0704	0.134	0	0.235	0.0971	0.155	0.162	0.098	0	0.114	0.131	0.101	0.112	0	0.144	0.0982	0.109	0.116	0.0672
Choline_Rest	1.52	3.36	2.62	1.31	2.42	4.59	2.98	4.8	2.99	4.17	1.24	1.9	2.9	3.34	4.09	2.24	4.4	3.11	3.27	3.29	4.16	2.09	1.91	3.33	4.14	3.84	4.19	4.83	3.42	1.06	3.02	1.34
2,3-Diaminopropionate_Rest	0	0	0	0	0	0	0	0	0	0	0	0	0	0	0	0	0	0	0	0	0	0	0	0	0	0	0	0	0	0	0	0
Ser_Rest	1.36	3.43	2.93	0.852	3.83	4.03	3.42	4.12	2.5	4.57	1.86	1.9	1.83	3.2	7.49	1.49	5.82	5.82	2.68	2.17	5.8	3.65	2.5	3.07	3.84	4.46	2.78	4.91	2.99	2.11	3	1.77
Diethanolamine_Rest	1.85	1.18	1.84	1.33	4.41	0.985	2.17	4.02	1.38	0.826	0.792	2.28	1.21	5.35	0.981	1.38	1.07	1.39	2.52	1.1	0.845	1.57	0.786	1.08	1.32	1.97	1.82	1.1	0.779	1.16	1.04	3.76
N-Methylaniline_Rest	0	0	0	0	0	0	0	0	0	0	0	0	0	0	0	0	0	0	0	0	0	0	0	0	0	0	0	0	0	0	0	0
β-Ala-Lys_Rest	0	0.0159	0.0113	0	0	0	0	0.00762	0.00788	0	0	0	0.0117	0	0	0.0171	0	0.0299	0.0127	0	0.0528	0.0125	0	0	0	0	0	0.0148	0	0.00961	0.00853	0
Hypotaurine_Rest	0.259	0.388	0.458	0.251	0.252	0.197	0.361	1.31	0	0.341	0.157	0.225	0.412	0.443	0.819	0.173	0	0	0	0.219	0.633	0.663	0.272	0.348	0.405	0.385	0	0.739	0.327	0	0.442	0
2-Aminophenol_Rest	0	0	0	0	0	0	0	0	0	0	0	0	0	0	0	0	0	0	0	0	0	0	0	0	0	0	0	0	0	0	0	0
Cytosine_Rest	0	0	0.0717	0.0316	0.0425	0	0.0631	0.0679	0.05	0.0598	0.0585	0.0588	0.0321	0.0233	0.0481	0.0413	0.127	0.0704	0.0565	0.082	0.0463	0.0371	0.0207	0	0.0741	0	0.117	0.0629	0.0755	0	0.0952	0.0229
Histamine_Rest	0.162	0.128	0.826	0.452	0.0935	0.0359	0.133	0.344	0.0397	0.0282	0.0408	0.0258	0.0301	0.0302	0	0.0265	0.0487	0.349	0.212	0.128	0.0457	0.121	0.0446	0.0226	0.0228	0.0452	0	0	1.05	0.0305	0.0423	0.0202
Uracil_Rest	0	0.29	0.852	0	0	0.822	0.34	2.98	0.557	0	0	0.908	0.997	0.622	3.13	0	2.3	0	0.232	2.03	1.06	1.56	0	1.47	0.874	0	2.49	1.83	0	0	0	0.46
Carnosine_Rest	0.0234	0.0186	0.0229	0	0.131	0	0.133	0.0355	0	0.0534	0.0341	0	0.188	0	0.244	0	0.0322	0.0204	0.0376	0.0264	0.0233	0.0337	0.0161	0	0.0162	0	0.025	0.159	0.0167	0	0	0.04
Creatinine_Rest	0.711	2.57	1.17	0.639	0.913	0.813	0.945	1.14	0.867	1.81	1.06	0.736	1.03	1.2	3.69	0.604	2.49	0.807	2.13	0.842	1.58	1.02	1.4	1.43	1.67	1.37	0.986	0.835	1.61	1	1.01	0.527
Muscimol_Rest	0	0	0	0	0	0	0	0	0	0	0	0	0	0	0	0	0	0	0	0	0	0	0	0	0	0	0	0	0	0	0	0
β-Cyanoalanine_Rest	0	0	0	0	0	0	0	0	0	0	0	0	0	0	0	0	0	0	0	0	0	0	0	0	0	0	0	0	0	0	0	0
Dihydrouracil_Rest	0	0	0	0	0	0	0	0	0	0	0	0	0	0	0	0	0	0	0	0	0	0	0	0	0	0	0	0	0	0	0	0
Pro_Rest	3.72	5.32	29.9	3.99	13.2	21.6	14	9.13	8.62	110	2.42	14.5	4.33	7.7	46.1	9.65	7.04	6.97	5.45	11.3	28.4	28.1	12.8	11.4	5.39	33	3.01	5.81	7.56	90.4	6.56	3.62
Guanidinoacetate_Rest	0	0	0.459	0.264	0.404	0	0	0.361	0	0	0.192	0	0	0.629	0	0	0.378	0.325	0	0.269	0	0	0.496	0	0.444	0.699	0.335	0.15	0.353	0.581	0.767	0
5-Aminovalerate_Rest	35.1	164	119	28.4	29.7	146	137	84.2	76.4	427	27.2	77.3	48.5	68.6	53.7	81.1	45.4	56	133	72.3	200	112	102	63.4	58.9	107	24.9	5.83	107	77.9	59.9	7.25
Val_Rest	0.966	2.73	4.67	0.468	1.97	5.9	2.92	3.67	1.41	10.4	0.853	1.64	1.81	1.29	5.56	1.35	3.63	3.09	1.19	1.06	4.5	2.06	6.54	1.73	2.13	1.29	2.27	1.81	2.97	0.649	3.91	0.798
Betaine_Rest	0.43	1.94	0.797	0.733	0.746	0.877	0.822	1.01	0.841	1.26	0.475	0.849	0.46	0.451	1.39	0.367	0.85	0.651	1.84	0.993	0.829	0.763	1.32	0.85	1.21	0.809	0.412	0.927	0.687	1.85	1.02	0.595
Benzimidazole_Rest	0	0	0	0	0	0	0	0	0	0	0	0	0	0	0	0	0	0	0	0	0	0	0	0	0	0	0	0	0	0	0	0
2,4-Diaminobutyrate_Rest	0	0	0	0	0	0	0	0	0	0	0	0	0	0	0	0	0	0	0	0	0	0	0	0	0	0	0	0	0	0	0	0
4-Amino-3-hydroxybutyrate_Rest	0	0	0	0	0	0	0	0	0	0	0	0	0	0	0	0	0	0	0	0	0	0	0	0	0	0	0	0	0	0	0	0
Homoserine_Rest	0.0475	0.0689	0	0	0.0832	0.0912	0.0869	0	0.0648	0.156	0.0498	0.0946	0.0914	0.0712	0.0823	0.0716	0.0931	0	0	0	0.139	0.113	0.0771	0.0765	0	0.11	0	0	0.0754	0.118	0	0
α-Methylserine_Rest	0	0	0	0	0	0	0	0	0	0	0	0	0	0	0	0	0	0	0	0	0	0	0	0	0	0	0	0	0	0	0	0
Thr_Rest	0.516	1.63	1.14	0.472	1.23	2.35	1.54	1.99	1.04	1.23	0.692	0.698	0.916	0.987	4.2	0.51	1.85	2.28	0.841	0.766	1.9	1.49	0.698	1.04	1.2	0.899	1.55	1.39	0.993	0.669	1.82	0.715
Purine_Rest	0	0	0	0	0	0	0	0	0	0	0	0	0	0	0	0	0	0	0	0	0	0	0	0	0	0	0	0	0	0	0	0
Anserine_Rest	0.0497	0	0.0629	0	0.0608	0.0213	0.352	0.0118	0	0.034	0.0227	0.026	0.719	0.0259	0.105	0	0.111	0.023	0.101	0.121	0	0	0	0	0	0	0	0.26	0.0954	0.0316	0	0
Homocarnosine_Rest	0	0	0	0	0	0	0	0	0	0.012	0	0	0.0826	0	0.0321	0	0	0	0	0	0	0	0	0	0	0	0	0	0	0	0	0
Benzamidine_Rest	0	0	0	0	0	0	0	0	0	0	0	0	0	0	0	0	0	0	0	0	0	0	0	0	0	0	0	0	0	0	0	0
Cys_Rest	0	0	0	0	0	0	0	0	0	0	0	0	0	0	0	0	0	0	0	0	0	0	0	0	0	0	0	0	0	0	0	0
Benzamide_Rest	1.51	16.5	3.43	3.19	3.16	5.13	7.39	3.5	3.22	13.3	4.81	5.24	1.76	5.03	3.16	1.84	4.83	1.59	2.79	2.81	3.98	2.38	4.63	3.6	4.53	5.5	5.4	1.55	5.17	1.75	5.96	2.05
Phenethylamine_Rest	0	0	0	0	0	0	0	0	0	0	0	0	0	0	0	0	0	0	0	0	0	0	0	0	0	0	0	0	0	0	0	0
α-Methylbenzylamine_Rest	0	0	0	0	0	0	0	0	0	0	0	0	0	0	0	0	0	0	0	0	0	0	0	0	0	0	0	0	0	0	0	0
2,4-Dimethylaniline_Rest	0	0	0	0	0	0	0	0	0	0	0	0	0	0	0	0	0	0	0	0	0	0	0	0	0	0	0	0	0	0	0	0
Isonicotinamide_Rest	0	0	0	0	0	0	0	0	0	0	0	0	0	0	0	0	0	0	0	0	0	0	0	0	0	0	0	0	0	0	0	0
Nicotinamide_Rest	0.06	0.135	0.115	0.0503	0.126	0.133	0.133	0.14	0.134	0.292	0.0614	0.0555	0.062	0.058	0.161	0.0795	0	0.0944	0.117	0.029	0.268	0.0827	0.064	0.0994	0.125	0.0725	0	0.144	0.107	0.106	0.0973	0.119

Picolinamide_Rest	0	0	0	0	0	0	0	0	0	0	0	0	0	0	0	0	0	0	0	0	0	0	0	0	0	0	0	0	0	0	0	0
3-Chloroalanine_Rest	0	0	0	0	0	0	0	0	0	0	0	0	0	0	0	0	0	0	0	0	0	0	0	0	0	0	0	0	0	0	0	0
Taurine_Rest	24.2	76.5	47.3	25.2	53.9	38	47.1	74.1	35.3	54.4	27	19.1	47.6	40.1	35.4	29.2	42.9	32.5	39.5	22.1	48.5	50.7	33	40.3	61.7	35.9	37.7	47	41.3	20.5	45.3	23.3
5-Methylcytosine_Rest	0	0	0	0	0	0	0	0	0	0	0	0	0	0	0	0	0	0	0	0	0	0	0	0	0	0	0	0	0	0	0	0
1-Methylhistamine_Rest	0	0	0.017	0.00719	0	0	0	0.00814	0	0	0	0	0	0	0	0	0	0.0168	0.00805	0.0108	0	0.00899	0	0	0	0	0	0	0.0163	0	0	0
Thymine_Rest	2.27	0	2.26	0.969	2.59	2.5	1.71	7.13	3.82	1.31	1.89	1.51	3.04	5.06	2.29	4.6	5.3	2.07	0	3.11	4.07	0	1.13	1.87	3.13	1.79	2.13	3.69	1.51	6.8	3.86	2.05
Imidazole-4-acetate_Rest	0	0	0	0	0	0	0	0	0	0	0	0	0	0	0	0	0	0	0	0	0	0	0	0	0	0	0	0	0	0	0	0
Melamine_Rest	0	0	0	0	0	0	0	0	0	0	0	0	0	0	0	0	0	0	0	0	0	0	0	0	0	0	0	0	0	0	0.0413	0
1-Amino-1-cyclopentanecarboxylate_Rest	0	0	0	0	0	0	0	0	0	0	0	0	0	0	0	0	0	0	0	0	0	0	0	0	0	0	0	0	0	0	0	0
Pipecolate_Rest	0.0668	0.0649	0.163	0.146	0.062	0.0934	0.103	0.0632	0.101	0.206	0.166	0.0421	0.0583	0.0367	0.1	0	0.37	0.0453	0.215	0.0482	0.111	0.0731	0.046	0.0687	0.16	0.113	0.0639	0.049	0.0814	0.0719	0.121	0.0417
Metformin_Rest	0	0	0	0	0	0	0	0	0	0	0	0	0	0	0	0	0	0	0	0	0	0	0	0	0	0	0	0	0	0	0	0
Octylamine_Rest	0	0	0	0	0	0	0	0	0	0	0	0	0	0	0	0	0	0	0	0	0	0	0	0	0	0	0	0	0	0	0	0
N-Acetylputrescine_Rest	0.25	0.807	0.777	0.138	0.313	0.401	0.721	0.533	0.347	1.79	0.281	0.352	0.254	0.318	0.197	0.294	0.424	0.218	0.606	0.315	1.24	0.642	0.746	0.246	0.449	0.789	0.256	0.133	0.546	0.72	0.506	0.0714
Agmatine_Rest	0.0346	0.106	0.057	0.034	0.0548	0.0526	0.0467	0.0273	0.0662	0.0937	0.0264	0.0347	0.0298	0.0464	0	0.0426	0.0457	0.0338	0.0699	0	0.0923	0.0381	0	0.0409	0.0426	0.0368	0.039	0.0211	0.0434	0.0617	0	0.0122
Hydroxyproline_Rest	0	0.214	0.413	0.149	0.214	0.302	0.292	0.188	0.172	0.276	0.245	0.157	0.148	0.222	1.1	0.213	0.712	0.236	0.158	0.189	0.174	0.265	0.087	0.169	0.193	0.194	0.271	0.195	0.151	0.117	0.217	0.141
5-Aminolevulinate_Rest	0	0.0393	0	0.0116	0.029	0.0355	0.0301	0	0	0.0301	0.0175	0.0166	0	0.0105	0.0243	0	0	0	0.0291	0	0.0154	0	0.022	0.0266	0.0125	0.0236	0	0	0	0.0308	0.0275	0.0106
Creatine_Rest	2.68	7.13	6.88	3.36	3.08	5.21	4	7	4.48	6.9	2.67	3.85	3.42	4.24	14.7	1.8	8.85	3.81	4.16	4.32	4.46	5.72	2.77	3.89	6.95	4.68	5.15	6.93	3.86	2.98	6.03	2.8
6-Aminohexanoate_Rest	0	0.363	0	0	0	0	0	0	0	0	0	0.358	0	0	0	0	0	0	0	0	0	0	0	0	0	0	0	0	0	0	0	0
β-Leucine_Rest	0	0	0	0	0	0	0	0	0	0	0	0	0	0	0	0	0	0	0	0	0	0	0	0	0	0	0	0	0	0	0	0
Ile_Rest	0.189	0.665	1.21	0.1	0.603	1.34	0.677	1.91	0.266	2.38	0.207	0.282	0.667	0.388	1.41	0.287	1.81	1.16	0.368	0.287	1.04	0.542	2.23	0.458	0.459	0.355	0.661	0.871	0.622	0.152	1.29	0.223
Leu_Rest	0.603	2.2	4.58	0.275	2.31	3.83	2.96	4.63	1.52	7.14	0.481	1.47	1.33	1.2	4.75	1.01	3.76	2.64	1.04	0.98	3.52	1.65	5.63	1.5	1.29	1.26	1.5	2.41	2.12	0.928	3.08	0.453
Bis(3-aminopropyl)amine_Rest	0	0	0	0	0	0	0	0	0	0	0	0	0	0	0	0	0	0	0	0	0	0	0	0	0	0	0	0	0	0	0	0
Gly-Gly_Rest	0	0	0.172	0.0693	0.0852	0.12	0.178	0	0.079	0.366	0.0654	0.0937	0	0.0328	0.132	0.0573	0.0428	0.116	0.105	0.0662	0.312	0.055	0.0862	0.112	0.148	0.167	0	0	0.0864	0.17	0.0722	0.042
Asn_Rest	0.125	0.4	0.216	0.169	0.796	0	0	0.292	0	0	0	0.239	0.264	0	2.49	0	0.32	0.0336	0.0864	0.205	0	0.465	0.129	0	0	0	0.19	0.272	0	0.452	0.434	0.195
Ornithine_Rest	1.34	2.4	5.73	0.736	3.59	3.18	4.45	1.2	2.88	10.1	0.719	3.21	0.91	2.33	4.28	2.49	1.12	3.33	1.68	1.29	8.61	3.65	3.26	2.47	2.2	7.52	0.622	1.11	4.01	6.76	1.07	0.477
Asp_Rest	1.31	2.28	2.28	1	2.3	3.11	3.02	1.69	2.48	4.11	1.84	2.04	1.62	2.91	10.7	2.09	3.54	4.86	2.16	3.21	7.08	2.98	2.34	3.12	4.17	4.41	2.74	3.18	3.97	2.18	2.72	0.956
2-Aminobenzimidazole_Rest	0	0	0	0	0	0	0	0	0	0	0	0	0	0	0	0	0	0	0	0	0	0	0	0	0	0	0	0	0	0	0	0
Homocysteine_Rest	0	0	0	0	0	0	0	0	0	0	0	0	0	0	0	0	0	0	0	0	0	0	0	0	0	0	0	0	0	0	0	0
Adenine_Rest	0.1	0.215	0.226	0.0624	0.125	0.36	0.24	0.254	0.186	0.293	0.169	0.0959	0.0764	0.265	0.157	0.191	0.566	0.31	0.263	0.146	0.289	0.152	0.147	0.488	0.433	0.17	0	0.275	0.239	0.0814	0.238	0.0733
Hypoxanthine_Rest	0.874	1.92	1.47	0.569	0.931	3.04	1.52	4.96	1.69	2.04	0.467	1.36	1.29	3.12	4.48	1.04	4.2	2.39	1.63	2.87	2.58	2.71	0.977	2.63	4.59	1.65	2.73	4.1	2.33	0.344	3.51	0.694
1-Methylnicotinamide_Rest	0	0.0723	0.029	0	0.0194	0.0234	0.0236	0.0311	0.0308	0.0347	0.0284	0	0.03	0.0256	0.0567	0	0.227	0	0.0391	0	0.0155	0	0.0396	0	0.0349	0.0391	0	0.0205	0.026	0	0.0207	0.0148
p-Aminobenzoate_Rest	0	0	0	0	0	0	0	0	0	0	0	0	0	0	0	0	0	0	0	0	0	0	0	0	0	0	0	0	0	0	0	0
Trigonelline_Rest	1.06	0.122	0.0574	0	0.226	0.0651	0.0669	0	0	0	0	0.111	0.0408	0	0.529	0	0	0	0.0316	0.032	0.0905	0.383	0	0	0	0.484	0	0	0	0.0464	0	0
Anthranilate_Rest	0	0	0	0	0	0	0	0	0	0	0	0	0	0	0	0	0	0	0	0	0	0	0	0	0	0	0	0	0	0	0	0
Phenylethanolamine_Rest	0	0	0	0	0	0	0	0	0	0	0	0	0	0	0	0	0	0	0	0	0	0	0	0	0	0	0	0	0	0	0	0
Tyramine_Rest	0	0.0494	0	0	0.0298	0	0	0	0	0	0.0343	0	0	0	0	0	0	0.139	0	0	0	0	0	0	0	0	0	0	0.0511	0	0	0
Urocanate_Rest	0.263	0.489	0.205	0.21	0.786	0.429	0.321	0.147	0.148	0.293	0.353	0.175	0.238	0.302	0.435	0.215	0.526	0.77	0.468	0.202	0.541	0.405	0.334	0.269	0.385	0.619	0	0.127	0.268	0.362	0.167	0.284
6-Hydroxynicotinate_Rest	0	0	0	0	0	0	0	0	0	0	0	0	0	0	0	0	0	0	0	0	0	0	0	0	0	0	0	0	0	0	0	0
Tropinone_Rest	0	0	0	0	0	0	0	0	0	0	0	0	0	0	0	0	0	0	0	0	0	0	0	0	0	0	0	0	0	0	0	0
Histidinol_Rest	0	0	0	0	0	0	0	0	0	0	0	0	0.00695	0	0	0	0	0	0	0	0	0	0	0	0	0	0	0	0	0	0	0
Tropine_Rest	0	0	0	0	0	0	0	0	0	0	0	0	0	0	0	0	0	0	0	0	0	0	0	0	0	0	0	0	0	0	0	0
Ectoine_Rest	0	0	0	0	0	0	0	0	0	0	0	0	0	0	0	0	0	0	0	0	0	0	0	0	0	0	0	0	0	0	0	0
4-Methyl-5-thiazoleethanol_Rest	0	0	0	0	0	0	0	0	0	0	0	0	0	0	0	0	0	0	0	0	0	0	0	0	0	0	0	0	0	0	0	0
Proline betaine_Rest	0.0539	0	0.0835	0.0355	0	0.145	0.0343	0	0	0.0561	0.0196	0	0	0.0173	0.399	0.0384	0.0315	0	0.0765	0.0247	0	0	0.0183	0	0.0884	0.017	0.0327	0.0369	0	0.0334	0.0286	0
N^1,N^{12}-Diacetylspermine_Rest	0	0.0282	0.0255	0	0.0198	0.0186	0.0309	0.0142	0.035	0.0335	0.0142	0.0177	0	0.0408	0	0	0.0173	0.0259	0.00875	0.00982	0.107	0.0273	0.0193	0.0187	0.0234	0.0223	0.0351	0	0.017	0	0.0137	0
γ-Guanidinobutyrate_Rest	0	0.0238	0	0	0	0	0	0	0	0	0	0	0	0	0	0	0	0.0225	0	0	0	0	0	0	0.0134	0.0212	0	0	0	0.0147	0	0
Acetylcholine_Rest	0	0	0	0	0	0	0	0	0	0	0	0	0	0	0	0	0	0	0	0	0	0	0	0	0	0	0	0	0	0	0	0
γ-Butyrobetaine_Rest	0.162	1.95	0.68	0.131	0.249	1.03	0.827	0.517	0.556	1.23	0.446	0.426	0.313	0.349	0.317	0.28	0.558	0.405	0.742	0.444	0.725	0.441	0.673	0.209	0.693	0.669	0.457	0.11	0.82	0.177	0.347	0.0494
Lysinamide_Rest	0	0	0	0	0	0.0652	0	0	0	0	0	0	0	0	0	0	0	0	0	0	0	0	0	0	0	0	0	0	0	0	0	0
Spermidine_Rest	0.0679	0.512	0.217	0.0522	0.155	0.622	0.433	0.284	0.626	0.708	0.141	0.197	0.12	0.196	0.344	0.163	0.284	0.375	0.283	0.0844	0.971	0.216	0.237	0.272	0.285	0.242	0.397	0.294	0.384	0.184	0.188	0.0363
Gln_Rest	1.72	5.3	6.72	1.64	4.22	6.66	4.11	8.08	3.82	7.13	1.67	2.11	3.67	3.82	14.3	2.45	8.46	3.65	2.91	2.69	6.73	7.59	2.71	3.91	6.6	5.09	5.09	6.68	3.21	2.47	6.53	2.08
5,6-Dimethylbenzimidazole_Rest	0	0	0	0	0	0	0	0	0	0	0	0	0	0	0	0	0	0	0	0	0	0	0	0	0	0	0	0	0	0	0	0
Lys_Rest	1.85	4.44	6.68	0.531	6.82	2.31	6.75	3.66	5.43	11.6	1.2	1.66	1.68	3.35	6.52	3.34	3.92	5.37	3.53	1.93	13.2	9.62	7.25	2.67	3.59	8.04	2.29	3.49	4.37	10	2	1.17
Trientine_Rest	0	0	0	0	0	0	0	0	0	0	0	0	0	0	0	0	0	0	0	0	0	0	0	0	0	0	0	0	0	0	0	0
Glu_Rest	3.59	8.04	13.6	3.81	7.16	12	10.5	11.7	7.11	16.2	3.66	7.08	4.94	8.26	33.4	5.31	12.9	8.46	5.73	7.03	16.8	12	6.59	7.24	8.59	9.82	10	7.37	7.42	10.5	10.9	2.19
O-Acetylserine_Rest	0	0.105	0.099	0	0.178	0	0.148	0.0327	0	0.173	0	0	0	0	0	0	0.0455	0.0251	0	0	0.219	0.169	0	0	0	0	0	0	0	0.175	0.0841	0
Nornicotine_Rest	0	0	0	0	0	0	0	0	0	0	0	0	0	0	0	0	0	0	0	0	0	0	0	0	0	0	0	0	0	0	0	0
Met_Rest	0	0.333	0.11	0.0385	0.0596	0.0607	0.0499	0.593	0	0.159	0	0	0.314	0.119	0.27	0	0.277	0.175	0.235	0	0.705	0.212	0.0771	0	0.153	0.117	0.196	0.445	0.196	0	0.622	0.116
3-Methyladenine_Rest	0	0	0	0	0	0	0	0	0	0	0	0	0	0	0	0	0	0	0	0	0	0	0	0	0	0	0	0	0	0	0	0
6-Methylaminopurine_Rest	0	0	0	0	0	0	0	0	0	0	0	0	0	0	0	0	0	0	0	0	0	0	0	0	0	0	0	0	0	0	0	0
Guanine_Rest	0.382	0.749	0.66	0.252	0.41	0.845	0.38	1.73	0.444	0.418	0.241	0.406	0.489	1.07	1.45	0.35	1.46	0.633	0.176	0.863	0.293	1.02	0.274	1.02	1.23	0.352	0.917	1.26	0.301	0.191	1.59	0.402
Amantadine_Rest	0	0	0	0	0	0	0	0	0	0	0	0	0	0	0	0	0	0	0	0	0	0	0	0	0	0	0	0	0	0	0	0
Xanthine_Rest	0.452	2.51	1.82	0.633	1.23	2.14	1.39	2.59	0.644	0.82	0	0.727	0.385	0.838	7.15	0.248	3.8	2.22	0.493	2.51	1.83	1.21	0.731	2.8	3.07	1.87	2.28	3.37	1.8	0	2.32	0
4-Aminosalicylate_Rest	0	0	0	0	0	0	0	0	0	0	0	0	0	0	0	0	0	0	0	0	0	0	0	0	0	0	0	0	0	0	0	0
3-Hydroxyanthranilate_Rest	0	0	0	0	0	0	0	0	0	0	0	0	0	0	0	0	0	0	0	0	0	0	0	0	0	0	0	0	0	0	0	0
Dopamine_Rest	0	0	0	0	0	0	0	0	0	0	0	0	0	0	0	0	0	0	0	0	0	0	0	0	0	0	0	0	0	0	0	0
4-(*β*-Acetylaminoethyl)imidazole_Rest	0	0.0233	0.0403	0.0139	0	0	0.0177	0	0	0.0117	0	0	0.00469	0.0121	0	0.00857	0.0138	0.0157	0.0203	0	0	0	0.0121	0	0	0	0	0	0.0425	0	0.00857	0
Pseudopelletierine_Rest	0	0	0	0	0	0	0	0	0	0	0	0	0	0	0	0	0	0	0	0	0	0	0	0	0	0	0	0	0	0	0	0
His_Rest	2.09	3.11	2.94	1.39	4.32	2.17	5.09	2.29	3.12	3.85	1.11	1.91	1.1	2.62	3.56	2.39	4.05	2.62	3.04	1.46	6.03	2.77	2.39	2.37	3.15	3.48	1.4	2.39	2.17	3.18	1.34	1.13
β-Imidazolelactate_Rest	0	0	0	0	0	0	0.0571	0	0	0	0	0	0	0.0931	0	0	0	0	0	0	0	0	0.07	0.0838	0	0.0549	0	0	0	0	0	0
Ibotenate_Rest	0	0	0	0	0	0	0	0	0	0	0	0	0	0	0	0	0	0	0	0	0	0	0	0	0	0	0	0	0	0	0	0
Allantoin_Rest	20.7	36.1	10	8.92	11.8	13.1	10.6	12	13.6	16.9	14.2	15.8	11	13.8	12.1	21.9	16.6	16.3	22.6	10.3	13.7	11.8	14.3	14.1	17.6	13.9	12.8	10.4	13.2	13.9	13.2	18.2
Indole-3-acetaldehyde_Rest	0	0	0	0	0	4.68	0	0	12.2	0	0	0	0	4.01	0	22.6	0	0	0	0	0	0	0	0	0	18	4.82	15.5	0	0	8.2	0
Betonicine_Rest	0	0	0	0	0	0	0	0	0	0	0	0	0	0	0	0	0	0	0	0	0	0	0	0	0	0	0	0	0	0	0	0
N-Acetylvaline_Rest	0	0	0	0	0	0	0	0	0	0	0	0	0	0	0	0	0	0	0	0	0	0	0	0	0	0	0	0	0	0	0	0
Ala-Ala_Rest	0.0692	0.109	0.176	0.0639	0.126	0	0.155	0.164	0	0.315	0.0973	0.101	0	0.106	0.0783	0.135	0.167	0.136	0.171	0.0937	0.392	0.117	0.112	0	0	0.157	0	0	0.186	0.139	0.115	0
Tryptamine_Rest	0	0	0	0	0	0	0	0	0	0	0	0	0	0	0	0	0	0	0	0	0	0	0	0	0	0	0	0	0	0	0	0
α-Aminoadipate_Rest	0.145	0.602	0.49	0.185	0.464	0.496	0.607	0.694	0.274	0.767	0.421	0.331	0.231	0.36	0.814	0.254	0.805	0.366	0.408	0.524	0.639	0.732	0.29	0.242	0.569	0.417	0.533	0.55	0.307	0.626	0.543	0.348
N-Methylglutamate_Rest	0	0	0	0	0	0	0	0	0	0	0	0	0	0	0	0	0	0	0	0	0	0	0	0	0	0	0	0	0	0	0	0
Indole-3-ethanol_Rest	0	0	0	0	0	0	0	0	0	0	0	0	0	0	0	0	0	0	0	0	0	0	0	0	0	0	0	0	0	0	0	0
Carnitine_Rest	0.205	0.746	0.365	0.248	0.403	0.718	0.412	0.709	0.458	0.619	0.241	0.445	0.205	0.471	1.3	0.27	0.762	0.4	0.689	0.486	0.41	0.506	0.452	0.503	0.723	0.683	0.382	0.588	0.435	0.276	0.35	0.147
2-Deoxystreptamine_Rest	0	0	0	0	0	0	0	0	0	0	0	0	0	0	0	0	0	0	0	0	0	0	0	0	0	0	0	0	0	0	0	0
5-Hydroxylysine_Rest	0	0	0	0	0	0	0	0	0	0	0	0	0	0	0	0	0	0	0	0	0	0	0	0	0	0	0	0	0	0	0	0
Nicotine_Rest	0	0	0	0	0	0	0	0	0	0	0	0	0	0	0	0	0	0	0	0	0	0	0	0	0	0	0	0	0	0	0	0
Pterin_Rest	0	0	0	0	0	0	0	0	0	0	0	0	0	0	0	0	0	0	0	0	0	0	0	0	0	0	0	0	0	0	0	0
S-Methylmethionine_Rest	0	0	0	0	0	0	0	0	0	0	0	0	0	0	0	0	0	0	0	0	0	0	0	0	0	0	0	0	0	0	0	0
Methionine sulfoxide_Rest	0	0	0	0	0	0.0949	0	0.147	0.0784	0	0.03	0	0.0454	0	0.105	0	0.0852	0.282	0	0.0518	0.0888	0.0679	0.0527	0	0	0	0	0	0.0852	0.0398	0.0927	0
7-Methylguanine_Rest	0	0.0545	0.0378	0.0237	0.0323	0.0249	0.0367	0	0.0388	0.0638	0.0298	0	0.031	0	0.0388	0.0195	0.0468	0.0268	0.0426	0.0256	0.0425	0.0412	0.0267	0.0165	0.0585	0.0326	0	0.0204	0.0281	0.028	0.0357	0.0201
3-Methylguanine_Rest	0	0	0	0	0	0	0	0	0	0	0	0	0	0	0	0	0	0	0	0	0	0	0	0	0	0	0	0	0	0	0	0
Phe_Rest	2.46	2.64	6	1.44	6.57	4.29	4.73	3.37	2.87	7.6	1.68	3.57	1.66	2.25	5.75	2.37	2.83	3.08	1.87	1.27	5.78	5.07	4.9	2.89	1.92	3.07	1.34	2.52	3.44	2.5	4.81	1.08
Taurocyamine_Rest	0	0	0	0	0	0	0	0	0	0	0	0	0	0	0	0	0	0	0	0	0	0	0	0	0	0	0	0	0	0	0	0
Pyridoxal_Rest	0	0	0	0	0	0	0	0	0	0	0	0	0	0	0	0	0	0	0	0	0	0	0	0	0	0	0	0	0	0	0	0

3-Methoxytyramine_Rest	0	0	0	0	0	0	0	0	0	0	0	0	0	0	0	0	0	0	0	0	0	0	0	0	0	0	0	0	0	0	0	0
Synephrine_Rest	0	0	0	0	0	0	0	0	0	0	0	0	0	0	0	0	0	0	0	0	0	0	0	0	0	0	0	0	0	0	0	0
Pyridoxamine_Rest	0	0	0	0	0	0	0	0	0	0	0	0	0	0	0	0	0	0	0	0	0	0	0	0	0	0	0	0	0	0	0	0
Pyridoxine_Rest	0	0	0	0	0	0	0	0	0	0	0	0	0	0	0	0	0	0	0	0	0	0	0	0	0	0	0	0	0	0	0	0
Noradrenaline_Rest	0	0	0	0	0	0	0	0	0	0	0	0	0	0	0	0	0	0	0	0	0	0	0	0	0	0	0	0	0	0	0	0
3-Methylhistidine_Rest	0.0466	0.0637	0.13	0.0205	0.0654	0.0696	0.231	0.0548	0.0575	0.0839	0.0372	0.0773	0.122	0.0446	0.397	0.0485	0.378	0.0619	0.193	0.126	0.0381	0.0405	0.0192	0.0628	0.0827	0.0374	0.0625	0.0592	0.143	0.0387	0.0312	0
Indole-3-acetamide_Rest	0	0	0	0.328	0.511	0	1.76	0.698	0	0	0	0	0	1.2	0	0	1.92	0	0	0	0	0	0	0.587	0	0	0	0	1.91	1.01	0	0
N-Acetylornithine_Rest	0	0.0361	0.0364	0.0161	0	0.0478	0.058	0.0343	0.0243	0.0819	0	0.0374	0.0251	0.0404	0	0	0	0.059	0	0	0.061	0	0	0	0	0	0	0	0.0507	0	0.0452	0
N-γ-Ethylglutamine_Rest	0	0.0871	0.171	0	0	0	0	0.0574	0.04	0	0	0	0.0571	0	0	0.0727	0	0.0541	0.0186	0	0.109	0.0738	0	0	0	0	0	0.031	0	0.0763	0.0714	0.0366
Arg_Rest	2.18	5.15	4.49	1.01	3.59	3.17	5.33	3.71	5.78	10.8	1.78	4.55	1.65	5.51	6.02	2.82	7.72	6.33	5.16	2.74	12.4	4.15	5.69	4.75	4	8.93	2.59	7.53	5.28	5.83	4.35	1.89
Gramine_Rest	0	0	0	0	0	0	0	0	0	0	0	0	0	0	0	0	0	0	0	0	0	0	0	0	0	0	0	0	0	0	0	0
N-ω-Methyltryptamine_Rest	0	0	0	0	0	0	0	0	0	0	0	0	0	0	0	0	0	0	0	0	0	0	0	0	0	0	0	0	0	0	0	0
Guanidinosuccinate_Rest	0	0	0	0	0	0	0	0	0	0	0	0	0	0	0	0	0	0	0	0	0	0	0	0	0	0	0	0	0	0	0	0
Indole-3-acetate_Rest	0	2.38	0.681	0	0	0.993	4.05	1.26	1.2	1.17	0.601	0.509	0	1.05	0.422	0.816	0.842	0.935	1.32	0.78	1.42	1.31	1.05	0.697	0.668	1.31	0.87	0	1.04	0	1.99	0
2-Guanidinobenzimidazole_Rest	0	0	0	0	0	0	0	0	0	0	0	0	0	0	0	0	0	0	0	0	0	0	0	0	0	0	0	0	0	0	0	0
Citrulline_Rest	0.878	1.38	1.27	0.568	1.33	1.87	1.99	1.83	1.71	2.82	0.828	0.975	0.719	1.66	6.52	0.782	1.83	3.58	1.33	0.962	5.88	1.48	1.88	1.97	2.24	5.46	0.426	1.59	1.84	1.69	1.4	0.478
Canavanine_Rest	0	0	0	0	0	0	0	0	0	0	0	0	0	0	0	0	0	0	0	0	0	0	0	0	0	0	0	0	0	0	0	0
Serotonin_Rest	0	0	0	0	0	0	0	0	0	0	0	0	0	0	0	0	0	0	0	0	0	0	0	0	0	0	0	0	0	0	0	0
Alliin_Rest	0	0	0	0	0	0	0	0	0	0	0	0	0	0	0.455	0	0	0	0	0	0	0	0	0	0	0	0	0	0	0	0	0
Cys-Gly_Rest	0	0	0	0	0	0	0	0	0	0	0	0	0	0	0	0	0	0	0	0	0	0	0	0	0	0	0	0	0	0	0	0
Xanthopterin_Rest	0	0	0	0	0	0	0	0	0	0	0	0	0	0	0	0	0	0	0	0	0	0	0	0	0	0	0	0	0	0	0	0
Mannosamine_Rest	0	0	0	0	0	0	0	0.0873	0	0.126	0	0.0232	0	0	0.0449	0	0.108	0	0	0	0	0	0	0	0.0356	0	0.0396	0	0	0	0.0189	0
Glucosamine_Rest	0	0.0717	0.032	0	0.0527	0.0705	0.0836	0.264	0.0863	0.139	0	0	0.0733	0.132	0	0	0.389	0.0427	0.0505	0.0542	0.19	0.0754	0	0	0.341	0.142	0.0779	0.0453	0.0962	0.0537	0	0
n-Butyl a-Picolinate_Rest	0	0	0	0	0	0	0	0	0	0	0	0	0	0	0	0	0	0	0	0	0	0	0	0	0	0	0	0	0	0	0	0
Methionine sulfoximine_Rest	0	0	0	0	0	0	0	0	0	0	0	0	0	0	0	0	0	0	0	0	0	0	0	0	0	0	0	0	0	0	0	0
Tyr_Rest	2.31	4.65	6.74	1.75	12.6	3.71	8.19	3.82	6.7	6.35	1.78	4.8	2.18	3.55	9.44	3.15	4.97	4.58	2.96	2.47	7.86	9.15	6.31	3.59	2.74	6.99	2.29	3.84	4.25	6.02	3.94	1.34
Harman_Rest	0	0	0	0	0	0	0	0	0	0	0	0	0	0	0	0	0	0	0	0	0	0	0	0	0	0	0	0	0	0	0	0
Phosphorylcholine_Rest	1.1	4.81	4.46	1.15	2.99	1.39	3.83	1.68	1.7	6.06	1.86	2.32	1.22	1.52	6.03	1.09	4.74	1.62	2.48	1.76	1.92	3.81	2.62	2.17	2.02	1.81	1.45	2.02	1.24	1.94	4.45	1.05
Epinephrine_Rest	0	0	0	0	0	0	0	0	0	0	0	0	0	0	0	0	0	0	0	0	0	0	0	0	0	0	0	0	0	0	0	0
N-α,N-α-Dimethylhistidine_Rest	0	0	0	0	0	0	0	0	0	0	0	0	0	0	0	0	0	0	0	0	0	0	0	0	0	0	0	0	0	0	0	0
N^8-Acetylspermidine_Rest	0	0.0308	0.0169	0.00305	0.012	0.0135	0.0107	0	0.0182	0.0305	0.0072	0.00933	0.00667	0.0083	0.00976	0	0.0353	0	0.0175	0.00894	0.029	0	0.00671	0.0107	0.0123	0.00676	0.0123	0	0.0092	0	0	0
N^1-Acetylspermidine_Rest	0.0606	0.159	0.204	0.016	0.0891	0.219	0.102	0.123	0.211	0.188	0.0641	0.0404	0.0319	0.063	0.0415	0.0847	0.312	0.181	0.138	0.0748	0.137	0.0885	0.0661	0.0937	0.201	0.0781	0.156	0.221	0.04	0.0506	0.085	0.0515
Gly-Leu_Rest	0.0249	0.0448	0.0677	0.0147	0.0463	0.0906	0.0871	0.167	0.0473	0.169	0.0293	0.0237	0.0571	0.0648	0.0917	0.0416	0.046	0.0864	0.0794	0.0265	0.261	0.0319	0.0308	0.0752	0.103	0.0784	0.0431	0.063	0.136	0.0392	0.0811	0.0152
N-ε-Acetyllysine_Rest	0.0367	0	0.0938	0.0375	0.0676	0.0517	0.051	0	0.0585	0.126	0.033	0.0516	0	0	0.0333	0.0456	0.138	0.0578	0.0555	0	0.0906	0.0449	0.0593	0	0.0722	0.0563	0.0465	0	0.0392	0.123	0.0546	0.0386
N^6,N^6,N^6-trimethyllysine_Rest	0.0195	0.0484	0.0301	0	0.0162	0.0498	0.033	0.0226	0	0.0347	0.0281	0	0	0.0261	0.0538	0.0232	0.0541	0.03	0.043	0.0221	0.0293	0.0307	0.029	0	0.0399	0.0408	0	0	0.0362	0	0	0
Castanospermine_Rest	0	0	0	0	0	0	0	0	0	0	0	0	0	0	0	0	0	0	0	0	0	0	0	0	0	0	0	0	0	0	0	0
5-Methoxytryptamine_Rest	0	0	0	0	0	0	0	0	0	0	0	0	0	0	0	0	0	0	0	0	0	0	0	0	0	0	0	0	0	0	0	0
5-Hydroxyindoleacetate_Rest	0	0	0	0	0	0	0	0	0	0	0	0	0	0	0	0	0	0	0	0	0	0	0	0	0	0	0	0	0	0	0	0
SAH_Rest	0	0	0	0	0	0	0	0	0	0	0	0	0	0	0	0	0	0	0	0	0	0	0	0	0	0	0	0	0	0	0	0
Glucosaminate_Rest	0	0	0	0	0	0	0	0	0	0	0	0	0	0	0	0	0	0	0	0	0	0	0	0	0	0	0	0	0	0	0	0
DOPA_Rest	0	0	0	0	0	0	0	0	0	0	0	0	0	0	0	0	0	0	0	0	0	0	0	0	0	0	0	0	0	0	0	0
N-Acetylhistidine_Rest	0	0	0	0	0	0	0	0	0	0	0	0	0	0	0	0	0	0	0	0	0	0	0	0	0	0	0	0	0	0	0	0
SAM+_Rest	0	0.0367	0.0184	0	0.017	0	0.0242	0.0455	0.0294	0.0319	0	0.0164	0.0249	0.0232	0.018	0	0.053	0.0268	0	0.0251	0.0378	0.0234	0.018	0	0.0262	0	0.0365	0.0273	0	0	0.0198	0
Arginine ethyl ester_Rest	0	0	0	0	0	0	0	0	0	0	0	0	0	0	0	0	0	0	0	0	0	0	0	0	0	0	0	0	0	0	0	0
ADMA_Rest	0	0.0377	0.0176	0	0.0154	0.0259	0.0352	0	0.0121	0.02	0	0	0	0	0.0245	0	0	0.0322	0.0168	0	0.0274	0.0199	0.0139	0	0.0265	0	0.0161	0	0.0164	0	0.0193	0
SDMA_Rest	0	0.0249	0	0	0.0113	0.0237	0.0056	0	0	0.0254	0	0.0175	0	0	0.0202	0	0.0232	0	0	0.00796	0.0213	0	0.0149	0.0207	0	0.0249	0.0192	0.0241	0.00623	0	0	0
o-Acetylcarnitine_Rest	0.167	1.47	0.275	0.187	0.414	0.439	0.701	0.451	0.675	0.52	0.15	0.621	0.231	0.301	0.897	0.19	0.845	0.203	0.412	0.297	0.398	0.4	0.534	0.414	0.425	0.794	0.229	0.619	0.378	0.389	0.414	0.167
Trp_Rest	0.112	0.209	0.185	0.118	0.168	0.288	0.285	0.35	0.191	0.349	0.0827	0.0596	0.108	0.191	0.359	0.143	0.358	0.329	0.164	0.104	0.474	0.139	0.261	0.119	0.209	0.11	0.184	0.156	0.21	0	0.282	0.0772
5-Methoxyindoleacetate_Rest	1.21	1.27	0.754	0.557	0.416	1.04	0.586	1.24	0.724	0.93	0.826	1.27	0.688	0	3.04	1.22	1.3	0.929	0.986	0	0.724	0.976	0	0	0.975	0.837	1.01	0.757	0.601	1.02	0.643	0.969
Kynurenine_Rest	0	0	0	0	0	0	0	0	0	0	0	0	0	0	0	0	0	0	0	0	0	0	0	0	0	0	0	0	0	0	0	0
Cysteine-glutathione disulfide_Rest	0	0.245	0.0691	0	0	0.0921	0	0.149	0.06	0	0	0	0.0909	0.0606	0.626	0.0682	0.139	0.0711	0	0.111	0.0312	0.193	0	0.122	0.09	0	0.286	0	0	0	0.145	0
Desthiobiotin_Rest	0	0	0	0	0	0	0	0.13	0	0	0	0	0	0	0	0	0	0	0	0	0	0	0	0	0	0	0	0	0	0	0	0
o-Succinylhomoserine_Rest	0	0	0	0	0	0	0	0	0	0	0	0	0	0	0	0	0	0	0	0	0	0	0	0	0	0	0	0	0	0	0	0
trans-Zeatin_Rest	0	0	0	0	0	0	0	0	0	0	0	0	0	0	0	0	0	0	0	0	0	0	0	0	0	0	0	0	0	0	0	0
5-Hydroxytryptophan_Rest	0	0	0	0	0	0	0	0	0	0	0	0	0	0	0	0	0	0	0	0	0	0	0	0	0	0	0	0	0	0	0	0
N-Acetylglucosylamine_Rest	0	0	0	0	0	0	0	0	0	0.0684	0	0	0	0	0	0	0	0	0	0	0.296	0	0	0	0	0.0544	0	0	0	0	0	0
N-Acetylglucosamine_Rest	2.78	2.23	9.5	0.974	1.74	6.71	6.06	2.26	6.84	11.2	1.43	3.79	0.634	5.23	1.75	1.9	1.61	3.55	2.99	2.43	19.3	9.56	7.17	12	2.4	13.5	1.17	3.15	5.96	3.33	2.87	0
Cystathionine_Rest	0	0	0	0	0	0	0	0	0	0	0	0	0	0	0	0	0	0	0	0	0	0	0	0	0	0	0	0	0	0	0	0
3-Hydroxykynurenine_Rest	0	0	0	0	0	0	0	0	0	0	0	0	0	0	0	0	0	0	0	0	0	0	0	0	0	0.061	0	0	0	0	0	0
2'-Deoxycytidine_Rest	0.03	0	0	0	0	0	0.00758	0	0	0	0	0	0	0	0	0	0	0	0	0	0	0	0	0	0	0.0267	0	0	0	0	0	0
N^1,N^8-Diacetylspermidine_Rest	0	0.0524	0.0201	0	0.0138	0.0162	0.0207	0.0247	0.0365	0.0229	0.0266	0.0171	0.0212	0.0261	0.0122	0.0347	0.0231	0.0112	0	0.0131	0.0465	0.0179	0.0105	0.0137	0.0255	0.0167	0.0275	0	0.0143	0.0108	0.0137	0
γ-Glu-2AB_Rest	0	0	0	0	0	0	0	0	0	0	0	0	0	0	0	0	0	0	0	0	0	0	0	0	0	0	0	0	0	0	0	0
Melatonin_Rest	0	0	0	0	0	0	0	0.0539	0	0	0	0	0.0445	0	0	0.142	0	0.0335	0	0	0	0	0	0	0	0	0	0.0609	0	0	0	0
Sepiapterin_Rest	0	0	0	0	0	0	0	0	0	0	0	0	0	0	0	0	0	0	0	0	0	0	0	0	0	0	0	0	0	0	0	0
7,8-Dihydrobiopterin_Rest	0	0	0	0	0	0	0	0	0	0	0	0	0	0	0	0	0	0	0	0	0	0	0	0	0	0	0	0	0	0	0	0
Cystine_Rest	0	0.0953	0.0738	0	0	0.0357	0.0401	0	0.0649	0.0494	0	0	0.0336	0	0.0934	0	0.356	0	0	0	0	0	0.0352	0	0	0	0.202	0.0643	0.0447	0	0.16	0
5-Methyl-2'-deoxycytidine_Rest	0	0	0	0	0	0	0	0	0	0	0	0	0	0	0	0	0	0	0	0	0	0	0	0	0	0	0	0	0	0	0	0
Thymidine_Rest	1.37	0	0.69	0.615	0.779	0.813	0.8	1.29	0	0.807	1.18	0	0	0.801	0	1.85	0.929	1.14	0.75	0.542	0	0	0	0.738	1.04	0.806	1.03	0	0.784	0	1.05	0
Cytidine_Rest	0.0681	0	0.116	0.0528	0.0438	0.129	0.103	0	0.143	0.367	0.123	0.185	0	0.116	0	0.152	0	0.183	0.169	0.0426	0.314	0.0611	0.134	0.207	0.106	0.106	0.036	0.0501	0.228	0.143	0	0
Uridine_Rest	2.43	1.65	1.54	0.956	1.02	1.39	1.35	2.33	1.69	1.67	1.1	1.76	1.01	1.54	2.61	2.33	2.93	2.22	1.77	1.6	2.8	1.89	1.55	1.85	2.52	1.86	1.48	2.12	1.28	1.57	2.11	1.49
N^1-Acetylspermine_Rest	0	0.0289	0.0328	0	0.0462	0.0469	0.0431	0	0	0.0294	0	0	0	0	0	0	0.0154	0	0.0115	0	0	0	0	0	0.02	0.0182	0.019	0	0.0186	0	0	0
Octopine_Rest	0	0.0143	0.0128	0	0.0449	0.0612	0	0.0237	0	0	0	0	0.0232	0	0	0.0178	0	0.0787	0.00858	0	0.107	0.0074	0.0382	0	0	0.0538	0	0	0	0.0204	0	0
Pyridoxamine 5'-phosphate_Rest	0	0	0	0	0	0	0	0	0	0	0	0	0	0	0	0	0	0	0	0	0	0	0	0	0	0	0	0	0	0	0	0
γ-Glu-cys_Rest	0	0	0	0	0	0	0	0	0	0	0	0	0	0	0	0	0	0	0	0	0	0	0	0	0	0	0	0	0	0	0	0
Muramate_Rest	0	0	0	0	0	0	0	0	0	0	0	0	0	0	0	0	0	0	0	0	0	0	0	0	0	0	0	0	0	0	0	0
5'-Deoxyadenosine_Rest	0	0	0	0	0	0	0	0	0	0	0	0	0	0	0	0	0	0	0	0	0	0	0	0	0	0	0	0	0	0	0	0
Purine riboside_Rest	0	0	0	0	0	0	0	0	0	0	0	0	0	0	0	0	0	0	0	0	0	0	0	0	0	0	0	0	0	0	0	0
2'-Deoxyinosine_Rest	0	0	0	0	0	0	0	0	0	0	0	0	0	0	0	0	0	0	0	0	0	0	0	0	0	0	0	0	0	0	0	0
7,8-Dihydroneopterin_Rest	0	0	0	0	0	0	0	0	0	0	0	0	0	0	0	0	0	0	0	0	0	0	0	0	0	0	0	0	0	0	0	0
Glycerophosphorylcholine_Rest	0	1.07	0.507	0.472	1.67	0	0.304	0.727	0	0.277	0.167	0.832	1.13	0.413	4.03	0	0.599	0	0	0.844	0	1.01	0.21	0.205	0	0	0.48	0.54	0.13	0.893	1.2	0.286
Thiamine_Rest	0	0	0	0	0	0	0	0	0	0	0	0	0	0	0	0	0	0	0	0	0	0	0	0	0	0	0	0	0	0	0	0
N^6-Methyl-2'-deoxyadenosine_Rest	0	0	0	0	0	0	0	0	0	0	0	0	0	0	0	0	0	0	0	0	0	0	0	0	0	0	0	0	0	0	0	0
Adenosine_Rest	0.0792	0.194	0.0727	0.0468	0.0227	0.0358	0.0549	0.0552	0.117	0.0555	0.244	0.139	0.0415	0.0614	0.0855	0.0352	0.33	0.0419	0.0309	0.075	0.0629	0.0545	0.0573	0.0732	0.052	0.0584	0.17	0.0469	0.0319	0.123	0.0598	0.0538
2'-Deoxyguanosine_Rest	0	0	0	0	0	0	0	0	0	0	0	0	0	0	0	0	0	0	0	0	0	0	0	0	0	0	0	0	0	0	0	0
Homocystine_Rest	0	0	0	0	0	0	0	0	0	0	0	0	0	0	0	0	0	0	0	0	0	0	0	0	0	0	0	0	0	0	0	0
Inosine_Rest	0	0	0.35	0.0613	0	0.606	0.28	0.52	0.357	0.157	0	0.143	0.191	0.275	0.368	0	0.566	0	0	0	0	0.489	0.271	0.38	0.45	0.301	0.419	0	0	0.226	0.478	0
Glu-Glu_Rest	0	0.027	0.0601	0.0329	0.0408	0	0.0696	0.0611	0.0527	0.131	0	0.0535	0.0348	0	0.0644	0	0.0642	0	0.0509	0	0.111	0.0518	0.0747	0.0527	0	0.0994	0	0	0.0627	0	0.0614	0.0305
Saccharopine_Rest	0	0.048	0	0	0	0.0362	0.045	0	0	0.0455	0	0.0304	0	0	0.078	0	0.12	0	0	0	0	0.0115	0	0	0	0.05	0	0	0.0206	0	0	0
1-Methyladenosine_Rest	0	0.036	0.0109	0	0	0.0248	0.0138	0.0306	0.0239	0.0222	0	0.022	0	0.0218	0.0619	0.0127	0.0397	0.0112	0.013	0.031	0.0182	0.0229	0	0.0281	0.0219	0.0102	0	0.023	0.0122	0	0.0233	0
Guanosine_Rest	0.0682	0.378	0.16	0.062	0.0636	0.242	0.0828	0.302	0.207	0	0.165	0.2	0.0758	0.0927	0.268	0.039	0.301	0.107	0.0369	0.206	0.0579	0.277	0.101	0.238	0.175	0.116	0.265	0.151	0.0441	0.123	0.306	0.0774

Xanthosine_Rest	0	0	0	0	0	0	0	0	0	0	0	0	0	0	0	0	0	0	0	0	0	0	0	0	0	0	0	0	0	0	0	0
Ophthalmate_Rest	0	0.0293	0	0	0	0	0	0	0	0	0	0	0	0	0.0451	0	0	0	0	0	0	0	0	0	0	0	0	0	0	0	0	0
5-Methylthioadenosine_Rest	0	0	0	0	0	0	0	0	0	0	0	0	0	0	0	0	0	0	0	0	0	0	0	0	0	0	0	0	0	0	0	0
Glutathione(ox)_Rest	0.0614	0.631	0.0879	0.0354	0.0902	0	0.0825	0.582	0.109	0.0529	0.0613	0.0455	0.058	0.142	1.01	0.0604	0	0	0	0.11	0	0.313	0.0296	0.0308	0	0.0632	0.284	0	0.0288	0	0.0313	0.0427
N-*α*-Benzenolarginine ethylester_Rest	0	0	0	0	0	0	0	0	0	0	0	0	0	0	0	0	0	0	0	0	0	0	0	0	0	0	0	0	0	0	0	0
3-Iodotyrosine_Rest	0	0	0	0	0	0	0	0	0	0	0	0	0	0	0	0	0	0	0	0	0	0	0	0	0	0	0	0	0	0	0	0
Glutathione(red)_Rest	0	0	0	0	0	0	0	2.22	0	0	0	0	0	0	0.743	0	0	0	0	0	0	0	0	0	0	0	0	0	0	0	0	0
Phe-Phe_Rest	0	0	0.008	0	0	0	0.0243	0.0196	0	0.0597	0	0.0116	0	0.0182	0	0	0	0.0167	0.0281	0.00428	0.0366	0	0.0218	0.0234	0.0168	0.0192	0	0.0139	0	0	0	0
AICAR_Rest	0	0.543	0.264	0	0	0	0	0.34	0.311	0	0	0	0.3	0	0	0.506	0	0.341	0.54	0	0.75	0	0	0	0	0	0	0.427	0	0.44	0	0.348
Thiamine monophosphate_Rest	0	0	0	0	0	0	0	0	0	0	0	0	0	0	0	0	0	0	0	0	0	0	0	0	0	0	0	0	0	0	0	0
Tetrahydropalmatine_Rest	0	0	0	0	0	0	0	0	0	0	0	0	0	0	0	0	0	0	0	0	0	0	0	0	0	0	0	0	0	0	0	0
TRH_Rest	0	0	0	0	0	0	0	0	0	0	0	0	0	0	0	0	0	0	0	0	0	0	0	0	0	0	0	0	0	0	0	0
Riboflavin_Rest	0	0	0	0	0	0	0.324	0	0	0	0	0	0	0	0	0	0	0	0	0.188	0	0	0	0	0	0	0	0	0	0	0	0
S-Lactoylglutathione_Rest	0	0	0	0	0	0	0	0	0	0	0	0	0	0	0	0	0	0	0	0	0	0	0	0	0	0	0	0	0	0	0	0
Leu-Leu-Tyr_Rest	0	0	0	0	0	0	0	0	0	0	0	0	0	0	0	0	0	0	0	0	0	0	0	0	0	0	0	0	0	0	0	0
3,5-Diiodo-tyrosine_Rest	0	0	0	0	0	0	0	0	0	0	0	0	0	0	0	0	0	0	0	0	0	0	0	0	0	0	0	0	0	0	0	0
5-Methyltetrahydrofolate_Rest	0	0	0.864	0	0	0	0	0	0.685	0	0	0	0	0	0	0	0	0	0.89	0	2.62	0	0	0	0	0	0	0	0	0	0	0
3,3',5-Triiodothyronine_Rest	0	0	0	0	0	0	0	0	0	0	0	0	0	0	0	0	0	0	0	0	0	0	0	0	0	0	0	0	0	0	0	0
NMN_Rest	0	0	0	0	0	0	0	0	0	0	0	0	0	0	0.0643	0	0.125	0	0	0	0	0	0	0	0	0	0	0	0	0	0	0
Glyoxylate_Rest	0	0	0	0	0	0	0	1.33	0	0	0	0	0	0	0.663	0	0	1.23	1.12	0	0.509	0	0.416	0.447	0	0	0	0	0.417	0.565	0.943	0
Propionate_Rest	4.14	116	10.2	3.11	11.6	8.32	31.2	10.9	10.6	47.8	3.34	12.7	4.94	11.3	5.49	8.22	12.4	2.93	9.29	3.24	16	18	7.74	5.75	22	8.42	8.23	1.5	23.3	2.82	106	1.83
Glycolate_Rest	5.8	5.64	0	1.91	3.95	3.39	3.56	3.04	2.44	2.66	3.8	2.08	3.1	3.92	3.07	3.62	3.39	0	4.14	2.51	2.68	3.51	3.01	2.64	4.23	3.06	2.05	3.56	3.14	2.45	4.08	2.21
Crotonate_Rest	0	0	0	0	0	0	0	0	0	0	0	0	0	0	0	0.596	0	0	0	0	0	0	0	0	0	0	0	0	0	0	0.519	0
Pyruvate_Rest	2.43	13	2.03	1.82	3.7	5.42	3.86	1.1	6.65	4.13	2.78	2.99	2.26	2.32	3.96	4.36	2.73	4.7	4.79	1.58	9.42	3.77	1.56	4.21	7.77	3.45	1.59	4.78	4.71	3.1	3.92	1.44
Butyrate_Rest	1.1	15.5	2.15	0.702	1.06	3.4	9.95	4.38	2.54	11.5	0.888	2.87	1.1	2.34	2.32	2.66	3	0.749	2.49	1.52	2.1	2.38	1.71	2.44	3.98	3.97	1.51	0.31	6.05	1.21	20.9	0
Lactate_Rest	198	191	38.7	187	78.1	123	108	134	158	203	118	68.1	68.5	282	172	182	101	97	156	196	169	117	45	127	241	155	65	143	137	71.8	120	96.7
3-Hydroxypropionate_Rest	18.8	12.1	4.73	20.2	11.5	15.9	10.5	11.7	19.2	10.1	10.6	7.28	10.7	16.3	12.7	19.2	10.8	3.93	16	18.9	16	9.48	4.96	12.3	15.3	15.2	7.7	18.6	20.5	2.57	8.14	6.83
Methanesulfonate_Rest	0	0	0	0	0	0	0	0	0	0	0	0	0	0	0	0	0	0	0	0	0	0	0	0	0	0	0	0	0	0	0	0
Tiglate_Rest	0	0	0	0	0	0	0	0	0	0	0	0	0	0	0	0	0	0	0	0	0	0	0	0	0	0	0	0	0	0	0	0
2-Oxobutyrate_Rest	0	0	0	0	0	0	0	0	0	0	0	0	0	0	0	0	0	0	0	0	0	0	0	0	0	0	0	0	0	0	0	0
Pentanoate_Rest	0	0	0	0	0	0	0	0	0	0	0	0	0	0	0	0	0	0	0	0	0	0	0	0	0	0	0	0	0	0	0	0
3-Methylbutanoate_Rest	0	2.53	1.04	0.206	0.242	0.78	3.16	0.879	1.14	2.97	0	0.651	0	0.69	0	0.651	1.12	0	1.93	0.359	1.86	0.504	0.35	0.589	1.75	0.898	0.498	0	2.03	0	5.86	0
Malonate_Rest	0.25	0.306	0.263	0.301	0.329	0.22	0.336	0.277	0.238	0.335	0.324	0.272	0.225	0.338	0.258	0.311	0.406	0.203	0.686	0.326	0.191	0.354	0.273	0.278	0.26	0.347	0.224	0.241	0.377	0.243	0.278	0.257
2-Hydroxyisobutyrate_Rest	0	0	0	0	0	0	0	0	0	0	0	0	0	0	0	0	0	0	0	0	0	0	0	0	0	0	0	0	0	0	0	0
2-Hydroxybutyrate_Rest	0.504	0.551	0	0.372	0.221	0.638	0.881	0.418	0.408	0.453	0.319	0.26	0.385	0.936	0.733	1	2.43	0.377	0.66	0.484	0.601	0.375	0	0.654	0.586	0.349	0	0.657	1.02	0.309	0.339	0.353
3-Hydroxybutyrate_Rest	2.3	8.39	1.01	1.65	0.964	3.37	2.97	1.16	1.51	2.09	2.03	1.94	1.08	3.3	2.58	4.38	3.39	1.32	2.43	2.05	1.87	1.67	0.928	1.46	3.39	2.84	1.04	1.53	3.22	0.722	1.62	1.68
(Methylthio)acetate_Rest	0	0	0	0	0	0	0	0	0	0	0	0	0	0	0	0	0	0	0	0	0	0	0	0	0	0	0	0	0	0	0	0
Glycerate_Rest	1.21	1.23	1.03	0.204	0.64	0.88	0.95	1.6	1.06	0.582	0.35	0.407	1.27	1.1	1.69	0.592	1.17	0.719	0.676	0.723	1.54	1.23	1.07	1.08	1.21	0.874	0.91	0.452	1.05	0.507	0.392	0
Pyrrole-2-carboxylate_Rest	0	0	0	0	0	0	0	0	0	0	0	0	0	0	0	0	0	0	0	0	0	0	0	0	0	0	0	0	0	0	0	0
Methyl sulfate_Rest	0	0	0	0	0	0	0	0	0	0	0	0	0	0	0	0	0	0	0	0	0	0	0	0	0	0	0	0	0	0	0	0
2-Furoate_Rest	0	0	0	0	0	0	0	0	0	0	0	0	0	0	0	0	0	0	0	0	0	0	0	0	0	0	0	0	0	0	0	0
Fumarate_Rest	0.398	0.449	0.329	0.319	0.631	0.375	0.4	0.61	0.448	0.333	0.311	0.483	0.375	0.607	1.62	0.23	0.617	0.71	0.387	0.573	0.475	0.52	0.359	0.451	0.473	0.424	0.492	0.713	0.38	0.526	0.402	0.223
2-Oxoisopentanoate_Rest	0	0	0	0.129	0.0725	0.226	0.134	0	0	0.177	0.168	0.0978	0	0.207	0.355	0	0.283	0	0	0.092	0.451	0	0.132	0.296	0.232	0.111	0.0542	0	0.338	0	0	0
4-Oxopentanoate_Rest	0.669	0.319	0	0.315	0	0.115	0	0	0.21	0	0.408	0	0	0.368	0.0997	0.732	0	0	0.443	0.309	0.141	0.336	0	0	0.415	0.402	0	0	0.701	0	0.0737	0.426
Hexanoate_Rest	0	0	0.351	0.0956	0.104	0.508	0.647	0.54	0	1.82	0.115	0.219	0.13	0.367	0	0.547	0.36	0	0.933	0.242	0.56	0.141	0.186	0.29	0.971	0.698	0.127	0	0.939	0	2.02	0
Succinate_Rest	12.2	6.84	13.5	25.7	38.9	32.3	32.1	20.7	7.47	18.5	6.23	26.8	16.1	14.3	10.6	16.9	9.13	8.61	12.1	22.8	18.6	19.7	13.6	27	14.9	9	13.6	10.1	39.9	35.1	16.8	4.88
2-Hydroxypentanoate_Rest	0.897	0.835	0.297	0.685	0.236	0.445	0.432	0.29	0.392	0.545	0.718	0.183	0.226	0.8	0.284	1.25	1.11	0.538	0.703	0.575	0.503	0.382	0.237	0.347	0.925	0.578	0.2	0.325	1.21	0.176	0.658	0.507
Benzoate_Rest	0.614	0	0	0.0855	0	0.107	0	0	0	0	0.289	0	0	0.255	0.279	0.389	0	0	0	0.152	0	0	0	0	0.218	0.272	0	0	0.413	0	0.278	0
Nicotinate_Rest	0.191	0	0	0	0.126	0.377	0.258	0.364	0.485	0.272	0.273	0	0.205	0.408	0	0.471	0.261	0	0.22	0.125	0.397	0	0.241	0.188	0.491	0.388	0.287	0.267	0.463	0	0.214	0
1-Aminoethylphosphonate_Rest	0	0	0	0	0	0	0	0	0	0	0	0	0	0	0	0	0	0	0	0	0	0	0	0	0	0	0	0	0	0	0	0
2-Aminoethylphosphonate_Rest	0	0	0	0	0	0	0	0	0	0	0	0	0	0	0	0	0	0	0	0	0	0	0	0	0	0	0	0	0	0	0	0
Isethionate_Rest	0	0.291	0	0	0.0906	0	0.084	0.0834	0.151	0.138	0	0.0947	0	0	0.113	0	0.122	0	0	0	0.0727	0	0	0.0735	0.0739	0.093	0	0	0	0	0.141	0
Barbiturate_Rest	0	0	0	0	0	0	0	0	0	0	0	0	0	0	0	0	0	0	0	0	0	0	0	0	0	0	0	0	0	0	0	0
5-Oxoproline_Rest	1.39	1.99	2.29	1.31	4.05	2.08	2.37	1.4	1.62	2.69	1.54	0.963	1.77	1.79	2.47	1.15	2.36	7.1	2.26	1.48	2.64	2.66	1.61	1.74	2.84	3.04	1.95	2.16	1.76	1.99	1.19	1.19
Itaconate_Rest	0.161	0	0	0	0	0	0	0	0	0	0	0	0	0	0	0	0	0	0	0	0	0	0	0	0	0	0	0	0	0	0	0
Citraconate_Rest	0	0	0	0	0	0	0	0	0	0	0	0	0	0	0	0	0	0	0	0	0	0	0	0	0	0	0	0	0	0	0	0
4-Methyl-2-oxopentanoate_Rest	0.341	0.514	0.243	0.178	0.256	0.49	0.437	0.258	0.561	0.502	0.261	0.199	0.199	0.486	0.485	0.495	0.422	0.484	0.399	0.205	0.861	0.234	0.172	0.417	0.491	0.333	0	0.603	0.512	0.245	0.238	0.206
4-Oxohexanoate_Rest	0	0	0	0	0	0	0	0	0	0	0	0	0	0	0	0	0	0	0	0	0	0	0	0	0	0	0	0	0	0	0	0
4-Acetylbutyrate_Rest	0.309	0	0	0.116	0.0629	0	0	0.0714	0	0.0862	0.174	0	0	0.124	0	0.374	0.0761	0	0.145	0.123	0.0974	0	0.0671	0	0.2	0.167	0	0.0922	0.216	0	0	0.208
Heptanoate_Rest	0	0	0	0	0	0	0	0	0	0	0	0	0	0	0	0	0	0	0	0	0	0	0	0	0	0	0	0	0	0	0	0
N-acetyl-*β*-alanine_Rest	0.128	0.233	0.365	0	0.176	0.295	0.181	0.231	0	0.464	0	0	0.201	0	0.393	0	0.266	0	0	0.196	0.305	0.244	0.187	0.209	0.327	0.179	0.184	0	0	0	0.247	0
Glutarate_Rest	0	0	0	0	0	0	0	0	0	0	0	0	0	0	0	0	0	0	0	0	0	0	0	0	0	0	0	0	0	0	0	0
3-Ureidopropionate_Rest	0	0	0	0	0	0	0	0	0	0	0	0	0	0	0	0	0	0	0	0	0	0	0	0	0	0	0	0	0	0	0	0
2-Hydroxy-4-methylpentanoate_Rest	0.352	0.228	0.292	0.223	0.159	0.52	0.419	0.2	0.37	0.72	0.377	0.121	0.134	0.292	0.0652	0.777	0	0	0.258	0.177	0.624	0.198	0.258	0.218	0.486	0.247	0.126	0	0.613	0	0.427	0.166
6-Hydroxyhexanoate_Rest	0.37	0	0	0.121	0	0.197	0	0	0	0.166	0.168	0	0	0.264	0	0.418	0.164	0	0.312	0.187	0	0	0	0	0.441	0.257	0	0	0.299	0	0	0.278
Malate_Rest	2.15	1.73	0.683	1.91	3.83	1.95	2.37	3.07	2.36	1.74	1.64	3.08	2.09	3.6	10.8	1.23	3.55	3.59	1.72	4.13	2.36	2.2	1.48	3.02	2.77	2.07	2.89	2.99	2.64	2.4	1.46	0.947
Threonate_Rest	0	0	0.46	0.523	1.17	0.433	0	0.464	0.592	0	0	0.62	0	0	1.26	0	0.843	0.354	0	0	0.562	0.302	0.602	0.86	0	0.591	0.501	0	0	0.733	0.276	0
o-Hydroxybenzoate_Rest	0	0	0	0.0977	0	0	0	0	0	0	0	0	0	0	0	0	0	0	0.21	0.121	0	0	0.141	0	0	0.117	0.243	0	0	0	0	0
p-Hydroxybenzoate_Rest	0	0.743	0.138	0	0	0	0	0	0.209	0	0	0.759	0	0	0.825	0	0	0	0	0	0	0	0	0.573	0	0	0	0	0	0	0	0
m-Hydroxybenzoate_Rest	0	0	0	0	0	0	0	0	0	0	0	0	0	0	0	0	0	0	0	0	0	0	0	0	0	0	0	0	0	0	0	0
Phosphonoacetate_Rest	0	0	0	0	0	0	0	0	0	0	0	0	0	0	0	0	0	0	0	0	0	0	0	0	0	0	0	0	0	0	0	0
Carbamoylphosphate_Rest	0	0	0	0	0	0	0	0	0	0	0	0	0	0	0	0	0	0	0	0	0	0	0	0	0	0	0	0	0	0	0	0
Ethanolamine phosphate_Rest	10.5	96.2	18.3	13.2	38.1	7.74	25.6	27	16.8	5.29	10.4	21.4	22.6	14.1	64.4	12.7	51.1	10.8	13.9	14.3	9.29	14.3	18.3	24.7	17.8	7.52	20.5	26.4	17.4	33.1	53.9	8.09
2-Thiopheneacetate_Rest	0	0	0	0	0	0	0	0	0	0	0	0	0	0	0	0	0	0	0	0	0	0	0	0	0	0	0	0	0	0	0	0
Octanoate_Rest	0	0	0	0	0	0	0	0	0	0	0	0	0	0	0	0	0	0	0	0	0	0	0	0	0	0	0	0	0	0	0	0
2-Oxoglutarate_Rest	0.441	0.873	0	0.31	0.659	0.416	0.681	0	0.833	0.574	0	1.03	0	0	0	0	0	0.663	0	0.442	1.55	0	0.354	0.719	0	0	0	0	0.694	2.02	0	0
Adipate_Rest	0	0	0	0	0	0	0	0	0	0	0	0	0	0	0	0	0	0	0	0	0	0	0	0	0	0	0	0	0	0	0	0
threo-*β*-methylaspartate_Rest	0	0	0	0	0	0	0	0	0	0	0	0	0	0	0	0	0	0	0	0	0	0	0	0	0	0	0	0	0	0	0	0
4-Methylthio-2-oxobutyrate_Rest	0	0	0	0	0	0	0	0	0	0	0	0	0	0	0	0	0	0	0	0	0	0	0	0	0	0	0	0	0	0	0	0
2-Hydroxyglutarate + Citramalate_Rest	0.383	0	0.607	0.47	0.662	0.832	0.634	0.306	0.355	1.2	0.318	0.426	0.283	0.621	0	0.529	0	0.541	0.619	0.247	0.886	0.663	0.43	0	0.664	0.671	0.215	0.235	0.58	0.512	0	0.275
trans-Cinnamate_Rest	0	0.375	0.666	0.263	0.403	0.606	0.513	0.395	0.406	0.873	0.241	0.5	0.253	0.534	1.23	0.398	0.697	0.4	0.401	0.327	0.66	0.521	0.336	0.468	0.536	0.487	0.421	0.291	0.52	0.459	0.443	0.106
Tartrate_Rest	0	0	0	0	0	0	0	0	0	0	0	0	0	0	0	0	0	0	0	0	0	0	0	0	0	0	0	0	0	0	0	0
Benzoylformate_Rest	0	0	0	0	0	0	0	0	0	0	0	0	0	0	0	0	0	0	0	0	0	0	0	0	0	0	0	0	0	0	0	0
3-Phenylpropionate_Rest	4.06	0.474	0.804	1.18	0.3	1.33	0.866	1.07	0.698	3.52	1.61	0.595	0.456	2.47	0.544	6.88	0.878	1.39	2.62	2.45	1.59	0.523	0.485	0.807	3.76	3.63	0.429	0.554	4.69	0.423	1.92	1.31
2-Hydroxyphenylacetate_Rest	0	0	0	0	0	0	0	0	0	0	0	0	0	0	0	0	0	0	0	0	0	0	0	0	0	0	0	0	0	0	0	0
p-Hydroxyphenylacetate_Rest	0.464	11.1	1.7	0	1.47	0.615	13	3.11	5.35	0.64	0	0.695	0.927	3.25	0	1.8	0.725	1.27	2.95	1.06	1.6	4.84	5.31	0	1.02	4.49	3.55	0	2.6	0	2.18	0
Cysteine sulfinate_Rest	0	0	0	0	0.272	0.293	0.36	0	0.328	0.255	0	0	0	0.228	0	0	0	0	0	0.412	0.423	0	0	0	0.449	0.299	0.284	0	0.243	0.232	0	0
2,5-Dihydroxybenzoate_Rest	0.753	0	0.609	0	0	0	0	0	0	0	0.313	0	0	0	0	0	0.325	0	0	0	0.792	0.779	0.809	0	0	0.245	0	0	0	0.784	0	0
Orotate_Rest	0	0	0	0	0	0	0	0	0	0	0	0	0	0	0	0	0	0	0	0	0.669	0	0	0	0	0	0	0	0	0	0	0
2,4-Dihydroxypyrimidine-5-carboxylate_Rest	0	0	0	0	0	0	0	0	0	0	0	0	0	0	0	0	0	0	0	0	0	0	0	0	0	0	0	0	0	0	0	0
Dihydroorotate_Rest	0	0	0	0	0	0	0	0	0	0	0	0	0	0	0	0	0	0	0	0	0	0	0	0	0	0	0	0	0	0	0	0
2-Oxooctanoate_Rest	0	0	0	0	0	0	0	0	0	0	0	0	0	0	0	0	0	0	0	0	0	0	0	0	0	0	0	0	0	0	0	0

Pelargonate_Rest	0	0	0	0	0	0	0	0	0	0	0	0	0	0	0	0.163	0	0	0	0	0	0	0	0	0	0.0374	0.042	0	0	0	0	0
2-Oxoadipate_Rest	0	0	0	0	0	0	0	0	0	0	0	0	0	0	0	0	0	0	0	0	0.47	0	0	0	0	0	0	0	0	0	0	0
Pimelate_Rest	0	0	0	0	0	0	0	0	0	0	0	0	0	0	0	0	0	0	0	0	0	0	0	0	0	0	0	0	0	0	0	0
2-Hydroxyoctanoate_Rest	0	0	0	0	0	0	0	0	0	0	0	0	0	0	0	0	0	0	0	0	0	0	0	0	0	0	0	0	0	0	0	0
N-Formylaspartate_Rest	0	0	0	0	0	0	0	0	0	0	0	0	0	0	0	0	0	0	0	0	0	0	0	0	0	0	0	0	0	0	0	0
3-Hydroxy-3-methylglutarate_Rest	0.0636	0.0472	0.041	0	0	0.0518	0.0316	0.0664	0.0277	0.0478	0.0395	0.0342	0	0	0.0251	0.0945	0.0207	0	0.136	0.0623	0.0489	0.0866	0.0397	0.0311	0.0872	0	0	0.0498	0	0.0523	0	0
Phenylpyruvate_Rest	0	0	0	0	0	0	0	0	0	0	0	0	0	0	0	0	0	0	0	0	0.258	0	0	0	0	0	0	0	0	0	0	0
p-Coumarate_Rest	0	0	0	0	0	0	0	0	0	0	0	0	0	0	0	0	0	0	0	0	0	0	0	0	0	0	0	0	0	0	0	0
o-Coumarate_Rest	0	0	0	0	0	0	0	0	0	0	0	0	0	0	0	0	0	0	0	0	0	0	0	0	0	0	0	0	0	0	0	0
Terephthalate_Rest	0	0.103	0	0	0	0	0	0	0	0	0	0	0.12	0	0	0	0	0	0	0	0	0	0	0	0	0	0	0	0	0	0	0.113
Phthalate_Rest	0.169	0.0829	0.124	0.165	0.0951	0.12	0.0806	0.126	0.128	0.135	0.109	0.0644	0.0857	0.102	0.052	0.18	0.0517	0.0966	0.177	0.148	0.0979	0.121	0.124	0.136	0.148	0.15	0.0723	0.0771	0.149	0.161	0.0854	0.101
3-Phenyllactate_Rest	0.232	0	0.406	0.216	0.263	0.505	0.336	0.199	0.215	0.784	0.314	0.221	0.132	0.481	0	0.354	0.1	0.375	0.4	0.114	0.507	0.255	0.349	0.307	0.348	0.27	0.135	0.114	0.362	0.222	0	0
3-(2-Hydroxyphenyl)propionate_Rest	0	0	0	0	0	0	0	0	0	0	0	0	0	0	0	0	0.0326	0	0	0	0	0	0	0	0	0	0	0	0	0	0	0
3-(4-Hydroxyphenyl)propionate_Rest	1.11	5.48	2.56	0.524	0.599	0.883	8.84	2.74	2.42	3.02	2.16	1.12	1.12	1.72	0	2.43	0.591	1.64	1.84	1.6	2.37	2.04	2.45	0.99	2.45	2.09	2.15	0.12	1.8	0.308	4.7	0
2,3-Pyridinedicarboxylate_Rest	0	0	0	0	0	0	0	0	0	0	0	0	0	0	0	0	0	0	0	0	0	0	0	0	0	0	0	0	0	0	0	0
PEP_Rest	0.181	0.493	0	0.162	0.173	0.263	0.252	1.54	0.224	0.225	0.384	0.267	0.208	0.39	2.84	0	1.32	0.218	0.163	0.298	0.263	0.372	0.138	0.311	0.378	0.255	0.358	0.772	0	0.132	0.391	0.23
Urate_Rest	37.8	103	88.8	29	90.5	70.8	73.5	28	59.1	59.3	74.7	93.9	27.2	41.8	228	21.4	179	50.3	43.3	49.3	57.3	50.4	54.5	59.6	51.3	39.8	56.4	54.6	91.2	100	77.9	23.6
4-Hydroxymandelate_Rest	0.301	4.32	1.4	0	0.965	0.722	6.83	1.53	2.31	0.819	0.785	0.61	0.545	1.2	0.474	0.886	0.834	0	1.84	0.899	1.21	2.32	2.84	0.637	0.774	3.34	1.84	0	1.59	0	1.41	0
Cysteate_Rest	0	0	0	0	0	0	0	0	0	0	0	0	0	0	0	0	0	0.151	0.137	0	0.188	0	0	0	0	0	0	0	0	0	0	0
2-Amino-3-phosphonopropionate_Rest	0	0	0	0	0	0	0	0	0	0	0	0	0	0	0	0	0	0	0	0	0	0	0	0	0	0	0	0	0	0	0	0
DHAP_Rest	0	2.14	0	0.137	0	0.177	0	0.362	0.191	0.264	0.168	0	0.229	0.391	0	0	0.717	0.16	0	0.204	0.495	0	0	0.253	0.452	0	0.114	0.475	0.25	0	3.69	0
G3P_Rest	0	0	0	0	0	0	0	0	0	0	0	0	0	0	0	0	0	0	0	0	0	0	0	0	0	0	0	0	0	0	0	0
Glycerophosphate_Rest	1.94	8.05	3.38	2.65	4.86	4.29	5.34	4.19	3.54	8.47	3.04	4.68	3.45	4.99	10.9	2.63	20.6	5.14	4.87	3.96	8.39	4.01	3.99	5.88	4.9	4.79	3.85	7.01	4.54	5.47	7.96	1.92
Decanoate_Rest	0	0	0	0	0	0	0	0	0	0	0	0	0	0	0	0	0	0	0	0	0	0	0	0	0	0	0	0	0	0	0	0
2-Quinolinecarboxylate_Rest	0	0	0	0	0	0	0	0	0	0	0	0	0	0	0	0	0	0	0	0	0	0	0	0	0	0	0	0	0	0	0	0
N-Acetylleucine_Rest	0	0	0	0	0.0294	0	0	0	0	0.071	0	0	0	0	0	0	0	0	0	0	0	0	0	0	0	0	0	0	0	0	0	0
Phenyl phosphate_Rest	0	0	0	0	0	0	0	0	0	0	0	0	0	0	0	0	0	0	0	0	0	0	0	0	0	0	0	0	0	0	0	0
trans-Aconitate_Rest	0	0	0	0	0	0	0	0	0	0	0	0	0	0	0.0887	0	0	0	0	0	0	0	0	0	0	0	0	0	0	0	0	0
cis-Aconitate_Rest	0.0893	0.169	0.0643	0.0643	0.239	0.106	0.146	0.0286	0.129	0.0314	0.0668	0.167	0.0634	0.111	1.01	0.0809	0.146	0.0576	0.0656	0.0586	0.04	0	0.102	0.12	0.0554	0.0924	0.0181	0.0811	0.0918	0.198	0.0608	0
Shikimate_Rest	0	0	0	0	0	0.353	0	0	0	0	0	0	0	0	0	0	0	0	0	0	0.422	0.316	0	0	0	0	0	0	0	0.171	0	0
N-Acetylaspartate_Rest	0.126	0.135	0.951	0.2	2.44	0.236	1.08	0.226	0.467	0.416	0.0797	0.63	0.136	0.205	2.7	0.258	0.45	0.375	0.269	0.477	0.349	0.172	0.619	0.742	0.215	1.89	0.114	0.988	0.359	2.21	0.281	0.106
Carbamoylaspartate_Rest	0	0	0	0	0	0	0.123	0	0	0.207	0	0	0	0	0	0	0	0.1	0.196	0	0.264	0	0	0	0	0	0	0	0	0	0	0
Allantoate_Rest	0	0	0	0	0	0	0	0	0	0	0	0	0	0	0	0	0	0	0	0	0.33	0	0	0	0	0	0	0	0	0	0	0
2-Isopropylmalate_Rest	0.13	0.0853	0.0528	0.13	0.2	0.0414	0.0455	0.0383	0.0457	0.244	0.136	0.056	0.0819	0.0839	0.0329	0.115	0.185	0.0428	0.286	0.118	0.0668	0.14	0.0646	0.0897	0.251	0.185	0.0771	0.0469	0.0752	0.0827	0.0552	0
N-Formylmethionine_Rest	0	0	0	0	0	0	0	0	0	0	0	0	0	0	0	0	0	0	0	0	0	0	0	0	0	0	0	0	0	0	0	0
Hippurate_Rest	0	0	0	0	0	0	0	0	0	0	0	0	0	0	0	0	0	0	0	0	0	0	0	0	0	0	0	0	0	0	0	0
Homovanillate_Rest	0.457	0.777	0.945	0.63	0.936	0.838	1.02	0.326	0.507	1.85	0.564	0.605	0.511	0.6	0	0.558	0.355	0.576	0.891	0	0.757	0.838	1.1	0.555	1.01	0.997	0	0.299	0.608	0.64	0	0
4-Pyridoxate_Rest	0	0	0	0	0	0	0	0	0	0	0	0	0	0	0	0	0	0	0	0	0	0	0	0	0	0	0	0	0	0	0	0
Serine O-sulfate_Rest	0	0	0	0	0	0	0	0	0	0	0	0	0	0	0	0	0	0	0	0	0	0	0	0	0	0	0	0	0	0	0	0
O-Phosphoserine_Rest	0	0	0	0	0.0693	0.087	0.125	0	0	0.17	0	0	0	0	0	0	0	0	0.102	0.0658	0.242	0	0.0809	0.0946	0.0627	0.119	0	0	0.0944	0.119	0	0
3PG + 2PG_Rest	0.516	2.08	0	0.421	0.69	0.768	0.796	6.01	1.27	0.45	1.21	1.02	0.753	1.14	12.6	0.442	4.15	0.744	0.399	0.911	1.2	1.36	0.417	1.17	1.17	0.747	1.27	3.07	0.39	0.316	2.28	0.848
Undecanoate_Rest	0	0	0	0	0	0	0	0	0	0	0	0	0	0	0	0	0	0	0	0	0	0	0	0	0	0	0	0	0	0	0	0
Azelate_Rest	0.161	0.161	0.138	0.115	0.152	0.085	0.141	0.0733	0.117	0.0989	0.11	0.0753	0.143	0.113	0.188	0.131	0.132	0.191	0.149	0.114	0.0999	0.103	0.156	0.118	0.166	0.138	0.133	0.218	0.0848	0.111	0.0867	0.165
10-Hydroxydecanoate_Rest	0	0	0	0	0	0	0	0	0	0	0	0	0	0	0	0	0	0	0	0	0	0	0	0	0	0	0	0	0	0	0	0
N-Acetylglutamate_Rest	0	0.0871	0.0829	0	0.123	0.0611	0.0813	0.0465	0	0.128	0.0479	0.0667	0	0	0.114	0	0.0713	0.176	0.122	0.0609	0.0782	0.0838	0.0592	0.0545	0.0658	0.0892	0	0.0575	0.0586	0.0934	0	0
N-Carbamylglutamate_Rest	0	0	0	0	0	0	0	0	0	0	0	0	0	0	0	0	0	0	0	0	0	0	0	0	0	0	0	0	0	0	0	0
N-Acetylmethionine_Rest	0	0	0	0	0	0	0	0	0	0	0	0	0	0	0	0	0	0	0	0	0	0	0	0	0	0	0	0	0	0	0	0
Isocitrate_Rest	0.186	0.224	0	0	0.354	0.177	0.245	0	0.321	0	0.166	0.207	0	0.192	1.68	0.203	0.3	0.145	0.163	0.211	0	0	0.188	0.444	0.162	0	0	0.266	0.274	0.214	0	0
Citrate_Rest	2.09	2.58	0.922	1.04	4.98	2.01	3.13	0.656	3.9	0.669	0.902	3.2	0.877	4.49	23.4	2.18	3.59	0.843	0.672	2.03	0.588	0.582	2.02	2.11	1.11	3.57	0.377	2.94	3.47	3.73	0.808	0.596
Quinate_Rest	2.22	0	0	0	0.497	0.297	0	0	0	0	0	0	0.116	1.76	0	0.379	0.264	0.677	0.505	0	0	0	0	0	0	0.739	0	0.107	0	0.462	0	0
Phenaceturate_Rest	0	0	0	0	0	0	0	0	0	0	0	0	0	0.108	0	0	0	0	0	0	0	0	0	0	0	0.0734	0	0	0	0	0	0
Glucuronate_Rest	0	0	0	0	0	0.0594	0	0	0	0	0	0	0	0	0.409	0	0	0	0	0	0	0	0	0	0	0.0853	0	0	0	0	0	0
trans-4-Hydroxy-3-methoxycinnamate_Rest	0	0	0	0	0	0	0	0	0	0	0	0	0	0	0	0	0	0	0	0	0	0	0	0	0	0	0	0	0	0	0	0
o-Hydroxyhippurate_Rest	0	0	0	0	0	0	0	0	0	0	0	0	0	0	0	0	0	0	0	0	0	0	0	0	0	0	0	0	0	0	0	0
Gluconate_Rest	0	0	0	0	0	0	0	0	0	0	0	0	0	0	0	0	0	0	0	0	0	0	0	0	0	0	0	0	0	0	0	0
Syringate_Rest	0.436	0	0.318	0.575	0	0.474	0	0.372	0.403	0	0.412	0.521	0.387	0.508	0	0.448	0	0.435	0	0.486	0.447	0.351	0	0	0.558	0.428	0	0	0.497	0	0	0.481
4-Hydroxy-3-methoxymandelate_Rest	0	0	0	0.261	0.388	0.369	0	0	0	0.543	0	0	0	0.253	0	0	0	0	0	0	0.291	0.276	0.428	0.225	0	0.427	0	0	0.359	0	0	0
Dodecanoate_Rest	0	0	0	0	0	0	0	0	0	0	0	0	0	0	0	0	0	0	0	0	0	0	0	0	0	0	0	0	0	0	0	0
Cysteine S-sulfate_Rest	0	0	0	0	0	0	0	0	0	0	0	0	0	0	0	0	0	0	0	0	0	0	0	0	0	0	0	0	0	0	0	0
Sebacate_Rest	0	0.0211	0	0.0162	0.0171	0	0.0114	0	0	0	0	0	0	0	0	0	0.0191	0.0264	0	0	0	0	0	0	0.0267	0.0145	0.0129	0	0.0109	0.0213	0	0
3-Indolebutyrate_Rest	0	0	0	0	0	0	0	0	0	0	0	0	0	0	0	0	0	0	0	0	0	0	0	0	0	0	0	0	0	0	0	0
Xanthurenate_Rest	0	0	0	0	0	0	0	0	0	0	0	0	0	0	0	0	0	0	0	0	0	0	0	0	0	0	0	0	0	0	0	0
N-Acetylphenylalanine_Rest	0	0	0	0	0	0	0	0	0	0	0	0	0	0	0	0	0	0	0	0	0	0	0	0	0	0	0	0	0	0	0	0
Benzylsuccinate_Rest	0	0	0	0	0	0	0	0	0	0	0	0	0	0	0	0	0	0	0	0	0	0	0	0	0	0	0	0	0	0	0	0
Saccharate_Rest	0	0	0	0	0	0	0	0	0	0	0	0	0	0	0	0	0	0	0	0	0	0	0	0	0	0	0	0	0	0	0	0
Mucate_Rest	0	0	0	0	0	0	0	0	0	0	0	0	0	0.186	0.0584	0	0	0	0	0	0	0	0	0	0.138	0.0792	0	0	0	0	0	0
3-Indoxyl sulfate_Rest	0	0	0	0	0	0	0	0	0	0	0	0	0	0	0	0	0	0	0	0	0	0	0	0	0	0	0	0	0	0	0	0
2-Deoxyribose 1-phosphate_Rest	0	0	0	0	0	0	0	0	0	0	0	0	0	0	0	0	0	0	0	0	0	0	0	0	0	0	0	0	0	0	0	0
Pantothenate_Rest	0	0.168	0	0	0	0	0	0.085	0	0	0	0	0	0	0.226	0.0877	0	0	0	0	0	0.0876	0	0.183	0	0	0	0	0	0	0	0
Sinapate_Rest	0	0	0	0	0	0	0	0	0	0	0	0	0	0	0	0	0	0	0	0	0	0	0	0	0	0	0	0	0	0	0	0
Porphobilinogen_Rest	0	0	0	0	0	0	0	0	0	0	0	0	0	0	0	0	0	0	0	0	0	0	0	0	0	0	0	0	0	0	0	0
Ru5P_Rest	0.571	2.27	0.406	0.266	0.544	0.869	0.917	1.33	0.587	0.333	0.568	0.881	0.861	0.461	1.94	0.309	1.14	0.745	0.941	0.456	1.2	1.25	0.764	1.12	0.954	0.439	1	0.845	0.573	0.458	2.36	0.542
R5P_Rest	0	0	0	0	0	0	0	0	0	0	0	0	0	0	0	0	0	0	0	0	0	0	0	0	0	0	0	0	0	0	0.378	0
Dodecanedioate_Rest	0	0	0	0	0	0	0	0	0	0	0	0	0	0	0	0	0	0	0	0	0	0	0	0	0	0	0	0	0	0	0	0
2-Deoxyglucose 6-phosphate_Rest	0	0	0	0	0	0	0	0	0	0	0	0	0	0	0	0	0	0	0	0	0	0	0	0	0	0	0	0	0	0	0	0
Biotin_Rest	0	0	0	0	0.26	0	0.345	0	0.182	0.466	0	0.319	0	0.186	0	0	0.469	0	0.219	0.157	0.222	0	0.255	0.236	0.222	0.232	0	0	0	0	0	0
Glucosamine 6-phosphate_Rest	0	0	0	0	0	0	0	0	0	0	0	0	0	0	0	0	0	0	0	0	0	0	0	0	0	0	0	0	0	0	0	0
G1P_Rest	0.229	0.336	0.102	0.136	0.252	0.295	0.237	0.511	0.198	0.393	0.45	0.607	0.227	0.439	0.516	0.129	0.894	0.311	0.221	0.303	0.669	0.287	0.2	0.25	0.699	0.353	0.272	0.532	0.499	0.281	0.422	0.129
F6P_Rest	0.32	0.396	0.223	0.117	0.431	0.376	0.335	0.81	0.435	0.532	0.271	0.368	0.343	0.667	0.536	0.216	0.969	0.523	0.267	0.365	1.02	0.534	0.259	0.283	0.758	0.431	0.457	0.717	0.404	0.203	0.346	0.157
G6P_Rest	1.06	2.37	0.819	0.454	1.97	1.46	1.7	3.23	1.71	2.61	1.01	1.37	1.48	2.56	3.91	0.953	5.16	1.99	1.04	1.2	4.39	2.06	0.958	1.2	3.21	1.51	1.78	2.6	1.45	0.953	1.92	0.564
Sorbitol 6-phosphate_Rest	0	0	0	0	0	0	0	0	0	0	0	0	0	0	0	0	0	0	0	0	0	0	0	0	0	0	0	0	0	0	0	0
2,3-DPG_Rest	0	0	0	0	0	0	0	0	0	0	0	0	0	0	0	0	0	0	0	0	0	0	0	0	0	0	0	0	0	0	0	0
Galacturonate 1-phosphate_Rest	0	0	0.197	0.07	0.0929	0	0.0627	0.105	0.0711	0.193	0.0586	0.122	0	0.111	0.217	0	0.188	0.0756	0.0972	0	0.072	0	0	0.0879	0.255	0.0988	0.045	0.181	0.065	0.233	0.18	0
6-Phosphogluconate_Rest	0.117	0.209	0.139	0.114	0.206	0	0.153	0.0809	0.139	0.26	0.0682	0.147	0.129	0.262	0.154	0.106	0.16	0	0.116	0	0.385	0.0845	0.136	0.238	0.128	0.133	0	0.104	0.223	0.145	0.122	0
S7P_Rest	0.394	0.806	0	0	0	0.431	0	0.826	0.488	0.652	0.216	0	0.196	0.488	0.875	0	0.97	0.477	0.437	0.315	0.714	0	0	0	0.681	0.43	0.5	0.661	0.281	0.385	0	0
Argininosuccinate_Rest	0	0	0	0	0	0	0	0	0	0	0	0	0	0	0	0	0	0	0	0	0	0	0	0	0	0	0	0	0	0	0	0
N-Acetylmuramate_Rest	0	0	0.126	0	0	0	0.0876	0.0838	0	0	0	0	0	0	0	0	0	0	0.174	0	0	0	0	0	0.274	0	0	0	0	0	0	0
N-Acetylglucosamine 1-phosphate_Rest	0.102	0	0	0	0.137	0.105	0.15	0.111	0.12	0.235	0.187	0.799	0.0708	0.168	0	0.123	0.328	0	0	0.0727	0.293	0.158	0.139	0.0914	0.157	0.127	0	0	0.534	0	0	0
N-Acetylglucosamine 6-phosphate_Rest	0	0	0	0	0	0.127	0	0.216	0.0866	0.144	0.0776	0	0.095	0.113	0.317	0	0.283	0	0	0.0898	0.395	0.117	0	0	0.149	0.118	0	0.248	0.093	0	0	0
2',3'-cCMP_Rest	0	0	0	0	0	0	0	0	0	0	0	0	0	0	0	0	0	0	0	0	0	0	0	0	0	0	0	0	0	0	0	0
dCMP_Rest	0	0	0	0	0	0	0	0	0	0	0	0	0	0	0	0	0	0	0	0	0	0	0	0	0	0	0	0	0	0	0	0
dUMP_Rest	0	0	0	0	0	0	0	0	0	0	0	0	0	0	0	0	0	0	0	0	0	0	0	0	0	0	0	0	0	0	0	0

N-Acetylneuraminate_Rest	34.8	13.1	6.44	3.91	4.69	7.52	4.91	8.53	13.4	44.3	17.3	23.6	2.47	18.2	2.87	23.4	6.28	21.4	11.7	11.1	55	11	14.8	24.5	5.67	14.1	3.79	5.58	26	7.14	23.3	3.37
Ribulose 1,5-diphosphate_Rest	0	0	0	0	0	0	0	0	0	0	0	0	0	0	0	0	0	0	0	0	0	0	0	0	0	0	0	0	0	0	0	0
3',5'-Cyclic dAMP_Rest	0	0	0	0	0	0	0	0	0	0	0	0	0	0	0	0	0	0	0	0	0	0	0	0	0	0	0	0	0	0	0	0
dTMP_Rest	0	0	0	0	0	0	0	0	0	0	0	0	0	0	0	0	0	0	0	0	0	0	0	0	0	0	0	0	0	0	0	0
CMP_Rest	0	0.474	0.28	0.132	0.334	0.0974	0.181	0.483	0.32	0.0828	0.248	0.24	0.214	0.231	0.718	0	0.849	0.268	0.179	0.197	0.12	0.32	0.127	0.329	0.474	0.222	0.46	0.802	0.155	0	0.657	0.443
UMP_Rest	0.177	0.341	0.23	0.131	0.233	0	0.277	0.436	0.259	0.126	0.199	0.646	0.317	0.259	0.87	0	0.756	0.283	0.302	0.238	0.29	0.372	0.203	0.444	0.632	0.262	0.347	0.779	0.288	0.0597	0.396	0.295
cAMP_Rest	0	0	0	0	0	0	0	0	0	0	0	0	0	0	0	0	0	0	0	0	0	0	0	0	0	0	0	0	0	0	0	0
cIMP_Rest	0	0	0	0	0	0	0	0	0	0	0	0	0	0	0	0	0	0	0	0	0	0	0	0	0	0	0	0	0	0	0	0
dAMP_Rest	0	0	0	0	0	0	0	0	0	0	0	0	0	0	0	0	0	0	0	0	0	0	0	0	0	0	0	0	0	0	0	0
F1,6P_Rest	0	0.0836	0	0	0.0708	0	0.0773	0.0847	0	0.106	0	0	0	0.0532	0.106	0	0.117	0	0	0	0	0	0	0	0	0	0	0	0	0.0861	0	0
cGMP_Rest	0	0	0	0	0	0	0	0	0	0	0	0	0	0	0	0	0	0	0	0	0	0	0	0	0	0	0	0	0	0	0	0
3'-AMP_Rest	0	0.328	0	0	0.198	0	0	0.43	0	0	0	0	0.123	0	0.725	0	0.574	0	0	0	0	0.137	0	0	0	0	0.232	0.29	0	0	0.447	0.276
AMP_Rest	0.154	1.03	0.252	0.116	1.29	0	0.402	1.97	0.267	0	0.135	0	1.41	0.165	1.71	0	0.649	0	0.148	0.1	0.16	0.37	0	0.382	0.278	0.171	0	1.34	0.0919	0	0.792	0.569
IMP_Rest	0	0	0	0	0	0	0	0.307	0	0	0	0	0.148	0	0	0	0	0	0	0	0	0	0	0	0	0	0	0.23	0	0	0	0
Prostaglandin E2_Rest	0	0	0	0	0	0	0	0	0	0	0	0	0	0	0	0	0	0	0	0	0	0	0	0	0	0	0	0	0	0	0	0
Prostaglandin F2*α* _Rest	0	0	0	0	0	0	0	0	0	0	0	0	0	0	0	0	0	0	0	0	0	0	0	0	0	0	0	0	0	0	0	0
GMP_Rest	0	0	0	0	0.251	0	0	0.555	0	0	0	0	0.393	0	0	0	0	0	0	0	0	0	0	0	0	0	0	0.353	0	0	0	0.167
XMP_Rest	0	0	0	0	0	0	0	0	0	0	0	0	0	0	0	0	0	0	0	0	0	0	0	0	0	0	0	0	0	0	0	0
Orotidine 5'-monophosphate_Rest	0	0	0	0	0	0	0	0	0	0	0	0	0	0	0	0	0	0	0	0	0	0	0	0	0	0	0	0	0	0	0	0
Digalacturonate_Rest	0	0	0	0	0	0	0	0	0	0	0	0	0	0	0	0	0	0	0	0	0	0	0	0	0	0	0	0	0	0	0	0
NADPH_Rest	0	0	0	0	0	0	0	0	0	0	0	0	0	0	0	0	0	0	0	0	0	0	0	0	0	0	0	0	0	0	0	0
CoA_Rest	0	0	0	0	0	0	0	0	0	0	0	0	0	0	0	0	0	0	0	0	0	0	0	0	0	0	0	0	0	0	0	0
dCDP_Rest	0	0	0	0	0	0	0	0	0	0	0	0	0	0	0	0	0	0	0	0	0	0	0	0	0	0	0	0	0	0	0	0
dUDP_Rest	0	0	0	0	0	0	0	0	0	0	0	0	0	0	0	0	0	0	0	0	0	0	0	0	0	0	0	0	0	0	0	0
PRPP_Rest	0	0	0	0	0	0	0	0	0	0	0	0	0	0	0	0	0	0	0	0	0	0	0	0	0	0	0	0	0	0	0	0
dTDP_Rest	0	0	0	0	0	0	0	0	0	0	0	0	0	0	0	0	0	0	0	0	0	0	0	0	0	0	0	0	0	0	0	0
CDP_Rest	0	0	0	0	0	0	0	0	0	0	0	0	0	0	0	0	0	0	0	0	0	0	0	0	0	0	0	0	0	0	0	0
UDP_Rest	0	0	0	0	0	0	0	0	0	0	0	0	0	0	0	0	0	0	0	0	0	0	0	0	0	0	0	0	0	0	0	0
Acetyl CoA_Rest	0	0	0	0	0	0	0	0	0	0	0	0	0	0	0	0	0	0	0	0	0	0	0	0	0	0	0	0	0	0	0	0
Cholate_Rest	0	0	0	0	0	0	0	0	0	0	0	0	0	0	0	0	0	0	0	0	0	0	0	0	0	0	0	0	0	0	0	0
dADP_Rest	0	0	0	0	0	0	0	0	0	0	0	0	0	0	0	0	0	0	0	0	0	0	0	0	0	0	0	0	0	0	0	0
P1, P4-Di(adenosine-5') tetraphosphate_Rest	0	0	0	0	0	0	0	0	0	0	0	0	0	0	0	0	0	0	0	0	0	0	0	0	0	0	0	0	0	0	0	0
Isobutyryl CoA_Rest	0	0	0	0	0	0	0	0	0	0	0	0	0	0	0	0	0	0	0	0	0	0	0	0	0	0	0	0	0	0	0	0
Trehalose 6-phosphate_Rest	0	0	0	0	0	0	0	0.517	0.164	0	0	0	0	0.112	1.2	0	0	0	0	0.103	0.32	0	0	0	0.186	0	0	0	0.104	0	0	0
Malonyl CoA_Rest	0	0	0	0	0	0	0	0	0	0	0	0	0	0	0	0	0	0	0	0	0	0	0	0	0	0	0	0	0	0	0	0
Adenosine 5'-phosphosulfate_Rest	0	0	0	0	0	0	0	0.0864	0	0	0	0	0	0	0	0	0	0	0	0	0	0	0	0	0	0	0	0	0	0	0	0
Adenosine 3',5'-diphosphate_Rest	0	0	0	0	0	0	0	0	0	0	0	0	0	0	0	0	0	0	0	0	0	0	0	0	0	0	0	0	0	0	0	0
ADP_Rest	0	0	0	0	0	0	0	0.0669	0	0	0	0	0	0	0.0528	0	0	0	0	0	0	0	0	0	0	0	0	0	0	0	0	0
IDP_Rest	0	0	0	0	0	0	0	0	0	0	0	0	0	0	0	0	0	0	0	0	0	0	0	0	0	0	0	0	0	0	0	0
Succinyl CoA_Rest	0	0	0	0	0	0	0	0	0	0	0	0	0	0	0	0	0	0	0	0	0	0	0	0	0	0	0	0	0	0	0	0
Folate_Rest	0	0	0	0	0	0	0	0	0	0	0	0	0	0	0	0	0	0	0	0	0	0	0	0	0	0	0	0	0	0	0	0
GDP_Rest	0	0	0	0	0	0	0	0	0	0	0	0	0	0	0	0	0	0	0	0	0	0	0	0	0	0	0	0	0	0	0	0
Adenylosuccinate_Rest	0	0	0	0	0	0	0	0	0	0	0	0	0	0	0	0	0	0	0	0	0	0	0	0	0	0	0	0	0	0	0	0
Glycocholate_Rest	0	0	0	0	0	0	0	0	0	0	0	0	0	0	0	0	0	0	0	0	0	0	0	0	0	0	0	0	0	0	0	0
dCTP_Rest	0	0	0	0	0	0	0	0	0	0	0	0	0	0	0	0	0	0	0	0	0	0	0	0	0	0	0	0	0	0	0	0
dUTP_Rest	0	0	0	0	0	0	0	0	0	0	0	0	0	0	0	0	0	0	0	0	0	0	0	0	0	0	0	0	0	0	0	0
dTTP_Rest	0	0	0	0	0	0	0	0	0	0	0	0	0	0	0	0	0	0	0	0	0	0	0	0	0	0	0	0	0	0	0	0
CTP_Rest	0	0	0	0	0	0	0	0	0	0	0	0	0	0	0	0	0	0	0	0	0	0	0	0	0	0	0	0	0	0	0	0
UTP_Rest	0	0	0	0	0	0	0	0	0	0	0	0	0	0	0	0	0	0	0	0	0	0	0	0	0	0	0	0	0	0	0	0
dATP_Rest	0	0	0	0	0	0	0	0	0	0	0	0	0	0	0	0	0	0	0	0	0	0	0	0	0	0	0	0	0	0	0	0
dITP_Rest	0	0	0	0	0	0	0	0	0	0	0	0	0	0	0	0	0	0	0	0	0	0	0	0	0	0	0	0	0	0	0	0
ATP_Rest	0	0	0	0	0	0	0	0	0	0	0	0	0	0	0	0	0	0	0	0	0	0	0	0	0	0	0	0	0	0	0	0
dGTP_Rest	0	0	0	0	0	0	0	0	0	0	0	0	0	0	0	0	0	0	0	0	0	0	0	0	0	0	0	0	0	0	0	0
ITP_Rest	0	0	0	0	0	0	0	0	0	0	0	0	0	0	0	0	0	0	0	0	0	0	0	0	0	0	0	0	0	0	0	0
Taurocholate_Rest	0	0	0	0	0	0	0	0	0	0	0	0	0	0	0	0	0	0	0	0	0	0	0	0	0	0	0	0	0	0	0	0
GTP_Rest	0	0	0	0	0	0	0	0	0	0	0	0	0	0	0	0	0	0	0	0	0	0	0	0	0	0	0	0	0	0	0	0
XTP_Rest	0	0	0	0	0	0	0	0	0	0	0	0	0	0	0	0	0	0	0	0	0	0	0	0	0	0	0	0	0	0	0	0
CDP-choline_Rest	0	0	0	0	0	0	0	0	0	0	0	0	0	0	0	0	0	0	0	0	0	0	0	0	0	0	0	0	0	0	0	0
ADP-ribose_Rest	0	0	0	0	0	0	0	0	0	0	0	0	0	0	0	0	0	0	0	0	0	0	0	0	0	0	0	0	0	0	0	0
TDP-glucose_Rest	0	0	0	0	0	0	0	0	0	0	0	0	0	0	0	0	0	0	0	0	0	0	0	0	0	0	0	0	0	0	0	0
UDP-glucose_Rest	0.0457	0	0.0798	0	0	0.148	0	0.194	0.0952	0	0	0	0.0638	0	0.18	0	0	0	0	0	0	0.106	0	0	0.142	0	0	0	0	0	0	0
UDP-glucuronate_Rest	0	0	0	0	0	0	0	0	0	0	0	0	0	0	0	0	0	0	0	0	0	0	0	0	0	0	0	0.0675	0	0	0	0
ADP-glucose_Rest	0	0	0	0	0	0	0	0	0	0	0	0	0	0	0	0	0	0	0	0	0	0	0	0	0	0	0	0	0	0	0	0
GDP-mannose_Rest	0	0	0	0	0	0	0	0	0	0	0	0	0	0	0	0	0	0	0	0	0	0	0	0	0	0	0	0	0	0	0	0
UDP-*N*-acetylglucosamine_Rest	0	0	0	0	0	0.208	0	0.342	0.138	0	0	0	0.14	0	0	0	0.392	0	0	0	0.281	0	0	0	0.441	0	0	0	0	0	0	0
CMP-*N*-acetylneuraminate_Rest	0	0	0	0	0	0	0	0	0	0	0	0	0	0	0	0	0	0	0	0	0	0	0	0	0	0	0	0	0	0	0	0
NAD+_Rest	0	0	0	0	0	0	0	0	0	0	0	0	0	0	0	0	0	0	0	0	0	0	0	0	0	0	0	0	0	0	0	0
Deamido-NAD+_Rest	0	0	0	0	0	0	0	0	0	0	0	0	0	0	0	0	0	0	0	0	0	0	0	0	0	0	0	0	0	0	0	0
NADH_Rest	0	0	0	0	0	0	0	0	0	0	0	0	0	0	0	0	0	0	0	0	0	0	0	0	0	0	0	0	0	0	0	0
NADP+_Rest	0	0	0	0	0	0	0	0	0	0	0	0	0	0	0	0	0	0	0	0	0	0	0	0	0	0	0	0	0	0	0	0
FAD_Rest	0	0	0	0	0	0	0	0	0	0	0	0	0	0	0	0	0	0	0	0	0	0	0	0	0	0	0	0	0	0	0	0
Urea_Post-UK	277	336	301	239	218	199	426	162	392	916	398	1010	348	657	1780	310	1610	509	933	619	648	272	817	900	333	343	364	590	371	1050	545	153
Pyrazole_Post-UK	0	0	0	0	0	0	0	0	0	0	0	0	0	0	0	0	0	0	0	0	0	0	0	0	0	0	0	0	0	0	0	0
3-Aminopropionitrile_Post-UK	0	0	0	0	0	0	0	0	0	0	0	0	0	0	0	0	0	0	0	0	0	0	0	0	0	0	0	0	0	0	0	0
Methylguanidine_Post-UK	0	0	0	0	0	0	0	0	0	0	0	0	0	0	0	0	0	0	0	0	0	0	0	0	0	0	0	0	0	0	0	0
Isobutylamine_Post-UK	0	0	0	0	0	0	0	0	0	0	0	0	0	0	0	0	0	0	0	0	0	0	0	0	0	0	0	0	0	0	0	0
1,3-Diaminopropane_Post-UK	0.184	0	0.146	0.13	0.0613	0.188	0.145	0.114	0.0847	0.349	0.118	0.0988	0.0828	0.0751	0.0918	0.109	0	0.345	0.347	0.14	0.36	0.0952	0.101	0.433	0.257	0.419	0.0643	0.174	0.387	0.0888	0.0567	0.0641
Gly_Post-UK	5.31	10.1	18.5	12	29.5	11.7	12.2	12.2	10.6	19.6	6.11	16.8	8.57	7.79	111	11.3	11.8	8.84	7.1	7.88	22.5	23.9	13.1	17.1	14.9	41.3	6.98	7.04	9.43	103	6.71	4.25
Trimethylamine *N*-oxide_Post-UK	0	0	0.263	0.0345	0	0.0328	0	0.0517	0.064	0.0256	0.16	0.042	0.0193	0.0323	0.118	0	1.03	0	0.66	0	0.082	0.0339	0.0357	0	0.038	0.176	0	0.21	0.0268	0.0364	0.0554	0.0179
Isopropanolamine_Post-UK	0.148	1.18	0.285	0.182	0.151	0.164	0.395	0.182	0.2	0.892	0.181	0.286	0.183	0.214	0.138	0.095	0.102	0.191	0.225	0.132	0.186	0.118	0.217	0.107	0.0894	0.115	0.214	0.065	0.075	0.14	0.508	0.071
Hydroxyurea_Post-UK	0	0	0	0	0	0	0	0	0	0	0	0	0	0	0	0	0	0	0	0	0	0	0	0	0	0	0	0	0	0	0	0
Trimethylsulfonium_Post-UK	0	0	0	0	0	0	0	0	0	0	0	0	0	0	0	0	0	0	0	0	0	0	0	0	0	0	0	0	0	0	0	0
Cysteamine_Post-UK	0	0	0	0	0	0	0	0	0	0	0	0	0	0	0	0	0	0	0	0	0	0	0	0	0	0	0	0	0	0	0	0
Isoamylamine_Post-UK	0	0	0	0	0	0	0	0	0	0	0	0	0	0	0	0	0	0	0	0	0	0	0	0	0	0	0	0	0	0	0	0
Putrescine(1,4-Butanediamine)_Post-UK	9.71	21.3	21.3	9.53	19.5	14.1	22.8	11.8	12.7	44.8	1.92	13.6	6.62	8.76	21.4	18.6	0.871	8.75	8.03	6.25	19.9	15	11.8	11.6	5.51	18.4	4.92	2.11	13.8	33.7	15.8	1.07
β-Ala_Post-UK	0.298	0.981	0.475	0.411	0.673	0.268	0.844	0.362	0.252	1.12	0.727	0.339	0.875	0.406	2.12	0.262	0.855	0.459	0.668	0.436	0.374	0.342	0.246	0.486	0.469	0.56	0.485	0.348	0.699	0.334	0.526	0.248
Ala_Post-UK	3.62	7.02	4.61	9.95	6.81	8.1	5.86	9.14	4.98	14.8	6.04	7.62	4.19	6.57	24.9	4.5	7.13	6.38	5.33	5.45	7.52	9.16	3.63	9.13	7.69	12	6.91	4.4	7.67	8.27	5.88	3.95
Sarcosine_Post-UK	0.926	2.71	3.66	2.73	2.09	1.38	2.41	1.78	1.25	3.88	2.93	2.81	1.63	1.56	3.21	1.98	3.58	1.37	0.941	1.36	2.16	3.06	2.09	1.46	2.28	2.34	2.77	0.809	2.01	2.15	1.67	1.54
3-Aminopropane-1,2-diol_Post-UK	0.0632	0.529	0	0	0.0137	0	0	0	1.76	0.0496	0	0	0.0252	0	0.0537	0	0	0	0.0979	0.0213	0	0	0	0	0	0	0	0	0	0.0339	0.398	0
Aniline_Post-UK	0	0	0	0	0	0	0	0	0	0	0	0	0	0	0	0	0	0	0	0	0	0	0	0	0	0	0	0	0	0	0	0
4-Hydroxymethylimidazole_Post-UK	0	0	0	0	0	0	0	0	0	0	0	0	0	0	0	0	0	0	0	0	0	0	0	0	0	0	0	0	0	0	0	0
1-Methyl-2-pyrrolidinone_Post-UK	0	0	0	0	0	0	0	0	0	0	0	0	0	0	0	0	0	0	0	0	0	0	0	0	0	0	0	0	0	0	0	0

Cyclohexylamine_Post-UK	0	0	0	0	0	0	0	0	0	0	0	0	0	0	0	0	0	0	0	0	0	0	0	0	0	0	0	0	0	0	0	0
1-Aminocyclopropane-1-carboxylate_Post-UK	0	0	0	0	0	0	0	0	0	0	0	0	0	0	0	0	0	0	0	0	0	0	0	0	0	0	0	0	0	0	0	0
Betaine aldehyde_Post-UK	0	0	0	0	0	0	0	0	0	0	0	0	0	0	0	0	0	0	0	0	0	0	0	0	0	0	0	0	0	0	0	0
Spermine_Post-UK	0.0398	0.122	0.105	0.0635	0.236	0.0394	0.0943	0.0543	0.0784	0.112	0.034	0.0964	0.0909	0.0979	0.199	0.0321	0.0175	0.0765	0.0743	0.0524	0.136	0.108	0.0305	0.107	0.0555	0.0306	0.0443	0.144	0.0187	0.0667	0.121	0.0246
Hexylamine_Post-UK	0	0	0	0	0	0	0	0	0	0	0	0	0	0	0	0	0	0	0.0676	0	0	0	0	0	0	0	0	0	0	0	0	0
Cadaverine_Post-UK	1.18	1.14	14.9	1.17	7.53	5.19	4.95	6.19	3.38	8.1	1.4	2.37	1.88	1.99	1.23	5.9	1.11	2.11	5.21	0.919	3.09	3.27	0.666	3.92	3.81	3.7	0.914	0.848	6.57	7.82	1.79	0.897
GABA_Post-UK	0.349	0.381	0.505	0.523	0.462	0.463	0.471	0.499	0.335	0.93	0.545	0.329	1.85	0.171	0.585	0.626	0.357	0.402	0.321	0.438	0.479	0.491	0.412	0.495	0.448	0.709	0.302	0.097	0.424	1.49	0.526	0.262
3-Aminoisobutyrate_Post-UK	0	0.68	0.143	0.232	0.394	0.349	0.238	0.306	0.159	0.264	0.879	0.469	0.331	0.162	0.59	0.295	0.213	0.26	0.485	0.217	0.0963	0.225	0.437	0.276	0.544	0.654	0.144	0.249	0.215	0.242	0.416	0.101
2AB_Post-UK	0.176	0.545	0.482	0.289	0.207	0.53	0.368	0.405	0.176	0.839	0.15	0.421	0.181	0.197	0.738	0.273	0.388	0.237	0.221	0.219	0.461	0.271	0.41	0.363	0.21	0.353	0.211	0.0978	0.365	0.132	0.31	0.121
N-Methylalanine_Post-UK	0	0	0	0	0	0	0	0	0	0	0	0	0	0	0	0	0	0	0.0651	0	0	0	0	0	0	0	0	0	0	0	0	0
N,N-Dimethylglycine_Post-UK	0.303	0.296	0.0988	0.114	0.138	0.146	0.147	0.0822	0.0946	0.218	0.137	0.151	0.142	0.113	0.191	0.0944	0.187	0.0835	0.251	0	0.0928	0.0918	0.138	0.104	0.11	0.147	0.103	0.102	0.0937	0.135	0.107	0.124
Choline_Post-UK	2.03	3.76	3.15	2.92	3.29	3.12	2.91	4.08	2.06	5.05	1.95	1.93	2.68	3.26	7.7	2.75	3.35	4.06	3.07	3.73	3.92	2.25	1.64	4.39	2.96	4.52	3.47	3.78	3.9	1.46	2.3	1.33
2,3-Diaminopropionate_Post-UK	0	0	0	0	0	0	0	0	0	0	0	0	0	0	0	0	0	0	0	0	0	0	0	0	0	0	0	0	0	0	0	0
Ser_Post-UK	1.55	4.33	2.49	1.14	3.91	3.58	2.71	4.06	1.96	4.28	2.34	2.33	1.86	2.83	10.7	1.46	5.59	2.62	2.98	2.07	3.94	3.49	2.24	5.44	3.31	6.18	2.12	2.77	2.86	3.23	1.84	1.81
Diethanolamine_Post-UK	1.99	1.56	0.837	0.759	0.913	3.26	1.25	1.1	0.67	1.09	1.56	1.09	1.84	1.82	1.02	0.887	1.64	1.54	0.947	1.58	0.909	0.922	2.94	0.975	1.98	2.02	1.27	1.23	2.95	1.1	1.32	3.57
N-Methylaniline_Post-UK	0	0	0	0	0	0	0	0	0	0	0	0	0	0	0	0	0	0	0	0	0	0	0	0	0	0	0	0	0	0	0	0
β-Ala-Lys_Post-UK	0	0	0	0	0	0	0	0	0	0	0	0	0.0165	0	0.0192	0	0	0.0182	0.0393	0	0.0203	0.0125	0	0	0	0	0	0.0113	0	0.0131	0	0
Hypotaurine_Post-UK	0.269	0.435	0.388	0	0.581	0	0.319	1.85	0.199	0.512	0.4	0.197	0	0.295	2.25	0	0.262	0.405	0	0	0.443	0.569	0.293	0.578	0.372	0.712	0.235	0.304	0.38	0.557	0.196	0.365
2-Aminophenol_Post-UK	0	0	0	0	0	0	0	0	0	0	0	0	0	0	0	0	0	0	0	0	0	0	0	0	0	0	0	0	0	0	0	0
Cytosine_Post-UK	0.0622	0.0368	0.107	0.113	0.0609	0.0359	0.0656	0.0394	0.224	0.0887	0.0853	0.0657	0.0526	0.0549	0.0975	0.0391	0.11	0.036	0	0.046	0.044	0.0463	0.0291	0.0667	0	0.0659	0.0994	0.0687	0.0756	0.0414	0.0774	0.052
Histamine_Post-UK	0.306	0.175	1.08	1.22	0.16	0.0343	0.118	0.226	0.0403	0.0427	0.033	0.0302	0.0396	0.0306	0	0.0334	0.029	0.439	0.223	0.162	0.0394	0.243	0.0579	0.0374	0.0211	0.0405	0.0325	0	1.02	0.0378	0.101	0.0323
Uracil_Post-UK	0	0	1.48	1.11	0	0	0	1.89	0	0	0.673	0.928	0	0.684	4.82	0	1.79	1.4	0	1.64	0.257	1.38	0	1.2	0.754	0	2.26	1.26	0.917	0.735	0.976	0.25
Carnosine_Post-UK	0	0	0	0	0.171	0	0.0676	0.0238	0	0.0593	0.0375	0.0171	0.224	0.0218	0.29	0.0105	0.0169	0	0.0591	0.0312	0.035	0.0269	0.0245	0	0.0151	0	0.0275	0.131	0.0194	0.0296	0	0.0419
Creatinine_Post-UK	0.721	2.95	1.18	0.856	1.14	0.54	0.847	1.05	0.96	2.14	1.24	0.869	1.19	1.41	4.47	0.688	1.63	0.925	2.03	0.871	1.7	0.794	1.43	1.66	1.63	1.62	0.777	0.84	1.33	1.4	0.86	0.677
Muscimol_Post-UK	0	0	0	0	0	0	0	0	0	0	0	0	0	0	0	0	0	0	0	0	0	0	0	0	0	0	0	0	0	0	0	0
β-Cyanoalanine_Post-UK	0	0	0	0	0	0	0	0	0	0	0	0	0	0	0	0	0	0	0	0	0	0	0	0	0	0	0	0	0	0	0	0
Dihydrouracil_Post-UK	0	0	0	0	0	0	0	0	0	0	0	0	0	0	0	0	0	0	0	0	0	0	0	0	0	0	0	0	0	0	0	0
Pro_Post-UK	3.32	3.67	23.9	10.2	18.9	10	11.6	10.2	4.94	26	3.31	38.8	5.62	5.68	60.5	10.5	6.91	4.65	4.72	6.22	20.9	16.6	5.74	10.8	4.67	20.4	2.42	4.04	6.77	159	4.73	3.84
Guanidinoacetate_Post-UK	0.458	0.698	1.29	0.473	0.336	0	0	0.884	0.46	2.49	1.02	0	0.484	0.361	1.06	0.85	0.324	0.668	0.927	0.698	0	0.647	0	0	0.507	1.02	0.229	0.137	0.583	0	0.312	0
5-Aminovalerate_Post-UK	41.4	145	137	70.7	66.9	132	113	88.7	69.2	452	35.5	136	82.7	70.2	171	115	26.5	60.5	111	75.4	173	83.6	89.7	100	55.1	142	24.7	5.88	99	170	82.9	8.67
Val_Post-UK	1.65	2.26	5.78	1.76	3.56	4.25	2.68	3.57	1.28	8.79	1.46	2.16	1.61	1.24	7.81	2.21	3.08	1.64	1.39	1.18	4.3	1.64	9.94	3.05	1.7	2.34	2	1.2	3.28	0.992	3.79	1.13
Betaine_Post-UK	0.736	2.02	1.21	1.26	0.769	0.512	0.738	1.16	0.874	2.84	0.955	1.14	0.541	0.642	1.55	0.503	0.777	0.335	2.02	1.13	0.994	0.626	1.24	0.978	0.796	1.3	0.266	0.935	0.545	2.57	0.776	0.599
Benzimidazole_Post-UK	0	0	0	0	0	0	0	0	0	0	0	0	0	0	0	0	0	0	0.061	0	0	0	0	0	0	0	0	0	0	0	0	0
2,4-Diaminobutyrate_Post-UK	0	0	0	0	0	0	0	0	0	0	0	0	0	0	0	0	0	0	0	0	0	0	0	0	0	0	0	0	0	0	0	0
4-Amino-3-hydroxybutyrate_Post-UK	0	0	0	0	0	0	0	0	0	0	0	0	0	0	0	0	0	0	0.241	0	0	0	0	0	0	0	0	0	0	0	0	0
Homoserine_Post-UK	0.0671	0	0.0889	0.101	0.123	0.0839	0	0.0705	0.0644	0.15	0.0704	0.112	0.0913	0.0968	0.119	0.0628	0.0615	0.0387	0.175	0	0.0969	0.0872	0	0	0	0.0945	0	0	0.0677	0.165	0.0454	0
α-Methylserine_Post-UK	0	0	0	0	0	0	0	0	0	0	0	0	0	0	0	0	0	0	0	0	0	0	0	0	0	0	0	0	0	0	0	0
Thr_Post-UK	0.663	1.67	1.26	0.635	1.31	2	1.33	1.84	0.758	1.6	0.988	0.883	0.837	0.87	6.17	0.643	1.81	0.951	1.04	0.655	1.26	1.45	0.594	1.54	0.889	1.57	1.18	0.96	1.14	0.876	1.16	0.997
Purine_Post-UK	0	0	0	0	0	0	0	0	0	0	0	0	0	0	0	0	0	0	0	0	0	0	0	0	0	0	0	0	0	0	0	0
Anserine_Post-UK	0.052	0.0186	0.0592	0.0115	0.0728	0.0211	0.261	0.0115	0.0255	0.0426	0.0336	0.0221	0.924	0	0.105	0.021	0.0671	0.0373	0.165	0.144	0.0226	0.026	0	0.0184	0	0.00594	0	0.239	0.0942	0.0345	0	0.0187
Homocarnosine_Post-UK	0	0	0	0	0	0	0	0	0	0	0	0	0	0.0161	0.0528	0	0	0	0.0547	0	0	0	0	0	0	0	0	0	0	0	0	0
Benzamidine_Post-UK	0	0	0	0	0	0	0	0	0	0	0	0	0	0	0	0	0	0	0.0705	0	0	0	0	0	0	0	0	0	0	0	0	0
Cys_Post-UK	0	0	0	0	0	0	0	0	0	0	0	0	0	0	0	0	0	0	0	0	0	0	0	0	0	0	0	0	0	0	0	0
Benzamide_Post-UK	2.19	16.1	5.31	1.88	3.3	1.94	9.83	3.04	2.95	10	1.77	5.12	1.81	4.49	2.71	2.93	3.97	3.46	1.59	2.63	6.51	2.16	5.21	4.78	3.51	2.91	4.29	3.29	5.25	2.06	7.14	3
Phenethylamine_Post-UK	0	0	0	0	0	0	0	0	0	0	0	0	0	0	0	0	0	0	0	0	0	0	0	0	0	0	0	0	0	0	0	0
α-Methylbenzylamine_Post-UK	0	0	0	0	0	0	0	0	0	0	0	0	0	0	0	0	0	0	0	0	0	0	0	0	0	0	0	0	0	0	0	0
2,4-Dimethylaniline_Post-UK	0	0	0	0	0	0	0	0	0	0	0	0	0	0	0	0	0	0	0	0	0	0	0	0	0	0	0	0	0	0	0	0
Isonicotinamide_Post-UK	0	0	0	0	0	0	0	0	0	0	0	0	0	0	0	0	0	0	0	0	0	0	0	0	0	0	0	0	0	0	0	0
Nicotinamide_Post-UK	0.0646	0.137	0.133	0.082	0.113	0.0912	0.0942	0.123	0	0.352	0.116	0.0862	0.0624	0.109	0.208	0.0802	0.021	0.11	0.233	0.0341	0.159	0.0928	0.0527	0.136	0.0937	0.117	0.051	0.081	0.123	0.103	0.0555	0.101
Picolinamide_Post-UK	0	0	0	0	0	0	0	0	0	0	0	0	0	0	0	0	0	0	0	0	0	0	0	0	0	0	0	0	0	0	0	0
3-Chloroalanine_Post-UK	0	0	0	0	0	0	0	0	0	0	0	0	0	0	0	0	0	0	0	0	0	0	0	0	0	0	0	0	0	0	0	0
Taurine_Post-UK	26.2	76.7	52.9	50.4	74.8	26.3	47.3	77.9	35.3	64.2	49	26.6	51.2	32.6	61.7	31.5	47.7	40.9	37.1	29.8	54.8	40.8	32.8	55.2	60.8	49.8	37	36.9	41.7	30.4	43.3	38.8
5-Methylcytosine_Post-UK	0	0	0	0	0	0	0	0	0	0	0	0	0	0	0	0	0	0	0.0476	0	0	0	0	0	0	0	0	0	0	0	0	0
1-Methylhistamine_Post-UK	0	0	0.0231	0.0192	0	0	0	0	0	0	0	0	0	0	0	0	0	0.0135	0.0623	0.0129	0	0.0154	0	0	0	0	0	0	0.0192	0	0	0
Thymine_Post-UK	1.2	5.27	1.69	1.77	3.07	2.45	1.42	6.92	3.04	4.01	2.74	1.79	3.51	2.34	3.49	1.62	6.54	1.73	2.15	2.73	4.73	3.63	1.41	2.7	3.11	4.59	2.39	2.53	1.61	4.55	2.82	2.21
Imidazole-4-acetate_Post-UK	0	0	0	0	0	0	0	0	0	0	0	0	0	0	0	0	0	0	0	0	0	0	0	0	0	0	0	0	0	0	0	0
Melamine_Post-UK	0	0	0	0	0	0	0	0	0	0	0	0	0	0	0	0	0	0	0.0576	0	0	0	0	0	0	0	0	0	0	0	0	0
1-Amino-1-cyclopentanecarboxylate_Post-UK	0	0	0	0	0	0	0	0	0	0	0	0	0	0	0	0	0	0	0.0588	0	0	0	0	0	0	0	0	0	0	0	0	0
Pipecolate_Post-UK	0.066	0.0972	0.208	0.293	0.112	0.0994	0.0994	0.074	0.124	0.276	0.257	0	0.083	0	0.159	0.0562	0.354	0.0776	0.296	0.0633	0.0894	0.0675	0.0636	0.114	0.19	0.199	0.0824	0.038	0.0823	0.0987	0.133	0.0564
Metformin_Post-UK	0	0	0	0	0	0	0	0	0	0	0	0	0	0	0	0	0	0	0.0752	0	0	0	0	0	0	0	0	0	0	0	0	0
Octylamine_Post-UK	0	0	0	0	0	0	0	0	0	0	0	0	0	0	0	0	0	0	0.0295	0	0	0	0	0	0	0	0	0	0	0	0	0
N-Acetylputrescine_Post-UK	0.351	1.01	0.738	0.394	0.626	0.287	0.811	0.517	0.377	2.72	0.444	0.499	0.356	0.38	0.437	0.459	0.282	0.347	0.586	0.424	0.798	0.558	0.846	0.38	0.345	1.17	0.244	0.111	0.627	1.35	0.548	0.0901
Agmatine_Post-UK	0.0361	0.125	0.0491	0.0544	0.0738	0.0508	0.0484	0.0349	0.0609	0.0787	0.0351	0.0382	0.0356	0.0518	0.0573	0.0537	0	0.0356	0.146	0.0303	0.0545	0.0403	0.0271	0.0445	0.0287	0.0484	0.0318	0	0.0391	0.0787	0	0
Hydroxyproline_Post-UK	0.0916	0.257	0.396	0.318	0.318	0.261	0.265	0.177	0.162	0.288	0.435	0.188	0.182	0.229	2.05	0.213	0.643	0.271	0.214	0.192	0.205	0.244	0.0756	0.17	0.187	0.248	0.31	0.179	0.147	0.174	0.14	0.219
5-Aminolevulinate_Post-UK	0	0.037	0.0301	0	0.0369	0.0447	0.0217	0	0	0.0514	0.041	0.02	0	0.0331	0.0372	0.0278	0.0243	0	0.0659	0	0.0233	0.0425	0.0267	0	0	0	0.0396	0	0	0.0533	0.0164	0
Creatine_Post-UK	3.36	6.92	5.66	6.25	4.49	4.76	3.64	6.83	3.82	9.57	5.7	4.46	3.87	5.24	21.3	2.38	7.01	4.19	4.54	4.79	5.33	5.43	2.73	4.19	6.26	6.76	4.45	5.4	2.99	4.71	3.73	4.23
6-Aminohexanoate_Post-UK	0	0.275	0	0	0	0	0	0	0	0	0	0.347	0	0	0	0	0	0	0.107	0	0	0	0	0	0	0	0	0	0	0	0	0
β-Leucine_Post-UK	0	0	0	0	0	0	0	0	0	0	0	0	0	0	0	0	0	0	0.0522	0	0	0	0	0	0	0	0	0	0	0	0	0
Ile_Post-UK	0.324	0.59	1.43	0.233	0.934	1.03	0.744	1.54	0.288	1.85	0.311	0.415	0.439	0.312	2.12	0.436	1.51	0.377	0.442	0.27	1.01	0.43	3.19	1.01	0.359	0.723	0.537	0.567	0.883	0.292	1.12	0.315
Leu_Post-UK	1.19	2.21	4.76	1.08	3.68	2.8	2.85	4.39	1.29	4.96	0.877	1.93	1.09	1	5.99	1.44	3.27	1.41	1.37	1.26	3.09	1.39	7.11	3.22	1.15	2.21	1.4	1.64	2.54	1.38	2.96	0.746
Bis(3-aminopropyl)amine_Post-UK	0	0	0	0	0	0	0	0	0	0	0	0	0	0	0	0	0	0	0.0541	0	0	0	0	0	0	0	0	0	0	0	0	0
Gly-Gly_Post-UK	0	0	0.197	0.148	0.0924	0.126	0.175	0.0521	0.108	0.222	0.0533	0.147	0.0761	0.0534	0.163	0.0906	0.07	0.114	0.127	0.0649	0.226	0	0.0849	0.132	0.141	0.294	0.0339	0.0516	0.104	0.187	0.0608	0.0571
Asn_Post-UK	0.231	0.327	0.23	0.281	0.957	0	0.049	0.176	0.0884	0	0.0467	0.14	0.294	0	2.53	0	0.339	0.172	0.132	0.326	0.0827	0.525	0.152	0	0.0596	0.0445	0	0.363	0	0.355	0.182	0.213
Ornithine_Post-UK	1.23	1.76	3.92	1.75	5.37	1.83	3.57	1.33	1.75	6.63	0.797	6	1.03	1.82	4.22	2.32	0.778	2.06	1.79	1.16	4.23	2.64	2.25	3.9	1.6	7.43	0.524	0.812	3.69	12.7	1.09	0.363
Asp_Post-UK	1.27	2.24	1.85	1.54	2.5	2.15	2.52	2.08	2.11	5.73	2.89	2.8	1.54	3.13	20.8	2.32	3.03	3.19	2.95	3.02	4.96	2.36	2.4	5.86	3.47	6.97	2.51	2.41	4.46	3.31	2.03	1.18
2-Aminobenzimidazole_Post-UK	0	0	0	0	0	0	0	0	0	0	0	0	0	0	0	0	0	0	0.062	0	0	0	0	0	0	0	0	0	0	0	0	0
Homocysteine_Post-UK	0	0	0	0	0	0	0	0	0	0	0	0	0	0	0	0	0	0	0	0	0	0	0	0	0	0	0	0	0	0	0	0
Adenine_Post-UK	0.127	0.314	0.337	0.124	0.207	0.261	0.168	0.244	0.173	0.342	0.34	0.115	0.0743	0.269	0.38	0.199	0.407	0.257	0.3	0.153	0.301	0.168	0.168	0.49	0.311	0.332	0.231	0.248	0.312	0.123	0.184	0.118
Hypoxanthine_Post-UK	0.852	2.49	1.56	1.15	0.947	1.89	1.12	4.37	1.15	2.18	1.2	1.39	1.06	2.42	4.85	0.998	3.77	2.41	2.01	2.4	2.43	2.49	0.95	3.32	2.8	3.09	2.51	2.84	2.64	0.412	2.25	1.25
1-Methylnicotinamide_Post-UK	0	0.0992	0.0299	0	0	0	0.04	0.0173	0	0.0297	0.0204	0.0251	0.0259	0	0.0648	0	0.121	0.0184	0.157	0	0	0	0.0285	0	0.0177	0.0332	0.0164	0	0	0	0.0298	0
p-Aminobenzoate_Post-UK	0	0	0	0	0	0	0	0	0	0	0	0	0	0	0	0	0	0	0	0	0	0	0	0	0	0	0	0	0	0	0	0
Trigonelline_Post-UK	0.956	0.126	0	0	0.265	0.0277	0.0703	0.0522	0	0.0386	0	0.128	0.0667	0	0.626	0	0	0	0.0884	0	0.09	0.283	0	0	0	0.467	0	0	0	0.0452	0	0
Anthranilate_Post-UK	0	0	0	0	0	0	0	0	0	0	0	0	0	0	0	0	0	0	0	0	0	0	0	0	0	0	0	0	0	0	0	0
Phenylethanolamine_Post-UK	0	0	0	0	0	0	0	0	0	0	0	0	0	0	0	0	0	0	0	0	0	0	0	0	0	0	0	0	0	0	0	0
Tyramine_Post-UK	0	0.0613	0	0	0.0467	0	0	0	0	0	0.0378	0	0.0246	0	0	0	0	0.117	0.0724	0	0	0	0	0	0	0	0	0	0.0668	0	0.0234	0
Urocanate_Post-UK	0.223	0.63	0.38	0.227	1	0.367	0.306	0.164	0.0593	0.293	0.383	0.295	0.226	0.23	0.72	0.242	0.35	0.363	0.503	0.0888	0.375	0.449	0.215	0.533	0.36	0.951	0.109	0	0.208	0.589	0.11	0.139
6-Hydroxynicotinate_Post-UK	0	0	0	0	0	0	0	0	0	0	0	0	0	0	0	0	0	0	0	0	0	0	0	0	0	0	0	0	0	0	0	0
Tropinone_Post-UK	0	0	0	0	0	0	0	0	0	0	0	0	0	0	0	0	0	0	0.0864	0	0	0	0	0	0	0	0	0	0	0	0	0
Histidinol_Post-UK	0	0	0	0	0.00688	0	0	0	0	0.0209	0	0	0	0	0	0	0	0	0.0608	0	0	0	0	0	0	0	0	0	0	0	0	0
Tropine_Post-UK	0	0	0	0	0	0	0	0	0	0	0	0	0	0	0	0	0	0	0.0822	0	0	0	0	0	0	0	0	0	0	0	0	0
Ectoine_Post-UK	0	0	0	0	0	0	0	0	0	0	0	0	0	0	0	0	0	0	0.0579	0	0	0	0	0	0	0	0	0	0	0	0	0
4-Methyl-5-thiazoleethanol_Post-UK	0	0	0	0	0	0	0	0	0	0	0	0	0	0	0	0	0	0	0	0	0	0	0	0	0	0	0	0	0	0	0	0

Proline betaine_Post-UK	0.08	0	0.113	0.0447	0.012	0.0684	0.023	0.0149	0.0377	0.108	0.0244	0.0124	0	0	0.397	0.0473	0.024	0.021	0.108	0	0.0193	0	0.0314	0.0161	0.0777	0	0.0238	0.0275	0.0106	0.0359	0.0419	0
N^1,N^{12}-Diacetylspermine_Post-UK	0	0.038	0.0263	0	0.0383	0	0.0223	0.0189	0.034	0.0434	0.0279	0.0228	0.0219	0.0303	0.0117	0.0161	0.0166	0.0281	0.0499	0.0182	0.0367	0.0217	0.0206	0.0262	0.0163	0	0.0247	0.00889	0.0244	0.0205	0.016	0
γ-Guanidinobutyrate_Post-UK	0.017	0	0	0.0295	0	0	0	0	0	0	0	0	0	0.0238	0.0398	0	0	0	0.0657	0	0	0	0	0	0.0167	0.0111	0	0	0	0.0529	0	0
Acetylcholine_Post-UK	0	0	0	0	0	0	0	0	0	0	0	0	0	0	0	0	0	0	0.0885	0	0	0	0	0	0	0	0	0	0	0	0	0
γ-Butyrobetaine_Post-UK	0.243	1.98	0.783	0.238	0.472	0.651	0.758	0.547	0.575	1.98	0.566	0.582	0.375	0.565	0.649	0.617	0.449	0.575	0.665	0.448	0.705	0.325	0.719	0.326	0.831	0.803	0.424	0.0963	0.663	0.432	0.513	0.0734
Lysinamide_Post-UK	0	0	0	0	0	0	0.0322	0	0	0	0	0	0	0	0	0	0	0	0.0479	0	0	0	0	0	0	0	0	0	0	0	0	0
Spermidine_Post-UK	0.117	0.527	0.303	0.16	0.322	0.407	0.462	0.226	0.336	1.05	0.165	0.172	0.111	0.252	0.545	0.281	0.122	0.47	0.409	0.125	0.696	0.294	0.157	0.467	0.236	0.406	0.336	0.33	0.416	0.145	0.201	0.0395
Gln_Post-UK	2.17	5.68	6.01	3.42	5.5	6.37	3.35	7.08	2.44	6.15	3.68	3.63	3.05	3.62	21.2	2.69	7.66	3.87	3.78	2.86	5.16	6.2	2.39	5.72	5.44	8.79	4.19	4.56	3.72	3.8	3.58	3.92
5,6-Dimethylbenzimidazole_Post-UK	0	0	0	0	0	0	0	0	0	0	0	0	0	0	0	0	0	0	0.0409	0	0	0	0	0	0	0	0	0	0	0	0	0
Lys_Post-UK	1.92	3.06	3.45	1.35	16	1.65	5.42	3.2	3.69	6.42	1.13	3.05	2.09	2.64	9.49	3.29	3.24	3.58	3.13	2.35	8.83	5.22	3.83	4.06	4.31	12.5	1.72	2.16	4.89	19.5	1.63	1.09
Trientine_Post-UK	0	0	0	0	0	0	0	0	0	0	0	0	0	0	0	0	0	0	0	0	0	0	0	0	0	0	0	0	0	0	0	0
Glu_Post-UK	4.18	8.43	12.9	8.02	8.64	8.71	8.8	9.54	5.64	14.3	6.49	10.4	4.01	7.97	55.5	5.28	10.1	6.92	6.46	6.52	12.2	9.59	5.42	9.26	6.84	17.4	8.44	5.41	8.01	13.7	6.21	3.07
O-Acetylserine_Post-UK	0	0	0.107	0	0.223	0	0.126	0	0	0.189	0.0883	0.112	0.0722	0	0.135	0	0	0	0.111	0.0147	0.144	0.109	0	0	0	0	0	0	0	0.227	0	0
Nornicotine_Post-UK	0	0	0	0	0	0	0	0	0	0	0	0	0	0	0	0	0	0	0	0	0	0	0	0	0	0	0	0	0	0	0	0
Met_Post-UK	0.102	0.209	0.197	0.0686	0.199	0.132	0.1	0.891	0.0461	0.367	0.171	0	0.235	0	0.847	0	0.33	0.206	0.288	0.0975	0.334	0.206	0.13	0.185	0.0772	0.495	0.134	0.109	0.323	0	0.231	0.224
3-Methyladenine_Post-UK	0	0	0	0	0	0	0	0	0	0	0	0	0	0	0	0	0	0	0.0853	0	0	0	0	0	0	0	0	0	0	0	0	0
6-Methylaminopurine_Post-UK	0	0	0	0	0	0	0	0	0	0	0	0	0	0	0	0	0	0	0	0	0	0	0	0	0	0	0	0	0	0	0	0
Guanine_Post-UK	0.418	0.797	0.793	0.473	0.462	0.736	0.32	1.13	0.339	0.456	0.594	0.474	0.443	0.874	1.92	0.36	0.934	0.68	0.204	0.765	0.41	1.03	0.319	0.916	0.957	0.364	1.06	1.16	0.307	0.231	0.701	0.616
Amantadine_Post-UK	0	0	0	0	0	0	0	0	0	0	0	0	0	0	0	0	0	0	0.0457	0	0	0	0	0	0	0	0	0	0	0	0	0
Xanthine_Post-UK	0.499	2.28	1.13	1.06	1.99	1.41	0.933	2.1	1.89	0	1.15	0.897	0	1.55	7.46	0.55	4.41	2.04	0.624	1.81	1.99	0.422	1.79	2.47	1.97	2.2	2.8	2.43	2.07	0.542	1.5	0
4-Aminosalicylate_Post-UK	0	0	0	0	0	0	0	0	0	0	0	0	0	0	0	0	0	0	0	0	0	0	0	0	0	0	0	0	0	0	0	0
3-Hydroxyanthranilate_Post-UK	0	0	0	0	0	0	0	0	0	0	0	0	0	0	0	0	0	0	0	0	0	0	0	0	0	0	0	0	0	0	0	0
Dopamine_Post-UK	0	0	0	0	0	0	0	0	0	0	0	0	0	0	0	0	0	0	0	0	0	0	0	0	0	0	0	0	0	0	0	0
4-(β-Acetylaminoethyl)imidazole_Post-UK	0.028	0	0.0412	0.0341	0.0114	0	0.0133	0.0136	0	0	0	0	0	0	0	0	0	0.0284	0.0835	0	0	0.0102	0.0149	0	0	0.0125	0	0	0.0498	0	0.0153	0
Pseudopelletierine_Post-UK	0	0	0	0	0	0	0	0	0	0	0	0	0	0	0	0	0	0	0.0556	0	0	0	0	0	0	0	0	0	0	0	0	0
His_Post-UK	2.29	2.67	2.32	2.95	5.5	1.7	4.68	2.3	2.4	3.42	1.43	2.79	1.18	2.88	5.06	2.31	3.55	2.32	3.07	1.74	4.1	2.39	2.15	2.9	3.19	5.69	1.11	1.61	2.32	4.43	0.935	1.11
β-Imidazolelactate_Post-UK	0	0	0	0	0	0	0.0712	0	0	0	0	0	0	0	0	0	0.0647	0	0	0	0	0.102	0	0.0768	0.0495	0	0	0.0525	0.0755	0	0	0
Ibotenate_Post-UK	0	0	0	0	0	0	0	0	0	0	0	0	0	0	0	0	0	0	0	0	0	0	0	0	0	0	0	0	0	0	0	0
Allantoin_Post-UK	13.1	13.6	24	18.5	20.9	15.3	15.6	9.26	23.5	23.8	13.6	12.6	17.4	14.8	21.1	21.5	11.9	17	18.2	17.8	21.9	14	9.92	16.4	13	19	12.3	14.3	15.9	16.4	9.75	10.5
Indole-3-acetaldehyde_Post-UK	0	4.91	20.2	0	0	0	17.5	0	20	0	0	0	18.9	3.7	0	18.6	0	14	0	0	0	12.4	0	0	7.28	0	0	5.39	0	0	9.01	0
Betonicine_Post-UK	0	0	0	0	0	0	0	0	0	0	0	0	0	0	0	0	0	0	0.0599	0	0	0	0	0	0	0	0	0	0	0	0	0
N-Acetylvaline_Post-UK	0	0	0	0	0	0	0	0	0	0	0	0	0	0	0	0	0	0	0	0	0	0	0	0	0	0	0	0	0	0	0	0
Ala-Ala_Post-UK	0	0	0.194	0.159	0.193	0.0914	0.17	0	0.127	0.353	0	0.14	0.0857	0	0.103	0	0.13	0	0.17	0.102	0.255	0	0.11	0	0.165	0.236	0.108	0.0705	0.25	0.203	0.0929	0.0579
Tryptamine_Post-UK	0	0	0	0	0	0	0	0	0	0	0	0	0	0	0	0	0	0	0	0	0	0	0	0	0	0	0	0	0	0	0	0
α-Aminoadipate_Post-UK	0.201	0.724	0.502	0.391	0.658	0.426	0.571	0.564	0.212	0.911	0.871	0.492	0.319	0.415	1.39	0.33	0.697	0.463	0.442	0.614	0.619	0.645	0.253	0.305	0.606	0.575	0.457	0.471	0.275	0.883	0.376	0.65
N-Methylglutamate_Post-UK	0	0	0	0	0	0	0	0	0	0	0	0	0	0	0	0	0	0	0	0	0	0	0	0	0	0	0	0	0	0	0	0
Indole-3-ethanol_Post-UK	0	0	0	0	0	0	0	0	0	0	0	0	0	0	0	0	0	0	0	0	0	0	0	0	0	0	0	0	0	0	0	0
Carnitine_Post-UK	0.236	0.812	0.442	0.371	0.441	0.456	0.317	0.662	0.337	0.838	0.419	0.53	0.207	0.447	1.26	0.375	0.643	0.422	0.534	0.465	0.497	0.456	0.389	0.555	0.64	0.617	0.268	0.59	0.356	0.559	0.249	0.192
2-Deoxystreptamine_Post-UK	0	0	0	0	0	0	0	0	0	0	0	0	0	0	0	0	0	0	0	0	0	0	0	0	0	0	0	0	0	0	0	0
5-Hydroxylysine_Post-UK	0	0	0	0	0	0	0	0	0	0	0	0	0	0	0	0	0	0	0	0	0	0	0	0	0	0	0	0	0	0	0	0
Nicotine_Post-UK	0	0	0	0	0	0	0	0	0	0	0	0	0	0	0	0	0	0	0.0636	0	0	0	0	0	0	0	0	0	0	0	0	0
Pterin_Post-UK	0	0	0	0	0	0	0	0	0	0	0	0	0	0	0	0	0	0	0	0	0	0	0	0	0	0	0	0	0	0	0	0
S-Methylmethionine_Post-UK	0	0	0	0	0	0	0	0	0	0	0	0	0	0	0	0	0	0	0.0535	0	0	0	0	0	0	0	0	0	0	0	0	0
Methionine sulfoxide_Post-UK	0	0	0	0	0	0	0.0902	0.129	0.037	0	0.0412	0	0	0	0.786	0.0695	0.0752	0.0701	0.138	0.0397	0	0	0	0	0.07	0.101	0.0532	0.067	0.094	0.0434	0.0774	0
7-Methylguanine_Post-UK	0	0.0521	0.0351	0.0451	0.047	0	0.035	0.0338	0	0.0756	0.0456	0.0366	0.041	0.0302	0.0446	0.036	0.0283	0.0524	0.0584	0	0	0.0482	0	0.0369	0.0445	0.0272	0.0433	0	0	0.0384	0.0356	0
3-Methylguanine_Post-UK	0	0	0	0	0	0	0	0	0	0	0	0	0	0	0.37	0	0	0	0	0	0	0	0	0	0	0	0	0	0	0	0	0
Phe_Post-UK	3.23	2.31	5.39	3.86	11.3	3.31	4.95	3.3	3.22	7.07	1.93	4.58	1.7	2.57	9.11	3.13	2.66	2.55	2.16	1.81	5.78	4.02	4.26	4.99	1.68	5.34	1.33	2.12	4.01	3.54	3.65	1.48
Taurocyamine_Post-UK	0	0	0	0	0	0	0	0	0	0	0	0	0	0	0	0	0	0	0	0	0	0	0	0	0	0	0	0	0	0	0	0
Pyridoxal_Post-UK	0	0	0	0	0	0	0	0	0	0	0	0	0	0	0	0	0	0	0	0	0	0	0	0	0	0	0	0	0	0	0	0
3-Methoxytyramine_Post-UK	0	0	0	0	0	0	0	0	0	0	0	0	0	0	0	0	0	0	0	0	0	0	0	0	0	0	0	0	0	0	0	0
Synephrine_Post-UK	0	0	0	0	0	0	0	0	0	0	0	0	0	0	0	0	0	0	0.061	0	0	0	0	0	0	0	0	0	0	0	0	0
Pyridoxamine_Post-UK	0	0	0	0	0	0	0	0	0	0	0	0	0	0	0	0	0	0	0.0385	0	0	0	0	0	0	0	0	0	0	0	0	0
Pyridoxine_Post-UK	0	0	0	0	0	0	0	0	0	0	0	0	0	0	0	0	0	0	0.042	0	0	0	0	0	0	0	0	0	0	0	0	0
Noradrenaline_Post-UK	0	0	0	0	0	0	0	0	0	0	0	0	0	0	0	0	0	0	0	0	0	0	0	0	0	0	0	0	0	0	0	0
3-Methylhistidine_Post-UK	0.0608	0.0688	0.124	0	0.0883	0.0645	0.225	0.0565	0.0606	0.101	0.0555	0.104	0.172	0.0438	0.546	0.0494	0.333	0.0871	0.241	0.124	0.0384	0.0477	0.0246	0.0608	0.0655	0	0.0506	0.0495	0.119	0.0558	0.0379	0
Indole-3-acetamide_Post-UK	0	0	0	0	0	0	0	0	0	0	0	0.473	0	0	0.64	0	0	0	0	0	0	0.56	1.54	0	0	0	0	1.38	2.15	0	0	0
N-Acetylornithine_Post-UK	0	0.0519	0.0295	0	0	0.0377	0.0621	0	0.0292	0.0821	0	0.0425	0.0249	0.027	0	0	0	0.0297	0.0758	0	0.0598	0	0	0.0502	0	0.0611	0.0399	0	0.0537	0.0234	0	0
N-γ-Ethylglutamine_Post-UK	0.0465	0	0.124	0.0546	0.144	0.0581	0	0.0549	0	0.282	0.068	0	0.0735	0	0.129	0.0727	0	0.0647	0.0558	0	0.0711	0.072	0	0	0	0.114	0	0	0	0.132	0	0
Arg_Post-UK	2.65	4.65	3.22	2.53	4.8	2.27	5.42	3.24	4.18	18.1	2.73	8.21	2.26	4.68	8.8	2.94	5.85	5.21	6.28	3.69	10.6	4.02	6.55	10.1	4.96	13.5	2.44	4.35	5.2	10.4	3.75	1.81
Gramine_Post-UK	0	0	0	0	0	0	0	0	0	0	0	0	0	0	0	0	0	0	0.0591	0	0	0	0	0	0	0	0	0	0	0	0	0
N-ω-Methyltryptamine_Post-UK	0	0	0	0	0	0	0	0	0	0	0	0	0	0	0	0	0	0	0.033	0	0	0	0	0	0	0	0	0	0	0	0	0
Guanidinosuccinate_Post-UK	0	0	0	0	0	0	0	0	0	0	0	0	0	0	0	0	0	0	0.0517	0	0	0	0	0	0	0	0	0	0	0	0	0
Indole-3-acetate_Post-UK	0.372	2.08	0	0	0.621	0.509	2.92	1.76	1.39	1.1	1.25	0.779	0.677	1.13	0.601	0.814	0.459	1.06	1.06	0.686	1.06	0.686	1.61	0.927	0.679	2.57	0.887	0	1.02	0.922	2.68	0
2-Guanidinobenzimidazole_Post-UK	0	0	0	0	0	0	0	0	0	0	0	0	0	0	0	0	0	0	0.0311	0	0	0	0	0	0	0	0	0	0	0	0	0
Citrulline_Post-UK	0.545	1.72	0.946	0.817	1.74	0.999	1.37	2.06	0.984	2.4	0.802	1.45	0.675	1.12	6.24	0.646	2.1	1.74	1.28	0.803	3.12	1.04	1.33	2.71	1.6	6.62	0.375	0.695	1.87	2.87	1.24	0.491
Canavanine_Post-UK	0	0	0	0	0	0	0	0	0	0	0	0	0	0	0	0	0	0	0.0412	0	0	0	0	0	0	0	0	0	0	0	0	0
Serotonin_Post-UK	0	0	0	0	0	0	0	0	0	0	0	0	0	0	0	0	0	0	0	0	0	0	0	0	0	0	0	0	0	0	0	0
Alliin_Post-UK	0	0	0	0	0	0	0	0	0	0	0	0	0	0	0.52	0	0	0	0	0	0	0	0	0	0	0	0	0	0	0	0	0
Cys-Gly_Post-UK	0	0	0	0	0	0	0	0	0	0	0	0	0	0	0	0	0	0	0	0	0	0	0	0	0	0	0	0	0	0	0	0
Xanthopterin_Post-UK	0	0	0	0	0	0	0	0	0	0	0	0	0	0	0	0	0	0	0	0	0	0	0	0	0	0	0	0	0	0	0	0
Mannosamine_Post-UK	0	0.0419	0	0	0	0	0	0.0799	0	0.187	0	0	0	0	0.0662	0	0.0827	0	0	0	0.146	0.0204	0	0.14	0	0	0	0	0	0	0.0589	0
Glucosamine_Post-UK	0.0558	0.13	0.0357	0.0631	0	0.0302	0.0613	0.264	0.0601	0.186	0.0508	0.0541	0.0962	0.0444	0.0851	0.0242	0.608	0.1	0.118	0.0511	0.312	0	0	0.109	0.147	0.298	0	0.0348	0	0.126	0	0
n-Butyl a-Picolinate_Post-UK	0	0	0	0	0	0	0	0	0	0	0	0	0	0	0	0	0	0	0	0	0	0	0	0	0	0	0	0	0	0	0	0
Methionine sulfoximine_Post-UK	0	0	0	0	0	0	0	0	0	0	0	0	0	0	0	0	0	0	0	0	0	0	0	0	0	0	0	0	0	0	0	0
Tyr_Post-UK	3.13	3.57	5.27	3.63	19.1	2.15	7.56	3.69	6.28	6.41	2.12	5.84	2.29	3.74	10.9	3.58	4.46	3.51	3.21	3.23	6.11	6.46	5.52	5.68	2.63	9.43	1.92	3.24	4.48	8.49	3.31	1.59
Harman_Post-UK	0	0	0	0	0	0	0	0	0	0	0	0	0	0	0	0	0	0	0.04	0	0	0	0	0	0	0	0	0	0	0	0	0
Phosphorylcholine_Post-UK	2.61	4.27	3.53	2.39	4.43	1.37	3.65	2.22	1.51	7.96	2.74	3.55	1.94	2.71	5.42	1.45	4	1.8	3.14	2.79	2.6	3.54	3.64	2.59	3.67	2.01	1.68	1.62	0.951	3.76	4.26	1.61
Epinephrine_Post-UK	0	0	0	0	0	0	0	0	0	0	0	0	0	0	0	0	0	0	0	0	0	0	0	0	0	0	0	0	0	0	0	0
N-α,N-α-Dimethylhistidine_Post-UK	0	0	0	0	0	0	0	0	0	0	0	0	0	0	0	0	0	0	0.052	0	0	0	0	0	0	0	0	0	0	0	0	0
N^8-Acetylspermidine_Post-UK	0	0.0315	0.0228	0	0.0139	0	0.00879	0.0101	0.0131	0.0372	0.00846	0.0073	0	0.0115	0	0	0.025	0.0123	0.0912	0.0187	0.0193	0	0.00728	0.0162	0.014	0.0151	0.0278	0.0141	0.00817	0.0112	0.00776	0.00753
N^1-Acetylspermidine_Post-UK	0.0911	0.177	0.201	0.052	0.128	0.235	0.085	0.0893	0.183	0.217	0.0964	0.0466	0.0443	0.0783	0.0722	0.0971	0.206	0.244	0.215	0.105	0.145	0.0977	0.0556	0.115	0.147	0.0873	0.122	0.183	0.0448	0.0957	0.054	0.08
Gly-Leu_Post-UK	0.036	0.0753	0.0416	0.0233	0.0562	0.041	0.0823	0.147	0.0444	0.142	0.0404	0.033	0.0468	0.0667	0	0.027	0.0473	0.067	0.146	0.0433	0.154	0.0261	0.0309	0.171	0.0593	0.158	0.0491	0.0347	0.204	0.0525	0.0815	0
N-ε-Acetyllysine_Post-UK	0.0401	0.0587	0.0871	0.103	0.123	0.0438	0.0553	0.044	0.0687	0.138	0.0777	0.0692	0.0495	0.0583	0	0.0377	0.133	0.0581	0.104	0.0336	0.0559	0.0757	0.0498	0	0.0813	0.0949	0.0798	0.0371	0.0476	0.192	0.0449	0
N^6,N^6,N^6-trimethyllysine_Post-UK	0.0228	0.0448	0.0306	0.0204	0.0353	0.0391	0.0243	0.0221	0.0237	0.0549	0.0408	0.0198	0.0172	0.0363	0.0872	0	0.0399	0.032	0.0796	0	0.0246	0.0322	0.0173	0.0315	0.0395	0.0433	0.0656	0.0271	0.0361	0.0202	0.0363	0.0281
Castanospermine_Post-UK	0	0	0	0	0	0	0	0	0	0	0	0	0	0	0	0	0	0	0.0533	0	0	0	0	0	0	0	0	0	0	0	0	0
5-Methoxytryptamine_Post-UK	0	0	0	0	0	0	0	0	0	0	0	0	0	0	0	0	0	0	0.0582	0	0	0	0	0	0	0	0	0	0	0	0	0
5-Hydroxyindoleacetate_Post-UK	0	0	0	0	0	0	0	0	0	0	0	0	0.304	0	0	0	0	0	0	0	0	0	0	0	0	0	0	0	0	0	0	0
SAH_Post-UK	0	0	0	0	0	0	0	0	0	0	0	0	0	0	0	0	0	0	0	0	0	0	0	0	0	0	0	0	0	0	0	0
Glucosaminate_Post-UK	0	0	0	0	0	0	0	0	0	0	0	0	0	0	0	0	0	0	0	0	0	0	0	0	0	0	0	0	0	0	0	0
DOPA_Post-UK	0	0	0	0	0	0	0	0	0	0	0	0	0	0	0	0	0	0	0	0	0	0	0	0	0	0	0	0	0	0	0	0
N-Acetylhistidine_Post-UK	0	0	0	0	0	0	0	0	0	0	0	0	0	0	0	0	0	0	0	0	0	0	0	0	0	0	0	0	0	0	0	0

SAM+_Post-UK	0	0.0453	0.015	0	0	0.0421	0.0251	0.0282	0.0206	0.0451	0.0185	0.0152	0	0.0179	0.0344	0	0.0399	0.0358	0.036	0	0.0255	0.0395	0	0	0.0259	0.0195	0	0.0328	0	0	0	0.0159
Arginine ethyl ester_Post-UK	0	0	0	0	0	0	0	0	0	0	0	0	0	0	0	0	0	0	0.06	0	0	0	0	0	0	0	0	0	0	0	0	0
ADMA_Post-UK	0	0.0371	0	0	0.0321	0.0288	0	0.0256	0	0	0.0235	0	0	0.0253	0.0509	0.0103	0	0	0.0651	0	0.0225	0	0.0196	0.0248	0.0202	0.0119	0.0265	0	0.0184	0.0242	0.0085	0
SDMA_Post-UK	0	0.0179	0	0.0173	0.0223	0	0.0188	0.0135	0	0.0225	0	0	0	0	0.0275	0	0.019	0	0.0538	0	0.0122	0	0	0.018	0	0	0	0	0	0.0182	0	0
o-Acetylcarnitine_Post-UK	0.339	1.18	0.348	0.428	0.536	0.243	0.641	0.454	0.475	0.863	0.289	0.785	0.27	0.369	0.715	0.303	0.636	0.266	0.396	0.482	0.604	0.363	0.604	0.508	0.491	0.555	0.153	0.685	0.302	0.717	0.302	0.203
Trp_Post-UK	0.142	0.262	0.197	0.156	0.24	0.229	0.254	0.31	0.184	0.345	0.125	0.0725	0.101	0.164	0.5	0.158	0.299	0.211	0.23	0.0967	0.312	0.145	0.183	0.211	0.145	0.232	0.177	0.108	0.283	0.0404	0.235	0.0897
5-Methoxyindoleacetate_Post-UK	0.846	0.837	0.961	0.911	1.16	0.914	0.861	0.887	1.16	1.2	1.09	0.921	0.99	0.837	2.6	1.07	0.754	0.816	0.899	0.856	1.03	0.881	0.567	0.857	0.523	1.25	0.702	0	0	1.25	0.538	0.549
Kynurenine_Post-UK	0	0	0	0	0	0	0	0	0	0	0	0	0	0	0	0	0	0	0	0	0	0	0	0	0	0	0	0	0	0	0	0
Cysteine-glutathione disulfide_Post-UK	0	0.327	0	0	0	0.166	0	0.238	0	0	0	0	0	0.0641	0.699	0	0	0	0	0.0849	0	0.19	0	0	0.0676	0	0.284	0.0869	0.0308	0	0.0553	0
Desthiobiotin_Post-UK	0	0	0	0	0.153	0	0	0.129	0	0	0	0	0	0	0	0	0	0.134	0	0	0	0.0936	0	0	0	0	0	0	0	0	0	0
o-Succinylhomoserine_Post-UK	0	0	0	0	0	0	0	0	0	0	0	0	0	0	0	0	0	0	0	0	0	0	0	0	0	0	0	0	0	0	0	0
trans-Zeatin_Post-UK	0	0	0	0	0	0	0	0	0	0	0	0	0	0	0	0	0	0	0	0	0	0	0	0	0	0	0	0	0	0	0	0
5-Hydroxytryptophan_Post-UK	0	0	0	0	0	0	0	0	0	0	0	0	0	0	0	0	0	0	0	0	0	0	0	0	0	0	0	0	0	0	0	0
N-Acetylglucosylamine_Post-UK	0.132	0	0	0	0	0	0	0	0	0.194	0	0	0	0	0	0	0	0	0	0	0.272	0	0	0	0	0	0	0	0	0	0	0
N-Acetylglucosamine_Post-UK	2.51	3.19	5.34	2.17	2.71	3.24	4.58	3.55	4.56	21.5	1.92	9.86	0.938	4.23	1.75	2.34	1.35	4.2	3.71	3.19	26.8	9.97	8.25	23.2	2.3	10.7	0.897	2.34	3.26	7	4.23	0.722
Cystathionine_Post-UK	0	0	0	0	0	0	0	0	0	0	0	0	0	0	0.181	0	0	0	0	0	0	0	0	0	0	0	0	0	0	0	0	0
3-Hydroxykynurenine_Post-UK	0	0	0	0	0	0	0	0	0	0	0	0	0	0	0	0	0	0	0	0	0	0	0	0	0	0	0	0	0	0	0	0
2'-Deoxycytidine_Post-UK	0	0	0	0	0.0365	0	0	0	0	0.036	0	0	0	0	0	0	0	0	0.0691	0	0	0.0233	0	0	0	0.0474	0	0	0	0	0	0
N^1,N^8-Diacetylspermidine_Post-UK	0	0.0611	0.0131	0	0.0199	0	0.0235	0.0228	0	0.0296	0.0371	0.0257	0.02	0.0272	0	0.0128	0.0153	0.0246	0.0509	0	0.0237	0.0216	0.013	0.0243	0.0145	0.0404	0.0258	0	0.0245	0.0143	0.0161	0.00699
γ-Glu-2AB_Post-UK	0	0	0	0	0	0	0	0	0	0	0	0	0	0	0	0	0	0	0	0	0	0	0	0	0	0	0	0	0	0	0	0
Melatonin_Post-UK	0.0494	0	0	0	0	0	0	0	0	0	0	0	0	0	0.106	0	0	0	0	0.0791	0	0	0	0.0467	0	0	0.053	0	0	0	0	0
Sepiapterin_Post-UK	0	0	0	0	0	0	0	0	0	0	0	0	0	0	0	0	0	0	0	0	0	0	0	0	0	0	0	0	0	0	0	0
7,8-Dihydrobiopterin_Post-UK	0	0	0	0	0	0	0	0	0	0	0	0	0	0	0	0	0	0	0	0	0	0	0	0	0	0	0	0	0	0	0	0
Cystine_Post-UK	0	0.131	0.106	0	0.0553	0	0.0372	0.0218	0.0704	0.102	0.0368	0	0	0.0613	0.121	0	0.286	0	0.108	0	0	0	0.0387	0.0842	0.0406	0	0.215	0.0691	0.053	0.051	0.0897	0
5-Methyl-2'-deoxycytidine_Post-UK	0	0	0	0	0	0	0	0	0	0	0	0	0	0	0	0	0	0	0.0381	0	0	0	0	0	0	0	0	0	0	0	0	0
Thymidine_Post-UK	0	1.14	1.01	0	1.06	0	0.748	0.888	1.44	0	1.23	0.546	0	0.633	1.19	0	0	0	0	0.994	1.51	0.934	0.773	0	0	1.11	0.689	0.935	0.84	0.991	0	0
Cytidine_Post-UK	0.0858	0	0.11	0.108	0.0914	0.127	0.103	0	0.118	0.444	0.206	0.387	0	0.152	0	0.155	0	0.127	0.293	0.0499	0.25	0.0897	0.154	0.341	0.146	0.208	0	0.0801	0.167	0.232	0	0
Uridine_Post-UK	1.61	1.39	1.99	1.11	1.59	1.8	1.15	1.88	1.58	2.92	1.31	1.78	1.45	1.27	3.01	1.5	2.62	1.67	2.1	2.27	2.84	1.83	1.11	1.94	2.06	2.68	1.15	1.43	1.56	2.22	1.61	1.11
N^1-Acetylspermine_Post-UK	0	0.0445	0.0261	0	0.047	0	0.0505	0.032	0	0.0297	0	0.013	0	0.0179	0	0	0	0	0	0	0.0269	0	0	0.036	0.0185	0	0.0159	0.0295	0.0161	0	0.0214	0
Octopine_Post-UK	0	0	0	0	0	0.0435	0	0	0	0.0435	0	0	0	0	0	0	0.0388	0.0546	0.0977	0	0.0833	0	0	0	0.0187	0.0703	0	0	0	0	0	0
Pyridoxamine 5'-phosphate_Post-UK	0	0	0	0	0	0	0	0	0	0	0	0	0	0	0	0	0	0	0	0	0	0	0	0	0	0	0	0	0	0	0	0
γ-Glu-cys_Post-UK	0	0	0	0	0	0	0	0	0	0	0	0	0	0	0	0	0	0	0	0	0	0	0	0	0	0	0	0	0	0	0	0
Muramate_Post-UK	0	0	0	0	0	0	0	0	0	0	0	0	0	0	0	0	0	0	0	0	0	0	0	0	0	0	0	0	0	0	0	0
5'-Deoxyadenosine_Post-UK	0	0	0	0	0	0	0	0	0	0	0	0	0	0	0	0	0	0	0.0533	0	0	0	0	0	0	0	0	0	0	0	0	0
Purine riboside_Post-UK	0	0	0	0	0	0	0	0	0	0	0	0	0	0	0	0	0	0	0	0	0	0	0	0	0	0	0	0	0	0	0	0
2'-Deoxyinosine_Post-UK	0	0	0	0	0	0	0	0	0	0	0	0	0	0	0	0	0	0	0	0	0	0	0	0	0	0	0	0	0	0	0	0
7,8-Dihydroneopterin_Post-UK	0	0	0	0	0	0	0	0	0	0	0	0	0	0	0	0	0	0	0	0	0	0	0	0	0	0	0	0	0	0	0	0
Glycerophosphorylcholine_Post-UK	0.227	0.797	0.362	0.855	1.77	0	0.347	0.385	0.357	0.554	0.29	0.947	1.04	0.762	4.24	0	0.38	0.251	0	0.858	0.25	0.852	0.355	0	0	0	0.481	0.782	0	1.06	0.687	0
Thiamine_Post-UK	0	0	0	0	0	0	0	0	0	0	0	0	0	0	0	0	0	0	0	0	0	0	0	0	0	0	0	0	0	0	0	0
N^6-Methyl-2'-deoxyadenosine_Post-UK	0	0	0	0	0	0	0	0	0	0	0	0	0	0	0	0	0	0	0.0474	0	0	0	0	0	0	0	0	0	0	0	0	0
Adenosine_Post-UK	0.0908	0.175	0.0861	0.0707	0.0293	0.0793	0.0593	0.0646	0.138	0.0682	0.314	0.128	0.0515	0.0911	0.143	0.0299	0.211	0.0504	0.0817	0.0733	0.0587	0.0788	0.0588	0.0792	0.0465	0.052	0.136	0.0553	0.0352	0.0955	0.0576	0.0515
2'-Deoxyguanosine_Post-UK	0	0	0	0	0	0	0	0	0	0	0	0	0	0	0	0	0	0	0	0	0	0	0	0	0	0	0	0	0	0	0	0
Homocystine_Post-UK	0	0	0	0	0	0	0	0	0	0	0	0	0	0	0	0	0	0	0	0	0	0	0	0	0	0	0	0	0	0	0	0
Inosine_Post-UK	0.221	0.663	0.295	0	0.203	0.501	0.334	0.404	0.257	0.346	0.299	0.278	0.25	0.282	0.5	0.136	0.469	0.312	0.281	0.257	0.571	0.604	0.276	0.394	0.507	0.511	0.369	0.396	0.196	0.181	0.322	0
Glu-Glu_Post-UK	0	0	0.0616	0	0.0469	0	0.0609	0.0388	0	0.106	0.0367	0.0432	0.0528	0	0.0259	0.0578	0.0516	0.0523	0.0966	0	0.0973	0	0.0661	0.0714	0.0591	0.11	0.0296	0	0.0605	0	0	0
Saccharopine_Post-UK	0	0	0.0171	0	0	0	0.0636	0	0	0	0	0	0	0	0.0241	0	0.114	0	0.0403	0	0	0	0	0.0215	0	0	0.0313	0	0	0.0268	0	0.0312
1-Methyladenosine_Post-UK	0.0205	0.0441	0.0169	0	0.0182	0	0.0182	0.0182	0.0187	0.0251	0.0132	0.0258	0	0.0283	0.0482	0	0	0.0177	0.0491	0.0314	0.0172	0.0263	0.0198	0.0277	0.0195	0.0104	0	0.0323	0	0.016	0.0214	0
Guanosine_Post-UK	0.107	0.351	0.168	0.0908	0.129	0.311	0.111	0.155	0.188	0.0831	0.344	0.211	0.0721	0.112	0.338	0.0485	0.185	0.162	0.0829	0.166	0.144	0.382	0.124	0.157	0.21	0.108	0.257	0.287	0.0422	0.101	0.177	0.132
Xanthosine_Post-UK	0	0	0	0	0	0	0	0	0	0	0	0	0	0	0	0	0	0	0	0	0	0	0	0	0	0	0	0	0	0	0	0
Ophthalmate_Post-UK	0	0.0313	0	0	0	0	0	0	0	0	0	0	0	0	0.0704	0	0	0	0	0	0	0	0	0	0	0	0	0	0	0	0	0
5-Methylthioadenosine_Post-UK	0	0	0	0	0	0	0	0	0	0	0	0	0	0	0	0	0	0	0	0	0	0	0	0	0	0	0	0	0	0	0	0
Glutathione(ox)_Post-UK	0	0.642	0.0625	0.0406	0.0234	0.15	0.0381	0.662	0.0372	0	0.058	0.0261	0	0.0951	1.36	0.0359	0	0.0243	0.0335	0.105	0.0515	0.359	0.0234	0	0.0511	0.0415	0.19	0.0461	0	0	0.0179	0.0982
N-α-Benzenolarginine ethylester_Post-UK	0	0	0	0	0	0	0	0	0	0	0	0	0	0	0	0	0	0	0.0472	0	0	0	0	0	0	0	0	0	0	0	0	0
3-Iodotyrosine_Post-UK	0	0	0	0	0	0	0	0	0	0	0	0	0	0	0	0	0	0	0.0406	0	0	0	0	0	0	0	0	0	0	0	0	0
Glutathione(red)_Post-UK	0	0	0	0	0	0	0	0.6	0	0	0	0	0	0	0.883	0	0	0	0	0	0	0	0	0	0	0	0	0	0	0	0	0
Phe-Phe_Post-UK	0	0.0126	0	0	0	0.00593	0.0214	0.0106	0	0.0415	0	0	0	0	0	0.0062	0	0.0308	0.0565	0	0.0313	0.00502	0	0	0	0.00932	0	0	0	0	0.00809	0
AICAR_Post-UK	0.406	0.152	0.486	0.286	0.598	0.407	0	0.281	0.477	0.566	0.371	0	0.321	0	0.501	0	0	0.39	0.536	0.403	0.483	0.363	0	0	0	0.335	0	0	0	0.386	0	0.0737
Thiamine monophosphate_Post-UK	0	0	0	0	0	0	0	0	0	0	0	0	0	0	0	0	0	0	0	0	0	0	0	0	0	0	0	0	0	0	0	0
Tetrahydropalmatine_Post-UK	0	0	0	0	0	0	0	0	0	0	0	0	0	0	0	0	0	0	0.0283	0	0	0	0	0	0	0	0	0	0	0	0	0
TRH_Post-UK	0	0	0	0	0	0	0	0	0	0	0	0	0	0	0	0	0	0	0	0	0	0	0	0	0	0	0	0	0	0	0	0
Riboflavin_Post-UK	0	0	0	0	0	0	0	0	0	0	0	0	0	0	0	0	0	0	0	0	0	0	0	0	0	0	0	0	0	0	0	0
S-Lactoylglutathione_Post-UK	0	0	0	0	0	0	0	0	0	0	0	0	0	0	0	0	0	0	0	0	0	0	0	0	0	0	0	0	0	0	0	0
Leu-Leu-Tyr_Post-UK	0	0	0	0	0	0	0	0	0	0	0	0	0	0	0	0	0	0	0.0404	0	0	0	0	0	0	0	0	0	0	0	0	0
3,5-Diiodo-tyrosine_Post-UK	0	0	0	0	0	0	0	0	0	0	0	0	0	0	0	0	0	0	0.0314	0	0	0	0	0	0	0	0	0	0	0	0	0
5-Methyltetrahydrofolate_Post-UK	0	0	0.69	0.449	0	0.522	0	0.593	0	1.88	0	0	0	0	0	0	0	0	0.815	0	1.77	0	0	0	0	0	0	0	0	0	0	0
3,3',5-Triiodothyronine_Post-UK	0	0	0	0	0	0	0	0	0	0	0	0	0	0	0	0	0	0	0	0	0	0	0	0	0	0	0	0	0	0	0	0
NMN_Post-UK	0	0	0	0	0	0	0	0	0	0	0	0	0	0	0.092	0	0	0	0	0	0	0	0	0	0	0	0	0	0	0	0	0
Glyoxylate_Post-UK	0	0.631	1.09	0	0	0	0	0	0.742	1.16	0.79	0	0	0.58	0	0.583	0.411	0	0	0	0	1.04	0	0	0.537	0	0	0	0	0	0	0
Propionate_Post-UK	5.36	41.7	9.66	9.96	15.1	4.11	29.2	12.1	11.9	49.8	5.18	16.1	10.7	16.4	7.51	10.7	13.9	11.9	6.22	7.2	31.1	10.7	16.7	9.03	20.8	21	6.52	1.71	11.7	7.04	21.3	1.33
Glycolate_Post-UK	3.49	3	2.83	4.27	2.69	4.38	3.45	1.94	3.31	4.98	3.3	3.13	5.46	2.96	5.42	4.65	2.48	1.14	2.7	4.16	2.76	4.67	2.85	2.6	3.71	2.54	2.68	0	3.13	2.16	1.36	1.54
Crotonate_Post-UK	0	0	0	0	0	0	0	0	0	0	0	0	0	0	0	0	0	0	0	0	0	0	0	0	0.404	0.823	0	0	0.804	0	0	0
Pyruvate_Post-UK	2.4	5.1	2.78	3.15	3.9	3.32	2.59	2.34	4.62	5.83	2.61	3.35	3.4	2.72	5.01	2.15	2.37	3.2	5.36	2.51	8.07	2.64	1.6	4.83	3.52	6.13	1.53	1.93	4.36	4.15	1.17	0.75
Butyrate_Post-UK	1.48	6.37	1.91	1.67	1.58	1.23	8.95	4.69	2.68	10.5	1.12	4.05	1.8	2.96	3.13	3.02	3.74	4.16	1.97	2.58	8.21	1.24	3.18	3.51	4.6	9.44	1.54	0	2.83	2.47	5.68	0
Lactate_Post-UK	88.8	113	41.8	187	94.6	79.1	53.5	88.7	70.8	166	140	82.6	59.3	128	220	140	113	64.7	125	131	151	115	34.5	114	179	190	47.8	75.4	112	82.4	26	80.1
3-Hydroxypropionate_Post-UK	12.4	11.6	7.12	35.7	6.2	6.1	8.14	5.15	6.49	14.6	17.7	8.54	10.7	9.87	31.6	10.1	6.59	6.61	21.1	14.9	9.19	12.6	4.78	7.33	23.7	17.1	7.55	6.6	12.8	6.94	4.13	10.5
Methanesulfonate_Post-UK	0	0	0	0	0	0	0	0	0	0	0	0	0	0	0	0	0	0	0	0	0	0	0	0	0	0	0	0	0	0	0	0
Tiglate_Post-UK	0	0	0	0	0	0	0	0	0	0	0	0	0	0	0	0	0	0	0	0	0	0	0	0	0	0	0	0	0	0	0	0
2-Oxobutyrate_Post-UK	0	0	0	0	0	0	0	0	0	0	0	0	0	0	0	0	0	0	0	0	0	0	0	0	0	0	0	0	0	0	0	0
Pentanoate_Post-UK	0	0	0	0	0	0	0	0	0	0	0	0	0	0	0	0	0	0	0	0	0	0	0	0	0	0	0	0	0	0	0	0
3-Methylbutanoate_Post-UK	0	1.65	0	0.472	0	0.683	2.89	1.1	1.34	3.81	0.571	0.682	0.476	0.958	0	1.01	1.46	1.66	1.35	0.572	2.74	0	0.784	0.869	2.33	3.13	0.686	0	1.16	0.263	1.8	0
Malonate_Post-UK	0.242	0.514	0.206	0.357	0.189	0.163	0.61	0.212	0.464	0.734	0.425	0.363	0.188	0.235	0.366	0.324	0.437	0.236	0.523	0.191	0.17	0.229	0.359	0.248	0.462	0.334	0.248	0.26	0.306	0.194	0.366	0.233
2-Hydroxyisobutyrate_Post-UK	0	0	0	0	0	0	0	0	0	0	0	0	0	0	0	0	0	0	0	0	0	0	0	0	0	0	0	0	0	0	0	0
2-Hydroxybutyrate_Post-UK	0.184	0	0	0.427	0.263	0.413	0.536	0.377	0	0.665	0.331	0.283	0.49	0.465	0.628	0.616	1.74	0.274	0.4	0.565	0.629	0.272	0	0.71	0.488	0.721	0.171	0.339	0.603	0.268	0	0.172
3-Hydroxybutyrate_Post-UK	0.769	4.82	1.01	1.56	1.17	2.25	2.15	1	0.979	2.18	1.66	1.91	1.23	1.59	3.12	3.68	2.72	0.992	1.93	1.38	1.71	1.26	0.886	1.68	2.81	3.98	1.02	0.934	2.84	0.912	0.703	0.815
(Methylthio)acetate_Post-UK	0	0	0	0	0	0	0	0	0	0	0	0	0	0	0	0	0	0	0	0	0	0	0	0	0	0	0	0	0	0	0	0
Glycerate_Post-UK	0.811	1.07	0.701	0.885	1.14	0.648	1.13	0.783	1.47	0.717	0	0.659	0	1.13	0.455	0.752	1.45	0.673	0.183	0.569	1.05	0.807	0.83	0.793	0.767	2.16	0.871	0	0.911	0.826	0.663	0.553
Pyrrole-2-carboxylate_Post-UK	0	0	0	0	0	0	0	0	0	0	0	0	0	0	0	0	0	0	0	0	0	0	0	0	0	0	0	0	0	0	0	0
Methyl sulfate_Post-UK	0	0	0	0	0	0	0	0	0	0	0	0	0	0	0	0	0	0	0	0	0	0	0	0	0	0	0	0	0	0	0	0
2-Furoate_Post-UK	0	0	0	0	0	0	0	0	0	0	0	0	0	0	0	0	0	0	0	0	0	0	0	0	0	0	0	0	0	0	0	0
Fumarate_Post-UK	0.442	0.485	0.279	0.395	0.444	0.327	0.487	0.495	0.419	0.677	0.467	0.593	0.45	0.629	1.97	0.367	0.542	0.555	0.431	0.481	0.466	0.576	0.263	0.398	0.513	0.573	0.462	0.5	0.275	0.482	0.308	0.469
2-Oxoisopentanoate_Post-UK	0	0.19	0	0	0	0	0	0	0	0	0	0.066	0	0.12	0.602	0	0.167	0	0	0	0.286	0	0	0.268	0.179	0.0574	0	0.114	0.22	0.182	0.0982	0.0504

4-Oxopentanoate_Post-UK	0.121	0	0	0	0	0.289	0.178	0	0	0.211	0.256	0	0.18	0.105	0	0.454	0	0.146	0	0	0.182	0.401	0	0	0.345	0.446	0.0889	0	0.7	0	0	0.0747
Hexanoate_Post-UK	0	0	0	0	0	0.42	0.591	0.552	0	2.43	0	0.182	0	0.518	0	0.598	0.467	1.04	0	0.507	0.78	0.151	0.302	0.509	1.18	1.48	0	0	0.683	0	0.778	0
Succinate_Post-UK	14.8	10.5	19.1	38.3	31.8	22.6	38	18.3	11.1	22.7	10.4	42.2	12	22.8	10.1	20.3	11.9	9.93	11	18.4	21.5	15.6	17	22.6	15	14.6	13.9	10.4	31.9	50.3	17.2	14
2-Hydroxypentanoate_Post-UK	0	0.303	0.32	0.562	0.389	0.314	0.334	0.271	0.227	0.897	0.57	0.207	0.261	0.302	0.347	0.977	0.976	0.373	0.451	0.292	0.457	0.343	0.337	0.33	0.939	0.879	0.215	0.21	1.1	0.206	0.273	0.256
Benzoate_Post-UK	0	0	0	0.26	0	0	0	0	0	0	0	0.193	0	0.0803	0	0	0.227	0	0	0	0	0	0	0	0	0.497	0	0	0	0	0	0
Nicotinate_Post-UK	0	0.291	0	0	0	0.404	0.239	0.428	0.355	0.296	0.299	0	0	0.29	0.396	0.202	0.256	0.273	0.173	0	0	0	0.19	0.237	0.288	0.734	0.358	0	0.529	0	0.168	0.073
1-Aminoethylphosphonate_Post-UK	0	0	0	0	0	0	0	0	0	0	0	0	0	0	0	0	0	0	0	0	0	0	0	0	0	0	0	0	0	0	0	0
2-Aminoethylphosphonate_Post-UK	0	0	0	0	0	0	0	0	0	0	0	0	0	0	0	0	0	0	0	0	0	0	0	0	0	0	0	0	0	0	0	0
Isethionate_Post-UK	0	0	0	0	0.118	0	0.0861	0	0.148	0.32	0	0.131	0	0.104	0	0	0.128	0	0	0	0	0	0	0.115	0	0.152	0	0	0.0624	0	0.128	0
Barbiturate_Post-UK	0	0	0	0	0	0	0	0	0	0	0	0	0	0	0	0	0	0	0	0	0	0	0	0	0	0	0	0	0	0	0	0
5-Oxoproline_Post-UK	1.17	3.02	1.79	2.05	3.28	1.62	2.01	1.41	2.3	3.18	1.54	1.33	1.6	2.93	3.52	1.1	2.35	1.81	2.09	1.13	2.19	2.11	1.72	2.07	2.53	3.18	1.5	1.46	1.7	3.46	1.01	1.56
Itaconate_Post-UK	0	0	0	0	0	0	0	0	0	0	0	0	0	0	0	0	0	0	0	0	0	0	0	0	0	0	0	0	0	0	0	0
Citraconate_Post-UK	0	0	0	0	0	0	0	0	0	0	0	0	0	0	0	0	0	0	0	0	0	0	0	0	0	0	0	0	0	0	0	0
4-Methyl-2-oxopentanoate_Post-UK	0.213	0.293	0	0.246	0.298	0.293	0.218	0.212	0.228	0.5	0.273	0.22	0.189	0.262	0.495	0.412	0.343	0.257	0.435	0.244	0.533	0.233	0.174	0.573	0.374	0.49	0	0.241	0.495	0.269	0.076	0.174
4-Oxohexanoate_Post-UK	0	0	0	0	0	0	0	0	0	0	0	0	0	0	0	0	0	0	0	0	0	0	0	0	0	0	0	0	0	0	0	0
4-Acetylbutyrate_Post-UK	0	0.111	0	0.108	0	0	0.0571	0	0	0	0	0	0	0	0	0.232	0.0748	0	0	0	0	0	0	0	0.153	0.272	0	0	0.211	0	0	0.0658
Heptanoate_Post-UK	0	0	0	0	0	0	0	0	0	0	0	0	0	0	0	0	0	0	0	0	0	0	0	0	0	0	0	0	0	0	0	0
N-acetyl-*β*-alanine_Post-UK	0	0.289	0	0.198	0.219	0	0.157	0.188	0	0.7	0	0.206	0	0.199	0.411	0	0.308	0	0	0	0.169	0.196	0.183	0.225	0	0.268	0.21	0	0.127	0.211	0.175	0
Glutarate_Post-UK	0	0	0	0	0	0	0	0	0	0	0	0	0	0	0	0	0	0	0	0	0	0	0	0	0	0	0	0	0	0	0	0
3-Ureidopropionate_Post-UK	0	0	0	0	0	0	0	0	0	0	0	0	0	0	0	0	0	0	0	0	0	0	0	0	0	0	0	0	0	0	0	0
2-Hydroxy-4-methylpentanoate_Post-UK	0	0.108	0	0.254	0.224	0.401	0.293	0.174	0.254	0	0.435	0.194	0.128	0.192	0	0.899	0	0.247	0	0.129	0	0.238	0.37	0.268	0.439	0.501	0.157	0	0.613	0	0	0.101
6-Hydroxyhexanoate_Post-UK	0	0	0	0.19	0	0	0	0	0	0	0	0.0958	0	0	0	3.47	0	0	0	0	0	0	0	0	0.333	0.494	0	0	0.521	0	0	0
Malate_Post-UK	1.69	2.57	0.54	1.63	1.6	1.38	2.2	1.74	2.35	2.46	1.6	4.14	1.65	3.3	11.2	1.17	3.96	2.54	1.32	1.98	1.63	2.43	2.19	1.96	3.48	2.51	2.76	2.86	1.49	1.8	2.13	3.11
Threonate_Post-UK	0.453	0.685	0.333	0.489	1.08	0	0	0.541	0.594	1.04	0.421	0.877	0.329	1.02	1.81	0.478	1.3	0.478	0	0.44	0	0.452	0.765	1.02	0	0	0.365	0.374	0	0.752	0.702	0.44
o-Hydroxybenzoate_Post-UK	0	0	0	0	0	0	0	0	0	0	0	0	0	0	0	0	0.051	0	0	0	0	0	0	0	0.0382	0.326	0.266	0	0	0	0	0.0825
p-Hydroxybenzoate_Post-UK	0	0	0	0	0	0.321	0	0	0.288	0	0.301	0.813	0	0	0.593	0	0	0	0	0.234	0	0	0	0.316	0.427	0.128	0	0	0	0	0	0
m-Hydroxybenzoate_Post-UK	0	0	0	0	0	0	0	0	0	0	0	0	0	0	0	0	0	0	0	0	0	0	0	0	0	0	0	0	0	0	0	0
Phosphonoacetate_Post-UK	0	0	0	0	0	0	0	0	0	0	0	0	0	0	0	0	0	0	0	0	0	0	0	0	0	0	0	0	0	0	0	0
Carbamoylphosphate_Post-UK	0	0	0	0	0	0	0	0	0	0	0	0	0	0	0	0	0	0	0	0	0	0	0	0	0	0	0	0	0	0	0	0
Ethanolamine phosphate_Post-UK	28.8	83.5	15.7	30.7	41	13.7	30.2	27.9	19.2	37.3	19.4	17.6	31.9	33.3	72	19.2	34.3	14.7	19.3	25.7	21.8	14.2	30.8	23.8	25.7	6.08	19	26.2	14.8	39.6	42.9	13.4
2-Thiopheneacetate_Post-UK	0	0	0	0	0	0	0	0	0	0	0	0	0	0	0	0	0	0	0	0	0	0	0	0	0	0	0	0	0	0	0	0
Octanoate_Post-UK	0	0	0	0	0	0	0	0	0	0	0	0	0	0	0	0	0	0	0	0	0	0	0	0	0	0	0	0	0	0	0	0
2-Oxoglutarate_Post-UK	0	0	0	0	0	0	1.29	0	1	0.751	0	1.27	0	0.255	0	0	0	0	0	0	1.13	0	0.423	0	0.21	0	0	0	0	0.849	0.462	0
Adipate_Post-UK	0	0	0	0	0	0	0	0	0	0	0	0	0	0	0	0	0	0	0	0	0	0	0	0	0	0	0	0	0	0	0	0
threo-*β*-methylaspartate_Post-UK	0	0	0	0	0	0	0	0	0	0	0	0	0	0	0	0	0	0	0	0	0	0	0	0	0	0	0	0	0	0	0	0
4-Methylthio-2-oxobutyrate_Post-UK	0	0	0	0	0	0	0	0	0	0	0	0	0	0	0	0	0	0	0	0	0	0	0	0	0	0	0	0	0	0	0	0
2-Hydroxyglutarate + Citramalate_Post-UK	0.216	0	0.369	0.774	0.854	0.48	0.421	0.301	0.413	1.2	0.504	0.554	0.387	0.557	0	0.686	0	0.489	0.474	0.199	0.599	0.46	0.327	0	0.586	0.538	0	0	0.583	0.932	0	0.267
trans-Cinnamate_Post-UK	0.35	0.499	0.605	0.349	0.323	0.415	0.619	0.382	0.553	0.778	0.348	0.79	0.226	0.574	1.73	0.296	0.538	0.383	0.392	0	0.588	0.472	0.364	0.493	0.508	0.715	0.417	0.253	0.355	0.51	0.398	0.193
Tartrate_Post-UK	0	0	0	0	0	0	0	0	0	0	0	0	0	0	0	0	0	0	0	0	0	0	0	0	0	0	0	0	0	0	0	0
Benzoylformate_Post-UK	0	0	0	0	0	0	0	0	0	0	0	0	0	0	0	0	0	0	0	0	0	0	0	0	0	0	0	0	0	0	0	0
3-Phenylpropionate_Post-UK	0.719	0.361	0.627	0.635	0.513	0.968	0.673	1	0.584	3.64	0.775	0.559	0.492	1.04	0.755	4.26	0.899	1.94	1.6	1.34	1.97	0.583	0.627	1.13	2.86	5.2	0.338	0.361	4.98	0.621	1.02	0.607
2-Hydroxyphenylacetate_Post-UK	0	0	0	0	0	0	0	0	0	0	0	0	0	0	0	0	0	0	0	0	0	0	0	0	0	0	0	0	0	0	0	0
p-Hydroxyphenylacetate_Post-UK	0	14.6	2.65	0.181	1.83	0.832	11	3.49	5.18	0.958	1.45	0.585	1.69	0	0	1.71	0	2.64	2.09	1.53	0	3.35	5.98	0.924	0.801	7	4.71	0.681	1.44	0	4.02	0
Cysteine sulfinate_Post-UK	0	0	0	0	0	0	0.313	0	0.447	0.435	0.345	0	0	0.28	0	0	0.267	0.446	0	0	0	0	0	0	0.356	0	0	0.244	0	0	0	0
2,5-Dihydroxybenzoate_Post-UK	0.589	0	0	0	0.593	0	0	0	0	0	0.993	0	0	0	0	0	0.362	0.6	0	0	0.726	0	0.962	0	0.533	0.711	0	0	0	1.19	0	0
Orotate_Post-UK	0	0	0	0	0	0	0	0	0	0.246	0	0	0	0	0	0	0	0	0	0	0	0	0	0	0	0.254	0	0	0	0	0	0
2,4-Dihydroxypyrimidine-5-carboxylate_Post-UK	0	0	0	0	0	0	0	0	0	0	0	0	0	0	0	0	0	0	0	0	0	0	0	0	0	0	0	0	0	0	0	0
Dihydroorotate_Post-UK	0	0	0	0	0	0	0	0	0	0	0	0	0	0	0	0	0	0	0	0	0	0	0	0	0	0	0	0	0	0	0	0
2-Oxooctanoate_Post-UK	0	0	0	0	0	0	0	0	0	0	0	0	0	0	0	0	0	0	0	0	0	0	0	0	0	0	0	0	0	0	0	0
Pelargonate_Post-UK	0	0	0	0	0	0	0	0	0	0	0	0	0	0	0	0	0	0.0213	0	0	0	0	0	0	0	0	0	0	0	0	0	0
2-Oxoadipate_Post-UK	0	0	0	0	0	0	0	0	0	0	0	0	0	0	0	0	0	0	0	0	0	0	0	0	0	0	0	0	0	0	0	0
Pimelate_Post-UK	0	0	0	0	0	0	0	0	0	0	0	0	0	0	0	0	0	0	0	0	0	0	0	0	0	0	0	0	0	0	0	0
2-Hydroxyoctanoate_Post-UK	0	0	0	0	0	0	0	0	0	0	0	0	0	0	0	0	0	0	0	0	0	0	0	0	0	0	0	0	0	0	0	0
N-Formylaspartate_Post-UK	0	0	0	0	0	0	0	0	0	0	0	0	0	0	0	0	0	0	0	0	0	0	0	0	0	0	0	0	0	0	0	0
3-Hydroxy-3-methylglutarate_Post-UK	0.0544	0.0575	0	0.108	0.0899	0.0305	0.0285	0.086	0.0703	0.174	0.0961	0.0235	0	0	0	0.108	0.0526	0.0503	0.0731	0.102	0.0464	0.0913	0.0323	0.0197	0.0854	0.118	0.0518	0	0.0434	0.0914	0	0.0445
Phenylpyruvate_Post-UK	0	0	0	0	0	0	0	0	0	0	0	0	0	0	0	0	0	0	0	0	0	0	0	0	0	0	0	0	0	0	0	0
p-Coumarate_Post-UK	0	0	0	0	0	0	0	0	0	0	0	0	0	0	0	0	0	0	0	0	0	0	0	0	0	0	0	0	0	0	0	0
o-Coumarate_Post-UK	0	0	0	0	0	0	0	0	0	0	0	0	0	0	0	0	0	0	0	0	0	0	0	0	0	0	0	0	0	0	0	0
Terephthalate_Post-UK	0	0	0	0.4	0	0	0	0	0	0	0	0	0	0	0	0	0	0.199	0	0	0	0	0	0	0	0	0	0	0	0	0	0
Phthalate_Post-UK	0.129	0.0644	0.064	0.109	0.104	0.0962	0.103	0.104	0.144	0.144	0.0997	0.0867	0.0836	0.0782	0.043	0.217	0.0899	0.127	0.0843	0.0819	0.0775	0.0867	0.113	0.0952	0.178	0.138	0.0771	0.0842	0.0905	0.108	0.054	0.168
3-Phenyllactate_Post-UK	0.19	0	0.371	0.452	0.424	0.361	0.417	0.219	0.315	0.997	0.361	0.369	0.192	0.339	0.357	0.322	0.152	0.365	0.322	0.133	0.379	0.206	0.399	0.313	0.344	0.404	0.158	0.106	0.328	0.304	0.162	0.103
3-(2-Hydroxyphenyl)propionate_Post-UK	0	0.055	0	0	0	0	0	0	0	0	0	0	0	0	0	0	0	0	0	0	0	0	0	0	0	0	0	0	0	0	0	0
3-(4-Hydroxyphenyl)propionate_Post-UK	1.15	8.23	2.01	0.885	0.712	0.625	8.56	3	3.84	3.02	2.19	1.37	2.34	1.47	0.422	3	0.442	1.57	1.75	1.12	1.54	1.73	5.06	1.48	2.82	2.68	2.71	0	1.88	0.515	6.8	0.188
2,3-Pyridinedicarboxylate_Post-UK	0	0	0	0	0	0	0	0	0	0	0	0	0	0	0	0	0	0	0	0	0	0	0	0	0	0	0	0	0	0	0	0
PEP_Post-UK	0.13	0.588	0	0	0.179	0.13	0.163	0.701	0.218	0	0.374	0.328	0.224	0.263	2.97	0.0832	0.997	0.195	0.175	0.172	0.263	0.273	0	0.289	0.328	0.274	0.275	0.338	0.204	0.133	0.244	0.301
Urate_Post-UK	70.8	99.4	74.9	43.5	109	72.7	84.3	32.8	62.7	125	105	92.9	36.9	69.2	238	30.7	136	70.3	145	69.7	84.8	44.7	74.2	69.4	77	45.5	58.1	55.2	82.9	128	68.7	31.2
4-Hydroxymandelate_Post-UK	0	8.21	0	0	1.08	0	7.29	1.31	2.86	0	0.722	0.738	0.896	1.9	0	1.14	0.778	1.31	1.59	0.991	0	1.37	3.14	0.824	0.727	3.78	2.42	0	0.918	0	2.4	0
Cysteate_Post-UK	0	0	0	0.112	0	0.149	0	0	0	0.267	0	0	0	0	0	0	0	0.116	0	0	0.144	0	0	0	0	0.214	0	0	0	0	0	0
2-Amino-3-phosphonopropionate_Post-UK	0	0	0	0	0	0	0	0	0	0	0	0	0	0	0	0	0	0	0	0	0	0	0	0	0	0	0	0	0	0	0	0
DHAP_Post-UK	0	0	0	0	0.203	0	0	0.56	0	0.447	0	0.205	0.199	0.242	0	0	0.347	0.186	0.231	0	0.346	0	0	0.268	0.244	0.272	0	0.31	0.303	0.208	0.201	0
G3P_Post-UK	0	0	0	0	0	0	0	0	0	0	0	0	0	0	0	0	0	0	0	0	0	0	0	0	0	0	0	0	0	0	0	0
Glycerophosphate_Post-UK	3.89	6.37	2.47	6.21	6.57	3.64	4.92	4.96	3.82	14.7	6.07	5.83	4.81	6.05	12.8	2.55	11.9	4.98	5.35	5.24	7.69	4.06	4.83	7.36	5.02	6.46	3.55	4.8	4.36	7.41	5.73	2.91
Decanoate_Post-UK	0	0	0	0	0	0	0	0	0	0	0	0	0	0	0	0	0	0	0	0	0	0	0	0	0	0	0	0	0	0	0	0
2-Quinolinecarboxylate_Post-UK	0	0	0	0	0	0	0	0	0	0	0	0	0	0	0	0	0	0	0	0	0	0	0	0	0	0	0	0	0	0	0	0
N-Acetylleucine_Post-UK	0	0	0	0	0	0	0	0	0	0	0	0	0	0	0	0	0	0	0	0	0	0	0	0	0	0	0	0	0	0	0.0292	0
Phenyl phosphate_Post-UK	0	0	0	0	0	0	0	0	0	0	0	0	0	0	0	0	0	0	0	0	0	0	0	0	0	0	0	0	0	0	0	0
trans-Aconitate_Post-UK	0	0	0	0	0	0	0	0	0	0	0	0	0	0	0	0	0	0	0	0	0	0	0	0	0	0	0	0	0	0	0	0
cis-Aconitate_Post-UK	0.0796	0.19	0.0331	0.0435	0.147	0.0413	0.176	0.0188	0.169	0.165	0	0.134	0.0694	0.121	0.47	0.0914	0.132	0.0701	0.0455	0.0824	0.0607	0.0396	0.154	0.132	0.0725	0.114	0.0298	0.183	0	0.154	0.0836	0.0426
Shikimate_Post-UK	0	0	0	0	0	0	0	0	0	0.369	0	0	0	0.17	0	0	0	0	0	0	0	0	0	0	0	0.518	0	0	0.174	0	0	0
N-Acetylaspartate_Post-UK	0.352	0.192	0.587	0.47	2.29	0.17	1.49	0.226	0.519	0.785	0.154	0.758	0.221	0.414	0.894	0.373	0.374	0.493	0.24	0.673	0.3	0.247	0.824	0.737	0.404	1.16	0.162	1.46	0.167	1.89	0.284	0.218
Carbamoylaspartate_Post-UK	0	0	0	0	0	0	0	0	0	0.198	0	0	0	0	0	0	0	0	0	0	0	0	0	0	0	0	0	0	0	0	0	0
Allantoate_Post-UK	0	0	0	0	0	0	0	0	0	0	0	0	0	0	0	0	0	0	0	0	0	0	0	0	0	0	0	0	0	0	0	0
2-Isopropylmalate_Post-UK	0.124	0.155	0	0.0969	0.109	0.031	0.097	0.0333	0.175	0.478	0.289	0.121	0.115	0.218	0.055	0.147	0.27	0.0689	0.19	0.134	0.0571	0.112	0.0752	0.0696	0.393	0.243	0.0897	0.0567	0.0583	0.156	0.0565	0.128
N-Formylmethionine_Post-UK	0	0	0	0	0	0	0	0	0	0	0	0	0	0	0.142	0	0	0	0	0	0	0	0	0	0	0	0	0	0	0	0	0
Hippurate_Post-UK	0	0	0	0	0	0	0	0	0	0	0	0	0	0	0	0	0	0	0	0	0	0	0	0	0	0	0	0	0	0	0	0
Homovanillate_Post-UK	0.42	1.57	0	0.764	1.07	0.501	2.29	0.371	0.763	1.83	0.761	0.892	0.51	1.23	0	0.547	0.394	0.625	0.89	0	0	0.772	1.35	0.917	0.749	1.11	0	0	0.697	0.887	0.734	0.296
4-Pyridoxate_Post-UK	0	0	0	0	0	0	0	0	0	0	0	0	0	0	0	0	0	0	0	0	0	0	0	0	0	0	0	0	0	0	0	0
Serine O-sulfate_Post-UK	0	0	0	0	0	0	0	0	0	0	0	0	0	0	0	0	0	0	0	0	0	0	0	0	0	0	0	0	0	0	0	0
O-Phosphoserine_Post-UK	0	0	0	0	0.079	0	0.107	0	0	0.166	0	0.0806	0	0	0.0863	0	0	0	0	0	0.159	0	0	0.101	0	0.255	0.0593	0	0.126	0.208	0	0
3PG + 2PG_Post-UK	0.389	2.36	0	0	0.582	0.524	0.63	2.1	0.711	0	1.15	1.1	0.768	0.8	12.8	0.294	3.09	0.7	0	0.691	0.982	1.15	0.309	1.13	0.828	0.699	0.957	1.31	0.533	0.579	1.45	1.07
Undecanoate_Post-UK	0	0	0	0	0	0	0	0	0	0	0	0	0	0	0	0	0	0	0	0	0	0	0	0	0	0	0	0	0	0	0	0
Azelate_Post-UK	0.16	0.222	0	0.157	0.136	0.108	0.266	0.133	0.201	0.163	0.149	0.124	0.129	0.172	0.107	0.104	0.107	0.109	0.126	0.107	0.156	0.149	0.197	0.105	0.0739	0.149	0.165	0.148	0.112	0.308	0.104	0.117

10-Hydroxydecanoate_Post-UK	0	0	0	0	0	0	0	0	0	0	0	0	0	0	0	0	0	0	0	0	0	0	0	0	0	0	0	0	0	0	0	0
N-Acetylglutamate_Post-UK	0	0.0976	0	0.0643	0.102	0	0.0768	0	0	0.171	0.0871	0.07	0	0.0656	0	0	0.0993	0.0605	0.0987	0	0	0.0631	0.0667	0.0494	0	0.0916	0	0.0432	0.0481	0.137	0.067	0
N-Carbamylglutamate_Post-UK	0	0	0	0	0	0	0	0	0	0	0	0	0	0	0	0	0	0	0	0	0	0	0	0	0	0	0	0	0	0	0	0
N-Acetylmethionine_Post-UK	0	0	0	0	0	0	0	0	0	0	0	0	0	0	0	0	0	0	0	0	0	0	0	0	0	0	0	0	0	0	0	0
Isocitrate_Post-UK	0.183	0.301	0	0.174	0.298	0	1.84	0	0	0.259	0	0.225	0.17	0.186	0.562	0	0.236	0.132	0	0	0	0	0.466	0.282	0.3	0.372	0	0.994	0	0.298	0.504	0
Citrate_Post-UK	1.78	3.13	0.549	0.55	3.49	0.747	3.37	0.6	1.92	1.42	0.908	2.76	1.2	2.55	15.2	1.35	2.97	1.28	0.665	1.63	1.43	0.499	3.06	3.54	2.2	3.44	0.233	4.36	1.2	3.14	1.11	0.613
Quinate_Post-UK	0.997	0	0	0	0.304	0.168	0	0	0	0.631	0	0	0	1.03	0	0.157	0.206	0	0.423	0	0.636	0.212	0	0	0	0.606	0	0	0	0	0	0
Phenaceturate_Post-UK	0	0	0	0	0	0	0	0	0	0	0	0	0	0.0669	0	0	0	0	0	0	0	0	0	0.125	0	0	0	0	0	0	0	0
Glucuronate_Post-UK	0	0.157	0	0	0	0	0.223	0	0	0	0	0	0	0	0	0	0	0	0	0	0	0	0	0	0	0	0	0	0	0	0	0
trans-4-Hydroxy-3-methoxycinnamate_Post-UK	0	0	0	0	0	0	0	0	0	0	0	0	0	0	0	0	0	0	0	0	0	0	0	0	0	0	0	0	0	0	0	0
o-Hydroxyhippurate_Post-UK	0	0	0	0	0	0	0	0	0	0	0	0	0	0	0	0	0	0	0	0	0	0	0	0	0	0	0	0	0	0	0	0
Gluconate_Post-UK	0	0	0	0	0	0	0	0	0	0	0	0	0	0	0	0	0	0	0	0	0	0	0	0	0	0	0	0	0	0	0	0
Syringate_Post-UK	0	0.213	0	0.405	0	0.41	0	0.417	0.346	1.04	0.387	0.318	0.396	0.405	0.38	0.401	0.363	0.369	0	0.428	0	0.287	0.475	0	0.5	0.436	0.46	0.359	0.425	0	0	0.469
4-Hydroxy-3-methoxymandelate_Post-UK	0	0	0	0.277	0	0	0.847	0	0	0	0	0.346	0	0.389	0	0	0	0	0	0	0	0.217	0.578	0.211	0.383	0.582	0.18	0	0	0	0.648	0
Dodecanoate_Post-UK	0	0	0	0	0	0	0	0	0	0	0	0	0	0	0	0	0	0	0	0	0	0	0	0	0	0	0	0	0	0	0	0
Cysteine S-sulfate_Post-UK	0	0	0	0	0	0	0	0	0	0	0	0	0	0	0	0	0	0	0	0	0	0	0	0	0	0	0	0	0	0	0	0
Sebacate_Post-UK	0	0	0	0	0	0	0.037	0.0223	0	0	0	0	0	0.0187	0	0	0	0	0.0297	0	0	0	0	0	0	0	0	0.0185	0.0137	0.0266	0	0
3-Indolebutyrate_Post-UK	0	0	0	0	0	0	0	0	0	0	0	0	0	0	0	0	0	0	0	0	0	0	0	0	0	0	0	0	0	0	0	0
Xanthurenate_Post-UK	0	0	0	0	0	0	0	0	0	0	0	0	0	0	0	0	0	0	0	0	0	0	0	0	0	0	0	0	0	0	0	0
N-Acetylphenylalanine_Post-UK	0	0	0	0	0	0	0	0	0	0	0	0	0	0	0	0	0	0	0	0	0	0	0	0	0	0	0	0	0	0	0	0
Benzylsuccinate_Post-UK	0	0	0	0	0	0	0	0	0	0	0	0	0	0	0	0	0	0	0	0	0	0	0	0	0	0	0	0	0	0	0	0
Saccharate_Post-UK	0	0	0	0	0	0	0	0	0	0	0	0	0	0	0	0	0	0	0	0	0.0634	0	0	0	0	0	0	0	0	0	0	0
Mucate_Post-UK	0	0	0	0	0	0	0	0	0	0	0	0	0	0	0	0	0	0	0	0	0	0	0	0	0.103	0	0	0	0	0	0	0
3-Indoxyl sulfate_Post-UK	0	0	0	0	0	0	0	0	0	0	0	0	0	0	0	0	0	0	0	0	0	0	0	0	0	0	0	0	0	0	0	0
2-Deoxyribose 1-phosphate_Post-UK	0	0	0	0	0	0	0	0	0	0	0	0	0	0	0	0	0	0	0	0	0	0	0	0	0	0	0	0	0	0	0	0
Pantothenate_Post-UK	0	0.209	0	0	0	0	0.0748	0	0	0	0	0	0	0	0	0	0	0	0	0	0	0.0427	0	0.225	0	0	0.0756	0	0	0	0	0
Sinapate_Post-UK	0	0	0	0	0	0	0	0	0	0	0	0	0	0	0	0	0	0	0	0	0	0	0	0	0	0	0	0	0	0	0	0
Porphobilinogen_Post-UK	0	0	0	0	0	0	0	0	0	0	0	0	0	0	0	0	0	0	0	0	0	0	0	0	0	0	0	0	0	0	0	0
Ru5P_Post-UK	0.606	2.48	0.284	0.419	0.751	0.805	0.961	1.15	0.659	1.09	0.909	0.861	0.894	0.623	3.09	0.422	0.809	0.926	1.66	0.729	1.36	1.12	0.463	1.29	1.11	0.676	0.709	0.943	0.451	0.578	1.74	0.696
R5P_Post-UK	0	0	0	0	0	0	0	0	0	0	0	0	0	0	0	0	0	0	0	0	0	0	0	0	0	0	0	0	0	0	0	0
Dodecanedioate_Post-UK	0	0	0	0	0	0	0	0	0	0	0	0	0	0	0	0.0447	0	0	0	0	0	0	0	0	0	0	0	0	0	0	0	0
2-Deoxyglucose 6-phosphate_Post-UK	0	0	0	0	0	0	0	0	0	0	0	0	0	0	0	0	0	0	0	0	0	0	0	0	0	0	0	0	0	0	0	0
Biotin_Post-UK	0	0.339	0	0	0	0	0.311	0	0	0.672	0	0.28	0	0.273	0	0.139	0.28	0.202	0	0.237	0	0	0.286	0	0.296	0.19	0	0.206	0	0	0	0
Glucosamine 6-phosphate_Post-UK	0	0	0	0	0	0	0	0	0	0	0	0	0	0	0	0	0	0	0	0	0	0	0	0	0	0	0	0	0	0	0	0
G1P_Post-UK	0.189	0.347	0.135	0.178	0.233	0.189	0.206	0.384	0.187	0.539	0.618	0.576	0.216	0.263	0.721	0.0763	0.801	0.297	0.378	0.238	0.402	0.29	0.138	0.372	0.329	0.684	0.194	0.248	0.482	0.248	0.278	0.143
F6P_Post-UK	0.261	0.625	0.158	0.156	0.403	0.253	0.198	0.718	0.268	0.728	0.417	0.394	0.372	0.41	0.816	0.159	0.836	0.387	0.344	0.309	0.62	0.484	0.169	0.444	0.502	0.633	0.293	0.431	0.458	0.343	0.24	0.278
G6P_Post-UK	0.916	2.86	0.56	0.698	1.64	1.2	1	2.81	1.15	3.47	1.66	1.59	1.35	1.87	5.19	0.596	4.43	1.85	1.42	1.08	2.74	1.91	0.641	1.76	1.67	2.52	1.33	1.62	1.61	1.08	1.14	0.925
Sorbitol 6-phosphate_Post-UK	0	0	0	0	0	0	0	0	0	0	0	0	0	0	0	0	0	0	0	0	0	0	0	0	0	0	0	0	0	0	0	0
2,3-DPG_Post-UK	0	0	0	0	0	0	0	0	0	0	0	0	0	0	0	0	0	0	0	0	0	0	0	0	0	0	0	0	0	0	0	0
Galacturonate 1-phosphate_Post-UK	0	0.0524	0	0.131	0.121	0	0.0833	0	0	0.202	0.0966	0.161	0	0.082	0.187	0	0.119	0.12	0.0865	0.114	0.105	0	0	0.15	0.114	0.122	0	0.188	0.105	0.409	0.12	0
6-Phosphogluconate_Post-UK	0	0.0733	0.103	0.112	0.262	0.119	0.101	0.12	0.119	0.223	0	0.17	0	0.133	0.137	0	0	0	0.162	0.101	0.199	0	0	0.571	0.0655	0.342	0.0657	0.0719	0.353	0.196	0.0637	0
S7P_Post-UK	0.297	0.614	0	0.262	0	0	0	0.74	0.518	0.664	0	0	0	0.326	1.25	0	0.906	0.415	0.424	0	0.374	0.215	0	0.351	0.493	0.949	0.32	0.191	0.254	0	0	0.251
Argininosuccinate_Post-UK	0	0	0	0	0	0	0	0	0	0	0	0	0	0	0	0	0	0	0	0	0	0	0	0	0	0	0	0	0	0	0	0
N-Acetylmuramate_Post-UK	0	0	0	0.423	0.738	0	0	0	0	0	0	0	0	0	0	0	0	0	0	0	0	0	0	0	0.178	0	0	0	0	0	0	0
N-Acetylglucosamine 1-phosphate_Post-UK	0	0.179	0	0	0.163	0.1	0	0.148	0.125	0.323	0	1.13	0	0.131	0	0.119	0.312	0.0857	0	0	0	0	0	0.0971	0.134	0.214	0	0.0873	0.336	0	0	0.0626
N-Acetylglucosamine 6-phosphate_Post-UK	0	0	0	0	0.148	0.0979	0	0	0	0	0	0.0733	0	0.11	0.354	0	0.24	0.119	0	0	0	0	0	0	0.118	0.171	0.0854	0.162	0.103	0	0	0.0587
2',3'-cCMP_Post-UK	0	0	0	0	0	0	0	0	0	0	0	0	0	0	0	0	0	0	0	0	0	0	0	0	0	0	0	0	0	0	0	0
dCMP_Post-UK	0	0	0	0	0	0	0	0	0	0	0	0	0	0	0	0	0	0	0	0	0	0	0	0	0	0	0	0	0	0	0	0
dUMP_Post-UK	0	0	0	0	0	0	0	0	0	0	0	0	0	0	0	0	0	0	0	0	0	0	0	0	0	0	0	0	0	0	0	0
N-Acetylneuraminate_Post-UK	41.4	19.4	5.36	6.99	4.85	10.1	5.16	9.33	15.9	72.9	45.3	42.7	4.72	22.7	6.38	32.8	7.36	27	19.9	12.5	40.7	11.1	19.3	24.7	3.96	30.1	6.27	5.83	36.6	10.1	28.1	8.49
Ribulose 1,5-diphosphate_Post-UK	0	0	0	0	0	0	0	0	0	0	0	0	0	0	0	0	0	0	0	0	0	0	0	0	0	0	0	0	0	0	0	0
3',5'-Cyclic dAMP_Post-UK	0	0	0	0	0	0	0	0	0	0	0	0	0	0	0	0	0	0	0	0	0	0	0	0	0	0	0	0	0	0	0	0
dTMP_Post-UK	0	0	0	0	0	0	0	0	0	0	0	0	0	0	0	0	0	0	0	0	0	0	0	0	0	0	0	0	0	0	0	0
CMP_Post-UK	0	0.643	0.214	0.105	0.379	0	0.156	0.343	0.213	0	0.346	0	0.2	0.277	1.25	0	0.587	0.304	0.157	0.192	0.2	0.262	0.0844	0.256	0.396	0.33	0.386	0.507	0.116	0	0	0.338
UMP_Post-UK	0	0.445	0.198	0.163	0.271	0.109	0.252	0.484	0.121	0.167	0.397	0.156	0.31	0.26	1.16	0	0.655	0.353	0.283	0.239	0.358	0.25	0.174	0.485	0.557	0.442	0.225	0.487	0.287	0	0	0.27
cAMP_Post-UK	0	0	0	0	0	0	0	0	0	0	0	0	0	0	0	0	0	0	0	0	0	0	0	0	0	0	0	0	0	0	0	0
cIMP_Post-UK	0	0	0	0	0	0	0	0	0	0	0	0	0	0	0	0	0	0	0	0	0	0	0	0	0	0	0	0	0	0	0	0
dAMP_Post-UK	0	0	0	0	0	0	0	0	0	0	0	0	0	0	0	0	0	0	0	0	0	0	0	0	0	0	0	0	0	0	0	0
F1,6P_Post-UK	0.0507	0.0838	0	0.0841	0	0	0.081	0.121	0	0.195	0	0	0.0712	0.0554	0.154	0	0.0643	0	0	0	0	0.0697	0.0408	0	0	0.0955	0	0	0	0.138	0.0648	0
cGMP_Post-UK	0	0	0	0	0	0	0	0	0	0	0	0	0	0	0	0	0	0	0	0	0	0	0	0	0	0	0	0	0	0	0	0
3'-AMP_Post-UK	0	0.385	0.0535	0	0.179	0	0	0.244	0	0	0	0	0	0	0.644	0	0.395	0	0	0	0	0	0	0	0	0	0.179	0.221	0	0	0	0.233
AMP_Post-UK	0.124	0.926	0.189	0	0.783	0.0993	0.441	1.95	0.17	0	0.138	0	0.738	0.136	2.42	0	0.417	0.167	0	0	0.276	0.266	0	0.414	0.313	0.197	0	0.927	0.0934	0	0.371	0.885
IMP_Post-UK	0	0	0	0	0	0	0	0.304	0	0	0	0	0	0	0.235	0	0	0	0	0	0	0	0	0	0	0	0	0	0	0	0	0
Prostaglandin E2_Post-UK	0	0	0	0	0	0	0	0	0	0	0	0	0	0	0	0	0	0	0	0	0	0	0	0	0	0	0	0	0	0	0	0
Prostaglandin F2α _Post-UK	0	0	0	0	0	0	0	0	0	0	0	0	0	0	0	0	0	0	0	0	0	0	0	0	0	0	0	0	0	0	0	0
GMP_Post-UK	0	0	0	0	0	0	0	0.408	0	0	0	0	0.195	0	0	0	0	0	0	0	0	0	0	0	0	0	0	0.243	0	0	0	0.128
XMP_Post-UK	0	0	0	0	0	0	0	0	0	0	0	0	0	0	0	0	0	0	0	0	0	0	0	0	0	0	0	0	0	0	0	0
Orotidine 5'-monophosphate_Post-UK	0	0	0	0	0	0	0	0	0	0	0	0	0	0	0	0	0	0	0	0	0	0	0	0	0	0	0	0	0	0	0	0
Digalacturonate_Post-UK	0	0	0	0	0	0	0	0	0	0	0	0	0	0	0	0	0	0	0	0	0	0	0	0	0	0	0	0	0	0	0	0
NADPH_Post-UK	0	0	0	0	0	0	0	0	0	0	0	0	0	0	0	0	0	0	0	0	0	0	0	0	0	0	0	0	0	0	0	0
CoA_Post-UK	0	0	0	0	0	0	0	0	0	0	0	0	0	0	0	0	0	0	0	0	0	0	0	0	0	0	0	0	0	0	0	0
dCDP_Post-UK	0	0	0	0	0	0	0	0	0	0	0	0	0	0	0	0	0	0	0	0	0	0	0	0	0	0	0	0	0	0	0	0
dUDP_Post-UK	0	0	0	0	0	0	0	0	0	0	0	0	0	0	0	0	0	0	0	0	0	0	0	0	0	0	0	0	0	0	0	0
PRPP_Post-UK	0	0	0	0	0	0	0	0	0	0	0	0	0	0	0	0	0	0	0	0	0	0	0	0	0	0	0	0	0	0	0	0
dTDP_Post-UK	0	0	0	0	0	0	0	0	0	0	0	0	0	0	0	0	0	0	0	0	0	0	0	0	0	0	0	0	0	0	0	0
CDP_Post-UK	0	0	0	0	0	0	0	0	0	0	0	0	0	0	0	0	0	0	0	0	0	0	0	0	0	0	0	0	0	0	0	0
UDP_Post-UK	0	0	0	0	0	0	0	0	0	0	0	0	0	0	0	0	0	0	0	0	0	0	0	0	0	0	0	0	0	0	0	0
Acetyl CoA_Post-UK	0	0	0	0	0	0	0	0	0	0	0	0	0	0	0	0	0	0	0	0	0	0	0	0	0	0	0	0	0	0	0	0
Cholate_Post-UK	0	0	0	0	0	0	0	0	0	0	0	0	0	0	0	0	0	0	0	0	0	0	0	0	0	0	0	0	0	0	0	0
dADP_Post-UK	0	0	0	0	0	0	0	0	0	0	0	0	0	0	0	0	0	0	0	0	0	0	0	0	0	0	0	0	0	0	0	0
P1, P4-Di(adenosine-5') tetraphosphate_Post-UK	0	0	0	0	0	0	0	0	0	0	0	0	0	0	0	0	0	0	0	0	0	0	0	0	0	0	0	0	0	0	0	0
Isobutyryl CoA_Post-UK	0	0	0	0	0	0	0	0	0	0	0	0	0	0	0	0	0	0	0	0	0	0	0	0	0	0	0	0	0	0	0	0
Trehalose 6-phosphate_Post-UK	0	0	0	0	0	0	0	0.308	0	0	0	0	0	0	1.8	0	0	0	0	0	0	0	0	0	0	0	0.13	0	0.0969	0	0	0
Malonyl CoA_Post-UK	0	0	0	0	0	0	0	0	0	0	0	0	0	0	0	0	0	0	0	0	0	0	0	0	0	0	0	0	0	0	0	0
Adenosine 5'-phosphosulfate_Post-UK	0	0	0	0	0	0	0	0	0	0	0	0	0	0	0	0	0	0	0	0	0	0	0	0	0	0	0	0	0	0	0	0
Adenosine 3',5'-diphosphate_Post-UK	0	0	0	0	0	0	0	0	0	0	0	0	0	0	0	0	0	0	0	0	0	0	0	0	0	0	0	0	0	0	0	0
ADP_Post-UK	0	0	0	0	0	0	0	0	0	0	0	0	0	0	0.0663	0	0	0	0	0	0	0	0	0	0	0	0	0	0	0	0	0
IDP_Post-UK	0	0	0	0	0	0	0	0	0	0	0	0	0	0	0	0	0	0	0	0	0	0	0	0	0	0	0	0	0	0	0	0
Succinyl CoA_Post-UK	0	0	0	0	0	0	0	0	0	0	0	0	0	0	0	0	0	0	0	0	0	0	0	0	0	0	0	0	0	0	0	0
Folate_Post-UK	0	0	0	0	0	0	0	0	0	0	0	0	0	0	0	0	0	0	0	0	0	0	0	0	0	0	0	0	0	0	0	0
GDP_Post-UK	0	0	0	0	0	0	0	0	0	0	0	0	0	0	0	0	0	0	0	0	0	0	0	0	0	0	0	0	0	0	0	0
Adenylosuccinate_Post-UK	0	0	0	0	0	0	0	0	0	0	0	0	0	0	0	0	0	0	0	0	0	0	0	0	0	0	0	0	0	0	0	0

Glycocholate_Post-UK	0	0	0	0	0	0	0	0	0	0	0	0	0	0	0	0	0	0	0	0	0	0	0	0	0	0	0	0	0	0	0	0
dCTP_Post-UK	0	0	0	0	0	0	0	0	0	0	0	0	0	0	0	0	0	0	0	0	0	0	0	0	0	0	0	0	0	0	0	0
dUTP_Post-UK	0	0	0	0	0	0	0	0	0	0	0	0	0	0	0	0	0	0	0	0	0	0	0	0	0	0	0	0	0	0	0	0
dTTP_Post-UK	0	0	0	0	0	0	0	0	0	0	0	0	0	0	0	0	0	0	0	0	0	0	0	0	0	0	0	0	0	0	0	0
CTP_Post-UK	0	0	0	0	0	0	0	0	0	0	0	0	0	0	0	0	0	0	0	0	0	0	0	0	0	0	0	0	0	0	0	0
UTP_Post-UK	0	0	0	0	0	0	0	0	0	0	0	0	0	0	0	0	0	0	0	0	0	0	0	0	0	0	0	0	0	0	0	0
dATP_Post-UK	0	0	0	0	0	0	0	0	0	0	0	0	0	0	0	0	0	0	0	0	0	0	0	0	0	0	0	0	0	0	0	0
dITP_Post-UK	0	0	0	0	0	0	0	0	0	0	0	0	0	0	0	0	0	0	0	0	0	0	0	0	0	0	0	0	0	0	0	0
ATP_Post-UK	0	0	0	0	0	0	0	0	0	0	0	0	0	0	0	0	0	0	0	0	0	0	0	0	0	0	0	0	0	0	0	0
dGTP_Post-UK	0	0	0	0	0	0	0	0	0	0	0	0	0	0	0	0	0	0	0	0	0	0	0	0	0	0	0	0	0	0	0	0
ITP_Post-UK	0	0	0	0	0	0	0	0	0	0	0	0	0	0	0	0	0	0	0	0	0	0	0	0	0	0	0	0	0	0	0	0
Taurocholate_Post-UK	0	0	0	0	0	0	0	0	0	0	0	0	0	0	0	0	0	0	0	0	0	0	0	0	0	0	0	0	0	0	0	0
GTP_Post-UK	0	0	0	0	0	0	0	0	0	0	0	0	0	0	0	0	0	0	0	0	0	0	0	0	0	0	0	0	0	0	0	0
XTP_Post-UK	0	0	0	0	0	0	0	0	0	0	0	0	0	0	0	0	0	0	0	0	0	0	0	0	0	0	0	0	0	0	0	0
CDP-choline_Post-UK	0	0	0	0	0	0	0	0	0	0	0	0	0	0	0	0	0	0	0	0	0	0.119	0	0	0	0	0	0	0	0	0	0
ADP-ribose_Post-UK	0	0	0	0	0	0	0	0	0	0	0	0	0	0	0	0	0	0	0	0	0	0	0	0	0	0	0	0	0	0	0	0
TDP-glucose_Post-UK	0	0	0	0	0	0	0	0	0	0	0	0	0	0	0	0	0	0	0	0	0	0	0	0	0	0	0	0	0	0	0	0
UDP-glucose_Post-UK	0	0.198	0	0	0	0.0999	0	0.197	0	0	0	0	0	0	0	0	0	0	0	0	0.167	0.114	0	0	0.109	0	0	0.0482	0	0	0	0.0489
UDP-glucuronate_Post-UK	0	0	0	0	0	0	0	0	0	0	0	0	0	0	0	0	0	0	0	0	0	0	0	0	0	0	0	0.0514	0	0	0	0
ADP-glucose_Post-UK	0	0	0	0	0	0	0	0	0	0	0	0	0	0	0	0	0	0	0	0	0	0	0	0	0	0	0	0	0	0	0	0
GDP-mannose_Post-UK	0	0	0	0	0	0	0	0	0	0	0	0	0	0	0	0	0	0	0	0	0	0	0	0	0	0	0	0	0	0	0	0
UDP-*N*-acetylglucosamine_Post-UK	0	0.339	0	0	0	0	0	0.329	0	0	0	0	0	0	0	0	0	0	0	0	0	0	0	0	0.205	0	0	0	0	0	0	0
CMP-*N*-acetylneuraminate_Post-UK	0	0	0	0	0	0	0	0	0	0	0	0	0	0	0	0	0	0	0	0	0	0	0	0	0	0	0	0	0	0	0	0
NAD+_Post-UK	0	0	0	0	0	0	0	0	0	0	0	0	0	0	0	0	0	0	0	0	0	0	0	0	0	0	0	0	0	0	0	0
Deamido-NAD+_Post-UK	0	0	0	0	0	0	0	0	0	0	0	0	0	0	0	0	0	0	0	0	0	0	0	0	0	0	0	0	0	0	0	0
NADH_Post-UK	0	0	0	0	0	0	0	0	0	0	0	0	0	0	0	0	0	0	0	0	0	0	0	0	0	0	0	0	0	0	0	0
NADP+_Post-UK	0	0	0	0	0	0	0	0	0	0	0	0	0	0	0	0	0	0	0	0	0	0	0	0	0	0	0	0	0	0	0	0
FAD_Post-UK	0	0	0	0	0	0	0	0	0	0	0	0	0	0	0	0	0	0	0	0	0	0	0	0	0	0	0	0	0	0	0	0
Urea_Recover	235	310	353	281	313	251	387	202	281	598	237	920	287	440	1870	304	1750	550	913	507	654	270	701	630	217	193	432	549	402	1130	334	148
Pyrazole_Recover	0	0	0	0	0	0	0	0	0	0	0	0	0	0	0	0	0	0	0	0	0	0	0	0	0	0	0	0	0	0	0	0
3-Aminopropionitrile_Recover	0	0	0	0	0	0	0	0	0	0	0	0	0	0	0	0	0	0	0	0	0	0	0	0	0	0	0	0	0	0	0	0
Methylguanidine_Recover	0	0	0	0	0	0	0	0	0	0	0	0	0	0	0	0	0	0	0	0	0	0	0	0	0	0	0	0	0	0	0	0
Isobutylamine_Recover	0	0	0	0	0	0	0	0	0	0	0	0	0	0	0	0	0	0	0	0	0	0	0	0	0	0	0	0	0	0	0	0
1,3-Diaminopropane_Recover	0.196	0.0372	0.0964	0.0817	0	0.177	0.103	0.125	0.0904	0.459	0.158	0.132	0.0578	0.0688	0.0971	0.0991	0.117	0.372	0.459	0.122	0.221	0.101	0.0627	0.398	0.354	0.3	0	0.0604	0.184	0.112	0.0903	0.0638
Gly_Recover	5.62	8.58	14.3	7.89	11.5	11	10.1	8.66	13.4	29.9	5.29	11.5	8.37	6.38	70.6	8.38	8.81	9.11	7.79	9.99	11	24.9	10	14.1	12.8	26.5	5.78	5.45	8.49	35.4	5.17	3.9
Trimethylamine *N*-oxide_Recover	0	0.0516	0.248	0.0216	0.02	0.0393	0	0.0635	0.0621	0.0393	0.151	0.0433	0	0.0173	0.121	0.0153	0.749	0.0253	0.743	0.00837	0.0799	0.0397	0.0394	0	0.023	0.225	0	0.134	0.0285	0.053	0.0417	0
Isopropanolamine_Recover	0.0926	0.761	0.162	0.0886	0.0946	0.135	0.259	0.154	0.109	0.545	0.171	0.265	0.187	0.135	0.107	0.0691	0.102	0.139	0.11	0.0561	0.141	0.0933	0.129	0.124	0	0.0509	0.162	0.0668	0.0455	0.0982	0.289	0.0587
Hydroxyurea_Recover	0	0	0	0	0	0	0	0	0	0	0	0	0	0	0	0	0	0	0	0	0	0	0	0	0	0	0	0	0	0	0	0
Trimethylsulfonium_Recover	0	0	0	0	0	0	0	0	0	0	0	0	0	0	0	0	0	0	0	0	0	0	0	0	0	0	0	0	0	0	0	0
Cysteamine_Recover	0	0	0	0	0	0	0	0	0	0	0	0	0	0	0	0	0	0	0	0	0	0	0	0	0	0	0	0	0	0	0	0
Isoamylamine_Recover	0	0	0	0	0	0	0	0	0	0	0	0	0	0	0	0	0	0	0	0	0	0	0	0	0	0	0	0	0	0	0	0
Putrescine(1,4-Butanediamine)_Recover	8.03	17	10.5	6.18	7.76	11.5	17.8	8.39	11.2	37.1	2.18	13.4	5.15	6.1	15.3	11.8	1.12	6.81	6.68	4.77	13.9	10.4	8.63	11.2	6.34	9.14	3.54	1.39	11.3	13.5	7.97	1.3
β-Ala_Recover	0.239	0.513	0.364	0.269	0.446	0.225	0.71	0.31	0.166	0.788	0.836	0.317	0.926	0.402	1.36	0.219	0.66	0.407	0.531	0.288	0.27	0.247	0.23	0.376	0.487	0.475	0.426	0.231	0.554	0.214	0.18	0.201
Ala_Recover	2.92	6.14	4.34	6.03	4.89	6.88	5.55	6.43	4.33	13.4	5.29	7.04	4.99	6.06	16	3.79	5.76	6.46	5.52	4.39	4.62	6.38	3.94	6.74	6.75	9.68	6.07	2.99	5.66	4.38	4.42	2.68
Sarcosine_Recover	0.63	1.82	2.69	1.4	0.95	1.18	1.57	1.36	0.953	3.07	2.66	1.8	1.46	1.17	2.1	1.65	2.47	1.28	0.902	1.04	1.55	2.19	1.53	1.27	1.43	1.68	1.78	0.522	1.57	0.945	0.963	1.07
3-Aminopropane-1,2-diol_Recover	0	0	0	0.0154	0	0	0	0.023	0	0	0	0	0	0	0	0	0	0	0	0	0	0	0	0	0	0	0.0118	0	0	0	0.033	0
Aniline_Recover	0	0	0	0	0	0	0	0	0	0	0	0	0	0	0	0	0	0	0	0	0	0	0	0	0	0	0	0	0	0	0	0
4-Hydroxymethylimidazole_Recover	0	0	0	0	0	0	0	0	0	0	0	0	0	0	0	0	0	0	0	0	0	0	0	0	0	0	0	0	0	0	0	0
1-Methyl-2-pyrrolidinone_Recover	0	0	0	0	0	0	0	0	0	0	0	0	0	0	0	0	0	0	0	0	0	0	0	0	0	0	0	0	0	0	0	0
Cyclohexylamine_Recover	0	0	0	0	0	0	0	0	0	0	0	0	0	0	0	0	0	0	0	0	0	0	0	0	0	0	0	0	0	0	0	0
1-Aminocyclopropane-1-carboxylate_Recover	0	0	0	0	0	0	0	0	0	0	0	0	0	0	0	0	0	0	0	0	0	0	0	0	0	0	0	0	0	0	0	0
Betaine aldehyde_Recover	0	0	0	0	0	0	0	0	0	0	0	0	0	0	0	0	0	0	0	0	0	0	0	0	0	0	0	0	0	0	0	0
Spermine_Recover	0.019	0.11	0.0622	0.0312	0.202	0	0.0364	0.0502	0.0405	0.0577	0	0.121	0.0748	0.0375	0.199	0.0207	0.0209	0.0488	0.0346	0.0174	0.0866	0.0358	0	0.0516	0.0205	0	0.0456	0.0666	0.0721	0.0554	0.0832	0.0189
Hexylamine_Recover	0	0	0	0	0	0	0	0	0	0	0	0	0	0	0	0	0	0	0	0	0	0	0	0	0	0	0	0	0	0	0	0
Cadaverine_Recover	0.912	0.816	8.11	0.627	4.23	5.25	4	4.21	2.44	6.83	1.37	1.94	1.65	1.2	1.17	3.69	1.01	1.91	5.05	0.615	2.48	2.42	0.52	3.98	3.12	2.31	0.583	0.529	5.45	4.42	1.13	0.814
GABA_Recover	0.261	0.376	0.335	0.301	0.251	0.365	0.324	0.425	0.189	0.567	0.429	0.268	1.58	0.161	0.289	0.482	0.249	0.375	0.211	0.285	0.36	0.301	0.351	0.445	0.365	0.495	0.218	0.0831	0.333	0.688	0.294	0.159
3-Aminoisobutyrate_Recover	0	0.539	0.0895	0.113	0.229	0.257	0.196	0.255	0.0948	0.214	0.747	0.43	0.301	0.132	0.397	0.245	0.164	0.164	0.296	0.142	0.0817	0.147	0.383	0.253	0.459	0.573	0.109	0.175	0.201	0.133	0.283	0.0809
2AB_Recover	0.142	0.473	0.361	0.171	0.113	0.451	0.342	0.306	0.148	0.707	0.182	0.408	0.255	0.182	0.477	0.209	0.346	0.199	0.185	0.167	0.276	0.168	0.347	0.288	0.192	0.303	0.242	0.0928	0.298	0.0861	0.192	0.109
N-Methylalanine_Recover	0	0	0	0	0	0	0	0	0	0	0	0	0	0	0	0	0	0	0	0	0	0	0	0	0	0	0	0	0	0	0	0
N,N-Dimethylglycine_Recover	0.213	0.226	0.0777	0.0781	0.118	0.137	0.151	0.0442	0.0669	0.179	0.14	0.106	0.163	0.0898	0.177	0.0827	0.201	0.109	0.193	0.085	0.1	0.087	0.103	0.102	0	0.138	0.0604	0.0618	0	0.115	0.0678	0.0965
Choline_Recover	1.77	2.76	2.37	2.11	2.37	3.2	2.85	3.06	2.23	4.65	2.05	2.25	2.81	2.56	4.98	2.12	3.19	3.97	3.37	2.83	2.84	2.21	1.49	3.29	3.2	3.7	3.46	2.44	3.31	1.36	2.11	1.46
2,3-Diaminopropionate_Recover	0	0	0	0	0	0	0	0	0	0	0	0	0	0	0	0	0	0	0	0	0	0	0	0	0	0	0	0	0	0	0	0
Ser_Recover	1.26	3.6	2.22	1.11	3.52	3.66	2.93	2.55	2.11	5.57	1.71	3.17	1.91	2.39	7.9	1.62	4.31	2.97	2.93	1.8	2.38	3.05	2.54	3.37	2.76	4.7	2.3	1.88	2.49	2.56	1.67	1.46
Diethanolamine_Recover	5.33	1.15	4.27	1.68	0.82	1.47	0.606	1.32	1.23	0.858	2.32	0.934	1.94	1.9	1.27	0.721	1.54	2.27	1.87	1.17	1.36	1.12	0.682	2	1.17	3.39	1.92	2.46	0.885	0.949	1.12	0.789
N-Methylaniline_Recover	0	0	0	0	0	0	0	0	0	0	0	0	0	0	0	0	0	0	0	0	0	0	0	0	0	0	0	0	0	0	0	0
β-Ala-Lys_Recover	0	0	0	0	0	0	0.0186	0	0	0	0	0	0.0142	0	0	0.0158	0	0	0	0.00576	0	0	0	0	0.0223	0	0	0	0	0	0	0
Hypotaurine_Recover	0	0.509	0.271	0.345	0.362	0	0.454	0.893	0	0.267	0.333	0.241	0.322	0.381	1.07	0.195	0.29	0.222	0	0.27	0.247	0.222	0.187	0.369	0.357	0.458	0.276	0	0.336	0	0.351	0.269
2-Aminophenol_Recover	0	0	0	0	0	0	0	0	0	0	0	0	0	0	0	0	0	0	0	0	0	0	0	0	0	0	0	0	0	0	0	0
Cytosine_Recover	0	0.032	0.0967	0.0562	0.0531	0.0497	0.053	0.0401	0.0499	0.0886	0.106	0.0846	0.0494	0.0346	0	0.0565	0.0796	0.0583	0.0828	0	0.0459	0.0604	0.0282	0	0.042	0.0532	0.0594	0.0469	0.0626	0	0.0447	0.0319
Histamine_Recover	0.302	0.11	0.83	0.766	0.0763	0.0491	0.0899	0.156	0.0379	0.0319	0.0484	0.044	0.0329	0.0372	0.0339	0.0185	0.0104	0.446	0.174	0.129	0.0425	0.171	0.052	0.0393	0.0204	0.0365	0.0221	0	0.757	0.0424	0.0344	0.0224
Uracil_Recover	0.608	0.616	1.06	0	0	0.593	0.305	0.804	0.328	0	1.12	0.699	0.79	0.708	2.34	0.75	1.12	1.13	0	0.967	0	0	0.567	1.42	0	0	1.88	1.14	0.775	0	0.949	0
Carnosine_Recover	0	0	0	0	0.111	0	0.0619	0.0219	0.00985	0.0449	0.0394	0	0.185	0.0176	0.251	0.014	0.0256	0.0298	0.0264	0	0.0193	0.0277	0	0.0175	0.0162	0	0	0.118	0	0.0155	0	0.0301
Creatinine_Recover	0.701	1.89	0.969	0.739	0.697	0.53	0.689	0.811	0.759	2.05	1.12	0.873	1.1	1.09	3.23	0.548	1.71	0.894	1.98	0.619	1.23	0.709	1.26	1.23	1.44	1.37	0.74	0.675	1.31	1.03	0.67	0.515
Muscimol_Recover	0	0	0	0	0	0	0	0	0	0	0	0	0	0	0	0	0	0	0	0	0	0	0	0	0	0	0	0	0	0	0	0
β-Cyanoalanine_Recover	0	0	0	0	0	0	0	0	0	0	0	0	0	0	0	0	0	0	0	0	0	0	0	0	0	0	0	0	0	0	0	0
Dihydrouracil_Recover	0	0	0	0	0	0	0	0	0	0	0	0	0	0	0	0	0	0	0	0	0	0	0	0	0	0	0	0	0	0	0	0
Pro_Recover	5.43	3.41	14.7	6.69	8.23	11.4	9.64	6.64	5.95	53	3.12	18.3	5.77	4.47	27.7	7.19	5.11	4.49	5.9	11.8	6.58	18.2	3.88	10.4	4.05	9.58	2.33	3.32	6.9	52.6	3.46	3.43
Guanidinoacetate_Recover	0.293	0.798	0.359	0.461	0.253	0	0	0	0.504	0	0.373	0	0	0	0.508	0	0.154	0.65	0	0	0	0.292	0	0.389	0.448	0.429	0.173	0.224	0.483	0.776	0.495	0.106
5-Aminovalerate_Recover	39.2	112	80.8	50	21.4	99.3	99.1	60.7	74.8	344	29.3	108	61.3	63	96	73.7	18.9	44.1	86.2	62.8	112	60.7	65	107	50.5	80.5	16.6	4.86	78.4	61.3	43.7	5.55
Val_Recover	1.28	2.01	4.25	1.05	1.88	3.97	2.52	2.38	1.26	7.44	1.42	2.19	2.13	1.2	5	1.59	2.4	1.76	1.46	1.01	2.36	1.43	7.32	2.08	1.49	1.76	1.81	0.873	2.4	0.483	2.39	0.905
Betaine_Recover	0.817	1.84	1.92	0.988	0.668	0.685	0.67	0.837	0.989	2.08	1.04	1.38	0.644	0.454	1.26	0.457	0.965	0.462	2.15	0.81	0.877	0.637	1.35	0.731	0.706	1.5	0.445	0.833	0.662	1.87	0.723	0.535
Benzimidazole_Recover	0	0	0	0	0	0	0	0	0	0	0	0	0	0	0	0	0	0	0	0	0	0	0	0	0	0	0	0	0	0	0	0
2,4-Diaminobutyrate_Recover	0	0	0	0	0	0	0	0	0	0	0	0	0	0	0	0	0	0	0	0	0	0	0	0	0	0	0	0	0	0	0	0
4-Amino-3-hydroxybutyrate_Recover	0	0	0	0	0	0	0	0	0	0	0	0	0	0	0	0	0	0	0	0	0	0	0	0	0	0	0	0	0	0	0	0
Homoserine_Recover	0	0	0	0.0537	0.0714	0	0.0769	0.0611	0	0.131	0.0689	0.0965	0.105	0.0578	0.0671	0.043	0.0649	0.0457	0.0467	0.0597	0.0964	0.0669	0	0.0649	0.0392	0.0176	0	0	0.0922	0.0839	0.0384	0.0305
α-Methylserine_Recover	0	0	0	0	0	0	0	0	0	0	0	0	0	0	0	0	0	0	0	0	0	0	0	0	0	0	0	0	0	0	0	0
Thr_Recover	0.466	1.64	1.39	0.534	1.37	1.87	1.4	1.18	0.786	1.63	0.844	0.98	0.997	0.829	4.09	0.588	1.59	1.1	0.962	0.571	0.927	1.1	0.617	1.03	0.894	1.23	1.21	0.682	0.921	0.566	1.02	0.782
Purine_Recover	0	0	0	0	0	0	0	0	0	0	0	0	0	0	0	0	0	0	0	0	0	0	0	0	0	0	0	0	0	0	0	0
Anserine_Recover	0.0465	0	0.0426	0	0.0635	0.0142	0.201	0	0	0.0334	0	0.0307	0.765	0	0.0875	0.0116	0.086	0.0227	0.0701	0.0991	0	0	0	0	0	0	0	0.167	0.102	0.0155	0	0
Homocarnosine_Recover	0	0	0	0	0.0362	0	0	0	0	0	0	0.0138	0.053	0	0	0	0.0126	0	0	0	0	0	0	0.0123	0	0	0	0.0251	0	0	0	0
Benzamidine_Recover	0	0	0	0	0	0	0	0	0	0	0	0	0	0	0	0	0	0	0	0	0	0	0	0	0	0	0	0	0	0	0	0
Cys_Recover	0	0	0	0	0	0	0	0	0	0	0	0	0	0	0	0	0	0	0	0	0	0	0	0	0	0	0	0	0	0	0	0

Benzamide_Recover	2.34	9.79	4.35	2.39	2.55	4.68	5.34	2.17	4.21	8.87	3.28	6.26	1.84	4.18	4.08	2.1	2.58	5.51	3.81	2.23	4.75	3.94	4.23	5.19	2.43	3.59	1.62	2	1.44	3.17	2.45	3.22
Phenethylamine_Recover	0	0	0	0	0	0	0	0	0	0	0	0	0	0	0	0	0	0	0	0	0	0	0	0	0	0	0	0	0	0	0	0
α-Methylbenzylamine_Recover	0	0	0	0	0	0	0	0	0	0	0	0	0	0	0	0	0	0	0	0	0	0	0	0	0	0	0	0	0	0	0	0
2,4-Dimethylaniline_Recover	0	0	0	0	0	0	0	0	0	0	0	0	0	0	0	0	0	0	0	0	0	0	0	0	0	0	0	0	0	0	0	0
Isonicotinamide_Recover	0	0	0	0	0	0	0	0	0	0	0	0	0	0	0	0	0	0	0	0	0	0	0	0	0	0	0	0	0	0	0	0
Nicotinamide_Recover	0.0824	0.128	0.115	0.0799	0.124	0.0986	0.0939	0.0774	0.128	0.293	0.09	0.0961	0.0729	0.065	0.152	0.0789	0.0253	0.0674	0.153	0.0367	0.141	0.0805	0.0714	0.109	0.099	0.0975	0.0225	0.0749	0.133	0.0724	0.0644	0.0867
Picolinamide_Recover	0	0	0	0	0	0	0	0	0	0	0	0	0	0	0	0	0	0	0	0	0	0	0	0	0	0	0	0	0	0	0	0
3-Chloroalanine_Recover	0	0	0	0	0	0	0	0	0	0	0	0	0	0	0	0	0	0	0	0	0	0	0	0	0	0	0	0	0	0	0	0
Taurine_Recover	27	62.8	40.5	35.3	50.4	24.6	43.4	62.4	36.9	40.5	46.8	23.6	58.3	32.9	47	25.7	41.4	37.4	38.6	22.7	38.2	34.5	30	41.8	53.8	45.7	28.3	27	37.6	22.1	35.1	24.2
5-Methylcytosine_Recover	0	0	0	0	0	0	0	0	0	0	0	0	0	0	0	0	0	0	0	0	0	0	0	0	0	0	0	0	0	0	0	0
1-Methylhistamine_Recover	0	0	0.0192	0.00965	0	0	0	0	0	0	0	0	0	0	0	0	0	0.0177	0	0.0115	0	0.0104	0	0	0	0	0	0	0.0195	0	0	0
Thymine_Recover	1.92	7.26	1.78	1.88	3.52	1.56	1.87	5.24	2.64	2.06	3	1.86	3.01	2.95	2.64	1.1	5.35	1.86	1.54	2.7	2.79	2.14	1.49	1.8	3.43	2.45	1.83	2.44	2.9	2.99	2.59	1.54
Imidazole-4-acetate_Recover	0	0	0	0	0	0	0	0	0	0	0	0	0	0	0	0	0	0	0	0	0	0	0	0	0	0	0	0	0	0	0	0
Melamine_Recover	0	0	0	0	0	0	0	0	0	0	0	0	0	0	0	0	0	0	0	0	0	0	0	0	0	0	0	0	0	0	0	0
1-Amino-1-cyclopentanecarboxylate_Recover	0	0	0	0	0	0	0	0	0	0	0	0	0	0	0	0	0	0	0	0	0	0	0	0	0	0	0	0	0	0	0	0
Pipecolate_Recover	0.056	0.079	0.178	0.185	0.0535	0.0815	0.0803	0.0731	0.102	0.227	0.261	0.0524	0.0591	0.0468	0.122	0.055	0.238	0.0609	0.228	0.0417	0.0706	0.0634	0.0528	0.1	0.17	0.169	0.0505	0.0369	0.0917	0.056	0.0916	0.0395
Metformin_Recover	0	0	0	0	0	0	0	0	0	0	0	0	0	0	0	0	0	0	0	0	0	0	0	0	0	0	0	0	0	0	0	0
Octylamine_Recover	0	0	0	0	0	0	0	0	0	0	0	0	0	0	0	0	0	0	0	0	0	0	0	0	0	0	0	0	0	0	0	0
N-Acetylputrescine_Recover	0.25	0.801	0.379	0.238	0.345	0.252	0.623	0.37	0.3	1.74	0.355	0.46	0.34	0.312	0.32	0.319	0.241	0.276	0.487	0.295	0.58	0.391	0.55	0.325	0.319	0.747	0.128	0.0649	0.433	0.583	0.337	0.065
Agmatine_Recover	0.0338	0.0989	0.0371	0.0419	0.0615	0.0379	0.0496	0.0318	0.0415	0.0754	0.0438	0	0.0232	0.0397	0	0.0412	0	0.0434	0.0606	0.0239	0.0385	0.0275	0	0.0414	0.0272	0.0358	0.0269	0	0.026	0.0602	0	0
Hydroxyproline_Recover	0	0.19	0.259	0.188	0.208	0.197	0.211	0.143	0.118	0.239	0.348	0.175	0.188	0.166	1.26	0.168	0.471	0.256	0.131	0.147	0.134	0.185	0	0.172	0.129	0.196	0.193	0.126	0.116	0.118	0.109	0.151
5-Aminolevulinate_Recover	0	0.0579	0	0	0.0383	0.0328	0	0	0	0.0433	0.0281	0	0.0393	0	0.0385	0	0	0	0	0	0.0307	0.0251	0	0	0	0	0	0	0	0	0	0
Creatine_Recover	2.6	6.76	4.87	4.43	3.1	3.9	3.17	4.77	3.27	9.34	4.55	5.48	3.73	4.3	15.7	1.7	6.5	3.85	3.95	3.14	3.78	3.63	2.47	3.24	5.51	6.21	4.9	4.15	4.12	3.17	3.37	3.28
6-Aminohexanoate_Recover	0	0.261	0	0	0	0	0	0	0	0	0	0.252	0	0	0	0	0	0	0	0	0	0	0	0	0	0	0	0	0	0	0	0
β-Leucine_Recover	0	0	0	0	0	0	0	0	0	0	0	0	0	0	0	0	0	0	0	0	0	0	0	0	0	0	0	0	0	0	0	0
Ile_Recover	0.256	0.539	1.03	0.178	0.634	1	0.634	0.796	0.307	1.67	0.257	0.363	0.759	0.284	1.53	0.37	1.05	0.454	0.439	0.266	0.554	0.395	2.38	0.589	0.329	0.429	0.469	0.317	0.614	0.148	0.712	0.302
Leu_Recover	0.82	2.07	3.69	0.623	2.05	2.73	2.45	2.21	1.62	5	0.681	2.22	1.75	0.868	5	1.19	2.55	1.36	1.51	0.869	1.78	1.08	5.41	1.86	1.01	1.39	1.28	1.2	1.89	0.807	1.89	0.73
Bis(3-aminopropyl)amine_Recover	0	0	0	0	0	0	0	0	0	0	0	0	0	0	0	0	0	0	0	0	0	0	0	0	0	0	0	0	0	0	0	0
Gly-Gly_Recover	0.0774	0	0.131	0.0669	0.04	0.101	0.169	0.0431	0.0578	0.295	0.0766	0.0892	0.045	0	0.126	0.0931	0.053	0.1	0.133	0.0398	0.135	0	0	0.0976	0.0959	0.153	0.0407	0	0.0904	0.106	0	0
Asn_Recover	0.163	0.456	0.224	0.203	0.746	0	0.0615	0.155	0.106	0	0	0.211	0.272	0	2.74	0.0273	0.253	0	0	0.144	0	0.281	0	0	0.0437	0	0.0641	0.543	0.0578	0.196	0.209	0.215
Ornithine_Recover	1.03	1.63	2.51	1.18	2.69	1.55	3.33	0.893	2	11.7	0.811	3.92	0.831	1.56	2.85	2.18	0.984	2.1	1.56	1.23	1.81	3.07	1.8	2.99	1.74	4.49	0.518	0.653	3.55	5.3	0.703	0.389
Asp_Recover	1.14	2.14	1.82	1.3	2.03	2.35	2.61	1.54	2.29	6.3	2.49	3.23	1.57	2.55	14.1	2.76	2.51	3.38	3.11	2.42	3.13	2.19	2.25	3.99	2.99	4.89	2.33	1.51	3.46	3.22	1.56	0.9
2-Aminobenzimidazole_Recover	0	0	0	0	0	0	0	0	0	0	0	0	0	0	0	0	0	0	0	0	0	0	0	0	0	0	0	0	0	0	0	0
Homocysteine_Recover	0	0	0	0	0	0	0	0	0	0	0	0	0	0	0	0	0	0	0	0	0	0	0	0	0	0	0	0	0	0	0	0
Adenine_Recover	0.106	0.234	0.397	0.114	0.223	0.264	0.202	0.274	0.183	0.293	0.4	0.139	0.12	0.226	0.199	0.186	0.294	0.279	0.261	0.128	0.227	0.135	0.201	0.405	0.34	0.305	0.183	0.153	0.227	0.0864	0.19	0.095
Hypoxanthine_Recover	0.785	1.93	1.54	0.734	0.928	1.55	0.897	2.87	0.91	1.99	1.12	1.1	1.54	2.32	3.08	0.891	2.57	2.31	1.97	1.63	1.74	1.52	0.872	2.44	2.26	2.42	1.84	1.6	2.01	0.313	1.83	1.03
1-Methylnicotinamide_Recover	0	0.0652	0.0284	0	0	0.0131	0	0	0.0233	0.0472	0.0119	0.0354	0.0271	0.0168	0.0683	0	0.106	0	0.049	0	0	0	0.0255	0.0223	0	0	0.017	0	0	0	0	0
p-Aminobenzoate_Recover	0	0	0	0	0	0	0	0	0	0	0	0	0	0	0	0	0	0	0	0	0	0	0	0	0	0	0	0	0	0	0	0
Trigonelline_Recover	0.801	0.092	0.0547	0	0.184	0	0.0388	0.0568	0	0	0	0.127	0.0641	0	0.522	0	0	0	0.0342	0	0	0.254	0	0	0	0.4	0	0	0	0	0	0
Anthranilate_Recover	0	0	0	0	0	0	0	0	0	0	0	0	0	0	0	0	0	0	0	0	0	0	0	0	0	0	0	0	0	0	0	0
Phenylethanolamine_Recover	0	0	0	0	0	0	0	0	0	0	0	0	0	0	0	0	0	0	0	0	0	0	0	0	0	0	0	0	0	0	0	0
Tyramine_Recover	0	0.0632	0	0	0.0252	0	0	0	0	0	0.0434	0	0.0151	0	0	0	0	0.0628	0	0	0	0	0	0	0	0	0	0	0.0886	0	0.00723	0
Urocanate_Recover	0.179	0.476	0.216	0.136	0.802	0.549	0.285	0.119	0.138	0.364	0.259	0.347	0.213	0.24	0.699	0.149	0.281	0.436	0.567	0.158	0.313	0.446	0.248	0.37	0.27	0.613	0.115	0.0536	0.131	0.375	0.1	0.144
6-Hydroxynicotinate_Recover	0	0	0	0	0	0	0	0	0	0	0	0	0	0	0	0	0	0	0	0	0	0	0	0	0	0	0	0	0	0	0	0
Tropinone_Recover	0	0	0	0	0	0	0	0	0	0	0	0	0	0	0	0	0	0	0	0	0	0	0	0	0	0	0	0	0	0	0	0
Histidinol_Recover	0	0	0	0	0	0	0	0	0	0.0132	0	0	0	0	0	0	0	0	0	0	0	0	0	0	0.0236	0	0	0	0	0	0	0
Tropine_Recover	0	0	0	0	0	0	0	0	0	0	0	0	0	0	0	0	0	0	0	0	0	0	0	0	0	0	0	0	0	0	0	0
Ectoine_Recover	0	0	0	0	0	0	0	0	0	0	0	0	0	0	0	0	0	0	0	0	0	0	0	0	0	0	0	0	0	0	0	0
4-Methyl-5-thiazoleethanol_Recover	0	0	0	0	0	0	0	0	0	0	0	0	0	0	0	0	0	0	0	0	0	0	0	0	0	0	0	0	0	0	0	0
Proline betaine_Recover	0.0903	0	0.14	0.0343	0.0226	0.0808	0.0225	0	0.0336	0.0881	0.0273	0	0	0	0.342	0.0434	0.0237	0.0141	0.068	0.0128	0	0.0228	0.0412	0	0.0669	0	0.0193	0.0166	0	0.0216	0.0273	0
N^1,N^{12}-Diacetylspermine_Recover	0	0.0261	0.0131	0	0.0209	0.0155	0.0251	0.00904	0.0269	0.0387	0.0214	0.0217	0.0196	0.0232	0	0.0201	0	0.0286	0.014	0	0.0326	0.0212	0.014	0.0148	0.0117	0	0.016	0	0	0.0148	0	0
γ-Guanidinobutyrate_Recover	0	0	0	0	0	0	0	0	0	0	0	0	0	0.0137	0	0	0	0	0	0	0	0.0286	0.0248	0	0	0	0	0	0	0	0	0
Acetylcholine_Recover	0	0	0	0	0	0	0	0	0	0	0	0	0	0	0	0	0	0	0	0	0	0	0	0	0	0	0	0	0	0	0	0
γ-Butyrobetaine_Recover	0.253	1.49	0.597	0.192	0.176	0.759	0.572	0.446	0.646	1.55	0.635	0.602	0.348	0.37	0.496	0.395	0.507	0.668	0.774	0.323	0.544	0.4	0.526	0.3	0.685	0.827	0.305	0.0643	0.44	0.18	0.309	0.0776
Lysinamide_Recover	0	0	0	0	0	0.0295	0	0	0	0	0	0	0	0	0	0	0	0	0	0	0	0	0	0	0	0	0	0	0	0	0	0
Spermidine_Recover	0.0995	0.44	0.254	0.138	0.243	0.315	0.571	0.175	0.416	0.924	0.254	0.359	0.0632	0.162	0.422	0.191	0.122	0.567	0.361	0.0995	0.487	0.195	0.163	0.401	0.243	0.301	0.236	0.205	0.314	0.211	0.133	0.063
Gln_Recover	1.92	6.02	5.03	2.39	4.85	5.26	3.4	4.87	2.74	8.2	3.42	3.27	3.78	3.53	14.1	2.53	6.08	4.14	3.62	2.1	3.04	4.93	2.37	4.19	5.45	6.7	3.86	3.3	3.5	2.23	3.26	2.94
5,6-Dimethylbenzimidazole_Recover	0	0	0	0	0	0	0	0	0	0	0	0	0	0	0	0	0	0	0	0	0	0	0	0	0	0	0	0	0	0	0	0
Lys_Recover	1.73	2.83	2.24	1.13	5.72	1.52	5	1.89	4.51	10.1	0.944	2.53	1.89	2.37	6.19	2.99	3.18	3.19	3.6	1.97	3.61	6.98	2.36	2.84	3.65	5.64	1.97	1.68	4.47	8.9	1.35	1.13
Trientine_Recover	0	0	0	0	0	0	0	0	0	0	0	0	0	0	0	0	0	0	0	0	0	0	0	0	0	0	0	0	0	0	0	0
Glu_Recover	3.23	7.35	10.5	4.88	5.87	6.86	9.06	5.84	4.57	13.4	5.87	8.44	4.91	6.71	34.2	4.92	7.98	6.81	5.82	4.52	7.45	6.72	4.8	6.71	5.65	11.1	7.18	3.69	6.25	8.16	5	2.49
O-Acetylserine_Recover	0	0	0	0	0.122	0	0.074	0.0393	0	0.18	0	0	0	0	0.059	0	0.029	0	0	0	0.0769	0.0582	0	0.0588	0	0	0	0	0	0.115	0	0
Nornicotine_Recover	0	0	0	0	0	0	0	0	0	0	0	0	0	0	0	0	0	0	0	0	0	0	0	0	0	0	0	0	0	0	0	0
Met_Recover	0	0.341	0.0898	0	0.116	0.08	0	0.497	0	0.112	0.144	0.0353	0.442	0.0809	0.285	0.145	0.465	0.0896	0.171	0.144	0.096	0.0616	0.0966	0.112	0	0.163	0.318	0.179	0.321	0	0.362	0.148
3-Methyladenine_Recover	0	0	0	0	0	0	0	0	0	0	0	0	0	0	0	0	0	0	0	0	0	0	0	0	0	0	0	0	0	0	0	0
6-Methylaminopurine_Recover	0	0	0	0	0	0	0	0	0	0	0	0	0	0	0	0	0	0	0	0	0	0	0	0	0	0	0	0	0	0	0	0
Guanine_Recover	0.328	0.726	0.726	0.284	0.419	0.571	0.257	0.833	0.258	0.353	0.485	0.307	0.535	0.792	1.1	0.283	0.639	0.671	0.202	0.486	0.33	0.936	0.269	0.616	0.759	0.29	0.735	0.735	0.454	0.211	0.569	0.442
Amantadine_Recover	0	0	0	0	0	0	0	0	0	0	0	0	0	0	0	0	0	0	0	0	0	0	0	0	0	0	0	0	0	0	0	0
Xanthine_Recover	0	0.996	0.656	0.821	0.809	2.35	1.36	1.31	0.736	1.19	1.41	0.692	0.939	0.887	5.75	1.09	2.38	2.46	0.629	1.41	0.972	0.347	1.01	1.93	1.19	2.09	1.8	1.72	0	0.85	1.1	0
4-Aminosalicylate_Recover	0	0	0	0	0	0	0	0	0	0	0	0	0	0	0	0	0	0	0	0	0	0	0	0	0	0	0	0	0	0	0	0
3-Hydroxyanthranilate_Recover	0	0	0	0	0	0	0	0	0	0	0	0	0	0	0	0	0	0	0	0	0	0	0	0	0	0	0	0	0	0	0	0
Dopamine_Recover	0	0	0	0	0	0	0	0	0	0	0	0	0	0	0	0	0	0	0	0	0	0	0	0	0	0	0	0	0	0	0	0
4-(*β*-Acetylaminoethyl)imidazole_Recover	0.015	0.0247	0.0385	0.0248	0	0	0.0105	0	0	0	0.0073	0	0	0	0	0	0.00884	0.0224	0.0151	0.00733	0	0	0	0	0	0	0	0	0.0351	0.00847	0	0
Pseudopelletierine_Recover	0	0	0	0	0	0	0	0	0	0	0	0	0	0	0	0	0	0	0	0	0	0	0	0	0	0	0	0	0	0	0	0
His_Recover	1.73	2.29	1.53	2.04	3.57	1.35	4.15	1.49	2.2	3.3	1.11	2.72	1.14	2.39	3.38	2.26	2.92	1.91	2.49	1.19	2.38	2.34	1.79	2.04	2.61	3.39	1.49	1.21	1.98	2.66	0.779	1.05
β-Imidazolelactate_Recover	0	0	0.114	0	0	0	0	0	0	0.0352	0	0.124	0	0	0	0	0	0.166	0	0	0	0.0967	0.0866	0	0	0.0811	0	0	0	0.089	0	0
Ibotenate_Recover	0	0	0	0	0	0	0	0	0	0	0	0	0	0	0	0	0	0	0	0	0	0	0	0	0	0	0	0	0	0	0	0
Allantoin_Recover	22.4	15.3	13.7	19.9	17	12.7	20.1	9.31	19.8	15.4	22.6	16.4	10.1	10.8	16.1	11.4	12.2	17.7	15.4	19.6	13.1	15.2	12.5	17.7	18.7	11.2	9.27	18.6	19.5	20.9	10.3	13.2
Indole-3-acetaldehyde_Recover	0	10.3	0	0	0	0	0	0	0	0	18.8	4.08	0	0	0	9.25	0	4.43	15.2	0	0	14.9	0	0	18.3	0	0	19.5	14	0	0	0
Betonicine_Recover	0	0	0	0	0	0	0	0	0	0	0	0	0	0	0	0	0	0	0	0	0	0	0	0	0	0	0	0	0	0	0	0
N-Acetylvaline_Recover	0	0	0	0	0	0	0	0	0	0	0	0	0	0	0	0	0	0	0	0	0	0	0	0	0	0	0	0	0	0	0	0
Ala-Ala_Recover	0	0	0.138	0.0912	0.119	0	0.167	0	0.0915	0.291	0.16	0.15	0	0	0	0	0.106	0.115	0	0.0662	0.154	0	0.106	0.122	0.131	0.163	0.0832	0	0.194	0.134	0	0
Tryptamine_Recover	0	0	0	0	0	0	0	0	0	0	0	0	0	0	0	0	0	0	0	0	0	0	0	0	0	0	0	0	0	0	0	0
α-Aminoadipate_Recover	0.157	0.506	0.321	0.249	0.348	0.365	0.442	0.442	0.145	0.656	0.658	0.427	0.266	0.341	0.886	0.25	0.479	0.333	0.304	0.393	0.409	0.413	0.16	0.219	0.422	0.336	0.358	0.368	0.279	0.469	0.263	0.404
N-Methylglutamate_Recover	0	0	0	0	0	0	0	0	0	0	0	0	0	0	0	0	0	0	0	0	0	0	0	0	0	0	0	0	0	0	0	0
Indole-3-ethanol_Recover	0	0	0	0	0	0	0	0	0	0	0	0	0	0	0	0	0	0	0	0	0	0	0	0	0	0	0	0	0	0	0	0
Carnitine_Recover	0.284	0.512	0.376	0.308	0.354	0.53	0.267	0.485	0.34	0.654	0.412	0.57	0.276	0.421	1.05	0.252	0.613	0.557	0.593	0.335	0.335	0.433	0.32	0.419	0.583	0.743	0.247	0.41	0.312	0.407	0.216	0.134
2-Deoxystreptamine_Recover	0	0	0	0	0	0	0	0	0	0	0	0	0	0	0	0	0	0	0	0	0	0	0	0	0	0	0	0	0	0	0	0
5-Hydroxylysine_Recover	0	0	0	0	0	0	0	0	0	0	0	0	0	0	0	0	0	0	0	0	0	0	0	0	0	0	0	0	0	0	0	0
Nicotine_Recover	0	0	0	0	0	0	0	0	0	0	0	0	0	0	0	0	0	0	0	0	0	0	0	0	0	0	0	0	0	0	0	0
Pterin_Recover	0	0	0	0	0	0	0	0	0	0	0	0	0	0	0	0	0	0	0	0	0	0	0	0	0	0	0	0	0	0	0	0
S-Methylmethionine_Recover	0	0	0	0	0	0	0	0	0	0	0	0	0	0	0	0	0	0	0	0	0	0	0	0	0	0	0	0	0	0	0	0

Methionine sulfoxide_Recover	0.0632	0.0713	0.0849	0	0	0.0905	0	0.0868	0.0651	0	0	0	0	0.0556	0	0	0	0	0.0883	0.0412	0	0.0402	0	0.0718	0.0594	0.0693	0	0.045	0	0	0.0687	0
7-Methylguanine_Recover	0.0118	0.0481	0.0286	0	0.0303	0.0273	0.0279	0.0224	0.028	0.0437	0.0416	0.0283	0.0268	0.0254	0.0437	0	0.0404	0.0314	0	0	0.0249	0.0214	0.0232	0	0	0	0	0	0.0329	0.0243	0.0211	0
3-Methylguanine_Recover	0	0	0	0	0	0	0	0	0	0	0	0	0	0	0	0	0	0	0	0	0	0	0	0	0	0	0	0	0	0	0	0
Phe_Recover	2.42	2.14	3.5	2.18	5.53	2.75	3.64	2.29	2.37	5.56	1.76	3.74	1.93	2.1	7.37	2.5	2.49	2.06	2.01	1.27	3.71	2.72	3.55	3.19	1.4	3.3	1.28	1.26	3.2	1.86	2.66	1.29
Taurocyamine_Recover	0	0	0	0	0	0	0	0	0	0	0	0	0	0	0	0	0	0	0	0	0	0	0	0	0	0	0	0	0	0	0	0
Pyridoxal_Recover	0	0	0	0	0	0	0	0	0	0	0	0	0	0	0	0	0	0	0	0	0	0	0	0	0	0	0	0	0	0	0	0
3-Methoxytyramine_Recover	0	0	0	0	0	0	0	0	0	0	0	0	0	0	0	0	0	0	0	0	0	0	0	0	0	0	0	0	0	0	0	0
Synephrine_Recover	0	0	0	0	0	0	0	0	0	0	0	0	0	0	0	0	0	0	0	0	0	0	0	0	0	0	0	0	0	0	0	0
Pyridoxamine_Recover	0	0	0	0	0	0	0	0	0	0	0	0	0	0	0	0	0	0	0	0	0	0	0	0	0	0	0	0	0	0	0	0
Pyridoxine_Recover	0	0	0	0	0	0	0	0	0	0	0	0	0	0	0	0	0	0	0	0	0	0	0	0	0	0	0	0	0	0	0	0
Noradrenaline_Recover	0	0	0	0	0	0	0	0	0	0	0	0	0	0	0	0	0	0	0	0	0	0	0	0	0	0	0	0	0	0	0	0
3-Methylhistidine_Recover	0.0401	0.0623	0.103	0.0225	0.0553	0.0479	0.153	0.0415	0.0383	0.0784	0.0498	0.0933	0.15	0.043	0.353	0.0371	0.21	0.0705	0.172	0.0787	0.035	0.0439	0.0194	0.0535	0.06	0.0408	0.0389	0.039	0.101	0.0443	0.024	0.027
Indole-3-acetamide_Recover	0	1.1	0.556	0	0	0	0	0	0	1.07	0	0	0	0	1.64	1.04	0	0	1.28	0	0	1.29	0	0	0	0	0	0	0	0	0	1.19
N-Acetylornithine_Recover	0	0	0	0	0	0.0525	0.0384	0	0	0.087	0.0419	0	0.0245	0.0304	0.043	0	0	0	0.0518	0	0	0	0	0	0	0.0308	0	0	0	0.0207	0.0285	0
N-γ-Ethylglutamine_Recover	0	0.0618	0	0.0478	0.0938	0	0.0562	0.036	0	0	0.0532	0	0.0661	0	0	0.0622	0.0591	0	0	0.041	0	0	0	0	0	0	0.0449	0.0222	0.0842	0	0.0441	0
Arg_Recover	2	4.34	2.12	1.98	3.12	2.41	4.52	2.16	6.09	23.1	2.03	10.3	2.15	4.07	6.85	3.12	5.89	4.45	5.77	2.68	5.99	2.95	4.85	6.68	4.48	8.91	3.4	2.94	6.07	7.14	3.07	1.86
Gramine_Recover	0	0	0	0	0	0	0	0	0	0	0	0	0	0	0	0	0	0	0	0	0	0	0	0	0	0	0	0	0	0	0	0
N-ω-Methyltryptamine_Recover	0	0	0	0	0	0	0	0	0	0	0	0	0	0	0	0	0	0	0	0	0	0	0	0	0	0	0	0	0	0	0	0
Guanidinosuccinate_Recover	0	0	0	0	0	0	0	0	0	0	0	0	0	0	0	0	0	0	0	0	0	0	0	0	0	0	0	0	0	0	0	0
Indole-3-acetate_Recover	0	1.86	0.287	0	0	0.492	2.8	1.26	2.07	1.11	1.15	0.644	0.758	1.1	0	0.778	0.726	0.996	0.949	0.645	0.697	0.778	1.13	0.748	0.769	1.62	0.838	0	0.804	0	1.65	0
2-Guanidinobenzimidazole_Recover	0	0	0	0	0	0	0	0	0	0	0	0	0	0	0	0	0	0	0	0	0	0	0	0	0	0	0	0	0	0	0	0
Citrulline_Recover	0.482	1.32	0.696	0.604	1.05	0.931	1.83	1.29	1.16	3.18	0.714	1.12	0.763	1.09	3.89	0.766	1.78	1.77	1.23	0.824	1.59	1.18	1.33	1.93	1.51	4.2	0.45	0.437	1.51	1.53	0.981	0.446
Canavanine_Recover	0	0	0	0	0	0	0	0	0	0	0	0	0	0	0	0	0	0	0	0	0	0	0	0	0	0	0	0	0	0	0	0
Serotonin_Recover	0	0	0	0	0	0	0	0	0	0	0	0	0	0	0	0	0	0	0	0	0	0	0	0	0	0	0	0	0	0	0	0
Alliin_Recover	0	0	0	0	0	0	0	0	0	0	0	0	0	0	0.306	0	0	0	0	0	0	0	0	0	0	0	0	0	0	0	0	0
Cys-Gly_Recover	0	0	0	0	0	0	0	0	0	0	0	0	0	0	0	0	0	0	0	0	0	0	0	0	0	0	0	0	0	0	0	0
Xanthopterin_Recover	0	0	0	0	0	0	0	0	0	0	0	0	0	0	0	0	0	0	0	0	0	0	0	0	0	0	0	0	0	0	0	0
Mannosamine_Recover	0	0	0	0	0	0	0	0	0.00901	0.297	0	0	0	0	0	0	0.0769	0.0319	0	0	0	0	0	0	0	0	0	0	0	0	0.0289	0
Glucosamine_Recover	0	0.0327	0.0518	0	0	0.0422	0	0.122	0	0.263	0	0	0	0	0	0	0.162	0.0872	0.127	0	0.114	0	0	0.0916	0.0729	0	0.053	0	0	0	0	0
n-Butyl a-Picolinate_Recover	0	0	0	0	0	0	0	0	0	0	0	0	0	0	0	0	0	0	0	0	0	0	0	0	0	0	0	0	0	0	0	0
Methionine sulfoximine_Recover	0	0	0	0	0	0	0	0	0	0	0	0	0	0	0	0	0	0	0	0	0	0	0	0	0	0	0	0	0	0	0	0
Tyr_Recover	2.37	3.32	3.58	2.54	7.91	2.29	5.95	2.5	5.43	6.57	1.94	5.69	2.39	2.93	9.71	3.34	3.76	3.39	3	2.14	3.91	5.54	3.68	3.29	2.35	5.65	2.02	2.4	4.02	5.35	2.22	1.58
Harman_Recover	0	0	0	0	0	0	0	0	0	0	0	0	0	0	0	0	0	0	0	0	0	0	0	0	0	0	0	0	0	0	0	0
Phosphorylcholine_Recover	1.66	4.2	2.94	1.9	2.89	1.27	3.04	1.83	1.68	11.8	1.72	3.84	1.86	2	6.36	1.66	4.75	1.75	3.01	1.52	1.89	3.15	2.81	1.88	2.14	1.71	3.43	1.42	2.22	2.59	3.63	1.62
Epinephrine_Recover	0	0	0	0	0	0	0	0	0	0	0	0	0	0	0	0	0	0	0	0	0	0	0	0	0	0	0	0	0	0	0	0
N-α,N-α-Dimethylhistidine_Recover	0	0	0	0	0	0	0	0	0	0	0	0	0	0	0	0	0	0	0	0	0	0	0	0	0	0	0	0	0	0	0	0
N^8-Acetylspermidine_Recover	0	0.0227	0.015	0	0.015	0.014	0	0	0	0.0371	0	0.0115	0.00702	0.00855	0.0154	0	0.0167	0.0119	0.0131	0	0.0147	0.0126	0.00716	0.00947	0	0.0104	0	0.0107	0	0.0108	0	0.00834
N^1-Acetylspermidine_Recover	0.0578	0.176	0.133	0.0302	0.104	0.189	0.0852	0.081	0.153	0.218	0.101	0.0636	0.0423	0.0535	0.06	0.0803	0.201	0.223	0.19	0.0671	0.137	0.072	0.0342	0.112	0.145	0.0804	0.0889	0.117	0.0417	0.0888	0.058	0.0759
Gly-Leu_Recover	0.0229	0.0679	0.0292	0.0239	0.0435	0.0389	0.0808	0.079	0.0323	0.129	0.0334	0	0.0685	0.068	0.0893	0.0323	0.0546	0.0723	0.124	0.0266	0.096	0.0267	0.0165	0.116	0.064	0.064	0.0397	0.0222	0.131	0.0256	0.0549	0
N-ε-Acetyllysine_Recover	0.0437	0.0444	0.067	0.0376	0.0668	0.0521	0.0437	0	0	0.0897	0.0612	0.0608	0.0375	0.0398	0	0.0492	0.0921	0.0591	0	0.033	0.0578	0	0.0374	0.0358	0.0817	0.0437	0.0405	0.0372	0.052	0.109	0	0
N^6,N^6,N^6-trimethyllysine_Recover	0	0.0407	0	0.0169	0.00643	0.0284	0	0	0.0142	0.0488	0.0399	0.0188	0.0178	0.0237	0.0603	0	0.0333	0.0355	0.0391	0.0236	0.0246	0.0323	0.0187	0.0337	0.0307	0	0	0	0.0186	0.0146	0.0199	0
Castanospermine_Recover	0	0	0	0	0	0	0	0	0	0	0	0	0	0	0	0	0	0	0	0	0	0	0	0	0	0	0	0	0	0	0	0
5-Methoxytryptamine_Recover	0	0	0	0	0	0	0	0	0	0	0	0	0	0	0	0	0	0	0	0	0	0	0	0	0	0	0	0	0	0	0	0
5-Hydroxyindoleacetate_Recover	0	0	0	0	0	0	0	0	0	0	0	0	0	0	0	0	0	0	0	0	0	0	0	0	0	0	0	0	0	0	0	0
SAH_Recover	0	0	0	0	0	0	0	0	0	0	0	0	0	0	0	0	0	0	0	0	0	0	0	0	0	0	0	0	0	0	0	0
Glucosaminate_Recover	0	0	0	0	0	0	0	0	0	0	0	0	0	0	0	0	0	0	0	0	0	0	0	0	0	0	0	0	0	0	0	0
DOPA_Recover	0	0	0	0	0	0	0	0	0	0	0	0	0	0	0	0	0	0	0	0	0	0	0	0	0	0	0	0	0	0	0	0
N-Acetylhistidine_Recover	0	0	0	0	0	0	0	0	0	0	0	0	0	0	0	0	0	0	0	0	0	0	0	0	0	0	0	0	0	0	0	0
SAM+_Recover	0	0.0628	0.0239	0	0	0.0338	0.0223	0.0228	0.0225	0.0389	0.0198	0	0.0131	0	0.0301	0	0.0304	0.0237	0	0.0169	0.0219	0.017	0	0.0204	0.0157	0	0.0266	0	0.0126	0	0.0193	0.0201
Arginine ethyl ester_Recover	0	0	0	0	0	0	0	0	0	0	0	0	0	0	0	0	0	0	0	0	0	0	0	0	0	0	0	0	0	0	0	0
ADMA_Recover	0	0.0243	0	0.011	0	0.0224	0.0214	0.0143	0.0239	0.0268	0.0163	0	0.00728	0	0.025	0	0.0159	0	0	0	0.0194	0.0287	0	0	0	0.0232	0	0	0	0.0185	0	0
SDMA_Recover	0	0.0213	0	0.0109	0	0.0243	0	0	0	0.0261	0	0	0.0152	0	0.0064	0.0118	0	0	0.019	0	0.0129	0	0	0	0.0185	0	0	0	0	0.0205	0	0
o-Acetylcarnitine_Recover	0.322	0.948	0.269	0.358	0.377	0.37	0.558	0.326	0.744	0.721	0.25	0.944	0.352	0.301	0.772	0.231	0.758	0.285	0.432	0.301	0.367	0.322	0.446	0.381	0.42	0.634	0.192	0.575	0.362	0.468	0.301	0.182
Trp_Recover	0.122	0.209	0.141	0.126	0.166	0.2	0.267	0.21	0.163	0.326	0.111	0.0883	0.129	0.173	0.355	0.14	0.255	0.211	0.184	0.0777	0.205	0.125	0.133	0.133	0.155	0.163	0.14	0.079	0.223	0.0395	0.191	0.0874
5-Methoxyindoleacetate_Recover	1.19	0.756	0.576	1.05	1.39	0.763	0.955	0.831	1.14	0.918	1.15	0	0.727	0.58	1.71	0.656	0.854	0.784	0.638	0.972	0.559	0.881	0.718	0.681	1.04	0	0.499	1.15	0.996	0	0.674	0.84
Kynurenine_Recover	0	0	0	0	0	0	0	0	0	0	0	0	0	0	0	0	0	0	0	0	0	0.111	0	0	0	0	0	0	0	0	0	0
Cysteine-glutathione disulfide_Recover	0	0.355	0.1	0	0.0434	0.119	0.0505	0.191	0	0	0	0	0.0864	0.0957	0.534	0	0	0	0	0.0925	0.0702	0.124	0	0.104	0.0661	0.147	0.178	0	0	0	0	0.0478
Desthiobiotin_Recover	0	0	0	0	0	0	0	0	0	0	0	0	0	0	0	0	0	0	0	0	0	0	0	0	0	0	0	0	0	0	0	0
o-Succinylhomoserine_Recover	0	0	0	0	0	0	0	0	0	0	0	0	0	0	0	0	0	0	0	0	0	0	0	0	0	0	0	0	0	0	0	0
trans-Zeatin_Recover	0	0	0	0	0	0	0	0	0	0	0	0	0	0	0	0	0	0	0	0	0	0	0	0	0	0	0	0	0	0	0	0
5-Hydroxytryptophan_Recover	0	0	0	0	0	0	0	0	0	0	0	0	0	0	0	0	0	0	0	0	0	0	0	0	0	0	0	0	0	0	0	0
N-Acetylglucosylamine_Recover	0	0	0	0	0	0	0	0	0	0.173	0	0	0	0	0	0	0	0.0665	0	0	0	0	0	0.0497	0	0	0	0	0	0	0	0
N-Acetylglucosamine_Recover	2.43	2.02	7.13	2.22	2.24	4.99	5.06	2.31	6.94	26.9	1.59	10.7	1.24	3.6	1.97	2.91	1.31	4.63	5.14	3.22	19.5	8.95	6.73	17	2.46	13	1.51	1.15	4.86	7.17	3.08	1.13
Cystathionine_Recover	0	0	0	0	0	0	0	0	0	0	0	0	0	0	0	0	0	0	0	0	0	0	0	0	0	0	0	0	0	0	0	0
3-Hydroxykynurenine_Recover	0	0	0	0	0	0	0	0	0	0	0	0	0	0	0	0	0	0	0	0	0	0	0	0	0	0	0	0	0	0	0	0
2'-Deoxycytidine_Recover	0	0	0	0.0122	0.039	0	0	0	0	0	0	0	0	0	0.0167	0	0	0	0.0246	0	0	0	0	0	0	0.0275	0	0	0	0	0	0
N^1,N^8-Diacetylspermidine_Recover	0	0.0445	0.0115	0	0	0.00791	0.0163	0.019	0.0201	0.0304	0.0349	0.0239	0.0196	0.0197	0.015	0.0139	0.0152	0.028	0.0123	0.0115	0.0192	0.018	0.00844	0.0159	0	0.017	0	0	0	0.0159	0.00884	0.00466
γ-Glu-2AB_Recover	0	0	0	0	0	0	0	0	0	0	0	0	0	0	0	0	0	0	0	0	0	0	0	0	0	0	0	0	0	0	0	0
Melatonin_Recover	0	0.0833	0	0	0	0	0	0	0	0	0	0	0.0364	0	0	0.0662	0	0	0	0	0	0	0	0	0	0	0.0182	0	0	0	0.0627	0
Sepiapterin_Recover	0	0	0	0	0	0	0	0	0	0	0	0	0	0	0	0	0	0	0	0	0	0	0	0	0	0	0	0	0	0	0	0
7,8-Dihydrobiopterin_Recover	0	0	0	0	0	0	0	0	0	0	0	0	0	0	0	0	0	0	0	0	0	0	0	0	0	0	0	0	0	0	0	0
Cystine_Recover	0	0.104	0.0931	0	0.0583	0	0.0432	0	0	0	0.037	0	0.0532	0.0408	0.152	0	0.132	0	0.0612	0	0	0	0.0315	0	0.0371	0.0422	0.121	0	0	0	0.0654	0
5-Methyl-2'-deoxycytidine_Recover	0	0	0	0	0	0	0	0	0	0	0	0	0	0	0	0	0	0	0	0	0	0	0	0	0	0	0	0	0	0	0	0
Thymidine_Recover	0	0	0.865	0	1.39	0.955	0	0	0	0.963	0	0	0	0.532	0.902	0.599	0.905	0.81	0.938	0	0	0.707	0	0.917	0.904	0	0	0.982	0	0	0.86	0.669
Cytidine_Recover	0.0703	0	0.0943	0.0929	0.0564	0.105	0.0887	0	0.117	0.356	0.214	0.219	0	0.128	0	0.116	0.0295	0.11	0.226	0.0486	0.169	0.0628	0.133	0.332	0.127	0.159	0.0195	0.0358	0.142	0.2	0	0
Uridine_Recover	1.36	2.02	1.27	1.34	1.97	0.91	1.86	1.52	1.52	1.62	1.11	2.78	1.15	1.02	2.05	0.962	2.05	1.75	1.85	1.61	1.67	1.12	1.34	1.97	2.24	2.04	1.38	1.55	2.14	1.92	1.62	1.18
N^1-Acetylspermine_Recover	0	0	0.0202	0	0.0569	0.0243	0.0404	0	0	0.0295	0	0	0.0251	0.0112	0.0129	0	0	0.0313	0.019	0	0.0229	0.0161	0	0.023	0	0	0	0.0197	0	0	0	0.0112
Octopine_Recover	0	0	0	0.0102	0	0	0.0139	0.0469	0	0.128	0.0351	0	0	0	0	0.0334	0	0.0383	0	0	0	0	0	0	0.0489	0	0	0	0.0419	0	0.0536	0
Pyridoxamine 5'-phosphate_Recover	0	0	0	0	0	0	0	0	0	0	0	0	0	0	0	0	0	0	0	0	0	0	0	0	0	0	0	0	0	0	0	0
γ-Glu-cys_Recover	0	0	0	0	0	0	0	0	0	0	0	0	0	0	0	0	0	0	0	0	0	0	0	0	0	0	0	0	0	0	0	0
Muramate_Recover	0	0	0	0	0	0	0	0	0	0	0	0	0	0	0	0	0	0	0	0	0	0	0	0	0	0	0	0	0	0	0	0
5'-Deoxyadenosine_Recover	0	0	0	0	0	0	0	0	0	0	0.0152	0	0	0	0	0	0	0	0	0	0	0	0	0	0	0	0	0	0	0	0	0
Purine riboside_Recover	0	0	0	0	0	0	0	0	0	0	0	0	0	0	0	0	0	0	0	0	0	0	0	0	0	0	0	0	0	0	0	0
2'-Deoxyinosine_Recover	0	0	0	0	0	0	0	0	0	0	0	0	0	0	0	0	0	0	0	0	0	0	0	0	0	0	0	0	0	0	0	0
7,8-Dihydroneopterin_Recover	0	0	0	0	0	0	0	0	0	0	0	0	0	0	0	0	0	0	0	0	0	0	0	0	0	0	0	0	0	0	0	0
Glycerophosphorylcholine_Recover	0	0.797	0.432	0.457	1.33	0	0	0.243	0	0	0	0.636	1.15	0.613	2.74	0	0.33	0	0	0.483	0.22	0.447	0	0	0	0	0	0.886	0	0.429	0.601	0.325
Thiamine_Recover	0	0	0	0	0	0	0	0	0	0	0	0	0	0	0	0	0	0	0	0	0	0	0	0	0	0	0	0	0	0	0	0
N^6-Methyl-2'-deoxyadenosine_Recover	0	0	0	0	0	0	0	0	0	0	0	0	0	0	0	0	0	0	0	0	0	0	0	0	0	0	0	0	0	0	0	0
Adenosine_Recover	0.0754	0.13	0.0597	0.046	0.0328	0.0453	0.049	0.0673	0.0973	0.0625	0.312	0.151	0.0417	0.0752	0.124	0	0.106	0.0435	0.036	0.0461	0.0468	0.0687	0.0581	0.072	0.041	0.0388	0.125	0.04	0.0369	0.0796	0.0425	0.0537
2'-Deoxyguanosine_Recover	0	0	0	0	0	0	0	0	0	0	0	0	0	0	0	0	0	0	0	0	0	0	0	0	0	0	0	0	0	0	0	0

Homocystine_Recover	0	0	0	0	0	0	0	0	0	0	0	0	0	0	0	0	0	0	0	0	0	0	0	0	0	0	0	0	0	0	0	0
Inosine_Recover	0	0.606	0.367	0.156	0.245	0.547	0	0.441	0.265	0.195	0.275	0.232	0.183	0.281	0.265	0.146	0.484	0.278	0	0.222	0.612	0.315	0.254	0.43	0.355	0.532	0.321	0.191	0.233	0	0.302	0
Glu-Glu_Recover	0	0.0151	0.0403	0	0	0	0.048	0	0	0.126	0	0	0.0466	0.0298	0.0487	0.0489	0.0341	0.0512	0.073	0	0.0577	0	0	0	0	0	0	0	0	0	0	0
Saccharopine_Recover	0	0	0	0	0	0	0.0135	0	0	0.0829	0	0.0474	0	0	0.0722	0	0.101	0.0356	0	0	0.0504	0.017	0	0	0	0	0.0163	0	0	0	0	0.0494
1-Methyladenosine_Recover	0	0.0352	0.0171	0	0.0186	0.0175	0	0.0167	0.0133	0.026	0.0154	0.0306	0.0132	0.0213	0.0393	0.011	0.0192	0.0256	0	0.0145	0.0169	0	0	0	0.00956	0.00986	0.0181	0	0	0.0177	0.0193	0.0132
Guanosine_Recover	0.0725	0.261	0.195	0.0637	0.0979	0.285	0.108	0.193	0.141	0.0443	0.24	0.151	0.0665	0.101	0.172	0	0.145	0.112	0.0494	0.123	0.182	0.208	0.142	0.17	0.152	0.126	0.187	0.114	0	0.0795	0.127	0.0861
Xanthosine_Recover	0	0	0	0	0	0	0	0	0	0	0	0	0	0	0	0	0	0	0	0	0	0	0	0	0	0	0	0	0	0	0	0
Ophthalmate_Recover	0	0.0233	0	0	0	0	0	0	0	0	0	0	0	0	0.0464	0	0	0	0	0	0	0	0	0	0	0	0	0	0	0	0	0
5-Methylthioadenosine_Recover	0	0	0	0	0	0	0	0	0	0	0	0	0	0	0	0	0	0	0	0	0	0	0	0	0	0	0	0	0	0	0	0
Glutathione(ox)_Recover	0.0513	0.746	0.087	0.038	0.0608	0.12	0.0487	0.455	0.0582	0	0.0367	0.0662	0.059	0.0901	1.17	0.0308	0	0	0.0298	0.0518	0.0595	0.177	0.0196	0.0186	0.0193	0.0829	0.156	0.0316	0	0	0	0.0882
N-α-Benzenolarginine ethylester_Recover	0	0	0	0	0	0	0	0	0	0	0	0	0	0	0	0	0	0	0	0	0	0	0	0	0	0	0	0	0	0	0	0
3-Iodotyrosine_Recover	0	0	0	0	0	0	0	0	0	0	0	0	0	0	0	0	0	0	0	0	0	0	0	0	0	0	0	0	0	0	0	0
Glutathione(red)_Recover	0	0	0	0	0	0	0	0	0	0	0	0	0	0	0.253	0	0	0	0	0	0	0	0	0	0	0	0	0	0	0	0	0
Phe-Phe_Recover	0	0.0105	0	0	0	0	0	0.0112	0	0.0464	0	0.0139	0	0.0133	0.00862	0.00791	0	0	0.0306	0	0.0241	0	0	0.0251	0	0	0.00376	0.00702	0.0141	0	0.0178	0
AICAR_Recover	0	0.391	0.0316	0.445	0.784	0	0.372	0	0.577	0	0.524	0	0	0	0	0.269	0.294	0	0	0.354	0	0	0	0	0.566	0	0	0.474	0.524	0	0.237	0
Thiamine monophosphate_Recover	0	0	0	0	0	0	0	0	0	0	0	0	0	0	0	0	0	0	0	0	0	0	0	0	0	0	0	0	0	0	0	0
Tetrahydropalmatine_Recover	0	0	0	0	0	0	0	0	0	0	0	0	0	0	0	0	0	0	0	0	0	0	0	0	0	0	0	0	0	0	0	0
TRH_Recover	0	0	0	0	0	0	0	0	0	0	0	0	0	0	0	0	0	0	0	0	0	0	0	0	0	0	0	0	0	0	0	0
Riboflavin_Recover	0	0	0	0	0	0	0	0	0	0	0	0	0	0	0	0	0	0	0	0	0	0	0	0	0.415	0	0	0	0	0	0	0
S-Lactoylglutathione_Recover	0	0	0	0	0	0	0	0	0	0	0	0	0	0	0	0	0	0	0	0	0	0	0	0	0	0	0	0	0	0	0	0
Leu-Leu-Tyr_Recover	0	0	0	0	0	0	0	0	0	0	0	0	0	0	0	0	0	0	0	0	0	0	0	0	0	0	0	0	0	0	0	0
3,5-Diiodo-tyrosine_Recover	0	0	0	0	0	0	0	0	0	0	0	0	0	0	0	0	0	0	0	0	0	0	0	0	0	0	0	0	0	0	0	0
5-Methyltetrahydrofolate_Recover	0	0	0	0	0	0	0	0	0	0	0	0	0	0	0	0	0	0	0	0	0	0	0	0	0.396	0	0	0	0.622	0	0	0
3,3',5-Triiodothyronine_Recover	0	0	0	0	0	0	0	0	0	0	0	0	0	0	0	0	0	0	0	0	0	0	0	0	0	0	0	0	0	0	0	0
NMN_Recover	0	0	0	0	0	0	0	0	0	0	0	0	0	0	0	0	0	0	0	0	0	0	0	0	0	0	0.0588	0	0	0	0	0
Glyoxylate_Recover	0	0.579	0	0	1.2	0	0	0	0	0	0	0.347	0.664	0.497	0.386	0	0	0	0	0.834	0	0	0.443	0	0	0.802	1.03	0	0	0	0	0.508
Propionate_Recover	2.8	130	7.18	3.81	5.33	4.26	21.9	5.12	10	39.9	4.93	15.2	8.34	10.4	3.75	9.52	1.68	7.78	5.46	2.26	12.6	6.06	8.98	9.43	10.4	6.46	6.1	0.698	9.17	1.95	7.93	0.928
Glycolate_Recover	3.34	3.56	2.34	7.29	0	2.55	6.6	3.28	1.75	3.35	3.61	1.95	0	2.1	4.2	2.77	3.93	1.8	2.99	2.28	3	2.48	2.45	0	7.55	3.39	6.73	2.73	4.87	2.66	2.51	2.19
Crotonate_Recover	0	0	0	0	0	0	0.351	0	0	1.39	0	0	0	0	0	0	0	0	0	0	0	0	0	0	0	0.681	0	0	0	0	0	0
Pyruvate_Recover	2.33	10.6	1.77	2.98	3.56	2.83	3.34	2.77	3.86	5.73	3.84	3.48	1.81	1.35	3.53	3.23	2.83	2.71	3.12	2.36	3.51	2.25	1.62	3.42	5.55	2.94	1.88	2.65	4.88	3.73	1.73	0.864
Butyrate_Recover	0.975	22.6	1.69	0.96	0.84	1.99	8.23	2.1	2.54	10.3	1.13	4.04	2.14	2.57	1.51	2.73	0	2.34	1.58	1.1	3.1	0.89	1.65	3.73	2.47	2.2	1.13	0	3.59	0.619	2.58	0
Lactate_Recover	111	161	48.5	214	64.9	65.4	66.7	72	108	143	154	68.7	74.4	200	100	110	44.2	56.1	122	141	75.5	103	56.5	99	187	150	33.7	63	122	94.3	31.3	67.7
3-Hydroxypropionate_Recover	9.52	16	8.11	16.2	7.49	6.52	0	8.76	10.3	13	11.8	5.72	8.47	13.6	9.13	12.1	4.88	8.13	15.6	11.7	8.7	9.75	6.66	9.28	30.4	12.5	6.46	5.87	17.2	9.22	7.08	7.84
Methanesulfonate_Recover	0	0	0	0	0	0	0	0	0	0	0	0	0	0	0	0	0	0	0	0	0	0	0	0	0	0	0	0	0	0	0	0
Tiglate_Recover	0	0	0	0	0	0	0	0	0	0	0	0	0	0	0	0	0	0	0	0	0	0	0	0	0	0	0	0	0	0	0	0
2-Oxobutyrate_Recover	0	0	0	0	0	0	0	0	0	0	0	0	0	0	0	0	0	0	0	0	0	0	0	0	0	0	0	0	0	0	0	0
Pentanoate_Recover	0	0	0	0	0	0	0	0	0	0	0	0	0	0	0	0	0	0	0	0	0	0	0	0	0	0	0	0	0	0	0	0
3-Methylbutanoate_Recover	0	4.69	0.446	0	0	0.484	2.81	0.522	1.13	2.91	0.551	0.826	0	0.659	0	0.821	0	0.907	1.23	0	1.27	0	0.347	0.992	1.22	0.488	0	0	1.45	0	0	0
Malonate_Recover	0.237	0.327	0.296	0.166	0.248	0.279	0.265	0.175	0.33	0.361	0.366	0.36	0.282	0.285	0.291	0.159	0.259	0.27	0.611	0.48	0.221	0.391	0.251	0.257	0.276	0.479	0.137	0.169	0.182	0.137	0.171	0.134
2-Hydroxyisobutyrate_Recover	0	0	0	0	0	0	0	0	0	0	0	0	0	0	0	0	0	0	0	0	0	0	0	0	0	0	0	0	0	0	0	0
2-Hydroxybutyrate_Recover	0.29	0.498	0.133	0.437	0	0.402	0.798	0.349	0.407	0.551	0.329	0.303	0.544	0.573	0.377	0.641	1	0.23	0.605	0.568	0.352	0.25	0.138	0.662	0.593	0.42	0	0.343	0.925	0.303	0.127	0.167
3-Hydroxybutyrate_Recover	1.1	10.1	0.944	1.82	0.668	2.73	2.17	0.76	1.44	2.15	2	1.99	1.55	1.98	1.76	3.47	1.14	1.01	2.62	1.8	1.05	1.62	0.983	1.86	3.47	3.14	0.851	0.716	2.75	0.893	0.46	0.82
(Methylthio)acetate_Recover	0	0	0	0	0	0	0	0	0	0	0	0	0	0	0	0	0	0	0	0	0	0	0	0	0	0	0	0	0	0	0	0
Glycerate_Recover	0.633	1.65	0.608	1.33	0.92	0.851	1.4	1.12	1.17	0	0.789	1.22	0.982	1.01	1.39	0.88	1.25	0.529	0.951	0.494	0.891	0.952	0.693	1.1	0.867	1.47	0.816	0.523	1.2	0.864	1.02	0.212
Pyrrole-2-carboxylate_Recover	0	0	0	0	0	0	0	0	0	0	0	0	0	0	0	0	0	0	0	0	0	0	0	0	0	0	0	0	0	0	0	0
Methyl sulfate_Recover	0	0	0	0	0	0	0	0	0	0	0	0	0	0	0	0	0	0	0	0	0	0	0	0	0	0	0	0	0	0	0	0
2-Furoate_Recover	0.301	0	0	0	0	0	0	0	0	0	0	0	0	0	0	0	0	0	0	0	0	0	0	0	0	0	0	0	0	0	0	0
Fumarate_Recover	0.424	0.472	0.316	0.335	0.454	0.428	0.356	0.486	0.332	0.366	0.376	0.531	0.514	0.596	1.37	0.161	0.359	0.462	0.314	0.308	0.383	0.536	0.264	0.357	0.361	0.424	0.29	0.429	0.228	0.425	0.202	0.383
2-Oxoisopentanoate_Recover	0	0	0.0709	0	0	0.121	0	0	0	0.204	0	0.175	0	0.2	0.274	0	0	0.087	0.186	0	0.137	0.115	0.0985	0.208	0	0.155	0	0	0.535	0.105	0	0.0823
4-Oxopentanoate_Recover	0.286	0.27	0	0.4	0.247	0	0.106	0	0.201	0	0.321	0.0953	0.156	0.187	0.0767	0.392	0	0	0.281	0.305	0.175	0.252	0.0588	0.202	0.691	0.469	0	0	0.497	0.145	0	0.139
Hexanoate_Recover	0	0	0.153	0.12	0	0.319	0.659	0.353	0.182	2.01	0	0.265	0	0.316	0	0.499	0	0.501	0.494	0	0.353	0	0.15	0.551	0.691	0.377	0	0	0.627	0	0	0.0966
Succinate_Recover	13.4	6.68	22.6	21.4	19	26.1	22.4	16	7.73	19.5	10.9	25.3	19.3	17.7	8.37	13.3	4.89	10.3	15.9	10.9	21.9	16.3	13.5	19.2	11.7	10.9	8.25	7.36	22.1	30	8.02	9.27
2-Hydroxypentanoate_Recover	0.408	0.769	0.286	0.79	0.227	0.342	0.392	0.193	0.42	0.76	0.938	0.251	0.367	0.592	0.248	1	0.395	0.272	0.791	0.476	0.298	0.413	0.409	0.37	1.42	0.905	0.217	0.215	0.956	0.2	0	0.302
Benzoate_Recover	0	0	0	0	0	0.117	0	0	0	0	0.341	0.114	0	0.102	0	0	0	0.0929	0.105	0	0.192	0	0.219	0.318	0.515	0.388	0	0	0	0	0	0.228
Nicotinate_Recover	0.115	0.325	0.276	0	0	0.315	0.208	0.248	0.506	0.228	0.413	0	0	0.354	0.242	0.414	0.225	0.192	0.379	0	0.267	0.226	0.255	0.264	0.456	0.396	0.218	0	0.406	0	0.192	0
1-Aminoethylphosphonate_Recover	0	0	0	0	0	0	0	0	0	0	0	0	0	0	0	0	0	0	0	0	0	0	0	0	0	0	0	0	0	0	0	0
2-Aminoethylphosphonate_Recover	0	0	0	0	0	0	0	0	0	0	0	0	0	0	0	0	0	0	0	0	0	0	0	0	0	0	0	0	0	0	0	0
Isethionate_Recover	0	0.156	0.0704	0	0.149	0	0	0	0.128	0.201	0	0.0688	0	0	0.159	0	0	0	0	0	0	0	0	0	0	0	0	0	0	0.104	0	0
Barbiturate_Recover	0	0	0	0	0	0	0	0	0	0	0	0	0	0	0	0	0	0	0	0	0	0	0	0	0	0	0	0	0	0	0	0
5-Oxoproline_Recover	1.39	2.18	2.08	1.54	3.41	1.85	1.31	1.11	1.36	1.98	1.2	1.26	1.31	2.09	2.34	0.79	1.51	1.41	1.72	0.995	1.49	2.63	2.85	1.59	1.84	2.53	1.42	1.15	1.4	2.03	0.608	0.968
Itaconate_Recover	0	0	0	0	0	0	0	0	0	0	0	0	0	0	0	0	0	0	0	0	0	0	0	0	0	0	0	0	0	0	0	0
Citraconate_Recover	0	0	0	0	0	0	0	0	0	0	0	0	0	0	0	0	0	0	0	0	0	0	0	0	0	0	0	0	0	0	0	0
4-Methyl-2-oxopentanoate_Recover	0.275	0.485	0.196	0.275	0.204	0.279	0.293	0	0.451	0.461	0.44	0.262	0.228	0.291	0.351	0.474	0.196	0.233	0.424	0.228	0.293	0.252	0.265	0.457	0.51	0.361	0	0.272	0.542	0.268	0	0.156
4-Oxohexanoate_Recover	0	0	0	0	0	0	0	0	0	0	0	0	0	0	0	0	0	0	0	0	0	0	0	0	0	0	0	0	0	0	0	0
4-Acetylbutyrate_Recover	0.0685	0.201	0	0.115	0	0.0472	0	0	0.0846	0	0.0782	0	0	0.127	0	0.231	0	0	0.155	0.169	0.0641	0.0593	0.0491	0.0737	0.284	0.234	0	0	0.261	0.09	0	0.0616
Heptanoate_Recover	0	0	0	0	0	0	0	0	0	0	0	0	0	0	0	0	0	0	0	0	0	0	0	0	0	0	0	0	0	0	0	0
N-acetyl-*β*-alanine_Recover	0.168	0	0.201	0	0	0.147	0.135	0.141	0	0.35	0.112	0.141	0.133	0.169	0.337	0	0	0	0.197	0	0.151	0.169	0.175	0	0	0.194	0	0	0	0	0	0
Glutarate_Recover	0	0	0	0	0	0	0	0	0	0	0	0	0	0	0	0	0	0	0	0	0	0	0	0	0	0	0	0	0	0	0	0
3-Ureidopropionate_Recover	0	0	0	0	0	0	0	0	0	0	0	0	0	0	0	0	0	0	0	0	0	0	0	0	0	0	0	0	0	0	0	0
2-Hydroxy-4-methylpentanoate_Recover	0.213	0.184	0.317	0.384	0.158	0.406	0.394	0.148	0.438	0.834	0.672	0.175	0.191	0.337	0.0733	0.8	0	0.254	0.274	0.199	0.379	0.23	0.496	0.298	0.562	0.331	0	0	0.646	0.152	0	0.114
6-Hydroxyhexanoate_Recover	0	0	0	0	0	0.149	0	0	0	0	0	0	0	0.185	0	0.378	0	0	0.364	0.26	0	0	0	0	0.506	0.346	0	0	0	0	0	0
Malate_Recover	1.6	2.11	1.25	0.93	1.9	2.39	1.04	1.3	1.55	1.82	1.26	3.02	1.65	3.99	8.32	0.7	1.47	2.71	2.16	1.09	2.05	3.31	1.71	1.63	1.93	3.09	1.71	1.31	1.22	2.01	0.767	1.55
Threonate_Recover	0	0	0.503	0.375	0.825	0.46	0.376	0	0.475	0.609	0	0.802	0	0	0.906	0	0.651	0.307	0	0	0.604	0	0.82	0.352	0	0	0.437	0.325	0	0.625	0.38	0.253
o-Hydroxybenzoate_Recover	0	0	0	0	0	0	0	0	0	0	0	0	0	0	0	0	0	0.0483	0.159	0	0	0	0.0428	0	0	0.158	0.193	0	0	0.0341	0	0
p-Hydroxybenzoate_Recover	0	0.519	0	0	0	0	0	0	0.164	0	0.572	0.732	0	0	0.539	0	0	0	0	0.219	0	0	0	0	0	0.296	0	0	0	0	0	0
m-Hydroxybenzoate_Recover	0	0	0	0	0	0	0	0	0	0	0	0	0	0	0	0	0	0	0	0	0	0	0	0	0	0	0	0	0	0	0	0
Phosphonoacetate_Recover	0	0	0	0	0	0	0	0	0	0	0	0	0	0	0	0	0	0	0	0	0	0	0	0	0	0	0	0	0	0	0	0
Carbamoylphosphate_Recover	0	0	0	0	0	0	0	0	0	0	0	0	0	0	0	0	0	0	0	0	0	0	0	0	0	0	0	0	0	0	0	0
Ethanolamine phosphate_Recover	17.7	92.8	16.6	26.1	47.3	12.6	25.8	22.6	16.6	13.9	11.6	23.8	28.8	20.1	65.9	19.3	40.3	12.7	13.5	15.3	22.7	10.6	18.6	15.3	15.9	4.12	33.2	28.6	28.5	40.6	31.8	13.8
2-Thiopheneacetate_Recover	0	0	0	0	0	0	0	0	0	0	0	0	0	0	0	0	0	0	0	0	0	0	0	0	0	0	0	0	0	0	0	0
Octanoate_Recover	0	0	0	0	0	0	0	0	0	0	0	0	0	0	0	0	0	0	0	0	0	0	0	0	0	0	0	0	0	0	0	0
2-Oxoglutarate_Recover	0	0	0.278	0	0.609	0.43	0	0	0	0	0	0	0	0	0	0	0	0	0	0	0.271	0	0	0	0.754	0	0	0	0	1.26	0	0
Adipate_Recover	0	0	0	0	0	0	0	0	0	0	0	0	0	0	0	0	0	0	0	0	0	0	0	0	0	0	0	0	0	0	0	0
threo-*β*-methylaspartate_Recover	0	0	0	0	0	0	0	0	0	0	0	0	0	0	0	0	0	0	0	0	0	0	0	0	0	0	0	0	0	0	0	0
4-Methylthio-2-oxobutyrate_Recover	0	0	0	0	0	0	0	0	0	0	0	0	0	0	0	0	0	0	0	0	0	0	0	0	0	0	0	0	0	0	0	0
2-Hydroxyglutarate + Citramalate_Recover	0.395	0.222	0.319	0.586	0.463	0.474	0.55	0.28	0.348	0.906	0.37	0	0.452	0.471	0	0.396	0	0.49	0.3	0	0.333	0.477	0	0.289	0.538	0.58	0	0.257	0.426	0	0	0.24
trans-Cinnamate_Recover	0.286	0.42	0.66	0.313	0.362	0.376	0.434	0.271	0.328	0.768	0.268	0.623	0.235	0.543	1.12	0.287	0.354	0.428	0.346	0.217	0.446	0.461	0.329	0.368	0.333	0.559	0.359	0.22	0.295	0.503	0.227	0
Tartrate_Recover	0	0	0	0	0	0	0	0	0	0	0	0	0	0	0	0	0	0	0	0	0	0	0	0	0	0	0	0	0	0	0	0
Benzoylformate_Recover	0	0	0	0	0	0	0	0	0	0	0	0	0	0	0	0	0	0	0	0	0	0	0	0	0	0	0	0	0	0	0	0
3-Phenylpropionate_Recover	1.03	0.403	0.638	1.09	0.461	0.995	0.774	0.787	0.778	3.5	1.38	0.648	0.582	1.69	0.496	3.3	0.368	1.28	2.44	2.09	1.1	0.627	0.574	1.36	4.9	7.47	0.414	0.419	3.23	0.439	0.612	0.603
2-Hydroxyphenylacetate_Recover	0	0	0	0	0	0	0	0	0	0	0	0	0	0	0	0	0	0	0	0	0	0	0	0	0	0	0	0	0	0	0	0
p-Hydroxyphenylacetate_Recover	0	11.1	0.608	0.435	1.17	0.301	8.87	2.85	4.87	0	1.33	1.18	1.13	1.07	0	1.1	0.72	1.5	1.57	1.19	0.974	1.81	3.36	1.64	0.841	5.23	2.04	0	1.52	0	1.47	0

Cysteine sulfinate_Recover	0	0	0.294	0	0	0.19	0	0	0	0.239	0	0.261	0	0	0	0	0.427	0.26	0	0	0.307	0	0	0	0	0.273	0	0	0	0.301	0	0
2,5-Dihydroxybenzoate_Recover	0	0	0.306	0	0	0	0	0	0	0	0.667	0	0	0	0	0	0	0.213	0	0	0.525	0.45	0.459	0	0.842	0.239	0	0	0	0	0	0
Orotate_Recover	0	0	0	0	0	0	0	0	0	0	0	0	0	0	0	0.142	0	0	0	0	0	0	0	0	0	0	0	0	0	0	0	0
2,4-Dihydroxypyrimidine-5-carboxylate_Recover	0	0	0	0	0	0	0	0	0	0	0	0	0	0	0	0	0	0	0	0	0	0	0	0	0	0	0	0	0	0	0	0
Dihydroorotate_Recover	0	0	0	0	0	0	0	0	0	0	0	0	0	0	0	0	0	0	0	0	0	0	0	0	0	0	0	0	0	0	0	0
2-Oxooctanoate_Recover	0	0	0	0	0	0	0	0	0	0	0	0	0	0	0	0	0	0	0	0	0	0	0	0	0	0	0	0	0	0	0	0
Pelargonate_Recover	0.031	0	0	0	0	0	0	0	0	0	0	0	0	0	0	0	0	0	0	0	0	0	0	0	0	0	0	0	0	0	0	0
2-Oxoadipate_Recover	0	0	0	0	0	0	0	0	0	0	0	0	0	0	0	0	0	0	0	0	0	0	0	0	0	0	0	0	0	0	0	0
Pimelate_Recover	0	0	0	0	0	0	0	0	0	0	0	0	0	0	0	0	0	0	0	0	0	0	0	0	0	0	0	0	0	0	0	0
2-Hydroxyoctanoate_Recover	0	0	0	0	0	0	0	0	0	0	0	0	0	0	0	0	0	0	0	0	0	0	0	0	0	0	0	0	0	0	0	0
N-Formylaspartate_Recover	0	0	0	0	0	0	0	0	0	0	0	0	0	0	0	0	0	0	0	0	0	0	0	0	0	0	0	0	0	0	0	0
3-Hydroxy-3-methylglutarate_Recover	0.0737	0.0394	0.0504	0.0965	0.0707	0.0562	0	0	0.0423	0.0303	0.0511	0	0	0	0	0.0635	0.0729	0.0302	0.0632	0	0.0211	0.0445	0.0481	0	0.0881	0	0.0533	0	0	0	0	0
Phenylpyruvate_Recover	0	0	0	0	0	0	0	0	0	0	0	0	0	0	0	0	0	0	0	0	0	0	0	0	0	0	0	0	0	0	0	0
p-Coumarate_Recover	0	0	0	0	0	0	0	0	0	0	0	0	0	0	0	0	0	0	0	0	0	0	0	0	0	0	0	0	0	0	0	0
o-Coumarate_Recover	0	0	0	0	0	0	0	0	0	0	0	0	0	0	0	0	0	0	0	0	0	0	0	0	0	0	0	0	0	0	0	0
Terephthalate_Recover	0	0	0	0	0	0	0	0	0	0	0	0	0	0	0	0	0	0	0	0	0	0	0	0	0	0	0	0	0	0	0	0
Phthalate_Recover	0.164	0.0837	0.151	0.111	0.0979	0.167	0.1	0.101	0.134	0.0697	0.0879	0.0795	0.106	0.145	0.0408	0.0975	0.0981	0.0756	0.15	0.0807	0.109	0.17	0.133	0.0756	0.149	0.187	0.0994	0.121	0.105	0.0681	0.108	0.117
3-Phenyllactate_Recover	0.22	0	0.358	0.347	0.253	0.4	0.287	0.193	0.224	0.797	0.35	0.255	0.181	0.411	0	0.251	0	0.335	0.31	0	0.347	0.239	0.315	0.31	0.293	0.294	0.0935	0.126	0.32	0.17	0.0948	0.0967
3-(2-Hydroxyphenyl)propionate_Recover	0	0	0	0	0	0	0	0	0	0	0	0	0	0	0	0	0	0	0	0	0	0	0	0	0	0	0	0	0	0	0	0
3-(4-Hydroxyphenyl)propionate_Recover	1.04	7.24	1.51	0.78	0.499	0.531	6.79	2.44	2.47	3.38	2.51	1.43	1.54	1.18	0	2.43	0	1.56	1.79	0.967	2.1	1.76	3.61	1.66	2.16	2.05	1.64	0	1.42	0.179	3.66	0
2,3-Pyridinedicarboxylate_Recover	0	0	0	0	0	0	0	0	0	0	0	0	0	0	0	0	0	0	0	0	0	0	0	0	0	0	0	0	0	0	0	0
PEP_Recover	0.145	0.508	0	0	0.198	0.105	0	0.331	0.172	0.243	0.298	0.19	0.267	0.237	1.52	0.134	0.541	0.155	0	0.163	0.134	0.206	0	0.2	0.234	0.193	0.174	0	0	0.183	0.176	0.249
Urate_Recover	43.5	101	62	31.8	86.3	75.5	61.4	30.4	54.5	108	68.8	99.5	33.2	49.1	229	32	141	62	121	48.3	68.9	38.9	49.1	50.3	44.4	28.9	74	48	120	111	43.3	29.2
4-Hydroxymandelate_Recover	0.293	4.7	0.725	0	0.814	0.451	4.37	0.953	2.61	0.67	0.485	0.589	0.459	1.28	0	0.559	0.379	0.714	1.67	0.534	0.883	1.97	1.94	0.727	0.593	2.8	0	0	1.39	0	0	0
Cysteate_Recover	0	0	0	0	0	0	0	0	0	0	0	0	0	0	0	0	0	0	0	0	0	0	0	0	0.135	0	0	0	0	0	0	0
2-Amino-3-phosphonopropionate_Recover	0	0	0	0	0	0	0	0	0	0	0	0	0	0	0	0	0	0	0	0	0	0	0	0	0	0	0	0	0	0	0	0
DHAP_Recover	0	4.1	0	0	0.229	0	0.173	0.318	0	0.467	0	0.246	0.258	0.216	0	0	0.47	0	0.3	0	0	0	0	0.129	0	0.251	0	0	0	0	0	0
G3P_Recover	0	0	0	0	0	0	0	0	0	0	0	0	0	0	0	0	0	0	0	0	0	0	0	0	0	0	0	0	0	0	0	0
Glycerophosphate_Recover	2.51	8.02	2.33	4.97	6.29	3.13	4.14	3.84	3.67	10.8	4.18	7.1	3.85	5.01	10.4	2.92	12.7	4.19	4.29	3.44	6.2	2.41	3.47	5.47	3.86	5.1	6.26	3.97	4.58	5.65	4.81	2.72
Decanoate_Recover	0	0	0	0	0	0	0	0	0	0	0	0	0	0	0	0	0	0	0	0	0	0	0	0	0	0	0	0	0	0	0	0
2-Quinolinecarboxylate_Recover	0	0	0	0	0	0	0	0	0	0	0	0	0	0	0	0	0	0	0	0	0	0	0	0	0	0	0	0	0	0	0	0
N-Acetylleucine_Recover	0	0	0	0	0.0507	0	0	0	0	0	0	0	0	0	0	0	0	0	0	0	0	0	0	0	0	0	0	0	0	0	0	0
Phenyl phosphate_Recover	0	0	0	0	0	0	0	0	0	0	0	0	0	0	0	0	0	0	0	0	0	0	0	0	0	0	0	0	0	0	0	0
trans-Aconitate_Recover	0	0	0	0	0	0	0	0	0	0	0	0	0	0	0.0505	0	0	0	0	0	0	0	0	0	0	0	0	0	0	0	0	0
cis-Aconitate_Recover	0.0728	0.218	0.0751	0.0643	0.181	0.175	0.0995	0	0.146	0.0933	0	0.166	0.0468	0.133	0.538	0.0488	0.0718	0.071	0.0762	0.0843	0.0689	0.0453	0.0717	0.0664	0.0783	0.0796	0	0.119	0.0623	0.213	0	0
Shikimate_Recover	0	0	0	0	0.228	0	0	0	0	0.456	0	0	0	0	0	0	0	0	0	0	0	0.2	0	0	0	0.29	0	0	0	0	0	0
N-Acetylaspartate_Recover	0.205	0.183	0.908	0.3	1.63	0.369	0.735	0.152	0.478	0.56	0.117	1.09	0.136	0.249	1.76	0.263	0.289	0.386	0.317	0.332	0.315	0.302	0.52	0.436	0.231	1.24	0.169	1.25	0.294	2.2	0.176	0.226
Carbamoylaspartate_Recover	0	0	0	0	0	0	0	0	0	0.185	0	0	0	0	0	0	0	0	0	0	0.115	0	0	0	0	0	0	0	0	0	0	0
Allantoate_Recover	0	0	0	0	0	0	0	0	0	0	0	0	0	0	0	0	0	0	0	0	0	0	0	0	0	0	0	0	0	0	0	0
2-Isopropylmalate_Recover	0.0937	0.0735	0.042	0.103	0.167	0.0537	0	0	0.0533	0.197	0.133	0.0713	0.0973	0.128	0.0313	0	0.0689	0.0512	0.259	0	0.0535	0.143	0.0655	0.0334	0.231	0.155	0.0544	0	0.0429	0.136	0	0.0587
N-Formylmethionine_Recover	0	0	0	0	0	0	0	0	0	0	0	0	0	0	0	0	0	0	0	0	0	0	0	0	0	0	0	0	0	0	0	0
Hippurate_Recover	0	0	0	0	0	0	0	0	0	0	0	0	0	0	0	0	0	0	0	0	0	0	0	0	0	0	0	0	0	0	0	0
Homovanillate_Recover	0.389	0.64	0.77	0.609	0.667	0.608	0.608	0.335	0	1.54	0.461	0.709	0.442	0.919	0.24	0.369	0	0.653	0.834	0	0.67	0.78	1.06	0.672	0.441	0.736	0	0	0.505	0.686	0	0
4-Pyridoxate_Recover	0	0	0	0	0	0	0	0	0	0	0	0	0	0	0	0	0	0	0	0	0	0	0	0	0	0	0	0	0	0	0	0
Serine O-sulfate_Recover	0	0	0	0	0	0	0	0	0	0	0	0	0	0	0	0	0	0	0	0	0	0	0	0	0	0	0	0	0	0	0	0
O-Phosphoserine_Recover	0	0	0	0	0	0	0.151	0	0	0.152	0	0	0	0.067	0	0	0	0	0.113	0	0.109	0	0	0.082	0	0.123	0.106	0	0	0.117	0	0.0438
3PG + 2PG_Recover	0.322	1.88	0.159	0	0.394	0.463	0.612	1.09	0.657	0.657	0.896	0.831	0.713	0.732	7.21	0.393	1.85	0.761	0.524	0.446	0.76	0.822	0.323	0.757	0.619	0.52	0.671	0.63	0.36	0.445	0.924	1.02
Undecanoate_Recover	0	0	0	0	0	0	0	0	0	0	0	0	0	0	0	0	0	0	0	0	0	0	0	0	0	0	0	0	0	0	0	0
Azelate_Recover	0.11	0.179	0.14	0.166	0.227	0.153	0.172	0.198	0.125	0.1	0.131	0.094	0.126	0.156	0.0995	0.148	0.0767	0.108	0.0855	0.104	0.123	0.181	0.152	0.115	0.0829	0.108	0.13	0.156	0.131	0.107	0.102	0.0979
10-Hydroxydecanoate_Recover	0	0	0	0	0	0	0	0	0	0	0	0	0	0	0	0	0	0	0	0	0	0	0	0	0	0	0	0	0	0	0	0
N-Acetylglutamate_Recover	0	0.083	0.086	0	0.0596	0	0	0	0	0.132	0.0659	0.0652	0	0.0457	0.0732	0	0.0644	0.0564	0.104	0	0	0.0497	0.0472	0.0498	0	0.0823	0	0	0.0733	0.0807	0	0
N-Carbamylglutamate_Recover	0	0	0	0	0	0	0	0	0	0	0	0	0	0	0	0	0	0	0	0	0	0	0	0	0	0	0	0	0	0	0	0
N-Acetylmethionine_Recover	0	0	0	0	0	0	0	0	0	0	0	0	0	0	0	0	0	0	0	0	0	0	0	0	0	0	0	0	0	0	0	0
Isocitrate_Recover	0	0.281	0.174	0	0.181	0.282	0.19	0	0.262	0.218	0	0.272	0	0.265	0.819	0	0	0	0.302	0	0.156	0.297	0.228	0	0	0.248	0	0	0	1.76	0	0
Citrate_Recover	1.26	3.8	1.1	0.592	3.2	3.28	1.49	0.485	3.89	2.29	0.518	4.31	0.755	3.49	17.4	0.965	1.91	1.07	1.1	1.59	1.28	0.662	2.05	1.44	1.35	3.24	0.481	2.55	1.1	5.19	0.581	0.388
Quinate_Recover	0.907	0	0	0	0.278	0.216	0	0	0	0	0	0	0	0.85	0	0	0	0	0	0	0	0.167	0	0	0	0.643	0.582	0.359	0	0	0.41	0
Phenaceturate_Recover	0	0	0	0	0	0	0	0	0	0	0	0	0	0	0	0	0	0	0	0	0	0	0	0	0	0	0	0	0	0	0	0
Glucuronate_Recover	0	0	0.145	0	0	0	0	0	0	0	0	0.206	0	0	0	0	0	0	0	0	0	0	0	0	0	0	0	0	0	0.167	0	0
trans-4-Hydroxy-3-methoxycinnamate_Recover	0	0	0	0	0	0	0	0	0	0	0	0	0	0	0	0	0	0	0	0	0	0	0	0	0	0	0	0	0	0	0	0
o-Hydroxyhippurate_Recover	0	0	0	0	0	0	0	0	0	0	0	0	0	0	0	0	0	0	0	0	0	0	0	0	0	0	0	0	0	0	0	0
Gluconate_Recover	0	0	0	0	0	0	0	0	0	0	0	0	0	0	0	0	0	0	0	0	0	0	0	0	0	0	0	0	0	0	0	0
Syringate_Recover	0.44	0.321	0.465	0.412	0.245	0.468	0	0.44	0.407	0	0.336	0.124	0.459	0.472	0	0.342	0.327	0.415	0.378	0.426	0.339	0.588	0.451	0.319	0.333	0.548	0	0	0.491	0.365	0	0.422
4-Hydroxy-3-methoxymandelate_Recover	0	0	0.351	0	0	0.275	0	0	0	0.603	0	0	0	0.409	0	0	0	0.218	0.313	0	0.219	0.401	0.495	0	0	0.356	0	0	0	0	0	0
Dodecanoate_Recover	0	0	0	0	0	0	0	0	0	0	0	0	0	0	0	0	0	0	0	0	0	0	0	0	0	0.0836	0	0	0	0	0	0
Cysteine S-sulfate_Recover	0	0	0	0	0	0	0	0	0	0	0	0	0	0	0	0	0	0	0	0	0	0	0	0	0	0	0	0	0	0	0	0
Sebacate_Recover	0.0133	0	0.0164	0.0187	0	0	0	0	0	0	0	0.0245	0	0.0146	0	0.0256	0	0	0	0.033	0	0	0.0133	0	0	0.0145	0	0	0	0	0	0
3-Indolebutyrate_Recover	0	0	0	0	0	0	0	0	0	0	0	0	0	0	0	0	0	0	0	0	0	0	0	0	0	0	0	0	0	0	0	0
Xanthurenate_Recover	0	0	0	0	0	0	0	0	0	0	0	0	0	0	0	0	0	0	0	0	0	0	0	0	0	0	0	0	0	0	0	0
N-Acetylphenylalanine_Recover	0	0	0	0	0	0	0	0	0	0	0	0	0	0	0	0	0	0	0	0	0	0	0	0	0	0	0	0	0	0	0	0
Benzylsuccinate_Recover	0	0	0	0	0	0	0	0	0	0	0	0	0	0	0	0	0	0	0	0	0	0	0	0	0	0	0	0	0	0	0	0
Saccharate_Recover	0	0	0	0	0	0	0	0	0	0	0	0	0	0	0	0	0	0	0	0	0	0	0	0	0	0	0	0	0	0	0	0
Mucate_Recover	0	0	0	0	0	0	0	0	0	0	0	0	0	0.353	0	0	0	0	0	0	0	0	0	0	0	0.0831	0	0	0	0	0	0
3-Indoxyl sulfate_Recover	0	0	0	0	0	0	0	0	0	0	0	0	0	0	0	0	0	0	0	0	0	0	0	0	0	0	0	0	0	0	0	0
2-Deoxyribose 1-phosphate_Recover	0	0	0	0	0	0	0	0	0	0	0	0	0	0	0	0	0	0	0	0	0	0	0	0	0	0	0	0	0	0	0	0
Pantothenate_Recover	0	0.16	0.104	0	0	0	0	0	0	0	0	0.0855	0	0	0.201	0	0	0	0	0	0	0	0	0.165	0	0.104	0	0	0	0	0	0
Sinapate_Recover	0	0	0	0	0	0	0	0	0	0	0	0	0	0	0	0	0	0	0	0	0	0	0	0	0	0	0	0	0	0	0	0
Porphobilinogen_Recover	0	0	0	0	0	0	0	0	0	0	0	0	0	0	0	0	0	0	0	0	0	0	0	0	0	0	0	0	0	0	0	0
Ru5P_Recover	0.489	2.41	0.325	0.358	0.631	0.758	0.923	1.25	0.539	0.44	0.793	0.761	0.734	0.552	1.73	0.277	0.75	0.745	1.23	0.486	1.33	0.621	0.456	1.51	1.03	0.648	0.625	0.689	0.613	0.479	1.53	0.709
R5P_Recover	0	0.657	0	0	0	0	0	0	0	0	0	0	0	0	0	0	0	0	0	0	0	0	0	0	0	0	0	0	0	0	0	0
Dodecanedioate_Recover	0	0	0	0	0	0	0	0	0	0	0	0	0	0	0	0	0	0	0	0	0	0	0	0	0	0	0	0	0	0	0	0
2-Deoxyglucose 6-phosphate_Recover	0	0	0	0	0	0	0	0	0	0	0	0	0	0	0	0	0	0	0	0	0	0	0	0	0	0	0	0	0	0	0	0
Biotin_Recover	0	0	0.248	0	0	0.146	0.288	0	0	1.06	0	0.429	0.188	0.228	0.532	0	0	0.195	0.254	0.177	0	0.262	0.202	0	0	0.161	0	0	0	0.276	0	0.158
Glucosamine 6-phosphate_Recover	0	0	0	0	0	0	0	0	0	0	0	0	0	0	0	0	0	0	0	0	0	0	0	0	0	0	0	0	0	0	0	0
G1P_Recover	0.181	0.306	0.121	0.162	0.219	0.16	0.156	0.279	0.162	0.467	0.577	0.757	0.224	0.3	0.452	0.119	0.577	0.315	0.371	0.236	0.284	0.215	0.119	0.243	0.276	0.406	0.18	0.135	0.546	0.304	0.383	0.123
F6P_Recover	0.234	0.54	0.187	0.167	0.426	0.266	0.235	0.485	0.238	0.582	0.442	1.49	0.35	0.526	0.519	0.198	0.589	0.363	0.301	0.279	0.469	0.39	0.155	0.311	0.461	0.506	0.185	0.203	0.332	0.235	0.234	0.227
G6P_Recover	0.819	3.09	0.619	0.652	1.78	0.906	1.18	1.9	0.889	2.71	1.61	1.85	1.52	1.88	3.32	0.679	3	1.93	1.44	0.918	1.86	1.41	0.53	1.21	1.78	1.85	0.912	0.849	1.24	1.09	1.06	0.812
Sorbitol 6-phosphate_Recover	0	0	0	0	0	0	0	0	0	0	0	0	0	0	0	0	0	0	0	0	0	0	0	0	0	0	0	0	0	0	0	0
2,3-DPG_Recover	0	0	0	0	0	0	0	0	0	0	0	0	0	0	0	0	0	0	0	0	0	0	0	0	0	0	0	0	0	0	0	0
Galacturonate 1-phosphate_Recover	0	0	0.0476	0	0	0	0	0	0	0.261	0.0908	0.26	0	0	0.203	0	0	0	0.121	0.0582	0.0645	0	0	0.142	0.0905	0.0795	0	0.115	0.0779	0.26	0.0881	0
6-Phosphogluconate_Recover	0	0.144	0.071	0.0903	0.129	0.105	0.133	0.0823	0.0794	0.247	0	0.18	0	0.171	0.121	0	0	0	0.174	0	0.165	0.064	0.0931	0.262	0	0.239	0	0	0.187	0.145	0.132	0
S7P_Recover	0	0.716	0.164	0	0	0	0	0.438	0.318	0.508	0.285	0.377	0	0.444	0.6	0	0.71	0.317	0.488	0	0.204	0.355	0	0.262	0.442	0.588	0	0	0	0	0	0
Argininosuccinate_Recover	0	0	0	0	0	0	0	0	0	0	0	0	0	0	0	0	0	0	0	0	0	0	0	0	0	0	0	0	0	0	0	0

N-Acetylmuramate_Recover	0.146	0	0.096	0	0.887	0	0	0	0	0	0	0	0	0	0	0.344	0	0	0	0	0	0	0	0	0	0	0	0	0	0	0	0
N-Acetylglucosamine 1-phosphate_Recover	0.0849	0.171	0.0576	0	0	0.0675	0	0	0.0887	0.324	0.15	1.21	0	0.114	0	0.0755	0.229	0.122	0.103	0.0888	0.156	0.105	0.0793	0	0.145	0.161	0	0	0.621	0.136	0	0.082
N-Acetylglucosamine 6-phosphate_Recover	0	0.132	0	0	0	0	0	0.108	0	0.371	0.116	0	0.0677	0	0.219	0	0.153	0.134	0	0	0	0	0	0	0.0922	0.0925	0	0.106	0	0	0	0.0581
2',3'-cCMP_Recover	0	0	0	0	0	0	0	0	0	0	0	0	0	0	0	0	0	0	0	0	0	0	0	0	0	0	0	0	0	0	0	0
dCMP_Recover	0	0	0	0	0	0	0	0	0	0	0	0	0	0	0	0	0	0	0	0	0	0	0	0	0	0	0	0	0	0	0	0
dUMP_Recover	0	0	0	0	0	0	0	0	0	0	0	0	0	0	0	0	0	0	0	0	0	0	0	0	0	0	0	0	0	0	0	0
N-Acetylneuraminate_Recover	31.4	14.9	6.27	5.21	5.93	7.7	4.92	7.09	10.8	58.2	43.3	41.7	3.44	19.1	3.96	32.7	5.14	24.2	16	6.17	33.2	8.2	15.3	29.4	4.72	19.6	6.4	3.29	24.3	9.79	15.6	6.37
Ribulose 1,5-diphosphate_Recover	0	0	0	0	0	0	0	0	0	0	0	0	0	0	0	0	0	0	0	0	0	0	0	0	0	0	0	0	0	0	0	0
3',5'-Cyclic dAMP_Recover	0	0	0	0	0	0	0	0	0	0	0	0	0	0	0	0	0	0	0	0	0	0	0	0	0	0	0	0	0	0	0	0
dTMP_Recover	0	0	0	0	0	0	0	0	0	0	0	0	0	0	0	0	0	0	0	0	0	0	0	0	0	0	0	0	0	0	0	0
CMP_Recover	0.16	0.581	0.184	0.159	0.313	0	0.114	0.273	0	0	0.254	0.163	0.213	0.247	0.587	0	0.422	0.271	0.196	0.16	0.159	0.21	0.127	0.179	0.348	0.214	0	0.403	0.165	0	0.22	0.281
UMP_Recover	0.13	0.351	0.176	0.178	0.307	0.0823	0.222	0.337	0	0	0.299	0.707	0.37	0.225	0.767	0	0.428	0.317	0.331	0.229	0.223	0.196	0	0.307	0.388	0.259	0.227	0.214	0.324	0	0	0.264
cAMP_Recover	0	0	0	0	0	0	0	0	0	0	0	0	0	0	0	0	0	0	0	0	0	0	0	0	0	0	0	0	0	0	0	0
cIMP_Recover	0	0	0	0	0	0	0	0	0	0	0	0	0	0	0	0	0	0	0	0	0	0	0	0	0	0	0	0	0	0	0	0
dAMP_Recover	0	0	0	0	0	0	0	0	0	0	0	0	0	0	0	0	0	0	0	0	0	0	0	0	0	0	0	0	0	0	0	0
F1,6P_Recover	0	0.0875	0	0.0696	0.0868	0.0502	0.0528	0	0	0.101	0	0	0	0.0425	0.0923	0	0.079	0	0	0	0	0	0	0	0.0919	0	0	0	0	0.0728	0	0
cGMP_Recover	0	0	0	0	0	0	0	0	0	0	0	0	0	0	0	0	0	0	0	0	0	0	0	0	0	0	0	0	0	0	0	0
3'-AMP_Recover	0	0.295	0	0	0	0	0	0.133	0	0	0	0	0	0	0	0	0.296	0	0	0	0	0	0	0	0	0	0	0.226	0	0	0	0.196
AMP_Recover	0.231	1.53	0.139	0.146	1.19	0.0966	0.378	0.867	0.137	0	0.123	0	1.31	0.148	1.55	0	0.595	0.107	0.183	0.223	0.299	0.25	0	0.121	0.333	0.107	0	0.598	0.25	0	0.43	0.771
IMP_Recover	0	0	0	0	0.126	0	0	0.159	0	0	0	0	0	0	0	0	0	0	0	0	0	0	0	0	0	0	0	0	0	0	0	0
Prostaglandin E2_Recover	0	0	0	0	0	0	0	0	0	0	0	0	0	0	0	0	0	0	0	0	0	0	0	0	0	0	0	0	0	0	0	0
Prostaglandin F2α _Recover	0	0	0	0	0	0	0	0	0	0	0	0	0	0	0	0	0	0	0	0	0	0	0	0	0	0	0	0	0	0	0	0
GMP_Recover	0	0.301	0	0	0	0	0	0	0	0	0	0	0.378	0	0	0	0	0	0	0	0	0	0	0	0	0	0	0	0	0	0	0.154
XMP_Recover	0	0	0	0	0	0	0	0	0	0	0	0	0	0	0	0	0	0	0	0	0	0	0	0	0	0	0	0	0	0	0	0
Orotidine 5'-monophosphate_Recover	0	0	0	0	0	0	0	0	0	0	0	0	0	0	0	0	0	0	0	0	0	0	0	0	0	0	0	0	0	0	0	0
Digalacturonate_Recover	0	0	0	0	0	0	0	0	0	0	0	0	0	0	0	0	0	0	0	0	0	0	0	0	0	0	0	0	0	0	0	0
NADPH_Recover	0	0	0	0	0	0	0	0	0	0	0	0	0	0	0	0	0	0	0	0	0	0	0	0	0	0	0	0	0	0	0	0
CoA_Recover	0	0	0	0	0	0	0	0	0	0	0	0	0	0	0	0	0	0	0	0	0	0	0	0	0	0	0	0	0	0	0	0
dCDP_Recover	0	0	0	0	0	0	0	0	0	0	0	0	0	0	0	0	0	0	0	0	0	0	0	0	0	0	0	0	0	0	0	0
dUDP_Recover	0	0	0	0	0	0	0	0	0	0	0	0	0	0	0	0	0	0	0	0	0	0	0	0	0	0	0	0	0	0	0	0
PRPP_Recover	0	0	0	0	0	0	0	0	0	0	0	0	0	0	0	0	0	0	0	0	0	0	0	0	0	0	0	0	0	0	0	0
dTDP_Recover	0	0	0	0	0	0	0	0	0	0	0	0	0	0	0	0	0	0	0	0	0	0	0	0	0	0	0	0	0	0	0	0
CDP_Recover	0	0	0	0	0	0	0	0	0	0	0	0	0	0	0	0	0	0	0	0	0	0	0	0	0	0	0	0	0	0	0	0
UDP_Recover	0	0	0	0	0	0	0	0	0	0	0	0	0	0	0	0	0	0	0	0	0	0	0	0	0	0	0	0	0	0	0	0
Acetyl CoA_Recover	0	0	0	0	0	0	0	0	0	0	0	0	0	0	0	0	0	0	0	0	0	0	0	0	0	0	0	0	0	0	0	0
Cholate_Recover	0	0	0	0	0	0	0	0	0	0	0	0	0	0	0	0	0	0	0	0	0	0	0	0	0	0	0	0	0	0	0	0
dADP_Recover	0	0	0	0	0	0	0	0	0	0	0	0	0	0	0	0	0	0	0	0	0	0	0	0	0	0	0	0	0	0	0	0
P1, P4-Di(adenosine-5') tetraphosphate_Recover	0	0	0	0	0	0	0	0	0	0	0	0	0	0	0	0	0	0	0	0	0	0	0	0	0	0	0	0	0	0	0	0
Isobutyryl CoA_Recover	0	0	0	0	0	0	0	0	0	0	0	0	0	0	0	0	0	0	0	0	0	0	0	0	0	0	0	0	0	0	0	0
Trehalose 6-phosphate_Recover	0	0	0	0	0	0	0	0.0955	0	0	0.13	0	0	0.0639	1.41	0	0	0	0	0	0	0	0	0	0	0.0672	0	0	0	0	0	0
Malonyl CoA_Recover	0	0	0	0	0	0	0	0	0	0	0	0	0	0	0	0	0	0	0	0	0	0	0	0	0	0	0	0	0	0	0	0
Adenosine 5'-phosphosulfate_Recover	0	0	0	0	0	0	0	0	0	0	0	0	0	0	0	0	0	0	0	0	0	0	0	0	0	0	0	0	0	0	0	0
Adenosine 3',5'-diphosphate_Recover	0	0	0	0	0	0	0	0	0	0	0	0	0	0	0	0	0	0	0	0	0	0	0	0	0	0	0	0	0	0	0	0
ADP_Recover	0	0	0	0	0	0	0	0	0	0	0	0	0	0	0	0	0	0	0	0	0	0	0	0	0	0	0	0	0	0	0	0
IDP_Recover	0	0	0	0	0	0	0	0	0	0	0	0	0	0	0	0	0	0	0	0	0	0	0	0	0	0	0	0	0	0	0	0
Succinyl CoA_Recover	0	0	0	0	0	0	0	0	0	0	0	0	0	0	0	0	0	0	0	0	0	0	0	0	0	0	0	0	0	0	0	0
Folate_Recover	0	0	0	0	0	0	0	0	0	0	0	0	0	0	0	0	0	0	0	0	0	0	0	0	0	0	0	0	0	0	0	0
GDP_Recover	0	0	0	0	0	0	0	0	0	0	0	0	0	0	0	0	0	0	0	0	0	0	0	0	0	0	0	0	0	0	0	0
Adenylosuccinate_Recover	0	0	0	0	0	0	0	0	0	0	0	0	0	0	0	0	0	0	0	0	0	0	0	0	0	0	0	0	0	0	0	0
Glycocholate_Recover	0	0	0	0	0	0	0	0	0	0	0	0	0	0	0	0	0	0	0	0	0	0	0	0	0	0	0	0	0	0	0	0
dCTP_Recover	0	0	0	0	0	0	0	0	0	0	0	0	0	0	0	0	0	0	0	0	0	0	0	0	0	0	0	0	0	0	0	0
dUTP_Recover	0	0	0	0	0	0	0	0	0	0	0	0	0	0	0	0	0	0	0	0	0	0	0	0	0	0	0	0	0	0	0	0
dTTP_Recover	0	0	0	0	0	0	0	0	0	0	0	0	0	0	0	0	0	0	0	0	0	0	0	0	0	0	0	0	0	0	0	0
CTP_Recover	0	0	0	0	0	0	0	0	0	0	0	0	0	0	0	0	0	0	0	0	0	0	0	0	0	0	0	0	0	0	0	0
UTP_Recover	0	0	0	0	0	0	0	0	0	0	0	0	0	0	0	0	0	0	0	0	0	0	0	0	0	0	0	0	0	0	0	0
dATP_Recover	0	0	0	0	0	0	0	0	0	0	0	0	0	0	0	0	0	0	0	0	0	0	0	0	0	0	0	0	0	0	0	0
dITP_Recover	0	0	0	0	0	0	0	0	0	0	0	0	0	0	0	0	0	0	0	0	0	0	0	0	0	0	0	0	0	0	0	0
ATP_Recover	0	0	0	0	0	0	0	0	0	0	0	0	0	0	0	0	0	0	0	0	0	0	0	0	0	0	0	0	0	0	0	0
dGTP_Recover	0	0	0	0	0	0	0	0	0	0	0	0	0	0	0	0	0	0	0	0	0	0	0	0	0	0	0	0	0	0	0	0
ITP_Recover	0	0	0	0	0	0	0	0	0	0	0	0	0	0	0	0	0	0	0	0	0	0	0	0	0	0	0	0	0	0	0	0
Taurocholate_Recover	0	0	0	0	0	0	0	0	0	0	0	0	0	0	0	0	0	0	0	0	0	0	0	0	0	0	0	0	0	0	0	0
GTP_Recover	0	0	0	0	0	0	0	0	0	0	0	0	0	0	0	0	0	0	0	0	0	0	0	0	0	0	0	0	0	0	0	0
XTP_Recover	0	0	0	0	0	0	0	0	0	0	0	0	0	0	0	0	0	0	0	0	0	0	0	0	0	0	0	0	0	0	0	0
CDP-choline_Recover	0	0	0	0	0	0	0	0	0	0	0	0	0	0	0	0	0	0	0	0	0	0	0	0	0	0	0	0	0	0	0	0
ADP-ribose_Recover	0	0	0	0	0	0	0	0	0	0	0	0	0	0	0	0	0	0	0	0	0	0	0	0	0	0	0	0	0	0	0	0
TDP-glucose_Recover	0	0	0	0	0	0	0	0	0	0	0	0	0	0	0	0	0	0	0	0	0	0	0	0	0	0	0	0	0	0	0	0
UDP-glucose_Recover	0	0.198	0.0558	0	0.156	0.0807	0	0.143	0	0	0	0	0.0676	0	0	0	0	0.0514	0	0	0.139	0.085	0	0	0.0941	0.0375	0	0	0	0	0	0.0526
UDP-glucuronate_Recover	0	0	0	0	0	0	0	0	0	0	0	0	0	0	0	0	0	0	0	0	0	0	0	0	0	0	0	0	0	0	0	0
ADP-glucose_Recover	0	0	0	0	0	0	0	0	0	0	0	0	0	0	0	0	0	0	0	0	0	0	0	0	0	0	0	0	0	0	0	0
GDP-mannose_Recover	0	0	0	0	0	0	0	0	0	0	0	0	0	0	0	0	0	0	0	0	0	0	0	0	0	0	0	0	0	0	0	0
UDP-*N*-acetylglucosamine_Recover	0	0.314	0	0	0.268	0	0	0	0	0	0	0	0	0	0	0	0	0	0	0	0	0	0	0	0	0	0	0	0	0	0	0
CMP-*N*-acetylneuraminate_Recover	0	0	0	0	0	0	0	0	0	0	0	0	0	0	0	0	0	0	0	0	0	0	0	0	0	0	0	0	0	0	0	0
NAD+_Recover	0	0	0	0	0	0	0	0	0	0	0	0	0	0	0	0	0	0	0	0	0	0	0	0	0	0	0	0	0	0	0	0
Deamido-NAD+_Recover	0	0	0	0	0	0	0	0	0	0	0	0	0	0	0	0	0	0	0	0	0	0	0	0	0	0	0	0	0	0	0	0
NADH_Recover	0	0	0	0	0	0	0	0	0	0	0	0	0	0	0	0	0	0	0	0	0	0	0	0	0	0	0	0	0	0	0	0
NADP+_Recover	0	0	0	0	0	0	0	0	0	0	0	0	0	0	0	0	0	0	0	0	0	0	0	0	0	0	0	0	0	0	0	0
FAD_Recover	0	0	0	0	0	0	0	0	0	0	0	0	0	0	0	0	0	0	0	0	0	0	0	0	0	0	0	0	0	0	0	0
Urea_Post-WM	266	269	283	451	189	285	377	225	435	299	321	871	273	438	1270	454	1970	448	730	556	536	345	891	710	187	456	417	625	533	981	233	177
Pyrazole_Post-WM	0	0	0	0	0	0	0	0	0	0	0	0	0	0	0	0	0	0	0	0	0	0	0	0	0	0	0	0	0	0	0	0
3-Aminopropionitrile_Post-WM	0	0	0	0	0	0	0	0	0	0	0	0	0	0	0	0	0	0	0	0	0	0	0	0	0	0	0	0	0	0	0	0
Methylguanidine_Post-WM	0	0	0	0	0	0	0	0	0	0	0	0	0	0	0	0	0	0	0	0	0	0	0	0	0	0	0	0	0	0	0	0
Isobutylamine_Post-WM	0	0	0	0	0	0	0	0	0	0	0	0	0	0	0	0	0	0	0	0	0	0	0	0	0	0	0	0	0	0	0	0
1,3-Diaminopropane_Post-WM	0.108	0	0.161	0.105	0.083	0.175	0.0791	0.165	0.106	0.441	0.218	0.128	0.092	0.0897	0	0.109	0.15	0.301	0.421	0.119	0.2	0.0833	0.0739	0.195	0.548	0.305	0	0.0886	0.109	0.155	0.0657	0.0757
Gly_Post-WM	5.14	11.7	18	7.7	8.4	11.2	7.08	8	11.5	28	5.79	9.01	9.07	7.04	78	8.98	16	11.7	7.6	6.09	11.6	8.92	6.7	14.3	12.7	29.2	5.46	7.97	6.05	45.8	4.87	4.59
Trimethylamine *N*-oxide_Post-WM	0.0185	0.0606	0.223	0.0336	0.0127	0.0301	0.0217	0.0296	0.0672	0	0.103	0.028	0.0306	0.0164	0.0902	0	1.06	0.0139	0.599	0.0168	0.0516	0	0.043	0	0.0322	0.214	0	0.167	0.033	0.0426	0.0341	0
Isopropanolamine_Post-WM	0.121	0.499	0.27	0.106	0.0966	0.161	0.225	0.179	0.145	0.49	0.228	0.205	0.217	0.186	0.0968	0.119	0.16	0.11	0.0858	0.104	0.0914	0.126	0.137	0.103	0.0548	0.0729	0.184	0.0748	0	0.109	0.239	0.0785
Hydroxyurea_Post-WM	0	0	0	0	0	0	0	0	0	0	0	0	0	0	0	0	0	0	0	0	0	0	0	0	0	0	0	0	0	0	0	0
Trimethylsulfonium_Post-WM	0	0	0	0	0	0	0	0	0	0	0	0	0	0	0	0	0	0	0	0	0	0	0	0	0	0	0	0	0	0	0	0
Cysteamine_Post-WM	0	0	0	0	0	0	0	0	0	0	0	0	0	0	0	0	0	0	0	0	0	0	0	0	0	0	0	0	0	0	0	0
Isoamylamine_Post-WM	0	0	0	0	0	0	0	0	0	0	0	0	0	0	0	0	0	0	0	0	0	0	0	0	0	0	0	0	0	0	0	0
Putrescine(1,4-Butanediamine)_Post-WM	7.63	13.2	23.2	4.96	5.52	13.8	14.4	10.2	13.3	36.9	2.06	9.08	7.51	7.93	15.3	13.2	3.34	5.87	6.22	3.32	11	10.2	5.76	7.8	5.06	9.21	3.87	2.31	6.26	18	5.96	1.75
β-Ala_Post-WM	0.27	0.507	0.336	0.261	0.444	0.241	0.591	0.267	0.193	1	0.596	0.252	1.08	0.451	1.43	0.205	1.08	0.446	0.588	0.282	0.249	0.21	0.198	0.322	0.405	0.428	0.423	0.268	0.425	0.257	0.154	0.302

Ala_Post-WM	2.69	7.88	4.43	6.09	4.33	6.15	4.05	5.77	4.16	13.9	4.51	5.1	6.32	6.25	14.9	4.15	7.46	7.83	6.44	4.15	4.16	4.92	2.8	7.29	6.68	9.55	5.34	3.72	5.17	6.07	3.98	3.58
Sarcosine_Post-WM	0.878	2.25	3.15	1.55	0.999	1.3	1.53	1.3	1.04	3.79	2.87	1.52	1.89	1.26	1.92	1.88	3.84	1.58	0.869	1.07	1.37	2.09	1.25	1.35	1.64	1.74	2.01	0.69	1.48	1.27	0.99	1.62
3-Aminopropane-1,2-diol_Post-WM	0	0.0411	0	0	0.0822	0	0	0	0	0	0.023	0	0	0.0243	0	0	0.035	0	0	0	0.0264	0	0	0	0	0	0	0.02	0	0	0	0
Aniline_Post-WM	0	0	0	0	0	0	0	0	0	0	0	0	0	0	0	0	0	0	0	0	0	0	0	0	0	0	0	0	0	0	0	0
4-Hydroxymethylimidazole_Post-WM	0	0	0	0	0	0	0	0	0	0	0	0	0	0	0	0	0	0	0	0	0	0	0	0	0	0	0	0	0	0	0	0
1-Methyl-2-pyrrolidinone_Post-WM	0	0	0	0	0	0	0	0	0	0	0	0	0	0	0	0	0	0	0	0	0	0	0	0	0	0	0	0	0	0	0	0
Cyclohexylamine_Post-WM	0	0	0	0	0	0	0	0	0	0	0	0	0	0	0	0	0	0	0	0	0	0	0	0	0	0	0	0	0	0	0	0
1-Aminocyclopropane-1-carboxylate_Post-WM	0	0	0	0	0	0	0	0	0	0	0	0	0	0	0	0	0	0	0	0	0	0	0	0	0	0	0	0	0	0	0	0
Betaine aldehyde_Post-WM	0	0	0	0	0	0	0	0	0	0	0	0	0	0	0	0	0	0	0	0	0	0	0	0	0	0	0	0	0	0	0	0
Spermine_Post-WM	0.0228	0.133	0.0487	0.0346	0.168	0.0614	0.0343	0.0447	0.0494	0.0479	0	0.0445	0.111	0.0985	0.0995	0.035	0.069	0.0449	0.0244	0	0.0683	0.0295	0.028	0.0507	0	0.0216	0.0205	0.162	0.0143	0.0797	0.027	0.026
Hexylamine_Post-WM	0	0	0	0	0	0	0	0	0	0	0	0	0	0	0	0	0	0	0	0	0	0	0	0	0	0	0	0	0	0	0	0
Cadaverine_Post-WM	0.884	0.772	16	0.689	3.04	5.23	2.96	4.53	3.3	7.11	1.18	1.53	2.71	1.68	1.05	4.32	2.02	1.72	4.42	0.618	1.77	2.32	0.406	2.62	3.13	2.17	0.77	0.802	3.56	5.52	0.867	1.05
GABA_Post-WM	0.251	0.352	0.39	0.294	0.205	0.484	0.262	0.376	0.236	0.659	0.396	0.229	1.81	0.185	0.335	0.531	0.482	0.41	0.265	0.261	0.29	0.354	0.278	0.42	0.36	0.441	0.212	0.0942	0.312	0.952	0.29	0.276
3-Aminoisobutyrate_Post-WM	0.0631	0.623	0.13	0.0987	0.189	0.293	0.152	0.245	0.134	0.23	0.661	0.373	0.285	0.161	0.437	0.25	0.288	0.235	0.392	0.139	0.0581	0.149	0.333	0.238	0.447	0.57	0.0936	0.247	0.163	0.148	0.264	0.117
2AB_Post-WM	0.116	0.536	0.403	0.182	0.124	0.461	0.315	0.253	0.144	0.739	0.137	0.265	0.281	0.233	0.431	0.237	0.403	0.271	0.214	0.136	0.236	0.16	0.238	0.274	0.147	0.289	0.206	0.128	0.278	0.0839	0.183	0.172
N-Methylalanine_Post-WM	0	0	0	0	0	0	0	0	0	0	0	0	0	0	0	0	0	0	0	0	0	0	0	0	0	0	0	0	0	0	0	0
N,N-Dimethylglycine_Post-WM	0.268	0.217	0	0.101	0	0.165	0.0881	0.0715	0	0.147	0.116	0.106	0.175	0.0885	0.148	0.0991	0.238	0.117	0.172	0.0695	0.0726	0.105	0.103	0.0783	0.114	0.139	0	0.113	0.102	0.145	0.0696	0.133
Choline_Post-WM	1.55	2.59	3.23	2.02	1.92	2.71	2.04	3.03	2.21	4.16	1.57	1.66	3.96	3.58	4.12	2.16	5.07	3.6	2.99	2.3	2.7	1.68	1.41	2.37	3.17	3.45	3.89	3.31	2.6	1.31	1.63	1.64
2,3-Diaminopropionate_Post-WM	0	0	0	0	0	0	0	0	0	0	0	0	0	0	0	0	0	0	0	0	0	0	0	0	0	0	0	0	0	0	0	0
Ser_Post-WM	1.31	5.04	2.57	0.898	3.4	3.96	1.59	2.29	1.28	3.96	1.88	2.04	2.67	2.93	6.74	1.87	6.06	3.63	3.18	1.47	2.38	2.28	1.6	3.57	3.84	3.8	1.88	2.71	1.85	2.71	1.67	1.69
Diethanolamine_Post-WM	0.998	0.678	1.33	2.69	1.03	0.748	2.46	1.41	2.68	2.17	1.67	1.14	1.26	1.48	1.89	0.889	2.15	1.57	1.67	1.05	1.98	1.48	0.934	0.62	1.05	1.1	1.44	2.31	1.31	3.47	1.12	0.563
N-Methylaniline_Post-WM	0	0	0	0	0	0	0	0	0	0	0	0	0	0	0	0	0	0	0	0	0	0	0	0	0	0	0	0	0	0	0	0
β-Ala-Lys_Post-WM	0	0	0	0	0	0	0	0	0	0	0.0107	0	0.0145	0.00777	0.0176	0	0.0135	0	0.0213	0	0	0	0	0	0	0	0	0	0	0	0	0
Hypotaurine_Post-WM	0.149	0.4	0.249	0	0	0.25	0.232	0.683	0	0.362	0	0.189	0.357	0.602	1.41	0.0821	0.285	0.328	0.435	0	0.301	0.259	0	0	0.283	0.49	0.198	0.349	0	0.23	0	0.48
2-Aminophenol_Post-WM	0	0	0	0	0	0	0	0	0	0	0	0	0	0	0	0	0	0	0	0	0	0	0	0	0	0	0	0	0	0	0	0
Cytosine_Post-WM	0	0.0356	0.093	0.0926	0.0495	0.0522	0.0688	0.0418	0.0467	0.0901	0.0785	0.0444	0.0516	0	0.0642	0.0591	0.0901	0.0551	0.0676	0.0433	0	0.0518	0.0214	0.0454	0.0441	0.0551	0.0784	0.0503	0.0499	0.0699	0	0.0541
Histamine_Post-WM	0.272	0.0988	0.96	0.806	0.0586	0.0317	0.0926	0.174	0.037	0.0348	0.0404	0.0356	0.0521	0.0344	0	0.0252	0.0327	0.326	0.112	0.127	0.0284	0.148	0.0322	0.0306	0.0269	0.0342	0.0378	0	0.556	0.0348	0.0481	0.0289
Uracil_Post-WM	0	0	0.706	0	0	0	0.353	0	0	0	0	0.481	0	1.07	2.44	0.639	1.44	1.44	0	0.874	1.74	0	0.536	0.393	0.779	0.9	1.58	1.59	0	0	0.828	0
Carnosine_Post-WM	0	0	0	0	0.0716	0	0.0473	0	0	0.0461	0.0313	0	0.221	0.0197	0.153	0.0168	0.0382	0	0.0273	0.0194	0.015	0.027	0.0243	0	0	0	0	0.153	0.0159	0.0194	0	0.044
Creatinine_Post-WM	0.625	1.94	1.01	0.743	0.54	0.499	0.597	0.753	0.81	1.64	1.05	0.679	1.35	1.1	2.75	0.643	2.05	0.905	1.9	0.593	1.15	0.594	1.16	1.19	1.22	1.25	0.715	0.808	1.32	1.01	0.474	0.649
Muscimol_Post-WM	0	0	0	0	0	0	0	0	0	0	0	0	0	0	0	0	0	0	0	0	0	0	0	0	0	0	0	0	0	0	0	0
β-Cyanoalanine_Post-WM	0	0	0	0	0	0	0	0	0	0	0	0	0	0	0	0	0	0	0	0	0	0	0	0	0	0	0	0	0	0	0	0
Dihydrouracil_Post-WM	0	0	0	0	0	0	0	0	0	0	0	0	0	0	0	0	0	0	0	0	0	0	0	0	0	0	0	0	0	0	0	0
Pro_Post-WM	4.98	3.92	25.1	5.17	5.51	9.53	6.39	5.59	4.69	30.4	4.66	16.6	6.72	5.49	45.2	6.69	11.1	6.64	5.75	4.76	8.49	5.52	2.51	9.81	4.12	11.9	1.94	5.08	3.5	66.6	2.47	4.48
Guanidinoacetate_Post-WM	0.152	0.642	0	0.312	0.298	0	0	0	0	0	0.32	0	0.556	0.674	0.54	0.637	0	0.736	0.7	0.24	0	0.476	0.309	0	0.489	0.46	0.162	0.123	0	0.347	0.171	0.185
5-Aminovalerate_Post-WM	45.9	90.6	151	35.4	15.3	121	70	73	84.7	404	30.3	84.7	80.1	74.7	121	77.2	77	55.1	84.5	46.6	90	54.6	41.3	94.1	40.9	84.3	18.9	7.37	54.2	83.9	39.6	10.6
Val_Post-WM	0.95	2.2	4.21	1.12	1.87	4.02	1.89	1.88	0.878	6.8	1.28	0.788	2.39	1.58	4.58	1.88	3.07	2.22	1.83	0.913	1.94	0.89	3.91	1.97	1.52	1.76	1.65	1.16	2.06	0.509	2.41	1.27
Betaine_Post-WM	0.85	1.99	1.74	1.12	0.533	0.583	0.49	0.708	1.02	2.16	1.19	0.988	0.669	0.627	0.895	0.539	1.09	0.625	2.8	1.02	0.677	0.535	1.31	1.05	0.889	1.6	0.313	0.777	0.613	1.81	0.383	0.96
Benzimidazole_Post-WM	0	0	0	0	0	0	0	0	0	0	0	0	0	0	0	0	0	0	0	0	0	0	0	0	0	0	0	0	0	0	0	0
2,4-Diaminobutyrate_Post-WM	0	0	0	0	0	0	0	0	0	0	0	0	0	0	0	0	0	0	0	0	0	0	0	0	0	0	0	0	0	0	0	0
4-Amino-3-hydroxybutyrate_Post-WM	0	0	0	0	0	0	0	0	0	0	0	0	0	0	0	0	0	0	0	0	0	0	0	0	0	0	0	0	0	0	0	0
Homoserine_Post-WM	0	0	0.066	0	0	0.0922	0.0555	0	0.0424	0.14	0.052	0.0736	0.0856	0	0.0832	0	0	0	0.097	0	0.0558	0.0555	0.0541	0.0475	0	0.0735	0	0	0.0483	0.103	0.0759	0.0486
α-Methylserine_Post-WM	0	0	0	0	0	0	0	0	0	0	0	0	0	0	0	0	0	0	0	0	0	0	0	0	0	0	0	0	0	0	0	0
Thr_Post-WM	0.487	2.19	1.61	0.583	1.3	1.87	0.978	1.07	0.725	1.54	0.876	0.776	1.18	0.961	3.78	0.606	1.87	1.22	1.14	0.54	0.965	0.876	0.532	1	1.11	1.2	1.07	0.847	0.791	0.101	0.867	1.11
Purine_Post-WM	0	0	0	0	0	0	0	0	0	0	0	0	0	0	0	0	0	0	0	0	0	0	0	0	0	0	0	0	0	0	0	0
Anserine_Post-WM	0.0468	0	0.041	0	0.0378	0.00692	0.175	0.00528	0.012	0.022	0	0.0235	0.823	0.036	0.058	0	0.0994	0.0212	0.0767	0.0833	0	0.0156	0	0.0404	0	0	0	0.227	0.0723	0.0295	0	0
Homocarnosine_Post-WM	0	0	0	0	0	0	0	0	0	0	0	0	0	0	0.0271	0	0.015	0	0.0229	0	0	0	0	0	0	0	0	0	0	0	0	0
Benzamidine_Post-WM	0	0	0	0	0	0	0	0	0	0	0	0	0	0	0	0	0	0	0	0	0	0	0	0	0	0	0	0	0	0	0	0
Cys_Post-WM	0	0	0	0	0	0	0	0	0	0	0	0	0	0	0	0	0	0	0	0	0	0	0	0	0	0	0	0	0	0	0	0
Benzamide_Post-WM	2.76	6.14	8.01	3.46	1.43	2.15	5.82	2.93	4.43	12	2.24	5.03	5.56	3.67	1.97	3.74	5.33	3.51	2.11	2.73	2.83	3.96	3.78	3.6	3.49	3.77	4.33	1.22	3.15	2.85	3.98	1.15
Phenethylamine_Post-WM	0	0	0	0	0	0	0	0	0	0	0	0	0	0	0	0	0	0	0	0	0	0	0	0	0	0	0	0	0	0	0	0
α-Methylbenzylamine_Post-WM	0	0	0	0	0	0	0	0	0	0	0	0	0	0	0	0	0	0	0	0	0	0	0	0	0	0	0	0	0	0	0	0
2,4-Dimethylaniline_Post-WM	0	0	0	0	0	0	0	0	0	0	0	0	0	0	0	0	0	0	0	0	0	0	0	0	0	0	0	0	0	0	0	0
Isonicotinamide_Post-WM	0	0	0	0	0	0	0	0	0	0	0	0	0	0	0	0	0	0	0	0	0	0	0	0	0	0	0	0	0	0	0	0
Nicotinamide_Post-WM	0.0403	0.0892	0.133	0.086	0.124	0.104	0.0906	0.0923	0.143	0.319	0.108	0.104	0.0825	0.0946	0.151	0.0761	0.0381	0.081	0.162	0.0446	0.144	0.0759	0.0546	0.0875	0.0848	0.0807	0	0.123	0.0933	0.0782	0.0328	0.12
Picolinamide_Post-WM	0	0	0	0	0	0	0	0	0	0	0	0	0	0	0	0	0	0	0	0	0	0	0	0	0	0	0	0	0	0	0	0
3-Chloroalanine_Post-WM	0	0	0	0	0	0	0	0	0	0	0	0	0	0	0	0	0	0	0	0	0	0	0	0	0	0	0	0	0	0	0	0
Taurine_Post-WM	22.5	79.8	40.9	30.4	46.5	21.7	32.6	54.6	30.1	47.5	45	23.1	67.9	48.3	50.5	22.4	52.6	41.4	43.5	21.2	41	35.2	24.7	34	53.4	35.6	25.4	30	31.1	22	28	35
5-Methylcytosine_Post-WM	0	0	0	0	0	0	0	0	0	0	0	0	0	0	0	0	0	0	0	0	0	0	0	0	0	0	0	0	0	0	0	0
1-Methylhistamine_Post-WM	0	0	0.0215	0.00836	0	0	0	0	0	0	0	0	0	0	0	0	0	0	0	0.00969	0	0.00929	0	0	0	0	0	0	0.013	0	0	0
Thymine_Post-WM	0.985	9.12	1.21	0.289	2.43	2.48	1.13	4.72	2.31	1.74	2.12	2.1	3.2	3.52	4.79	1.01	5.99	2.21	2.2	1.94	4.41	1.98	0.963	1.69	2.26	1.83	1	2.37	1.15	2.69	1.87	1.89
Imidazole-4-acetate_Post-WM	0	0	0	0	0	0	0	0	0	0	0	0	0	0	0	0	0	0	0	0	0	0	0	0	0	0	0	0	0	0	0	0
Melamine_Post-WM	0	0	0	0	0	0	0	0	0	0	0	0	0	0	0	0	0.0343	0	0	0	0	0	0	0	0	0	0	0	0	0	0	0
1-Amino-1-cyclopentanecarboxylate_Post-WM	0	0	0	0	0	0	0	0	0	0	0	0	0	0	0	0	0	0	0	0	0	0	0	0.109	0	0	0	0	0	0	0	0
Pipecolate_Post-WM	0.0759	0.0686	0.186	0.155	0.0638	0.0937	0.0733	0.0556	0.106	0.212	0.294	0.0413	0.0848	0.0432	0.0987	0.053	0.45	0.0615	0.231	0.0525	0	0.0571	0.04	0.0888	0.156	0.167	0.0526	0	0.0615	0.0697	0.0894	0.0616
Metformin_Post-WM	0	0	0	0	0	0	0	0	0	0	0	0	0	0	0	0	0	0	0	0	0	0	0	0	0	0	0	0	0	0	0	0
Octylamine_Post-WM	0	0	0	0	0	0	0	0	0	0	0	0	0	0	0	0	0	0	0	0	0	0	0	0	0	0	0	0	0	0	0	0
N-Acetylputrescine_Post-WM	0.308	0.579	0.705	0.192	0.224	0.233	0.515	0.403	0.338	1.84	0.303	0.438	0.442	0.35	0.274	0.332	0.615	0.238	0.539	0.254	0.472	0.493	0.387	0.268	0.364	0.674	0.173	0.0966	0.363	0.706	0.252	0.0926
Agmatine_Post-WM	0.0255	0.089	0.0652	0.038	0.0489	0.05	0.0452	0.0312	0.0542	0.0835	0.0418	0.0343	0.0285	0.0569	0	0.0366	0.0392	0.0345	0.0463	0.0246	0.0455	0.0377	0	0.0288	0.0348	0.0278	0.031	0	0.0194	0.0633	0	0.0353
Hydroxyproline_Post-WM	0	0.252	0.304	0.179	0.2	0.174	0.206	0.13	0.118	0.277	0.325	0.146	0.187	0.206	1.24	0.179	0.713	0.272	0.128	0.119	0.117	0.18	0	0.15	0.156	0.207	0.173	0.174	0.113	0.148	0.103	0.248
5-Aminolevulinate_Post-WM	0	0	0.0311	0	0	0	0	0	0	0.044	0.0296	0	0.0343	0	0.0306	0	0	0	0	0	0	0.0285	0	0	0	0.0221	0	0	0.0136	0.0261	0	0
Creatine_Post-WM	2.5	9.53	5.23	3.83	2.96	4.3	2.3	4.6	3.31	6.28	4.43	3.47	3.98	5.3	15	1.81	7.57	4.46	4.46	2.84	3.29	2.99	2.34	3.29	5.08	5.96	3.59	5.32	2.77	3.82	2.13	4.96
6-Aminohexanoate_Post-WM	0	0.252	0	0	0	0	0	0	0	0	0	0.161	0	0	0	0	0	0	0	0	0	0	0	0	0	0	0	0	0	0	0	0
β-Leucine_Post-WM	0	0	0	0	0	0	0	0	0	0	0	0	0	0	0	0	0	0	0	0	0	0	0	0	0	0	0	0	0	0	0	0
Ile_Post-WM	0.212	0.689	1	0.174	0.511	0.939	0.418	0.577	0.142	1.35	0.256	0.119	1.22	0.373	1.42	0.381	1.37	0.53	0.589	0.251	0.493	0.259	1.19	0.528	0.441	0.442	0.452	0.446	0.539	0.129	0.663	0.343
Leu_Post-WM	0.73	2.13	3.92	0.689	1.75	2.79	1.72	1.71	0.664	3.9	0.722	0.611	2.52	1.18	4.04	1.59	3.56	1.68	1.75	0.846	1.49	0.752	3.3	1.54	1.19	1.41	1.14	1.65	1.69	0.83	1.75	0.891
Bis(3-aminopropyl)amine_Post-WM	0	0	0	0	0	0	0	0	0	0	0	0	0	0	0	0	0	0	0	0	0	0	0	0	0	0	0	0	0	0	0	0
Gly-Gly_Post-WM	0.0245	0	0.176	0.0659	0	0.105	0.103	0.0176	0.0731	0.263	0.0874	0	0.0445	0.0756	0.101	0.102	0.0889	0.145	0.112	0.079	0.113	0	0.0581	0.0862	0.127	0.155	0	0	0.0629	0.114	0.0272	0.0233
Asn_Post-WM	0.149	0.539	0	0.201	0.717	0.0264	0	0.0704	0	0	0	0	0.289	0	2.34	0.0533	0.264	0	0	0.105	0.0722	0.227	0.307	0	0	0	0	0.886	0.059	0.138	0.0991	0.217
Ornithine_Post-WM	1.02	2.35	3.87	1.01	2.27	1.72	1.9	1.24	1.57	8.58	1.46	3.58	0.922	2	2.88	2.51	1.71	2.95	2.1	0.748	1.92	1.31	1.14	2.56	1.74	4.18	0.429	1.14	1.98	6.28	0.587	0.556
Asp_Post-WM	1.23	2.27	2.06	1.17	1.84	2.82	1.7	1.76	1.66	4.54	3.04	2.66	1.86	2.68	12.4	3.35	3.9	4.19	4.05	2.36	2.7	2.09	1.59	4.35	3.65	5.47	2.18	2.02	3.63	3.48	1.32	1.16
2-Aminobenzimidazole_Post-WM	0	0	0	0	0	0	0	0	0	0	0	0	0	0	0	0	0	0	0	0	0	0	0	0	0	0	0	0	0	0	0	0
Homocysteine_Post-WM	0	0	0	0	0	0	0	0	0	0	0	0	0	0	0	0	0	0	0	0	0	0	0	0	0	0	0	0	0	0	0	0
Adenine_Post-WM	0.111	0.255	0.371	0.145	0.291	0.259	0.166	0.262	0.174	0.269	0.319	0.127	0.143	0.298	0.19	0.195	0.515	0.296	0.273	0.124	0.227	0.155	0.203	0.355	0.294	0.31	0.208	0.202	0.23	0.129	0.168	0.158
Hypoxanthine_Post-WM	0.734	2.51	1.52	0.902	0.952	1.32	0.749	2.52	0.937	1.92	0.596	0.764	1.87	2.51	2.76	0.888	3.58	2.74	2.22	1.47	1.55	1.39	0.91	2.01	2.7	2.23	1.7	2.27	1.99	0.399	1.49	1.31
1-Methylnicotinamide_Post-WM	0	0.0556	0.028	0	0	0.00748	0	0	0.0267	0.0293	0.0165	0	0.0314	0.0126	0.0444	0.0134	0.152	0	0.0441	0	0.0174	0	0.0257	0.0145	0	0.0261	0.0228	0	0.0157	0	0.0122	0
p-Aminobenzoate_Post-WM	0	0	0	0	0	0	0	0	0	0	0	0	0	0	0	0	0	0	0	0	0	0	0	0	0	0	0	0	0	0	0	0
Trigonelline_Post-WM	0.817	0.1	0.0615	0	0.115	0	0.0383	0.0336	0	0.0242	0	0.0923	0.0713	0	0.331	0	0	0	0.0325	0	0.0667	0.224	0	0	0	0.354	0	0	0	0.0293	0	0
Anthranilate_Post-WM	0	0	0	0	0	0	0	0	0	0	0	0	0	0	0	0	0	0	0	0	0	0	0	0	0	0	0	0	0	0	0	0
Phenylethanolamine_Post-WM	0	0	0	0	0	0	0	0	0	0	0	0	0	0	0	0	0	0	0	0	0	0	0	0	0	0	0	0	0	0	0	0
Tyramine_Post-WM	0	0.0827	0	0	0.0315	0	0	0	0	0	0.0404	0	0	0	0	0	0	0.0573	0	0	0	0	0	0	0	0	0	0	0.062	0	0	0
Urocanate_Post-WM	0.2	0.737	0.359	0.145	0.987	0.489	0.236	0.116	0.0934	0.344	0.308	0.258	0.242	0.2	0.507	0.223	0.391	0.443	0.435	0.119	0.345	0.339	0.141	0.478	0.484	0.527	0.109	0.0882	0.128	0.454	0.231	0.0962

6-Hydroxynicotinate_Post-WM	0	0	0	0	0	0	0	0	0	0	0	0	0	0	0	0	0	0	0	0	0	0	0	0	0	0	0	0	0	0	0	0
Tropinone_Post-WM	0	0	0	0	0	0	0	0	0	0	0	0	0	0	0	0	0	0	0	0	0	0	0	0	0	0	0	0	0	0	0	0
Histidinol_Post-WM	0	0	0	0	0	0	0	0	0	0	0.00602	0.0112	0	0	0	0	0	0	0.0206	0	0	0	0	0	0.0119	0	0	0	0	0.00715	0	0
Tropine_Post-WM	0	0	0	0	0	0	0	0	0	0	0	0	0	0	0	0	0	0	0	0	0	0	0	0	0	0	0	0	0	0	0	0
Ectoine_Post-WM	0	0	0	0	0	0	0	0	0	0	0	0	0	0	0	0	0	0	0	0	0	0	0	0	0	0	0	0	0	0	0	0
4-Methyl-5-thiazoleethanol_Post-WM	0	0	0	0	0	0	0	0	0	0	0	0	0	0	0	0	0	0	0	0	0	0	0	0	0	0	0	0	0	0	0	0
Proline betaine_Post-WM	0.105	0	0.153	0.0477	0.0153	0.0698	0	0	0.0324	0.0791	0.0265	0.012	0	0	0.203	0.0586	0.0322	0	0.066	0	0	0	0.0207	0	0.0821	0.0227	0.0266	0	0	0.0291	0.0241	0
N^1,N^{12}-Diacetylspermine_Post-WM	0.00924	0.0322	0.0279	0	0.0233	0.0115	0.0199	0.0226	0.0268	0.0439	0.0232	0.0185	0.0272	0.0263	0	0.0174	0.0308	0.0253	0.0129	0.0159	0.0352	0.0253	0.00967	0.0157	0.0238	0.0172	0.0192	0	0.0137	0.0247	0	0
γ-Guanidinobutyrate_Post-WM	0	0	0	0	0	0	0	0	0	0	0	0	0.0201	0	0	0	0	0	0	0	0	0	0	0	0.037	0.0155	0	0	0	0	0.018	0
Acetylcholine_Post-WM	0	0	0	0	0	0	0	0	0	0	0	0	0	0	0	0	0	0	0	0	0	0	0	0	0	0	0	0	0	0	0	0
γ-Butyrobetaine_Post-WM	0.359	1.15	0.927	0.176	0.107	0.595	0.504	0.383	0.76	1.85	0.649	0.536	0.625	0.531	0.311	0.573	0.979	0.754	0.789	0.329	0.407	0.323	0.367	0.301	0.74	0.765	0.416	0.0688	0.493	0.233	0.308	0.102
Lysinamide_Post-WM	0	0	0	0	0	0	0	0	0	0	0	0	0	0	0	0	0	0	0	0	0	0	0	0	0	0	0	0	0	0	0	0
Spermidine_Post-WM	0.101	0.502	0.434	0.126	0.217	0.409	0.346	0.264	0.44	0.902	0.282	0.285	0.104	0.185	0.226	0.288	0.21	0.455	0.321	0.0969	0.348	0.224	0.149	0.229	0.239	0.356	0.271	0.339	0.206	0.272	0.109	0.0809
Gln_Post-WM	1.64	7.93	7.48	2.7	4.51	5.1	2.25	4.29	2.51	7.1	2.74	2.6	4.44	3.91	13.4	2.5	8.38	4.7	3.83	1.95	3.08	3.14	2.09	4.11	5.34	6.7	2.85	4.49	2.63	2.72	2.5	4.14
5,6-Dimethylbenzimidazole_Post-WM	0	0	0	0	0	0	0	0	0	0	0	0	0	0	0	0	0	0	0	0	0	0	0	0	0	0	0	0	0	0	0	0
Lys_Post-WM	1.71	3.73	3.32	0.646	4.29	1.64	2.84	1.86	3.5	6.59	1.63	2.16	2.57	3.34	5.63	3.21	5.9	4.15	4.14	1.51	4.22	2.82	1.68	2.78	3.61	6.44	1.61	2.86	3.62	11.1	1.04	1.32
Trientine_Post-WM	0	0	0	0	0	0	0	0	0	0	0	0	0	0	0	0	0	0	0	0	0	0	0	0	0	0	0	0	0	0	0	0
Glu_Post-WM	3.07	8.64	11.9	4.66	5.12	6.88	5.75	5.25	4.61	12.8	6.21	7.29	5.95	8.22	31.4	4.83	10.2	8.42	7.25	4.15	7.04	5.37	3.94	6.26	6.46	11.5	6.15	4.99	5.41	10.1	3.83	3.1
O-Acetylserine_Post-WM	0	0	0.0807	0.0664	0.113	0.0523	0.0732	0	0.0492	0.117	0.0624	0	0.109	0	0	0	0	0	0	0	0	0	0.0547	0	0	0	0	0	0.0343	0.161	0	0.0526
Nornicotine_Post-WM	0	0	0	0	0	0	0	0	0	0	0	0	0	0	0	0	0	0	0	0	0	0	0	0	0	0	0	0	0	0	0	0
Met_Post-WM	0	0.397	0.0779	0	0.0296	0.169	0	0.34	0	0.157	0.151	0	0.306	0.222	0.552	0.0371	0.422	0.0883	0.589	0.0697	0.115	0.0683	0.0411	0.0774	0.0965	0.177	0.147	0.171	0.171	0	0.152	0.203
3-Methyladenine_Post-WM	0	0	0	0	0	0	0	0	0	0	0	0	0	0.0073	0	0	0	0	0	0	0	0	0	0	0	0	0	0	0	0	0	0
6-Methylaminopurine_Post-WM	0	0	0	0	0	0	0	0	0	0	0	0	0	0	0	0	0	0	0	0	0	0	0	0	0	0	0	0	0	0	0	0
Guanine_Post-WM	0.279	0.901	0.548	0.362	0.457	0.546	0.216	0.645	0.279	0.27	0.254	0.267	0.585	0.919	0.951	0.26	0.608	0.597	0.196	0.403	0.369	0.425	0.354	0.475	0.735	0.261	0.752	0.921	0.365	0.252	0.397	0.503
Amantadine_Post-WM	0	0	0	0	0	0	0	0	0	0	0	0	0	0	0	0	0	0	0	0	0	0	0	0	0	0	0	0	0	0	0	0
Xanthine_Post-WM	0.23	1.21	0.752	0.695	1.06	0.773	0.379	1.79	1.29	0.495	0.867	0	0.462	1.66	3.76	0.434	3.67	2.69	1.35	1.72	0.299	0.859	1.32	1.55	1.63	1.8	1.86	1.92	1.32	0	1.14	0
4-Aminosalicylate_Post-WM	0	0	0	0	0	0	0	0	0	0	0	0	0	0	0	0	0	0	0	0	0	0	0	0	0	0	0	0	0	0	0	0
3-Hydroxyanthranilate_Post-WM	0	0	0	0	0	0	0	0	0	0	0	0	0	0	0	0	0	0	0	0	0	0	0	0	0	0	0	0	0	0	0	0
Dopamine_Post-WM	0	0	0	0	0	0	0	0	0	0	0	0	0	0	0	0	0	0	0	0	0	0	0	0	0	0	0	0	0	0	0	0
4-(β-Acetylaminoethyl)imidazole_Post-WM	0.0133	0.0303	0.0335	0.0175	0	0	0	0.00729	0	0.0191	0	0	0	0	0	0	0.0213	0.014	0.0104	0	0	0	0	0	0	0.0133	0	0	0.0264	0	0	0
Pseudopelletierine_Post-WM	0	0	0	0	0	0	0	0	0	0	0	0	0	0	0	0	0	0	0	0	0	0	0	0	0	0	0	0	0	0	0	0
His_Post-WM	1.58	3.34	1.5	1.34	3.15	1.53	2.35	1.51	1.72	2.31	1.63	1.93	1.44	3.15	2.99	2.47	5.09	2.32	2.72	1	2.36	1.3	1.43	2.1	2.42	3.01	0.968	1.52	1.62	2.95	0.517	1.21
β-Imidazolelactate_Post-WM	0	0	0	0.088	0	0	0	0	0	0.0858	0	0	0.0913	0	0	0	0	0.0793	0	0	0	0	0.0493	0.0576	0	0	0	0	0	0	0	0
Ibotenate_Post-WM	0	0	0	0	0	0	0	0	0	0	0	0	0	0	0	0	0	0	0	0	0	0	0	0	0	0	0	0	0	0	0	0
Allantoin_Post-WM	9.69	17.7	14.1	14.2	17	15	14.6	17.3	19.1	17.7	15.1	12.8	14.8	21.3	23.9	10.5	22.7	12.2	14	10.8	21.3	13.3	10.8	11.6	15.1	11	15.8	11.3	10.6	14.3	10.3	15.1
Indole-3-acetaldehyde_Post-WM	0	15.3	0	4.3	20.8	15.3	0	18.9	16.7	0	3.11	0	16.4	0	0	4.38	0	0	0	2.53	0	12.8	10	0	0	0	0	0	0	9.36	0	15.2
Betonicine_Post-WM	0	0	0	0	0	0	0	0	0	0	0	0	0	0	0	0	0	0	0	0	0	0	0	0	0	0	0	0	0	0	0	0
N-Acetylvaline_Post-WM	0	0	0	0	0	0	0	0	0	0	0	0	0	0	0	0	0	0	0	0	0	0	0	0	0	0	0	0	0	0	0	0
Ala-Ala_Post-WM	0.0938	0.127	0.172	0	0.128	0.07	0.0428	0.122	0.109	0.336	0.201	0.117	0	0.13	0	0.0665	0.146	0.119	0.184	0.058	0.137	0.103	0.0738	0.0845	0	0.158	0	0	0.15	0.133	0	0.0685
Tryptamine_Post-WM	0	0	0	0	0	0	0	0	0	0	0	0	0	0	0	0	0	0	0	0	0	0	0	0	0	0	0	0	0	0	0	0
α-Aminoadipate_Post-WM	0.174	0.612	0.395	0.208	0.349	0.338	0.334	0.359	0.166	0.718	0.612	0.352	0.315	0.426	0.901	0.252	0.837	0.371	0.381	0.328	0.374	0.428	0.142	0.208	0.451	0.373	0.311	0.501	0.161	0.526	0.225	0.607
N-Methylglutamate_Post-WM	0	0	0	0	0	0	0	0	0	0	0	0	0	0	0	0	0	0	0	0	0	0	0	0	0	0	0	0	0	0	0	0
Indole-3-ethanol_Post-WM	0	0	0	0	0	0	0	0	0	0	0	0	0	0	0	0	0	0	0	0	0	0	0	0	0	0	0	0	0	0	0	0
Carnitine_Post-WM	0.251	0.625	0.406	0.313	0.254	0.361	0.197	0.355	0.427	0.763	0.297	0.499	0.338	0.511	0.725	0.293	0.796	0.616	0.642	0.333	0.374	0.279	0.347	0.449	0.636	0.64	0.264	0.42	0.348	0.395	0.178	0.18
2-Deoxystreptamine_Post-WM	0	0	0	0	0	0	0	0	0	0	0	0	0	0	0	0	0	0	0	0	0	0	0	0	0	0	0	0	0	0	0	0
5-Hydroxylysine_Post-WM	0	0	0	0	0	0	0	0	0	0	0	0	0	0	0	0	0	0	0	0	0	0	0	0	0	0	0	0	0	0	0	0
Nicotine_Post-WM	0	0	0	0	0	0	0	0	0	0	0	0	0	0	0	0	0	0	0	0	0	0	0	0	0	0	0	0	0	0	0	0
Pterin_Post-WM	0	0	0	0	0	0	0	0	0	0	0	0	0	0	0	0	0	0	0	0	0	0	0	0	0	0	0	0	0	0	0	0
S-Methylmethionine_Post-WM	0	0	0	0	0	0	0	0	0	0	0	0	0	0	0	0	0	0	0	0	0	0	0	0	0	0	0	0	0	0	0	0
Methionine sulfoxide_Post-WM	0	0	0.0617	0	0	0	0	0.0799	0	0.062	0	0	0.0779	0.0409	0.112	0.0535	0	0	0.0871	0	0	0	0	0	0	0.0727	0	0.0849	0.0773	0	0	0.054
7-Methylguanine_Post-WM	0.0258	0.06	0.042	0	0	0	0	0	0.0274	0.0536	0.039	0.0274	0.0451	0.0402	0.0292	0.0196	0.0514	0	0.0431	0.0211	0	0.0312	0.0252	0.0223	0.0333	0.0297	0	0.0303	0.0218	0.018	0.0258	0
3-Methylguanine_Post-WM	0	0	0	0	0	0	0	0	0	0	0	0	0	0	0	0	0	0	0	0	0	0	0	0	0	0	0	0	0	0	0	0
Phe_Post-WM	2.24	2.4	3.72	2.47	4.56	3.46	2.66	1.95	2.12	5.05	2.11	2.34	2.55	2.98	6.69	2.89	4.46	2.39	2.56	1.18	3.77	2.11	2.83	3.25	1.29	3.09	1.19	2.1	3.07	1.84	1.98	1.69
Taurocyamine_Post-WM	0	0	0	0	0	0	0	0	0	0	0	0	0	0	0	0	0	0	0	0	0	0	0	0	0	0	0	0	0	0	0	0
Pyridoxal_Post-WM	0	0	0	0	0	0	0	0	0	0	0	0	0	0	0	0	0	0	0	0	0	0	0	0	0	0	0	0	0	0	0	0
3-Methoxytyramine_Post-WM	0	0	0	0	0	0	0	0	0	0	0	0	0	0	0	0	0	0	0	0	0	0	0	0	0	0	0	0	0	0	0	0
Synephrine_Post-WM	0	0	0	0	0	0	0	0	0	0	0	0	0	0	0	0	0	0	0	0	0	0	0	0	0	0	0	0	0	0	0	0
Pyridoxamine_Post-WM	0	0	0	0	0	0	0	0	0	0	0	0	0	0	0	0	0	0	0	0	0	0	0	0	0	0	0	0	0	0	0	0
Pyridoxine_Post-WM	0	0	0	0	0	0	0	0	0	0	0	0	0	0	0	0	0	0	0	0	0	0	0	0	0	0	0	0	0	0	0	0
Noradrenaline_Post-WM	0	0	0	0	0	0	0	0	0	0	0	0	0	0	0	0	0	0	0	0	0	0	0	0	0	0	0	0	0	0	0	0
3-Methylhistidine_Post-WM	0.047	0.0735	0.133	0.0185	0.0517	0.05	0.14	0.0481	0.0507	0.0983	0.0586	0.0799	0.211	0.0473	0.303	0.0419	0.341	0.0786	0.199	0.0826	0.0344	0.039	0.0199	0.0345	0.0621	0.0408	0.0514	0.0493	0.0891	0.0545	0.0237	0.0274
Indole-3-acetamide_Post-WM	0.387	0	1.7	1.26	0	0	0	0	0	0	0	0	0	0	0	1.15	0	0	0	0	0	0	0.358	0	0	1.27	0	0	0	0.576	0	0
N-Acetylornithine_Post-WM	0	0	0.0478	0	0	0.0513	0.0458	0	0.0184	0.0909	0.0299	0.0302	0.0477	0	0	0	0.0384	0.0445	0	0.0235	0	0	0	0	0	0.0315	0	0	0.0326	0.0343	0.0243	0
N-γ-Ethylglutamine_Post-WM	0	0.0762	0	0	0.0836	0.0567	0	0.034	0.0343	0	0.0395	0	0	0	0.0753	0	0.0771	0	0.0418	0	0.0471	0	0	0	0	0	0	0	0	0	0	0.0362
Arg_Post-WM	2.12	7.43	2.93	1.18	2.73	2.59	2.94	2.36	2.63	9.5	2.84	6.27	2.99	5.91	6.26	3.11	9.75	5.25	7.34	2.25	5.15	2.22	3.89	6.47	4.92	7.24	2.4	4.9	4.24	8.06	1.89	2.32
Gramine_Post-WM	0	0	0	0	0	0	0	0	0	0	0	0	0	0	0	0	0	0	0	0	0	0	0	0	0	0	0	0	0	0	0	0
N-ω-Methyltryptamine_Post-WM	0	0	0	0	0	0	0	0	0	0	0	0	0	0	0	0	0	0	0	0	0	0	0	0	0	0	0	0	0	0	0	0
Guanidinosuccinate_Post-WM	0	0	0	0	0	0	0	0	0	0	0	0	0	0	0	0	0	0	0	0	0	0	0	0	0	0	0	0	0	0	0	0
Indole-3-acetate_Post-WM	0.536	1.82	0.603	0	0	0.561	2.31	1.06	1.84	0	1.13	0.691	0.801	1.21	0.868	0.66	1.08	1.03	1.22	0.587	1.6	0.996	0.638	0	0.667	1.8	0.638	0	0.762	0	1.69	0
2-Guanidinobenzimidazole_Post-WM	0	0	0	0	0	0	0	0	0	0	0	0	0	0	0	0	0	0	0	0	0	0	0	0	0	0	0	0	0	0	0	0
Citrulline_Post-WM	0.494	1.51	1.07	0.56	0.957	0.798	0.842	1.27	0.943	2.81	1.06	1.2	1.33	1.18	3.38	0.729	3.88	2.7	1.85	0.625	1.19	0.804	0.73	1.75	2.05	3.76	0.371	0.621	1.3	1.85	0.841	0.552
Canavanine_Post-WM	0	0	0	0	0	0	0	0	0	0	0	0	0	0	0	0	0	0	0	0	0	0	0	0	0	0	0	0	0	0	0	0
Serotonin_Post-WM	0	0	0	0	0	0	0	0	0	0	0	0	0	0	0	0	0	0	0	0	0	0	0	0	0	0	0	0	0	0	0	0
Alliin_Post-WM	0	0	0	0	0	0	0	0	0	0	0	0	0	0	0.317	0	0	0	0	0	0	0	0	0	0	0	0	0	0	0	0	0
Cys-Gly_Post-WM	0	0	0	0	0	0	0	0	0	0	0	0	0	0	0	0	0	0	0	0	0	0	0	0	0	0	0	0	0	0	0	0
Xanthopterin_Post-WM	0	0	0	0	0	0	0	0	0	0	0	0	0	0	0	0	0	0	0	0	0	0	0	0	0	0	0	0	0	0	0	0
Mannosamine_Post-WM	0	0	0.0261	0	0	0	0	0.0724	0	0.0552	0	0	0.032	0	0	0	0.171	0	0	0	0	0	0	0.0492	0	0.028	0	0	0	0	0	0
Glucosamine_Post-WM	0	0.054	0.0478	0.0334	0	0.0308	0	0.242	0	0.194	0.0477	0	0.146	0.0572	0	0.0528	0.478	0	0.0989	0.048	0	0.0395	0.0386	0.0866	0.12	0.0779	0	0	0	0	0	0
n-Butyl a-Picolinate_Post-WM	0	0	0	0	0	0	0	0	0	0	0	0	0	0	0	0	0	0	0	0	0	0	0	0	0	0	0	0	0	0	0	0
Methionine sulfoximine_Post-WM	0	0	0	0	0	0	0	0	0	0	0	0	0	0	0	0	0	0	0	0	0	0	0	0	0	0	0	0	0	0	0	0
Tyr_Post-WM	2.53	3.98	4.13	2.24	6.71	2.52	4.11	2.33	4.13	4.91	2.63	3.88	3.26	3.79	7.39	4.1	6.56	3.91	3.62	1.94	3.81	3.85	3.41	3.4	2.53	5.49	1.9	3.51	3.63	5.34	1.83	1.85
Harman_Post-WM	0	0	0	0	0	0	0	0	0	0	0	0	0	0	0	0	0	0	0	0	0	0	0	0	0	0	0	0	0	0	0	0
Phosphorylcholine_Post-WM	1.88	6.13	2.87	1.24	2.59	1.83	1.84	1.62	1.19	4.86	3.19	2.21	1.55	2.39	4.2	1.85	4.52	2.03	2.9	1.48	1.62	2.45	2.66	2.23	2.18	2	1.87	1.86	1.11	2.38	1.79	1.99
Epinephrine_Post-WM	0	0	0	0	0	0	0	0	0	0	0	0	0	0	0	0	0	0	0	0	0	0	0	0	0	0	0	0	0	0	0	0
N-α,N-α-Dimethylhistidine_Post-WM	0	0	0	0	0	0	0	0	0	0	0	0	0	0	0	0	0	0	0	0	0	0	0	0	0	0	0	0	0	0	0	0
N^8-Acetylspermidine_Post-WM	0.00742	0.0156	0.0172	0.00624	0.0124	0.0159	0.011	0	0.0229	0.0375	0	0.00825	0.0117	0	0	0.00795	0.0357	0.0121	0.0152	0.00964	0.0157	0.0139	0.0068	0.00982	0.0155	0.0106	0.0108	0	0.00735	0.00887	0.00431	0
N^1-Acetylspermidine_Post-WM	0.0586	0.197	0.217	0.0251	0.112	0.193	0.0672	0.09	0.189	0.243	0.0669	0.0542	0.0564	0.0741	0.0398	0.0716	0.316	0.212	0.172	0.0635	0.103	0.0623	0.0473	0.0699	0.138	0.0909	0.0896	0.166	0.0313	0.136	0.0412	0.107
Gly-Leu_Post-WM	0.0239	0.0755	0.0706	0.0134	0.0336	0.0264	0.0515	0.0894	0.0307	0.115	0.0387	0.0184	0.103	0.0705	0.0926	0.037	0.09	0.0849	0.16	0.0249	0.0842	0.0259	0.0239	0.0722	0.0808	0.0674	0.0303	0.0461	0.11	0.0262	0.0444	0.0233
N-ε-Acetyllysine_Post-WM	0	0.0462	0.0582	0.0367	0.0875	0.0518	0	0	0.0522	0.126	0.0787	0.0488	0.0448	0	0.0551	0.0574	0.152	0	0.0436	0	0.0742	0.0386	0.0377	0	0.0794	0.0396	0	0.042	0.0472	0.123	0.0355	0.0505
N^6,N^6,N^6-trimethyllysine_Post-WM	0.0255	0.0378	0.0304	0	0	0.0393	0.0242	0.0209	0.00945	0.0469	0.0293	0.0187	0.0298	0.0238	0.0398	0.0174	0.0536	0.0376	0.0365	0.0214	0.0159	0.0323	0.0168	0.0285	0.0311	0.0306	0.0408	0	0.0201	0	0	0.0314
Castanospermine_Post-WM	0	0	0	0	0	0	0	0	0	0	0	0	0	0	0	0	0	0	0	0	0	0	0	0	0	0	0	0	0	0	0	0

5-Methoxytryptamine_Post-WM	0	0	0	0	0	0	0	0	0	0	0	0	0	0	0	0	0	0	0	0	0	0	0	0	0	0	0	0	0	0	0	0
5-Hydroxyindoleacetate_Post-WM	0	0	0	0	0	0	0	0	0	0	0	0	0	0	0	0	0	0	0	0	0	0	0	0	0	0	0	0	0	0	0	0
SAH_Post-WM	0	0	0	0	0	0	0	0	0	0	0	0	0	0	0	0	0	0	0	0	0	0	0	0	0	0	0	0	0	0	0	0
Glucosaminate_Post-WM	0	0	0	0	0	0	0	0	0	0	0	0	0	0	0	0	0	0	0	0	0	0	0	0	0	0	0	0	0	0	0	0
DOPA_Post-WM	0	0	0	0	0	0	0	0	0	0	0	0	0	0	0	0	0	0	0	0	0	0	0	0	0	0	0	0	0	0	0	0
N-Acetylhistidine_Post-WM	0	0	0	0	0	0	0	0	0	0	0	0	0	0	0	0	0	0	0	0	0	0	0	0	0	0	0	0	0	0	0	0
SAM+_Post-WM	0	0.0637	0	0	0	0.0376	0.0213	0.0278	0	0.0457	0.0196	0	0.0205	0	0.0159	0	0.0317	0.0227	0.02	0.0137	0.0192	0.0183	0	0.012	0.0271	0.0236	0.0203	0.0214	0	0	0	0
Arginine ethyl ester_Post-WM	0	0	0	0	0	0	0	0	0	0	0	0	0	0	0	0	0	0	0	0	0	0	0	0	0	0	0	0	0	0	0	0
ADMA_Post-WM	0	0.053	0	0	0	0.00932	0.0254	0.0161	0.0235	0.026	0.0261	0	0	0.0202	0	0	0.018	0.01	0	0	0	0.0189	0.0146	0.00889	0.0279	0.0103	0	0	0.00961	0.0334	0	0.017
SDMA_Post-WM	0	0	0.0141	0.00708	0	0.0168	0	0	0	0	0.0187	0	0	0	0	0	0.0203	0.0111	0	0	0	0.0097	0	0	0	0.00999	0	0	0	0.0235	0	0
o-Acetylcarnitine_Post-WM	0.281	1.54	0.278	0.237	0.304	0.312	0.292	0.249	0.335	0.537	0.226	0.564	0.505	0.448	0.499	0.276	0.795	0.317	0.439	0.249	0.344	0.185	0.481	0.409	0.397	0.535	0.164	0.679	0.253	0.427	0.165	0.205
Trp_Post-WM	0.128	0.303	0.189	0.11	0.188	0.178	0.218	0.175	0.132	0.332	0.122	0.0734	0.16	0.189	0.315	0.169	0.419	0.276	0.239	0.0658	0.185	0.101	0.0912	0.111	0.18	0.19	0.141	0.0993	0.201	0.0393	0.172	0.103
5-Methoxyindoleacetate_Post-WM	0	0.796	0.984	0	0.725	0.925	0.837	0.975	0.954	0.867	0.632	0.662	0.894	0.992	1.81	0	1.27	0	0.641	0.574	1.34	0.749	0.446	0.58	0.764	0	0.843	0.638	0	0	0	0.946
Kynurenine_Post-WM	0	0	0	0	0	0	0	0	0	0	0	0	0	0	0	0	0	0	0	0	0	0	0	0	0	0	0	0	0	0	0	0
Cysteine-glutathione disulfide_Post-WM	0	0.459	0.106	0	0	0	0	0.144	0	0	0	0	0.111	0.1	0.424	0.0422	0	0.0684	0	0.0633	0.0797	0	0	0.0293	0.0666	0.102	0.171	0	0	0	0	0
Desthiobiotin_Post-WM	0	0	0	0	0	0	0	0	0	0	0	0	0	0	0	0	0	0	0	0	0	0	0	0	0	0	0	0	0	0	0	0.0782
o-Succinylhomoserine_Post-WM	0	0	0	0	0	0	0	0	0	0	0	0	0	0	0	0	0	0	0	0	0	0	0	0	0	0	0	0	0	0	0	0
trans-Zeatin_Post-WM	0	0	0	0	0	0	0	0	0	0	0	0	0	0	0	0	0	0	0	0	0	0	0	0	0	0	0	0	0	0	0	0
5-Hydroxytryptophan_Post-WM	0	0	0	0	0	0	0	0	0	0	0	0	0	0	0	0	0	0	0	0	0	0	0	0	0	0	0	0	0	0	0	0
N-Acetylglucosylamine_Post-WM	0	0	0	0	0	0	0	0	0	0	0	0	0	0	0	0	0	0	0	0	0	0	0	0	0	0	0	0	0	0	0	0
N-Acetylglucosamine_Post-WM	3.34	2.07	7.08	1.59	1.11	3.9	3.7	3.01	3.27	13.8	2.22	15.4	1.5	4.31	2.16	3.64	2.51	6.71	5.36	3.82	11.2	5.18	5.71	17.6	2.73	8.28	1.06	1.47	2.85	6.69	2.49	1.19
Cystathionine_Post-WM	0	0	0	0	0	0	0	0	0	0	0	0	0	0	0	0	0	0	0	0	0	0	0	0	0	0	0	0	0	0	0	0
3-Hydroxykynurenine_Post-WM	0	0	0	0	0	0	0	0	0	0	0	0	0	0	0	0	0	0	0	0	0	0.0456	0.0254	0	0	0	0	0	0	0	0	0
2'-Deoxycytidine_Post-WM	0.0395	0	0	0	0.0302	0	0	0	0	0	0	0	0	0	0	0	0.0311	0	0.0245	0	0	0.0294	0	0	0	0.0391	0	0	0	0	0	0
N^1,N^8-Diacetylspermidine_Post-WM	0.00765	0.0271	0.0219	0	0.0179	0	0.0157	0.0291	0.018	0.0227	0.0304	0.0212	0.0297	0.0161	0	0.014	0.0258	0.0288	0	0.0109	0.0204	0.0244	0.00815	0.0118	0.02	0.0148	0.0222	0	0.0123	0.0173	0.00968	0
γ-Glu-2AB_Post-WM	0	0	0	0	0	0	0	0	0	0	0	0	0	0	0	0	0	0	0	0	0	0	0	0	0	0	0	0	0	0	0	0
Melatonin_Post-WM	0	0	0	0	0	0.0532	0	0	0	0	0.104	0	0	0	0	0	0	0	0	0.0304	0.232	0	0	0	0.057	0	0	0	0.0426	0	0.0557	0
Sepiapterin_Post-WM	0	0	0	0	0	0	0	0	0	0	0	0	0	0	0	0	0	0	0	0	0	0	0	0	0	0	0	0	0	0	0	0
7,8-Dihydrobiopterin_Post-WM	0	0	0	0	0	0	0	0	0	0	0	0	0	0	0	0	0	0	0	0	0	0	0	0	0	0	0	0	0	0	0	0
Cystine_Post-WM	0	0.107	0.0822	0	0.035	0.041	0	0	0.0721	0.0799	0.0414	0	0.0552	0	0.088	0.037	0.211	0.0415	0	0.0324	0	0	0.0587	0	0	0.063	0.126	0	0.0541	0	0.0345	0
5-Methyl-2'-deoxycytidine_Post-WM	0	0	0	0	0	0	0	0	0	0	0	0	0	0	0	0	0	0	0	0	0	0	0	0	0	0	0	0	0	0	0	0
Thymidine_Post-WM	0.583	0	0.512	0.996	0	0	0	1.57	0.927	0.865	0.878	0	0.582	0	1.57	0.781	1.07	0	0.679	0.832	1.41	0.513	0.709	0	0.93	0.982	0	0	0.71	0	0.655	1.23
Cytidine_Post-WM	0.0791	0	0.143	0.0997	0.0787	0.139	0.0666	0.0802	0.134	0.386	0.209	0.257	0.0296	0.098	0	0.133	0.0832	0.187	0.223	0.0589	0.148	0.0994	0.111	0.291	0.151	0.224	0	0.0675	0.112	0.29	0.0116	0
Uridine_Post-WM	1.25	1.94	0.875	1.14	1.46	0.829	1.08	1.22	1.96	2.36	1.37	1.91	1.14	1.42	2.37	1.06	3.06	1.83	1.87	1.21	3.23	1.08	1.09	1.8	1.82	1.75	1.05	1.46	1.44	1.56	1.28	1.91
N^1-Acetylspermine_Post-WM	0	0	0.0269	0	0	0	0.0215	0	0	0.0156	0	0.0069	0.0296	0.0185	0	0.0145	0.0105	0.0268	0	0	0	0.0125	0	0.0136	0.0188	0	0	0	0	0.0131	0	0
Octopine_Post-WM	0	0	0	0	0.0343	0.0185	0	0.0101	0	0.1	0	0	0	0.00797	0	0	0.0308	0	0.0528	0	0.008	0	0	0	0	0	0	0	0	0	0	0
Pyridoxamine 5'-phosphate_Post-WM	0	0	0	0	0	0	0	0	0	0	0	0	0	0	0	0	0	0	0	0	0	0	0	0	0	0	0	0	0	0	0	0
γ-Glu-cys_Post-WM	0	0	0	0	0	0	0	0	0	0	0	0	0	0	0	0	0	0	0	0	0	0	0	0	0	0	0	0	0	0	0	0
Muramate_Post-WM	0	0	0	0	0	0	0	0	0	0	0	0	0	0	0	0	0	0	0	0	0	0	0	0	0	0	0	0	0	0	0	0
5'-Deoxyadenosine_Post-WM	0	0	0	0	0	0	0	0	0	0	0	0	0	0	0	0	0	0	0	0	0	0	0	0	0	0	0	0	0	0	0	0
Purine riboside_Post-WM	0	0	0	0	0	0	0	0	0	0	0	0	0	0	0	0	0	0	0	0	0	0	0	0	0	0	0	0	0	0	0	0
2'-Deoxyinosine_Post-WM	0	0	0	0	0	0	0	0	0	0	0	0	0	0	0	0	0	0	0	0	0	0	0	0	0	0	0	0	0	0	0	0
7,8-Dihydroneopterin_Post-WM	0	0	0	0	0	0	0	0	0	0	0	0	0	0	0	0	0	0	0	0	0	0	0	0	0	0	0	0	0	0	0	0
Glycerophosphorylcholine_Post-WM	0.116	1.2	0	0.354	1.49	0	0	0.176	0.192	0.143	0	0.329	1.09	0.767	1.9	0.115	0	0	0	0.45	0	0.34	0.336	0	0	0.095	0.257	1.39	0	0.411	0.358	0.517
Thiamine_Post-WM	0	0	0	0	0	0	0	0	0	0	0	0	0	0	0	0	0	0	0	0	0	0	0	0	0	0	0	0	0	0	0	0
N^6-Methyl-2'-deoxyadenosine_Post-WM	0	0	0	0	0	0	0	0	0	0	0	0	0	0	0	0	0	0	0	0	0	0	0	0	0	0	0	0	0	0	0	0
Adenosine_Post-WM	0.0678	0.144	0.0759	0.0484	0.0313	0.0726	0.04	0.0734	0.113	0.0732	0.238	0.087	0.0311	0.0716	0.121	0.0322	0.117	0.0351	0.0369	0.0464	0.0424	0.0702	0.0636	0.0579	0.0363	0.0536	0.132	0.0432	0.039	0.0928	0.0478	0.0643
2'-Deoxyguanosine_Post-WM	0	0	0	0	0	0	0	0	0	0	0	0	0	0	0	0	0	0	0	0	0	0	0	0	0	0	0	0	0	0	0	0
Homocystine_Post-WM	0	0	0	0	0	0	0	0	0	0	0	0	0	0	0	0	0	0	0	0	0	0	0	0	0	0	0	0	0	0	0	0
Inosine_Post-WM	0.195	0.871	0.392	0	0.285	0.477	0.255	0.47	0.279	0	0.274	0	0.148	0.241	0.289	0.12	0.437	0.275	0.173	0.228	0.508	0.329	0.214	0	0.42	0.529	0.346	0.211	0.154	0.16	0.277	0.299
Glu-Glu_Post-WM	0	0.0513	0.0411	0	0	0.03	0	0	0	0.117	0	0	0.0676	0	0	0	0.064	0.0421	0.0465	0	0.0627	0	0	0.0426	0	0.0772	0	0	0	0	0	0
Saccharopine_Post-WM	0	0	0	0	0	0	0	0	0	0.0642	0	0	0.0413	0.0172	0	0	0.126	0	0	0	0	0	0	0	0	0	0	0	0	0.0364	0	0
1-Methyladenosine_Post-WM	0.0164	0.0376	0.0194	0.0105	0.0133	0.0114	0.0114	0	0.0126	0.021	0	0.0171	0.0187	0.0239	0.031	0.0143	0.0298	0.018	0.0102	0.0162	0.0133	0.0183	0	0.022	0.0191	0.00821	0.0182	0.0166	0.00908	0	0.011	0
Guanosine_Post-WM	0.0807	0.287	0.188	0.0536	0.122	0.29	0.106	0.187	0.167	0.0577	0.196	0.111	0.056	0.0897	0.178	0.048	0.198	0.106	0.0358	0.134	0.164	0.174	0.132	0.112	0.165	0.155	0.219	0.128	0.0436	0.0922	0.125	0.134
Xanthosine_Post-WM	0	0	0	0	0	0	0	0	0	0	0	0	0	0	0	0	0	0	0	0	0	0	0	0	0	0	0	0	0	0	0	0
Ophthalmate_Post-WM	0	0	0	0	0	0	0	0	0	0	0	0	0	0	0.0363	0	0	0	0	0	0	0	0	0	0	0	0	0	0	0	0	0
5-Methylthioadenosine_Post-WM	0	0	0	0	0	0	0	0	0	0	0	0	0	0	0	0	0	0	0	0	0	0	0	0	0	0	0	0	0	0	0	0
Glutathione(ox)_Post-WM	0.0332	0.992	0.0894	0.0278	0	0.0858	0.0308	0.308	0.0621	0.0251	0	0.0661	0.0258	0.0567	0.824	0.0308	0	0	0	0.0579	0.045	0.129	0.0213	0.028	0	0.0789	0.112	0.0473	0	0.0248	0	0
N-α-Benzenolarginine ethylester_Post-WM	0	0	0	0	0	0	0	0	0	0	0	0	0	0	0	0	0	0	0	0	0	0	0	0	0	0	0	0	0	0	0	0
3-Iodotyrosine_Post-WM	0	0	0	0	0	0	0	0	0	0	0	0	0	0	0	0	0	0	0	0	0	0	0	0	0	0	0	0	0	0	0	0
Glutathione(red)_Post-WM	0	0	0	0	0	0	0	0	0	0	0	0	0	0	0.433	0	0	0	0	0	0	0	0	0	0	0	0	0	0	0	0	0
Phe-Phe_Post-WM	0	0	0	0	0.02	0	0.0101	0.0087	0	0.0402	0	0	0	0.0176	0.00455	0.0132	0	0.0194	0.0293	0	0.0224	0	0	0.0284	0	0	0	0.011	0.00435	0	0.0094	0
AICAR_Post-WM	0	0.458	0	0	0	0.33	0	0.531	0.493	0	0.301	0	0	0.541	0.566	0	0.617	0	0.346	0	0	0	0	0	0	0	0	0.314	0	0	0	0.458
Thiamine monophosphate_Post-WM	0	0	0	0	0	0	0	0	0	0	0	0	0	0	0	0	0	0	0	0	0	0	0	0	0	0	0	0	0	0	0	0
Tetrahydropalmatine_Post-WM	0	0	0	0	0	0	0	0	0	0	0	0	0	0	0	0	0	0	0	0	0	0	0	0	0	0	0	0	0	0	0	0
TRH_Post-WM	0	0	0	0	0	0	0	0	0	0	0	0	0	0	0	0	0	0	0	0	0	0	0	0	0	0	0	0	0	0	0	0
Riboflavin_Post-WM	0	0	0	0	0	0	0	0	0	0	0	0	0	0	0	0	0	0	0	0	0	0	0	0	0	0	0	0	0	0	0	0
S-Lactoylglutathione_Post-WM	0	0	0	0	0	0	0	0	0	0	0	0	0	0	0	0	0	0	0	0	0	0	0	0	0	0	0	0	0	0	0	0
Leu-Leu-Tyr_Post-WM	0	0	0	0	0	0	0	0	0	0	0	0	0	0	0	0	0	0	0	0	0	0	0	0	0	0	0	0	0	0	0	0
3,5-Diiodo-tyrosine_Post-WM	0	0	0	0	0	0	0	0	0	0	0	0	0	0	0	0	0	0	0	0	0	0	0	0	0	0	0	0	0	0	0	0
5-Methyltetrahydrofolate_Post-WM	0	0	0	0	0	0.515	0	0.367	0.462	0	0	0	0	0	0	0	0	0	0.982	0	1.32	0	0	0	0	0	0	0	0	0	0	0
3,3',5-Triiodothyronine_Post-WM	0	0	0	0	0	0	0	0	0	0	0	0	0	0	0	0	0	0	0	0	0	0	0	0	0	0	0	0	0	0	0	0
NMN_Post-WM	0	0	0	0	0	0	0	0	0	0	0	0	0	0	0	0	0	0	0	0	0	0	0	0	0	0.0474	0	0	0	0	0	0
Glyoxylate_Post-WM	0	0	0.256	0.674	0.685	0	0	0.883	0.95	0.308	1.19	0	0	0.92	0	0	0	0.439	0.592	0.421	0.647	0	0	0	0	0	0.235	0	0	0.27	0.844	0
Propionate_Post-WM	4.46	86.1	10.7	5.39	2.79	7.05	16.4	11.1	9.54	42.1	3.98	8.87	13.6	11.5	4.07	17.9	23.2	5.22	5.83	2.33	4.09	6.84	5.89	9.42	6.91	9.45	4.26	1.81	23.1	4.03	9.06	1.58
Glycolate_Post-WM	3.29	4.8	2.31	2.48	0	2.82	3.78	0	2.49	3.67	4.51	1.9	2.36	3.46	4.58	3.8	2.38	3.04	3.71	2.66	2.77	3.52	1.9	2.21	2.52	4.41	0	1.97	4.14	2.93	2.33	0
Crotonate_Post-WM	0	0	0	0	0	0.428	0	0	0	1.08	0	0	0	0	0	0	0	0	0	0	0	0	0	0	0	0	0	0	1.35	0	0	0
Pyruvate_Post-WM	1.58	11.3	2.84	2.17	3.49	2.49	2.91	3.59	4.41	5.05	3.7	2.68	2.39	2.98	3.49	3.78	3.79	3.25	6.54	1.55	5.11	2.12	1.9	3.32	3.77	2.62	1.69	2.2	3.58	3.92	0	2.03
Butyrate_Post-WM	1.21	14.6	1.97	1.15	0	3.13	5.16	3.77	2.38	7.96	1.35	2.24	2.66	2.67	2.14	4.01	6.41	1.72	1.55	0.956	1.07	0.969	1.44	3.75	1.51	3.43	0.811	0	4.64	0	2.04	0
Lactate_Post-WM	74.9	197	49.6	136	73.7	56.5	54.3	68.2	97.4	189	94.3	66.6	70.6	162	114	104	116	71.1	143	108	83.8	80.9	30.2	84.3	154	136	34.2	56.6	102	69.9	34.4	62.4
3-Hydroxypropionate_Post-WM	11.2	12.4	9.91	12.8	4.35	6.44	7.08	8.73	12.8	11.1	10.9	7.37	6.69	9.51	11.9	9.65	12.5	7.3	23.1	9.45	7.28	14.9	6.82	10.5	39.3	13.5	5.18	8.47	9.47	6.04	4.47	6.51
Methanesulfonate_Post-WM	0	0	0	0	0	0	0	0	0	0	0	0	0	0	0	0	0	0	0	0	0	0	0	0	0	0	0	0	0	0	0	0
Tiglate_Post-WM	0	0	0	0	0	0	0	0	0	0	0	0	0	0	0	0	0	0	0	0	0	0	0	0	0	0	0	0	0	0	0	0
2-Oxobutyrate_Post-WM	0	0	0	0	0	0	0	0	0	0	0	0	0	0	0	0	0	0	0	0	0	0	0	0	0	0	0	0	0	0	0	0
Pentanoate_Post-WM	0	0	0	0	0	0	0	0	0	0	0	0	0	0	0	0	0	0	0	0	0	0	0	0	0	0	0	0	0	0	0	0
3-Methylbutanoate_Post-WM	0.483	3.54	0.727	0	0	0.907	1.81	1.14	1.16	2.94	0.682	0.495	0.488	1	0	1.35	3.17	0.578	1.29	0	0.574	0.427	0.315	0.779	0.896	1.35	0	0	1.48	0	0.734	0
Malonate_Post-WM	0.316	0.355	0.394	0.355	0.181	0.129	0.261	0.132	0.282	0.655	0.394	0.304	0.317	0.168	0.268	0.307	0.631	0.345	0.425	0.295	0.24	0.399	0.189	0.371	0.409	0.475	0.166	0.197	0.391	0.231	0.238	0.289
2-Hydroxyisobutyrate_Post-WM	0	0	0	0	0	0	0	0	0	0	0	0	0	0	0	0	0	0	0	0	0	0	0	0	0	0	0	0	0	0	0	0
2-Hydroxybutyrate_Post-WM	0	0.59	0.16	0.355	0	0.364	0.378	0	0.36	0.64	0.333	0.189	0.488	0.553	0.444	0.599	1.63	0.283	0.806	0.333	0.393	0.24	0.137	0.527	0.477	0.356	0	0.47	0.801	0.226	0	0
3-Hydroxybutyrate_Post-WM	0.787	10.1	1.19	1.45	0.987	2.85	1.28	0.455	1.36	3.14	1.62	1.43	1.53	1.79	1.58	3.95	2.96	1.24	3.16	1.43	1	1.36	0.657	1.2	2.9	2.85	0.601	0.735	2.78	0.613	0.549	0.836
(Methylthio)acetate_Post-WM	0	0	0	0	0	0	0	0	0	0	0	0	0	0	0	0	0	0	0	0	0	0	0	0	0	0	0	0	0	0	0	0

Glycerate_Post-WM	0.573	1.03	0.769	0.781	0.763	0.395	0.742	0.781	0.623	1.01	0.949	1.22	0.594	1.43	1.22	0.654	0.641	0.809	0.862	0.779	0.793	0.757	0.491	0.845	1.07	1.06	0.909	0.615	1.03	1.1	0.381	0.769
Pyrrole-2-carboxylate_Post-WM	0	0	0	0	0	0	0	0	0	0	0	0	0	0	0	0	0	0	0	0	0	0	0	0	0	0	0	0	0	0	0	0
Methyl sulfate_Post-WM	0	0	0	0	0	0	0	0	0	0	0	0	0	0	0	0	0	0	0	0	0	0	0	0	0	0	0	0	0	0	0	0
2-Furoate_Post-WM	0	0	0	0	0	0	0	0	0	0	0	0	0	0	0	0	0	0	0	0	0	0	0	0	0	0	0	0	0	0	0	0
Fumarate_Post-WM	0.344	0.508	0.292	0.292	0.279	0.31	0.275	0.378	0.433	0.298	0.307	0.466	0.475	0.396	1.2	0.261	0.61	0.562	0.347	0.367	0.387	0.331	0.259	0.363	0.5	0.445	0.394	0.491	0.223	0.413	0.211	0.393
2-Oxoisopentanoate_Post-WM	0	0	0.0936	0.105	0	0	0	0	0	0.304	0	0.188	0.123	0.291	0	0.213	0	0.0921	0.278	0.0636	0.328	0.103	0	0.201	0.222	0.119	0.0886	0	0.222	0.085	0	0
4-Oxopentanoate_Post-WM	0.118	0.33	0.138	0.215	0	0.143	0	0	0	0.227	0	0	0	0	0	0.307	0.233	0.105	0.537	0.184	0	0.174	0	0.0957	0.351	0.396	0.0764	0	0.709	0	0.075	0
Hexanoate_Post-WM	0.201	0	0.267	0.0729	0	0.559	0.386	0.523	0	1.82	0.254	0.182	0.215	0.507	0	0.694	0.923	0.347	0.666	0.16	0	0	0.137	0.433	0.587	0.635	0	0	1	0	0.394	0
Succinate_Post-WM	16.9	6.09	23.5	33.8	19.9	22.3	23.7	13.6	10.2	25	9.2	19.7	22.7	15.9	6.74	19.5	8.94	14.3	11.2	15.5	17.3	11.4	13.1	19.5	8.32	14.9	8.44	8.72	26.7	45.7	11.4	10.3
2-Hydroxypentanoate_Post-WM	0.256	0.68	0.365	0.569	0.219	0.327	0.244	0.228	0.376	1.34	0.619	0.221	0.304	0.527	0.255	0.888	1.32	0.349	1.09	0.337	0.282	0.293	0.296	0.312	0.895	0.624	0.154	0.201	1.15	0.128	0.219	0.254
Benzoate_Post-WM	0.297	0	0.0972	0	0	0	0	0	0	0	0	0.248	0.167	0	0	0.111	0.505	0.12	0	0.102	0	0.269	0.0938	0	0.218	0.212	0	0	0.653	0	0	0
Nicotinate_Post-WM	0.151	0.594	0.217	0	0	0.331	0.205	0.378	0.58	0.353	0.42	0.118	0.231	0.375	0.237	0.249	0.318	0.278	0.493	0.146	0	0.23	0.18	0.205	0.43	0.527	0.196	0	0.466	0	0.18	0
1-Aminoethylphosphonate_Post-WM	0	0	0	0	0	0	0	0	0	0	0	0	0	0	0	0	0	0	0	0	0	0	0	0	0	0	0	0	0	0	0	0
2-Aminoethylphosphonate_Post-WM	0	0	0	0	0	0	0	0	0	0	0	0	0	0	0	0	0	0	0	0	0	0	0	0	0	0	0	0	0	0	0	0
Isethionate_Post-WM	0	0	0	0	0	0	0	0	0	0.15	0	0.0685	0	0	0.194	0.086	0.234	0	0	0	0	0	0	0	0	0.0981	0	0	0	0	0	0
Barbiturate_Post-WM	0	0	0	0	0	0	0	0	0	0	0	0	0	0	0	0	0	0	0	0	0	0	0	0	0	0	0	0	0	0	0	0
5-Oxoproline_Post-WM	1.02	2.93	2.3	1.57	3	2.2	1.54	1.47	1.35	2.11	1.39	1.07	2.25	1.18	2.37	1.18	1.98	1.87	1.35	1.02	1.55	1.5	1.1	2.3	3.12	2.68	1.71	1.4	1.53	2.09	1.59	1.17
Itaconate_Post-WM	0	0	0	0	0	0	0	0	0	0	0	0	0	0	0	0	0	0	0	0	0	0	0	0	0	0	0	0	0	0	0	0
Citraconate_Post-WM	0	0	0	0	0	0	0	0	0	0	0	0	0	0	0	0	0	0	0	0	0	0	0	0	0	0	0	0	0	0	0	0
4-Methyl-2-oxopentanoate_Post-WM	0.138	0.714	0.256	0.214	0.278	0.372	0.229	0	0.398	0.608	0.332	0.236	0.255	0.322	0.348	0.526	0.33	0.244	0.537	0.186	0.346	0.183	0.147	0.432	0.405	0.329	0	0.318	0.489	0.198	0.0796	0.245
4-Oxohexanoate_Post-WM	0	0	0	0	0	0	0	0	0	0	0	0	0	0	0	0	0	0	0	0	0	0	0	0	0	0	0	0	0	0	0	0
4-Acetylbutyrate_Post-WM	0	0	0.0402	0.0967	0	0	0	0	0	0.137	0	0.0552	0	0	0	0.145	0	0.0443	0.204	0.0732	0	0.043	0	0.0656	0.161	0.137	0	0	0.323	0	0	0
Heptanoate_Post-WM	0	0	0	0	0	0	0	0	0	0	0	0	0	0	0	0	0	0	0	0	0	0	0	0	0	0	0	0	0	0	0	0
N-acetyl-*β*-alanine_Post-WM	0	0	0.237	0.202	0	0	0.146	0.128	0	0.459	0	0.148	0.249	0.132	0.306	0	0.318	0.213	0	0	0	0.173	0	0.166	0.222	0.209	0.173	0	0.155	0.164	0.127	0
Glutarate_Post-WM	0	0	0	0	0	0	0	0	0	0	0	0	0	0	0	0	0	0	0	0	0	0	0	0	0	0	0	0	0	0	0	0
3-Ureidopropionate_Post-WM	0	0	0	0	0	0	0	0	0	0	0	0	0	0	0	0	0	0	0	0	0	0	0	0	0	0	0	0	0	0	0	0
2-Hydroxy-4-methylpentanoate_Post-WM	0.18	0.28	0.411	0.282	0.171	0.41	0.283	0.145	0.346	1.36	0.511	0.178	0.174	0.283	0.102	0.988	0	0.32	0.405	0.148	0.283	0.189	0.29	0.261	0.346	0.316	0.11	0	0.577	0.116	0.201	0.113
6-Hydroxyhexanoate_Post-WM	0	0	0	0	0	0	0	0	0	0.186	0	0	0.159	0	0	0.292	0	0	0.952	0	0	0	0	0	0.446	0.292	0	0	0.43	0	0	0
Malate_Post-WM	1.99	2.67	1.54	1.87	1.66	1.43	0.983	1.37	1.66	2.57	1.29	3.08	2.39	1.8	6.46	1.57	3.41	3.82	1.09	2.44	2.22	1.69	1.65	2.36	3	3.4	1.6	2.38	2.08	2.05	1.29	1.91
Threonate_Post-WM	0.49	0.468	0.663	0.46	0.601	0.442	0	0.28	0.508	0.642	0.546	0.78	0.425	0	1.23	0	0	0.653	0	0.366	0	0.387	0.595	0.909	0.911	0	0.288	0.449	0	0.668	0.488	0.442
o-Hydroxybenzoate_Post-WM	0	0	0	0	0	0	0	0	0	0	0	0	0.0551	0	0	0.0441	0	0	0	0	0	0	0	0	0.129	0.0433	0.125	0	0	0	0	0
p-Hydroxybenzoate_Post-WM	0	0.515	0	0	0	0	0	0	0	0	0	0.554	0	0	0.557	0	0.373	0	0	0	0	0	0	0.432	0.424	0	0.141	0	0	0	0	0
m-Hydroxybenzoate_Post-WM	0	0	0	0	0	0	0	0	0	0	0	0	0	0	0	0	0	0	0	0	0	0	0	0	0	0	0	0	0	0	0	0
Phosphonoacetate_Post-WM	0	0	0	0	0	0	0	0	0	0	0	0	0	0	0	0	0	0	0	0	0	0	0	0	0	0	0	0	0	0	0	0
Carbamoylphosphate_Post-WM	0	0	0	0	0	0	0	0	0	0	0	0	0	0	0	0	0	0	0	0	0	0	0	0	0	0	0	0	0	0	0	0
Ethanolamine phosphate_Post-WM	15.9	112	12.5	17.9	35.4	22.9	18	25.2	16	2.7	14.4	11.9	29.3	24.4	48.5	26	38	9.12	11.2	14.5	18.6	9.87	31.4	17.5	13.2	8.82	21.9	34.9	18.1	33.8	15.7	21.9
2-Thiopheneacetate_Post-WM	0	0	0	0	0	0	0	0	0	0	0	0	0	0	0	0	0	0	0	0	0	0	0	0	0	0	0	0	0	0	0	0
Octanoate_Post-WM	0	0	0	0	0	0	0	0	0	0	0	0	0	0	0	0	0	0	0	0	0	0	0	0	0	0	0	0	0	0	0	0
2-Oxoglutarate_Post-WM	0.33	1.51	0.22	0.515	0	0	0.38	0	0.836	0.587	0	0.507	0.57	0	0	0	0	0	0	0	0.656	0	0	0.767	0.264	0.265	0	0	0	1.1	0	0
Adipate_Post-WM	0	0	0	0	0	0	0	0	0	0	0	0	0	0	0	0	0	0	0	0	0	0	0	0	0	0	0	0	0	0	0	0
threo-*β*-methylaspartate_Post-WM	0	0	0	0	0	0	0	0	0	0	0	0	0	0	0	0	0	0	0	0	0	0	0	0	0	0	0	0	0	0	0	0
4-Methylthio-2-oxobutyrate_Post-WM	0	0	0	0	0	0	0	0	0	0	0	0	0	0	0	0	0	0	0	0	0	0	0	0	0	0	0	0	0	0	0	0
2-Hydroxyglutarate + Citramalate_Post-WM	0	0.313	0.385	0.444	0.438	0.379	0.297	0	0.463	1.56	0.5	0.655	0.439	0.477	0	0.519	0	0.597	0.2	0.144	0.482	0.24	0	0	0.625	0.681	0	0	0.515	0	0	0.227
trans-Cinnamate_Post-WM	0.182	0.45	0.694	0.35	0.334	0.294	0.42	0.295	0.321	0.773	0.326	0.593	0.362	0.507	1.1	0.463	0.527	0.582	0.398	0.272	0.444	0.317	0.291	0.396	0.544	0.594	0.284	0.234	0.398	0.591	0.197	0
Tartrate_Post-WM	0	0	0	0	0	0	0	0	0	0	0	0	0	0	0	0	0	0	0	0	0	0	0	0	0	0	0	0	0	0	0	0
Benzoylformate_Post-WM	0	0	0	0	0	0	0	0	0	0	0	0	0	0	0	0	0	0	0	0	0	0	0	0	0	0	0	0	0	0	0	0
3-Phenylpropionate_Post-WM	0.889	0.479	0.768	0.744	0.599	1.03	0.829	0.994	0.539	4.31	0.606	0.608	0.781	1.45	0.495	3.08	1.71	1.31	4.74	1.1	0.863	0.545	0.41	1.22	2.92	3.31	0.384	0.396	6.26	0	0.648	0.436
2-Hydroxyphenylacetate_Post-WM	0	0	0	0	0	0	0	0	0	0	0	0	0	0	0	0	0	0	0	0	0	0	0	0	0	0	0	0	0	0	0	0
p-Hydroxyphenylacetate_Post-WM	0.368	10.1	3.77	0	1.11	0.99	7.87	3.52	5.42	0.869	1.13	0.665	1.96	0	0	0.558	1.97	1.34	2.15	1.09	0.972	3.86	2.17	0.957	0.614	5.17	2.08	0	0	0	1.87	0
Cysteine sulfinate_Post-WM	0.226	0	0.406	0.303	0	0	0	0	0	0.386	0.464	0	0	0	0	0.191	0	0.309	0	0	0	0.165	0	0	0.404	0.287	0.299	0.244	0.279	0.193	0	0
2,5-Dihydroxybenzoate_Post-WM	0.338	0	0.498	0	0	0	0	0	0	0	0.59	0	0	0	0	0	0	0.265	0	0.233	0	0.611	0.408	0	0.572	0	0	0	0	0.933	0.128	0
Orotate_Post-WM	0	0	0	0	0	0	0	0	0	0	0	0	0	0	0	0	0	0	0	0	0	0	0	0	0	0	0	0	0	0	0	0
2,4-Dihydroxypyrimidine-5-carboxylate_Post-WM	0	0	0	0	0	0	0	0	0	0	0	0	0	0	0	0	0	0	0	0	0	0	0	0	0	0	0	0	0	0	0	0
Dihydroorotate_Post-WM	0	0	0	0	0	0	0	0	0	0	0	0	0	0	0	0	0	0	0	0	0	0	0	0	0	0	0	0	0	0	0	0
2-Oxooctanoate_Post-WM	0	0	0	0	0	0	0	0	0	0	0	0	0	0	0	0	0	0	0	0	0	0	0	0	0	0	0	0	0	0	0	0
Pelargonate_Post-WM	0	0	0	0	0	0	0	0	0	0	0	0	0	0	0	0	0	0	0	0	0	0	0	0	0	0	0	0	0.198	0	0	0
2-Oxoadipate_Post-WM	0	0	0	0	0	0	0	0	0	0	0	0	0	0	0	0	0	0	0	0	0	0	0	0	0	0	0	0	0	0	0	0
Pimelate_Post-WM	0	0	0	0	0	0	0	0	0	0	0	0	0	0	0	0	0	0	0	0	0	0	0	0	0	0	0	0	0	0	0	0
2-Hydroxyoctanoate_Post-WM	0	0	0	0	0	0	0	0	0	0	0	0	0	0	0	0	0	0	0	0	0	0	0	0	0	0	0	0	0	0	0	0
N-Formylaspartate_Post-WM	0	0	0	0	0	0	0	0	0	0	0	0	0	0	0	0	0	0	0	0	0	0	0	0	0	0	0	0	0	0	0	0
3-Hydroxy-3-methylglutarate_Post-WM	0	0.0544	0.0471	0.0604	0.0722	0	0	0	0.0446	0.0724	0.105	0.034	0	0.0377	0.0417	0	0.128	0.0495	0.0603	0	0.0713	0.0405	0	0.0449	0.074	0	0	0.0409	0	0.0288	0.035	0.058
Phenylpyruvate_Post-WM	0	0	0	0	0	0	0	0	0	0	0	0.053	0	0	0	0	0	0	0	0	0	0	0	0	0	0	0	0	0	0	0	0
p-Coumarate_Post-WM	0	0	0	0	0	0	0	0	0	0	0	0	0	0	0	0	0	0	0	0	0	0	0	0	0	0	0	0	0	0	0	0
o-Coumarate_Post-WM	0	0	0	0	0	0	0	0	0	0	0	0	0	0	0	0	0	0	0	0	0	0	0	0	0	0	0	0	0	0	0	0
Terephthalate_Post-WM	0	0	0	0	0.221	0	0	0	0	0	0	0	0	0	0	0	0	0	0	0	0	0	0	0	0	0	0	0	0	0	0	0
Phthalate_Post-WM	0.151	0.093	0.164	0.199	0.0921	0.0928	0.139	0.0907	0.136	0.176	0.116	0.128	0.112	0.0779	0.0877	0.128	0.163	0.133	0.117	0.122	0.16	0.112	0.114	0.106	0.191	0.123	0.12	0.113	0.171	0.107	0.122	0.0948
3-Phenyllactate_Post-WM	0.214	0.168	0.37	0.331	0.217	0.395	0.291	0.23	0.261	1.03	0.457	0.289	0.203	0.271	0.173	0.315	0	0.435	0.302	0.0768	0.421	0.211	0.364	0.324	0.315	0.377	0.132	0.114	0.295	0.267	0.126	0.127
3-(2-Hydroxyphenyl)propionate_Post-WM	0	0	0	0	0	0	0	0	0	0	0	0	0	0	0	0	0	0	0	0	0	0	0	0	0	0	0	0	0	0	0	0
3-(4-Hydroxyphenyl)propionate_Post-WM	1.81	6.05	2.91	0.75	0.596	0.615	7.12	3.06	2.92	3.86	2.84	1.32	3.08	1.18	0.379	3.65	0.884	1.89	1.72	1.02	1.48	2.8	2.64	1.39	2.55	2.36	2.1	0	1.37	0	4.65	0
2,3-Pyridinedicarboxylate_Post-WM	0	0	0	0	0	0	0	0	0	0	0	0	0	0	0	0	0	0	0	0	0	0	0	0	0	0	0	0	0	0	0	0
PEP_Post-WM	0	0.422	0	0	0	0	0.159	0.211	0.135	0.175	0.214	0.241	0.211	0.23	0.98	0.13	0.884	0.187	0	0.137	0.116	0.138	0	0.247	0.204	0.119	0.191	0.247	0.13	0.155	0.13	0.183
Urate_Post-WM	52.9	99.7	60.3	42.4	71.6	91.4	46.4	32.3	56.7	54.8	114	67.6	35.7	57.6	148	44.9	150	55.8	109	47.8	55.7	39.2	65.3	59.4	48.8	47.5	58.1	65.9	100	108	24.4	37.4
4-Hydroxymandelate_Post-WM	0.42	4.14	1.99	0.175	0.442	1.02	5.15	1.15	2.9	0.743	0.748	0.763	1.45	0.837	0	0.835	2.13	1.11	1.14	0.623	0.802	2.25	1.27	0.875	0.632	2.7	1.44	0	0.873	0	1.11	0
Cysteate_Post-WM	0	0	0	0	0	0.148	0	0	0	0	0	0	0	0	0	0	0	0	0.162	0	0	0	0	0	0	0	0	0	0	0	0	0
2-Amino-3-phosphonopropionate_Post-WM	0	0	0	0	0	0	0	0	0	0	0	0	0	0	0	0	0	0	0	0	0	0	0	0	0	0	0	0	0	0	0	0
DHAP_Post-WM	0	3.7	0	0	0	0	0.0991	0.421	0.183	0.319	0	0.19	0.249	0.464	0	0	0.234	0	0	0	0	0	0	0.14	0.219	0.253	0.122	0	0.228	0	0	0
G3P_Post-WM	0	0.497	0	0	0	0	0	0	0	0	0	0	0	0	0	0	0	0	0	0	0	0	0	0	0	0	0	0	0	0	0	0
Glycerophosphate_Post-WM	2.41	14.6	2.3	2.83	5	4.1	3.04	3.68	3.04	7.14	4.96	4.64	3.87	6.84	8.09	3.21	12.1	4.7	5.16	3.01	4.96	2.52	4.26	6.04	4.02	5.4	3.25	4.87	3.43	5.97	2.69	3.61
Decanoate_Post-WM	0	0	0	0	0	0	0	0	0	0	0	0	0	0	0	0	0	0	0	0	0	0	0	0	0	0	0	0	0	0	0	0
2-Quinolinecarboxylate_Post-WM	0	0	0	0	0	0	0	0	0	0	0	0	0	0	0	0	0	0	0	0	0	0	0	0	0	0	0	0	0	0	0	0
N-Acetylleucine_Post-WM	0	0	0	0.0238	0	0	0	0	0	0.0423	0	0	0	0	0	0	0	0	0	0	0	0	0	0	0	0	0	0	0	0	0	0
Phenyl phosphate_Post-WM	0	0	0	0	0	0	0	0	0	0	0	0	0	0	0	0	0	0	0	0	0	0	0	0	0	0	0	0	0	0	0	0
trans-Aconitate_Post-WM	0	0	0	0	0	0	0	0	0	0	0	0	0	0	0	0	0	0	0	0	0	0	0	0	0	0	0	0	0	0	0	0
cis-Aconitate_Post-WM	0.0649	0.175	0.0947	0.062	0.135	0.11	0.0557	0	0.101	0.0668	0.0635	0.131	0.0825	0.0709	0.42	0.0981	0.152	0.0921	0.0607	0.0722	0.0911	0.0387	0.106	0.17	0.0776	0.141	0	0.175	0.108	0.161	0.0176	0.0422
Shikimate_Post-WM	0	0	0	0	0	0	0	0	0.224	0.539	0	0	0	0	0	0	0.184	0	0	0	0	0	0	0	0	0	0	0	0	0	0	0
N-Acetylaspartate_Post-WM	0.265	0.202	0.982	0.229	1.62	0.33	0.541	0.185	0.23	0.561	0.187	0.523	0.396	0.193	1.52	0.601	0.49	0.625	0.156	0.552	0.314	0.157	0.699	0.671	0.281	1.17	0.165	2.13	0.257	1.99	0.164	0.196
Carbamoylaspartate_Post-WM	0	0	0	0	0	0	0	0	0	0.192	0	0	0	0	0	0.0687	0	0	0	0	0	0	0	0	0	0	0	0	0	0.157	0	0
Allantoate_Post-WM	0	0	0	0	0	0	0	0	0	0	0	0	0	0	0	0	0	0	0	0	0	0	0	0	0	0	0	0	0	0	0	0
2-Isopropylmalate_Post-WM	0.134	0.0808	0.0622	0.0958	0.0922	0.0385	0.0636	0.042	0.0669	0.436	0.335	0.146	0.153	0.0642	0.044	0.118	0.259	0.0831	0.157	0.103	0.0876	0.116	0.0535	0.0705	0.359	0.157	0.0624	0.0389	0.0733	0.212	0.0406	0.0501
N-Formylmethionine_Post-WM	0	0	0	0	0	0	0	0	0	0	0	0	0	0	0	0	0	0	0	0	0	0	0	0	0	0	0	0	0	0	0	0
Hippurate_Post-WM	0	0	0	0	0	0	0	0	0	0	0	0	0	0	0	0	0	0	0	0	0	0	0	0	0	0	0	0	0	0	0	0
Homovanillate_Post-WM	0.428	0.812	0.895	0.741	0.568	0.649	1.15	0.334	0.66	1.94	0.77	0.824	0.624	0.587	0.457	0.66	0.525	0.865	0.645	0	0.669	0.738	0.844	0.995	0.626	0.917	0	0	0.785	0.789	0.404	0.331

4-Pyridoxate_Post-WM	0	0	0	0	0	0	0	0	0	0	0	0	0	0	0	0	0	0	0	0	0	0	0	0	0	0	0	0	0	0	0	0
Serine O-sulfate_Post-WM	0	0	0	0	0	0	0	0	0	0	0	0	0	0	0	0	0	0	0	0	0	0	0	0	0	0	0	0	0	0	0	0
O-Phosphoserine_Post-WM	0	0	0	0	0	0	0	0	0	0.147	0.126	0	0	0	0	0.0725	0	0.0881	0.118	0	0	0	0	0.091	0.076	0.118	0.086	0	0.0763	0.139	0	0
3PG + 2PG_Post-WM	0.403	1.93	0.234	0.181	0.464	0.33	0.385	0.875	0.483	0.505	0.872	0.673	0.702	0.634	4.2	0.34	2.67	0.798	0.633	0.397	0.564	0.501	0.149	0.739	0.711	0.646	0.603	0.893	0.466	0.532	0.81	0.946
Undecanoate_Post-WM	0	0	0	0	0	0	0	0	0	0	0	0	0	0	0	0	0	0	0	0	0	0	0	0	0	0	0	0	0	0	0	0
Azelate_Post-WM	0.105	0.141	0.106	0.115	0.144	0.228	0.163	0.215	0.104	0.0675	0.113	0.108	0.104	0.0763	0.107	0.0868	0.123	0.16	0.117	0.123	0.0946	0.084	0.0934	0.132	0.152	0.159	0.182	0.125	0.109	0.189	0.149	0.159
10-Hydroxydecanoate_Post-WM	0	0	0	0	0	0	0	0	0	0	0	0	0	0	0	0	0	0	0	0	0	0	0	0	0	0	0	0	0	0	0	0
N-Acetylglutamate_Post-WM	0	0.083	0.0641	0	0.103	0	0.053	0.0539	0	0.146	0.0782	0.0508	0.0616	0	0	0.0397	0.118	0.0918	0.0709	0.0411	0	0.0497	0	0.0833	0.0687	0.075	0	0.0525	0.0504	0.0904	0.0416	0
N-Carbamylglutamate_Post-WM	0	0	0	0	0	0	0	0	0	0	0	0	0	0	0	0	0	0	0	0	0	0	0	0	0	0	0	0	0	0	0	0
N-Acetylmethionine_Post-WM	0	0	0	0	0	0	0	0	0	0	0	0	0	0	0	0	0	0	0	0	0	0	0	0	0	0	0	0	0	0	0	0
Isocitrate_Post-WM	0.172	0.298	0.132	0.189	0.215	0.218	0	0	0	0.133	0	0.434	0	0	0.615	1.95	0.372	0.239	0	0.18	0	0	0.283	0.222	0	0.317	0	0.284	0.282	0.295	0	0
Citrate_Post-WM	1.89	2.99	0.968	1.08	2.47	2.92	1.01	0.539	0.785	0.625	0.897	3.29	1.11	1.66	9.03	1.52	3.98	1.52	0.711	1.83	0.907	0.351	2.17	3.85	1.97	4	0.282	6.23	4.43	3.67	0.336	0.389
Quinate_Post-WM	0.969	0	0	0	0.229	0.0901	0	0	0	0	0	0	0	0.334	0	0	0	0	0.483	0	0	0	0	0	0	0.45	0	0	0	0	0	0
Phenaceturate_Post-WM	0.0773	0	0	0	0	0	0	0	0	0	0	0	0	0	0	0	0	0	0	0	0	0	0	0.0969	0	0	0	0	0	0	0	0
Glucuronate_Post-WM	0	0.193	0	0	0.159	0	0	0	0	0	0	0	0.12	0	0	0	0	0	0	0	0.137	0	0	0	0	0	0	0	0	0	0	0
trans-4-Hydroxy-3-methoxycinnamate_Post-WM	0	0	0	0	0	0	0	0	0	0	0	0	0	0	0	0	0	0	0	0	0	0	0	0	0	0	0	0	0	0	0	0
o-Hydroxyhippurate_Post-WM	0	0	0	0	0	0	0	0	0	0	0	0	0	0	0	0	0	0	0	0	0	0	0	0	0	0	0	0	0	0	0	0
Gluconate_Post-WM	0	0	0	0	0	0	0	0	0	0	0	0	0	0	0	0	0	0	0	0	0	0	0	0	0	0	0	0	0	0	0	0
Syringate_Post-WM	0.418	0	0.561	0.415	0.446	0.391	0.396	0	0.382	0.5	0.323	0.339	0.526	0.384	0.205	0.444	0	0.465	0	0.523	0	0.482	0.463	0	0.52	0.548	0.362	0	0.503	0.316	0.432	0.455
4-Hydroxy-3-methoxymandelate_Post-WM	0	0	0.49	0.344	0.125	0	0	0	0.33	0.807	0	0.27	0.295	0	0	0.348	0	0.355	0	0	0.246	0	0.379	0.259	0.358	0.39	0	0	0.248	0.253	0	0
Dodecanoate_Post-WM	0	0	0	0	0	0	0	0	0	0	0	0	0	0	0	0	0	0	0	0	0	0	0	0	0	0	0	0	0.179	0	0	0
Cysteine S-sulfate_Post-WM	0	0	0	0	0	0	0	0	0	0	0	0	0	0	0	0	0	0	0	0	0	0	0	0	0	0	0	0	0	0	0	0
Sebacate_Post-WM	0	0	0	0	0	0	0.0127	0	0	0	0	0	0	0	0	0	0	0	0	0	0	0	0	0	0	0	0.0226	0	0	0.0187	0.015	0
3-Indolebutyrate_Post-WM	0	0	0	0	0	0	0	0	0	0	0	0	0	0	0	0	0	0	0	0	0	0	0	0	0	0	0	0	0	0	0	0
Xanthurenate_Post-WM	0	0	0	0	0	0	0	0	0	0	0	0	0	0	0	0	0	0	0	0	0	0	0	0	0	0	0	0	0	0	0	0
N-Acetylphenylalanine_Post-WM	0	0	0	0	0	0	0	0	0	0	0	0	0	0	0	0	0	0	0	0	0	0	0	0	0	0	0	0	0	0	0	0
Benzylsuccinate_Post-WM	0	0	0	0	0	0	0	0	0	0	0	0	0	0	0	0	0	0	0	0	0	0	0	0	0	0	0	0	0	0	0	0
Saccharate_Post-WM	0	0	0	0	0	0	0	0	0	0	0	0	0	0	0	0	0	0	0	0	0	0	0	0	0	0	0	0	0	0	0	0
Mucate_Post-WM	0	0	0	0	0	0	0	0	0	0	0	0	0	0	0	0	0	0	0	0	0	0	0	0	0	0.354	0	0	0.064	0	0	0
3-Indoxyl sulfate_Post-WM	0	0	0	0	0	0	0	0	0	0	0	0	0	0	0	0	0	0	0	0	0	0	0	0	0	0	0	0	0	0	0	0
2-Deoxyribose 1-phosphate_Post-WM	0	0	0	0	0	0	0	0	0	0	0	0	0	0	0	0	0	0	0	0	0	0	0	0	0	0	0	0	0	0	0	0
Pantothenate_Post-WM	0	0	0.0964	0	0	0	0	0	0	0	0	0.0716	0.0767	0	0	0.137	0	0.102	0	0	0	0	0	0.173	0	0.13	0.0831	0	0	0	0.0927	0
Sinapate_Post-WM	0	0	0	0	0	0	0	0	0	0	0	0	0	0	0	0	0	0	0	0	0	0	0	0	0	0	0	0	0	0	0	0
Porphobilinogen_Post-WM	0	0	0	0	0	0	0	0	0	0	0	0	0	0	0	0	0	0	0	0	0	0	0	0	0	0	0	0	0	0	0	0
Ru5P_Post-WM	0.556	2.98	0.428	0.316	0.441	0.628	0.524	1.68	0.653	0.41	1	0.651	0.561	0.565	1.54	0.498	0.747	0.667	1.19	0.461	1.21	0.655	0.323	1.27	0.964	1	0.519	0.874	0.635	0.665	1.08	1.23
R5P_Post-WM	0	1.2	0	0	0	0	0	0	0	0	0	0	0	0	0	0	0	0	0	0	0	0	0	0	0	0	0	0	0	0	0	0
Dodecanedioate_Post-WM	0	0	0	0	0	0	0	0	0	0	0	0	0	0	0	0	0	0	0	0	0	0	0	0	0	0.011	0	0	0.0114	0	0	0
2-Deoxyglucose 6-phosphate_Post-WM	0	0	0	0	0	0	0	0	0	0	0	0	0	0	0	0	0	0	0	0	0	0	0	0	0	0	0	0	0	0	0	0
Biotin_Post-WM	0.17	0.53	0.263	0.117	0.266	0	0	0	0	0.487	0	0	0.151	0	0	0.166	0.391	0.17	0	0.152	0	0.204	0.231	0	0.252	0	0.151	0	0	0.247	0	0.209
Glucosamine 6-phosphate_Post-WM	0	0	0	0	0	0	0	0	0	0	0	0	0	0	0	0	0	0	0	0	0	0	0	0	0	0	0	0	0	0	0	0
G1P_Post-WM	0.165	0.479	0.12	0.16	0.196	0.177	0.138	0.311	0.134	0.483	0.557	0.599	0.34	0.296	0.367	0.125	1.06	0.334	0.607	0.167	0.259	0.167	0.109	0.241	0.406	0.391	0.157	0.193	0.435	0.267	0.114	0.137
F6P_Post-WM	0.175	0.782	0.217	0.151	0.485	0.259	0.207	0.464	0.33	0.61	0.331	0.41	0.514	0.485	0.512	0.134	0.604	0.427	0.432	0.263	0.505	0.286	0.141	0.257	0.61	0.482	0.248	0.269	0.331	0.446	0.19	0.334
G6P_Post-WM	0.773	3.77	0.753	0.508	1.7	0.989	0.838	2.05	1.09	2.62	1.53	1.55	1.97	2.29	2.78	0.677	3.77	2.03	1.94	0.863	1.94	1.1	0.584	1.06	1.79	1.68	0.759	1.37	1.17	1.37	0.731	1.16
Sorbitol 6-phosphate_Post-WM	0	0	0	0	0	0	0	0	0	0	0	0	0	0	0	0	0.201	0	0	0	0	0	0	0	0	0	0	0	0	0	0	0
2,3-DPG_Post-WM	0	0	0	0	0	0	0	0	0	0	0	0	0	0	0	0	0	0	0	0	0	0	0	0	0	0	0	0	0	0	0	0
Galacturonate 1-phosphate_Post-WM	0	0.0864	0.0701	0	0	0	0	0	0	0.139	0	0.1	0.0643	0	0.198	0	0.214	0.102	0	0	0	0	0.0602	0	0.106	0.108	0	0.184	0	0.207	0	0
6-Phosphogluconate_Post-WM	0	0	0.132	0	0	0	0.0713	0.17	0	0.192	0	0.101	0	0	0.0985	0	0.08	0	0.269	0.0664	0.153	0	0	0.212	0.0735	0.188	0	0.125	0.225	0.226	0.0964	0
S7P_Post-WM	0	0.82	0.246	0	0	0.276	0	0.322	0.555	0.628	0	0.516	0.239	0	0.774	0	1.22	0.56	0.482	0	0.386	0	0	0	0.421	0.458	0.312	0	0.292	0.456	0	0
Argininosuccinate_Post-WM	0	0	0	0	0	0	0	0	0	0	0	0	0	0	0	0	0	0	0	0	0	0	0	0	0	0	0	0	0	0	0	0
N-Acetylmuramate_Post-WM	0	0	0.0896	0	0	0	0	0	0	0	0	0	0	0	0	0	0	0	0	0	0	0	0	0	0.113	0	0	0	0	0	0	0
N-Acetylglucosamine 1-phosphate_Post-WM	0	0.188	0	0.0751	0.143	0	0.0849	0	0.137	0.279	0.26	0.739	0	0.13	0	0	0.406	0.139	0.156	0.0803	0.13	0.142	0.0591	0	0.137	0.145	0.0606	0.0956	0.395	0.133	0.0643	0
N-Acetylglucosamine 6-phosphate_Post-WM	0.0692	0.131	0	0	0.127	0	0	0	0	0.272	0	0.0905	0.0857	0.116	0	0	0.168	0.138	0	0.0552	0.111	0	0	0	0.134	0.108	0	0.15	0.1	0	0	0.088
2',3'-cCMP_Post-WM	0	0	0	0	0	0	0	0	0	0	0	0	0	0	0	0	0	0	0	0	0	0	0	0	0	0	0	0	0	0	0	0
dCMP_Post-WM	0	0	0	0	0	0	0	0	0	0	0	0	0	0	0	0	0	0	0	0	0	0	0	0	0	0	0	0	0	0	0	0
dUMP_Post-WM	0	0	0	0	0	0	0	0	0	0	0	0	0	0	0	0	0	0	0	0	0	0	0	0	0	0	0	0	0	0	0	0
N-Acetylneuraminate_Post-WM	39	16	7.46	7.82	4.89	10	4.86	13.3	12.2	51.5	41.7	42.7	5.09	19	3.73	40.4	14.8	27.7	20.4	8.76	29.7	10.1	13.5	29.3	5.18	23.3	5.38	4.68	24.2	10.4	15.2	12.2
Ribulose 1,5-diphosphate_Post-WM	0	0	0	0	0	0	0	0	0	0	0	0	0	0	0	0	0	0	0	0	0	0	0	0	0	0	0	0	0	0	0	0
3',5'-Cyclic dAMP_Post-WM	0	0	0	0	0	0	0	0	0	0	0	0	0	0	0	0	0	0	0	0	0	0	0	0	0	0	0	0	0	0	0	0
dTMP_Post-WM	0	0	0	0	0	0	0	0	0	0	0	0	0	0	0	0	0	0	0	0	0	0	0	0	0	0	0	0	0	0	0	0
CMP_Post-WM	0	0.736	0	0	0.302	0	0	0.285	0	0	0.116	0.134	0.222	0.272	0.658	0	0.552	0.211	0.16	0.14	0.175	0.158	0.127	0.158	0.22	0.261	0.292	0.502	0.126	0.109	0.208	0.461
UMP_Post-WM	0	0.405	0	0.12	0.278	0	0	0.343	0.19	0.0895	0.267	0.15	0.466	0.307	0.6	0	0.404	0.277	0.31	0.193	0.236	0.177	0.178	0.229	0.267	0.37	0.129	0.372	0	0	0.164	0.383
cAMP_Post-WM	0	0	0	0	0	0	0	0	0	0	0	0	0	0	0	0	0	0	0	0	0	0	0	0	0	0	0	0	0	0	0	0
cIMP_Post-WM	0	0	0	0	0	0	0	0	0	0	0	0	0	0	0	0	0	0	0	0	0	0	0	0	0	0	0	0	0	0	0	0
dAMP_Post-WM	0	0	0	0	0	0	0	0	0	0	0	0	0	0	0	0	0	0	0	0	0	0	0	0	0	0	0	0	0	0	0	0
F1,6P_Post-WM	0.0564	0.195	0.0547	0	0	0	0	0.0898	0	0	0	0.0528	0.0911	0	0.116	0	0	0	0	0	0	0	0	0.0443	0.0465	0.0714	0	0	0	0	0	0
cGMP_Post-WM	0	0	0	0	0	0	0	0	0	0	0	0	0	0	0	0	0	0	0	0	0	0	0	0	0	0	0	0	0	0	0	0
3'-AMP_Post-WM	0	0.419	0	0	0	0	0	0	0	0	0	0	0	0	0	0	0.231	0	0	0	0	0	0	0	0	0	0.116	0.27	0	0	0.117	0.247
AMP_Post-WM	0.0852	1.42	0	0	0.833	0	0.186	0.728	0.135	0	0	0	1.55	0.295	1.64	0	0	0	0.189	0.104	0.456	0	0	0	0.132	0.149	0	0.846	0.13	0	0.214	0.823
IMP_Post-WM	0	0	0	0	0	0	0	0	0	0	0	0	0.198	0	0	0	0	0	0	0	0	0	0	0	0	0	0	0	0	0	0	0
Prostaglandin E2_Post-WM	0	0	0	0	0	0	0	0	0	0	0	0	0	0	0	0	0	0	0	0	0	0	0	0	0	0	0	0	0	0	0	0
Prostaglandin F2α_Post-WM	0	0	0	0	0	0	0	0	0	0	0	0	0	0	0	0	0	0	0	0	0	0	0	0	0	0	0	0	0	0	0	0
GMP_Post-WM	0	0.167	0	0	0.18	0	0	0.203	0	0	0	0	0.486	0	0	0	0	0	0	0	0	0	0	0	0.0853	0	0	0.187	0	0	0	0.203
XMP_Post-WM	0	0	0	0	0	0	0	0	0	0	0	0	0	0	0	0	0	0	0	0	0	0	0	0	0	0	0	0	0	0	0	0
Orotidine 5'-monophosphate_Post-WM	0	0	0	0	0	0	0	0	0	0	0	0	0	0	0	0	0	0	0	0	0	0	0	0	0	0	0	0	0	0	0	0
Digalacturonate_Post-WM	0	0	0	0	0	0	0	0	0	0	0	0	0	0	0	0	0	0	0	0	0	0	0	0	0	0	0	0	0	0	0	0
NADPH_Post-WM	0	0	0	0	0	0	0	0	0	0	0	0	0	0	0	0	0	0	0	0	0	0	0	0	0	0	0	0	0	0	0	0
CoA_Post-WM	0	0	0	0	0	0	0	0	0	0	0	0	0	0	0	0	0	0	0	0	0	0	0	0	0	0	0	0	0	0	0	0
dCDP_Post-WM	0	0	0	0	0	0	0	0	0	0	0	0	0	0	0	0	0	0	0	0	0	0	0	0	0	0	0	0	0	0	0	0
dUDP_Post-WM	0	0	0	0	0	0	0	0	0	0	0	0	0	0	0	0	0	0	0	0	0	0	0	0	0	0	0	0	0	0	0	0
PRPP_Post-WM	0	0	0	0	0	0	0	0	0	0	0	0	0	0	0	0	0	0	0	0	0	0	0	0	0	0	0	0	0	0	0	0
dTDP_Post-WM	0	0	0	0	0	0	0	0	0	0	0	0	0	0	0	0	0	0	0	0	0	0	0	0	0	0	0	0	0	0	0	0
CDP_Post-WM	0	0	0	0	0	0	0	0	0	0	0	0	0	0	0	0	0	0	0	0	0	0	0	0	0	0	0	0	0	0	0	0
UDP_Post-WM	0	0	0	0	0	0	0	0	0	0	0	0	0	0	0	0	0	0	0	0	0	0	0	0	0	0	0	0	0	0	0	0
Acetyl CoA_Post-WM	0	0	0	0	0	0	0	0	0	0	0	0	0	0	0	0	0	0	0	0	0	0	0	0	0	0	0	0	0	0	0	0
Cholate_Post-WM	0	0	0	0	0	0	0	0	0	0	0	0	0	0	0	0	0	0	0	0	0	0	0	0	0	0	0	0	0	0	0	0
dADP_Post-WM	0	0	0	0	0	0	0	0	0	0	0	0	0	0	0	0	0	0	0	0	0	0	0	0	0	0	0	0	0	0	0	0
P1, P4-Di(adenosine-5') tetraphosphate_Post-WM	0	0	0	0	0	0	0	0	0	0	0	0	0	0	0	0	0	0	0	0	0	0	0	0	0	0	0	0	0	0	0	0
Isobutyryl CoA_Post-WM	0	0	0	0	0	0	0	0	0	0	0	0	0	0	0	0	0	0	0	0	0	0	0	0	0	0	0	0	0	0	0	0
Trehalose 6-phosphate_Post-WM	0	0	0	0	0	0	0	0	0.132	0	0.152	0	0.172	0	2.25	0	0	0	0	0	0	0	0	0	0.0926	0.0666	0	0	0.0756	0	0.109	0
Malonyl CoA_Post-WM	0	0	0	0	0	0	0	0	0	0	0	0	0	0	0	0	0	0	0	0	0	0	0	0	0	0	0	0	0	0	0	0
Adenosine 5'-phosphosulfate_Post-WM	0	0	0	0	0	0	0	0	0	0	0	0	0	0	0	0	0	0	0	0	0	0	0	0	0	0	0	0	0	0	0	0
Adenosine 3',5'-diphosphate_Post-WM	0	0	0	0	0	0	0	0	0	0	0	0	0	0	0	0	0	0	0	0	0	0	0	0	0	0	0	0	0	0	0	0

ADP_Post-WM	0	0	0	0	0	0	0	0	0	0	0	0	0	0	0	0	0	0	0	0	0	0	0	0	0	0	0	0	0	0	0	0
IDP_Post-WM	0	0	0	0	0	0	0	0	0	0	0	0	0	0	0	0	0	0	0	0	0	0	0	0	0	0	0	0	0	0	0	0
Succinyl CoA_Post-WM	0	0	0	0	0	0	0	0	0	0	0	0	0	0	0	0	0	0	0	0	0	0	0	0	0	0	0	0	0	0	0	0
Folate_Post-WM	0	0	0	0	0	0	0	0	0	0	0	0	0	0	0	0	0	0	0	0	0	0	0	0	0	0	0	0	0	0	0	0
GDP_Post-WM	0	0	0	0	0	0	0	0	0	0	0	0	0	0	0	0	0	0	0	0	0	0	0	0	0	0	0	0	0	0	0	0
Adenylosuccinate_Post-WM	0	0	0	0	0	0	0	0	0	0	0	0	0	0	0	0	0	0	0	0	0	0	0	0	0	0	0	0	0	0	0	0
Glycocholate_Post-WM	0	0	0	0	0	0	0	0	0	0	0	0	0	0	0	0	0	0	0	0	0	0	0	0	0	0	0	0	0	0	0	0
dCTP_Post-WM	0	0	0	0	0	0	0	0	0	0	0	0	0	0	0	0	0	0	0	0	0	0	0	0	0	0	0	0	0	0	0	0
dUTP_Post-WM	0	0	0	0	0	0	0	0	0	0	0	0	0	0	0	0	0	0	0	0	0	0	0	0	0	0	0	0	0	0	0	0
dTTP_Post-WM	0	0	0	0	0	0	0	0	0	0	0	0	0	0	0	0	0	0	0	0	0	0	0	0	0	0	0	0	0	0	0	0
CTP_Post-WM	0	0	0	0	0	0	0	0	0	0	0	0	0	0	0	0	0	0	0	0	0	0	0	0	0	0	0	0	0	0	0	0
UTP_Post-WM	0	0	0	0	0	0	0	0	0	0	0	0	0	0	0	0	0	0	0	0	0	0	0	0	0	0	0	0	0	0	0	0
dATP_Post-WM	0	0	0	0	0	0	0	0	0	0	0	0	0	0	0	0	0	0	0	0	0	0	0	0	0	0	0	0	0	0	0	0
dITP_Post-WM	0	0	0	0	0	0	0	0	0	0	0	0	0	0	0	0	0	0	0	0	0	0	0	0	0	0	0	0	0	0	0	0
ATP_Post-WM	0	0	0	0	0	0	0	0	0	0	0	0	0	0	0	0	0	0	0	0	0	0	0	0	0	0	0	0	0	0	0	0
dGTP_Post-WM	0	0	0	0	0	0	0	0	0	0	0	0	0	0	0	0	0	0	0	0	0	0	0	0	0	0	0	0	0	0	0	0
ITP_Post-WM	0	0	0	0	0	0	0	0	0	0	0	0	0	0	0	0	0	0	0	0	0	0	0	0	0	0	0	0	0	0	0	0
Taurocholate_Post-WM	0	0	0	0	0	0	0	0	0	0	0	0	0	0	0	0	0	0	0	0	0	0	0	0	0	0	0	0	0	0	0	0
GTP_Post-WM	0	0	0	0	0	0	0	0	0	0	0	0	0	0	0	0	0	0	0	0	0	0	0	0	0	0	0	0	0	0	0	0
XTP_Post-WM	0	0	0	0	0	0	0	0	0	0	0	0	0	0	0	0	0	0	0	0	0	0	0	0	0	0	0	0	0	0	0	0
CDP-choline_Post-WM	0	0	0	0	0	0	0	0	0	0	0	0	0	0	0	0	0	0	0	0	0	0	0	0	0	0	0	0	0	0	0	0
ADP-ribose_Post-WM	0	0	0	0	0	0	0	0	0	0	0	0	0	0	0	0	0	0	0	0	0	0	0	0	0	0	0	0	0	0	0	0
TDP-glucose_Post-WM	0	0	0	0	0	0	0	0	0	0	0	0	0	0	0	0	0	0	0	0	0	0	0	0	0	0	0	0	0	0	0	0
UDP-glucose_Post-WM	0	0.249	0.0835	0	0.118	0	0	0.121	0.0625	0	0	0	0.0829	0.0583	0.174	0	0	0	0	0	0.139	0	0	0	0.0918	0	0	0	0	0	0.0462	0.0788
UDP-glucuronate_Post-WM	0	0.058	0	0	0	0	0	0	0	0	0	0	0	0	0	0	0	0	0	0	0	0	0	0	0	0	0	0	0	0	0	0
ADP-glucose_Post-WM	0	0	0	0	0	0	0	0	0	0	0	0	0	0	0	0	0	0	0	0	0	0	0	0	0	0	0	0	0	0	0	0
GDP-mannose_Post-WM	0	0	0	0	0	0	0	0	0	0	0	0	0	0	0	0	0	0	0	0	0	0	0	0	0	0	0	0	0	0	0	0
UDP-*N*-acetylglucosamine_Post-WM	0	0.355	0	0	0.256	0	0	0.298	0	0	0	0	0.212	0	0	0	0	0	0	0	0.221	0	0	0	0	0	0	0	0	0	0	0
CMP-*N*- acetylneuraminate_Post-WM	0	0	0	0	0	0	0	0	0	0	0	0	0	0	0	0	0	0	0	0	0	0	0	0	0	0	0	0	0	0	0	0
NAD+_Post-WM	0	0	0	0	0	0	0	0	0	0	0	0	0	0	0	0	0	0	0	0	0	0	0	0	0	0	0	0	0	0	0	0
Deamido-NAD+_Post-WM	0	0	0	0	0	0	0	0	0	0	0	0	0	0	0	0	0	0	0	0	0	0	0	0	0	0	0	0	0	0	0	0
NADH_Post-WM	0	0	0	0	0	0	0	0	0	0	0	0	0	0	0	0	0	0	0	0	0	0	0	0	0	0	0	0	0	0	0	0
NADP+_Post-WM	0	0	0	0	0	0	0	0	0	0	0	0	0	0	0	0	0	0	0	0	0	0	0	0	0	0	0	0	0	0	0	0
FAD_Post-WM	0	0	0	0	0	0	0	0	0	0	0	0	0	0	0	0	0	0	0	0	0	0	0	0	0	0	0	0	0	0	0	0
LF_Power_Rest	805	2960	410	541	281	6250	96.4	174	404	326	2970	387	449	1040	406	1660	257	1720	523	143	348	802	848	161	142	861	2130	178	1110	324	44.8	454
HF_Power_Rest	504	1450	275	305	103	991	36.8	39.2	364	267	733	85.6	918	493	256	670	78.9	592	755	88.3	136	234	746	114	95.2	258	652	144	374	138	236	177
LF/HF_Rest	1.63	1.99	1.63	1.67	2.76	6.32	2.77	4.42	1.11	1.25	4.21	4.27	0.564	2.15	3.17	2.46	3.77	3.39	0.663	1.95	2.47	3.46	1.12	1.44	1.5	3.62	3.36	1.19	2.99	2.28	0.197	2.46
SDNN_Rest	47.1	90.4	39.1	51	43.4	84.8	30.2	19.5	39.3	46.9	97	36.3	49.6	54.6	33.5	58.4	21.7	61	48.2	38.1	38.1	51.2	55.5	25.2	25.2	41.9	64.4	20.7	60.2	37.3	32.4	35.2
HeartRate_Rest	44.7	49.9	52.8	50.1	52.8	44.4	42.8	39	44.2	37.7	48.6	44.7	51.2	43	47.2	52.1	45	49.5	51.9	35.2	55.9	52.2	51.8	52.9	46.3	49.8	54.1	36	50.5	54.4	54.6	36.4
BodySurfaceTemp_Rest	31.5	29	29.3	32.6	25.4	31	31.6	30	29.3	31.1	26.8	30.7	30.2	30.5	28.1	30.2	30.9	28.7	28.4	32.2	29.7	27.6	29.6	28.1	29.9	32	28	32.2	27	30.9	28.4	29.2
LF_Power_Post-UK	2450	1090	1190	431	162	134	94.3	126	300	729	647	193	1510	615	161	653	196	602	1070	174	178	487	644	1140	321	297	1280	390	1370	494	170	434
HF_Power_Post-UK	1570	803	1810	221	105	330	42.1	53	140	357	1350	403	843	628	44.8	683	187	723	571	77.6	350	161	387	573	64.9	235	754	303	395	333	258	238
LF/HF_Post-UK	1.57	1.54	0.909	2.11	1.54	0.408	2.67	2.44	2.54	2.24	0.479	0.88	2.29	1.02	3.37	0.99	1.06	0.742	2.17	2.9	0.603	2.94	1.94	2.21	5.03	1.33	1.67	1.64	4.46	1.48	0.681	1.81
SDNN_Post-UK	81	63.2	52.9	56.2	32.8	29	24.7	35.7	34.9	44.2	59.8	40.5	83.5	55.8	24.7	47.7	32.2	49.1	60.9	31.4	65.3	35.1	87.3	82.5	51.1	48.8	66.3	38.3	73.1	41.9	45.1	34.4
HeartRate_Post-UK	44.4	49.7	54.2	47.3	51.2	39.5	40.5	38.6	40.7	40.5	54.2	45	50.6	44	50	54.1	41.6	49.7	55.9	35.7	48.4	51.2	47.4	54.3	45.8	50.7	52.4	35.9	50.2	57.8	53.2	39.5
BodySurfaceTemp_Post-UK	31.8	29.8	29.9	33.6	26.1	31.4	32.2	30.3	29.9	31.2	27.8	31.2	30.9	30.5	29.5	31	32.5	29.6	28.9	32.4	30.6	28.3	29.8	29.4	30.2	32.4	28.7	33.7	28.2	31.2	29.8	29.6
LF_Power_Recover	611	3350	1020	851	132	3570	67.1	377	314	278	2090	306	600	379	88.8	824	181	661	340	116	72.7	732	477	1140	459	413	1800	360	1020	234	53.2	295
HF_Power_Recover	484	1920	1500	321	70.8	982	9.09	110	207	281	766	71.9	952	464	30.9	741	88.4	150	450	20.9	125	148	1010	222	155	385	558	166	367	178	272	181
LF/HF_Recover	1.24	1.77	1.65	2.67	1.71	3.62	8.26	4.02	1.75	0.921	3.59	4.15	1.08	0.85	2.85	1.14	2.51	4.32	0.762	5.04	0.57	5.3	0.526	5.02	4.62	1.17	3.74	2.41	2.82	1.38	0.223	1.68
SDNN_Recover	46.8	77.3	51.9	50.7	26.7	72.2	27.5	28.4	34.2	34.5	76.2	36.1	76.8	62.6	42.9	60.3	28	63.2	42.8	35.4	24	43.9	58.8	47.9	40.2	53.1	64.7	27	59	43.4	31.4	28.1
HeartRate_Recover	43.7	46	56.8	54.1	50.3	45.1	41.8	39	43.2	38.5	50.2	42.4	50.4	44.1	37	51.5	41.1	32.8	54.6	36.1	57.5	53.2	54	52.4	44.6	47.1	53	36.5	48.9	57.8	51.7	38.2
BodySurfaceTemp_Recover	31.8	29.9	30.2	33.7	26.2	31.4	32.2	30.6	30	31.1	27.8	31.2	30.8	30.5	29.3	30.9	32.3	29.6	29.1	32.5	30.9	29.1	29.9	29.5	30.3	32.5	28.8	33.4	28.4	31.4	30.1	29.7
LF_Power_Post-WM	517	448	174	896	187	308	100	175	337	266	1600	243	1760	853	26.1	727	59.3	182	218	91.3	284	232	992	1270	291	342	720	620	986	435	168	247
HF_Power_Post-WM	361	1830	217	221	105	675	9.72	96.9	100	230	1850	146	2720	847	28.9	594	84	99.2	317	29.7	279	159	757	383	50	330	511	418	486	149	208	159
LF/HF_Post-WM	1.42	0.289	0.925	4.16	1.74	0.519	10.8	2.04	3.18	1.14	0.921	2.62	0.551	1.1	1.01	1.26	0.784	1.69	0.686	2.79	0.949	1.53	1.67	3.37	5.49	1.25	1.88	1.32	2.35	3.01	0.944	1.55
SDNN_Post-WM	50	68.9	34.3	56.9	58.3	44.3	33.4	28	49.2	33.5	67.5	38.9	88.9	70.1	30.1	71.1	24.5	44.6	32.8	26.8	67.7	39.3	83	74.7	52.8	50.9	86.1	37.6	70.9	49.2	46.9	25.4
HeartRate_Post-WM	42.3	50.9	55.4	47.7	44.3	40.5	39.6	37.9	39.4	37.9	57.4	43.1	51.1	39.3	37.6	51.9	41.8	30.4	52.7	35.6	52.1	50.6	51.6	51.4	43.3	47	50.3	36.1	48.6	57	51.3	38.7
BodySurfaceTemp_Post-WM	31.9	30.2	30.4	33.6	26.2	31.6	32.3	30.8	30.2	31	27.9	31.3	30.9	30.8	29.3	31	31.9	29.6	29.1	32.4	30.9	29.3	30.1	29.8	30.8	32.8	29.2	33.1	28.8	31.5	30.3	29.7
